# Chemical evidence for the tradeoff-in-the-nephron hypothesis to explain secondary hyperparathyroidism

**DOI:** 10.1371/journal.pone.0272380

**Published:** 2022-08-01

**Authors:** Kenneth R. Phelps, Darren E. Gemoets, Peter M. May

**Affiliations:** 1 Research Service, Stratton Veterans’ Affairs Medical Center, Albany, NY, Uniyed States of America; 2 Department of Medicine, Albany Medical College, Albany, NY, Uniyed States of America; 3 Department of Chemistry, Murdoch University, Murdoch, WA, Australia; Anatomy, SWITZERLAND

## Abstract

**Background:**

Secondary hyperparathyroidism (SHPT) complicates advanced chronic kidney disease (CKD) and causes skeletal and other morbidity. In animal models of CKD, SHPT was prevented and reversed by reduction of dietary phosphate in proportion to GFR, but the phenomena underlying these observations are not understood. The tradeoff-in-the-nephron hypothesis states that as GFR falls, the phosphate concentration in the distal convoluted tubule ([P]_DCT_]) rises, reduces the ionized calcium concentration in that segment ([Ca^++^]_DCT_), and thereby induces increased secretion of parathyroid hormone (PTH) to maintain normal calcium reabsorption. In patients with CKD, we previously documented correlations between [PTH] and phosphate excreted per volume of filtrate (E_P_/C_cr_), a surrogate for [P]_DCT_. In the present investigation, we estimated [P]_DCT_ from physiologic considerations and measurements of phosphaturia, and sought evidence for a specific chemical phenomenon by which increased [P]_DCT_ could lower [Ca^++^]_DCT_ and raise [PTH].

**Methods and findings:**

We studied 28 patients (“CKD”) with eGFR of 14–49 mL/min/1.73m^2^ (mean 29.9 ± 9.5) and 27 controls (“CTRL”) with eGFR > 60 mL/min/1.73m^2^ (mean 86.2 ± 10.2). In each subject, total [Ca]_DCT_ and [P]_DCT_ were deduced from relevant laboratory data. The Joint Expert Speciation System (JESS) was used to calculate [Ca^++^]_DCT_ and concentrations of related chemical species under the assumption that a solid phase of amorphous calcium phosphate (Ca_3_(PO_4_)_2_ (am., s.)) could precipitate. Regressions of [PTH] on eGFR, [P]_DCT_, and [Ca^++^]_DCT_ were then examined. At filtrate pH of 6.8 and 7.0, [P]_DCT_ was found to be the sole determinant of [Ca^++^]_DCT_, and precipitation of Ca_3_(PO_4_)_2_ (am., s.) appeared to mediate this result. At pH 6.6, total [Ca]_DCT_ was the principal determinant of [Ca^++^]_DCT_, [P]_DCT_ was a minor determinant, and precipitation of Ca_3_(PO_4_)_2_ (am., s.) was predicted in no CKD and five CTRL. In CKD, at all three pH values, [PTH] varied directly with [P]_DCT_ and inversely with [Ca^++^]_DCT_, and a reduced [Ca^++^]_DCT_ was identified at which [PTH] rose unequivocally. Relationships of [PTH] to [Ca^++^]_DCT_ and to eGFR resembled each other closely.

**Conclusions:**

As [P]_DCT_ increases, chemical speciation calculations predict reduction of [Ca^++^]_DCT_ through precipitation of Ca_3_(PO_4_)_2_ (am., s.). [PTH] appears to rise unequivocally if [Ca^++^]_DCT_ falls sufficiently. These results support the tradeoff-in-the-nephron hypothesis, and they explain why proportional phosphate restriction prevented and reversed SHPT in experimental CKD. Whether equally stringent treatment can be as efficacious in humans warrants investigation.

## Introduction

The parathyroid hormone concentration ([PTH]) rises as the glomerular filtration rate (GFR) falls in patients with chronic kidney disease (CKD) [1]. This phenomenon, secondary hyperparathyroidism (SHPT), causes skeletal morbidity and may contribute to other uremic manifestations [2–4]. PTH also raises concentrations of fibroblast growth factor 23, which may exert its own toxic effects [5–7].

During the past 50 years, seven theories have been advanced to explain the pathogenesis of SHPT [8]. The most recent of these, the tradeoff-in-the-nephron hypothesis, attributes SHPT to an increased phosphate concentration in the distal convoluted tubule ([P]_DCT_), where PTH regulates reabsorption of ionized Ca (Ca^++^) [8–11]. According to this hypothesis, high [P]_DCT_ reduces [Ca^++^]_DCT_, and [PTH] rises to maintain Ca^++^ reabsorption at a rate compatible with normocalcemia [8,12,13]. The hypothesis integrates the micropuncture observation that [P]_DCT_ rose in animals with CKD fed a standard diet [9], and explains why dietary restriction or intestinal binding of phosphate prevented, mitigated, or reversed SHPT in animals and humans [14–22]. The term “tradeoff-in-the-nephron” invites a comparison to the original tradeoff hypothesis, which attributed SHPT to an interaction between phosphate and Ca^++^ in plasma [23].

If creatinine clearance (C_cr_) is accepted as a surrogate for GFR, the ratio of the phosphorus excretion rate (E_P_) to C_cr_ quantifies the amount of P excreted per volume of filtrate. Moreover, E_P_/C_cr_ is proportional to [P]_DCT_ if fractional delivery of filtrate to the DCT is assigned a constant value [8,12,13,24,25]. In a cohort of 30 patients with stages G_3_ or G_4_ CKD, significant correlations between [PTH] and E_P_/C_cr_ were demonstrated under multiple conditions, but a chemical mechanism to explain these correlations was not investigated [24].

The Joint Expert Speciation System (JESS) employs a compilation of thousands of equilibria to predict concentrations of ions and other chemical entities under defined conditions [26,27]. In the present study, we deduced concentrations of Ca and P in the DCT ([Ca]_DCT_ and [P]_DCT_) from laboratory measurements and physiologic considerations, and posited evidence-based assumptions concerning other constituents in that segment. With this information, JESS was used to calculate [Ca^++^]_DCT_ under various modeling scenarios, of which the most germane proved to be ones that excluded or included possible precipitation of amorphous calcium phosphate (Ca_3_(PO_4_)_2_ (am., s.)). Regressions of [PTH] on [Ca^++^]_DCT_ were then performed at pH 6.6, 6.8, and 7.0, which are representative values over the documented pH range in the DCT [28]. If precipitation of amorphous calcium phosphate (Ca_3_(PO_4_)_2_ (am., s.)) occurred as filtrate reached saturation with this solid, total [P]_DCT_ reduced [Ca^++^]_DCT_ in advanced CKD to values associated with increased [PTH]. Relationships of [PTH] to [Ca^++^]_DCT_ and to eGFR were virtually identical.

## Methods

### Subjects and laboratory determinations

Between 2010 and 2013, data were collected from 28 control subjects with estimated GFR (eGFR) > 60 mL/min/1.73m^2^ and 30 patients with eGFR 14–49 mL/min/1.73m^2^. GFR was estimated with the 4-variable MDRD formula. All participants were normocalcemic (8.5–10.2 mg/dL in our hospital laboratory). Two patients with CKD were excluded from the present study, one because the serum ultrafilterable calcium concentration ([Ca_uf_]_s_) was reported to be lower than the ionized calcium concentration ([Ca^++^]_s_)–a physiologic impossibility–and one because the association of [PTH] of 169 pg/mL with [Ca^++^]_s_ of 1.35 mM implied autonomy of PTH secretion. A control subject who failed to collect a 24-hour urine specimen was also excluded. The study sample in the present report therefore includes 28 patients with CKD (denoted as “CKD”) and 27 control subjects (denoted as “CTRL”). CKD and CTRL were not matched for age, race, or gender.

The data considered herein were obtained before experimental interventions were initiated [12,13,24,25]. Aliquots of urine, serum, and plasma were obtained at a clinic visit occurring between 8:00 and 10:00 a.m. Subjects performed a urine collection during the 24 hours preceding the visit and were instructed to take no medicines or food after midnight of their appointment day. Serum (s) and urine (u) concentrations of creatinine, calcium, and phosphorus were measured by autoanalyzer. [Ca^++^]_s_, [Ca_uf_]_s_, and plasma [PTH]1–84 ([PTH]) were measured as previously described [12].

### Estimation of total phosphate and calcium concentrations in the DCT

Phosphate and calcium exist in filtrate of the DCT as either free ions (PO_4_^3–^ and Ca^++^) or chemical species derived from the ions by formation reactions. Most derived species, such as H_2_PO_4_^–^, HPO_4_^=^, and CaHPO_4_^0^, exist in solution (*i*.*e*., are dissolved), but solid phases such as brushite and amorphous calcium phosphate may precipitate if their solubility product constants are exceeded. The extent of formation of any chemical species, including precipitates, can be calculated from the total concentrations [P]_DCT_ and [Ca]_DCT_ using thermodynamic relationships and equilibrium constants described in the chemical literature. However, it is necessary in such calculations to stipulate *a priori* which if any precipitates may be formed. Such stipulations are based on certain empirical rules of thumb, particularly Ostwald’s Rule of Stages, as described below.

Total [P]_DCT_ and [Ca]_DCT_ were estimated in this work under the following assumptions: the rate of phosphate delivery to the DCT equals the excretion rate of phosphorus (E_P_) [29]; the rate of calcium delivery to this segment is 10% of the filtration rate of calcium, or 0.1(eGFR)[Ca_uf_]_s_ [30]; and fractional delivery of filtrate (FD_f_) to the DCT is 0.2 in controls and 0.35 in subjects with CKD [31–33]. Accordingly, total [Ca]_DCT_ was computed as 0.1(eGFR)[Ca_uf_]_s_/(0.35)eGFR in CKD and as 0.1[(eGFR)[Ca_uf_]_s_/(0.2)eGFR in CTRL. Similarly, total [P]_DCT_ was calculated as (24h E_P_)/0.35(eGFR) in CKD and as (24h E_P_)/0.2(eGFR) in CTRL. Equations for total [Ca]_DCT_ and [P]_DCT_ thus simplified to 0.1[Ca_uf_]_s_/FD_f_ and (24h E_P_)/{FD_f_(eGFR)}, respectively.

### Estimation of other total concentrations in the DCT

Filtrate pH and total concentrations in the DCT of sodium, potassium, magnesium, chloride, urate, sulfate, citrate, oxalate, bicarbonate, creatinine, and urea were estimated by appropriate combinations of the following: ultrafilterable concentrations in serum; estimated GFR (eGFR); published micropuncture data [28,30–38]; studies of urine dilution with water loading [39]; excretion rates of anions not reabsorbed or secreted in the distal nephron [40–45]; and assumptions concerning FD_f_ at normal and reduced GFR [31–33]. The concentration of ammonium in the DCT was assumed to be negligible. Details concerning assignment of concentrations, including pH, are provided in Supporting Information.

### Determination of [Ca^++^]_DCT_ using JESS

From estimated total concentrations and relevant equilibria, routine chemical speciation models were constructed to solve mass balance equations for each applicable DCT component. The calculated ionic strength of DCT filtrate allowed the ionic activity quotients of all chemical reactions to be inferred. For each possible solid phase, the saturation index (SI) was calculated as IAP/K_sp_, where IAP is the ion activity product and K_sp_ is the solubility product constant [27]. If logSI is < 0, it follows that SI is < 1 and the compound in question is fully dissolved at equilibrium. If logSI is ≥ 0, it follows that SI is ≥ 1 and filtrate is saturated or supersaturated with the solid.

In the present work, possible solid phases in the DCT included brushite (CaHPO_4_^.^2H_2_O) and Ca_3_(PO_4_)_2_, (am., s.), but the former was dismissed because logSIbrushite was uniformly negative in both groups and all scenarios. In contrast, at pH 6.8, logSICa_3_(PO_4_)_2_ (am., s.) exceeded zero in approximately half of CKD and a majority of CTRL when precipitation of this solid was not assumed. Ostwald’s rule of stages suggests that in a fluid supersaturated with multiple solids, the one with logSI closest to 0 is likely to precipitate first [27,46]. In the present study, JESS analyses indicated that this solid phase was Ca_3_(PO_4_)_2_ (am., s.) in the DCT of both CKD and CTRL. Two additional modeling scenarios, one with and one without precipitation of Ca_3_(PO_4_)_2_ (am., s.), were therefore investigated. When precipitation was assumed in states of saturation or supersaturation, consequences were examined at pH 6.6, 6.8, and 7.0. In each scenario, [Ca^++^]_DCT_, [CaHPO_4_^0^]_DCT_, [Cacit^–^]_DCT_, [Caox^0^]_DCT_, [CaHCO_3_^+^]_DCT_, and [CaSO_4_^0^]_DCT_ were calculated as concentrations of the most likely pertinent species of dissolved calcium.

### Statistical analysis

The ultimate goals of the present study were to identify a chemical phenomenon by which increased [P]_DCT_ could reduce [Ca^++^]_DCT_ in CKD; to ascertain whether [PTH] would vary significantly with [Ca^++^]_DCT_ if that reduction occurred; and to consider the possibility that [PTH] was related to eGFR because it was related to [Ca^++^]_DCT_. Table 1 summarizes the sequence of questions addressed and the examinations conducted to pursue these goals.

**Table 1:** Rationale for inquiries into the tradeoff-in-the-nephron hypothesis.

QUESTIONS	RELEVANT EXAMINATIONS
Were [PTH], total [P]_DCT_, and total [Ca]_DCT_ related to eGFR?	Regressions of [P]_DCT_ and [Ca]_DCT_ on eGFR, and of [PTH] on 100/eGFR (Fig 1)
Was [PTH] related to total [P]_DCT_ and total [Ca]_DCT_?	Regressions of [PTH] on [P]_DCT_ and [Ca]_DCT_ (Fig 1)
Did pH affect precipitation of Ca_3_(PO_4_)_2_ (am., s.) in the DCT?	Box-and-whisker plots of logSICa_3_(PO_4_)_2_ at pH 6.6, 6.8, and 7.0 (Fig 2); plot of logSICa_3_(PO_4_)_2_ against pH at fixed [P]_DCT_ and [Ca]_DCT_ (S1 Fig)
Was [Ca^++^]_DCT_ related to total [P]_DCT_? To total [Ca]_DCT_?	Regressions of [Ca^++^]_DCT_ on [P]_DCT_ and [Ca]_DCT_ at pH 6.6, 6.8, and 7.0 (Figs 3, 4, S2 and S3)
If [Ca^++^]_DCT_ was related to [P]_DCT_, did precipitation of Ca_3_(PO_4_)_2_ (am., s.) mediate that relationship?	Linkage of precipitation of Ca_3_(PO_4_)_2_ (am., s.) (Fig 2) to regressions of [Ca^++^]_DCT_ on [P]_DCT_ (Figs 3, 4, S2 and S3)
Was [Ca^++^]_DCT_ related to [CaHPO_4_^0^)_DCT_?	Plots of [Ca^++^]_DCT_ against [CaHPO_4_^0^]_DCT_ at pH 6.6, 6.8, and 7.0 (S4 and S5 Figs)
Did anions other than phosphate affect [Ca^++^]_DCT_?	Plots of [Ca^++^]_DCT_ against [Cacit^+^]_DCT_, [Caox^0^]_DCT_, [CaHCO_3_^+^]_DCT_ and [CaSO_4_^0^]_DCT_ at pH 6.8 (S5 Fig)
Was [PTH] related to [Ca^++^]_DCT_?	Regressions of [PTH] on [Ca^++^]_DCT_ (Figs 3, 4, S2 and S3)
Did the relationship of [PTH] to [Ca^++^]_DCT_ explain the relationship of [PTH] to eGFR?	Comparison of regressions of log[PTH] on log[Ca^++^]_DCT_ and log[PTH] on log(eGFR) after standardization of logarithmic values (Fig 5)

In each subset of subjects, mean values were determined for parameters not affected by pH or precipitation of Ca_3_(PO_4_)_2_ (am., s.), including eGFR, [PTH], 24h E_P_, 24h E_Ca_, [P]_s_, [Ca^++^]_s_, [Ca_uf_]_s_, [P]_DCT_, and [Ca]_DCT_. For each of these parameters, normality of distribution was examined with plots and with the Shapiro-Wilk test. When distributions were judged to be normal, differences between means were assessed with t-tests for unpaired values (unequal variances assumed). When distributions were skewed, differences between medians were assessed with the Mann Whitney U test. Results were considered to be statistically significant at p < 0.05.

**Fig 1:**
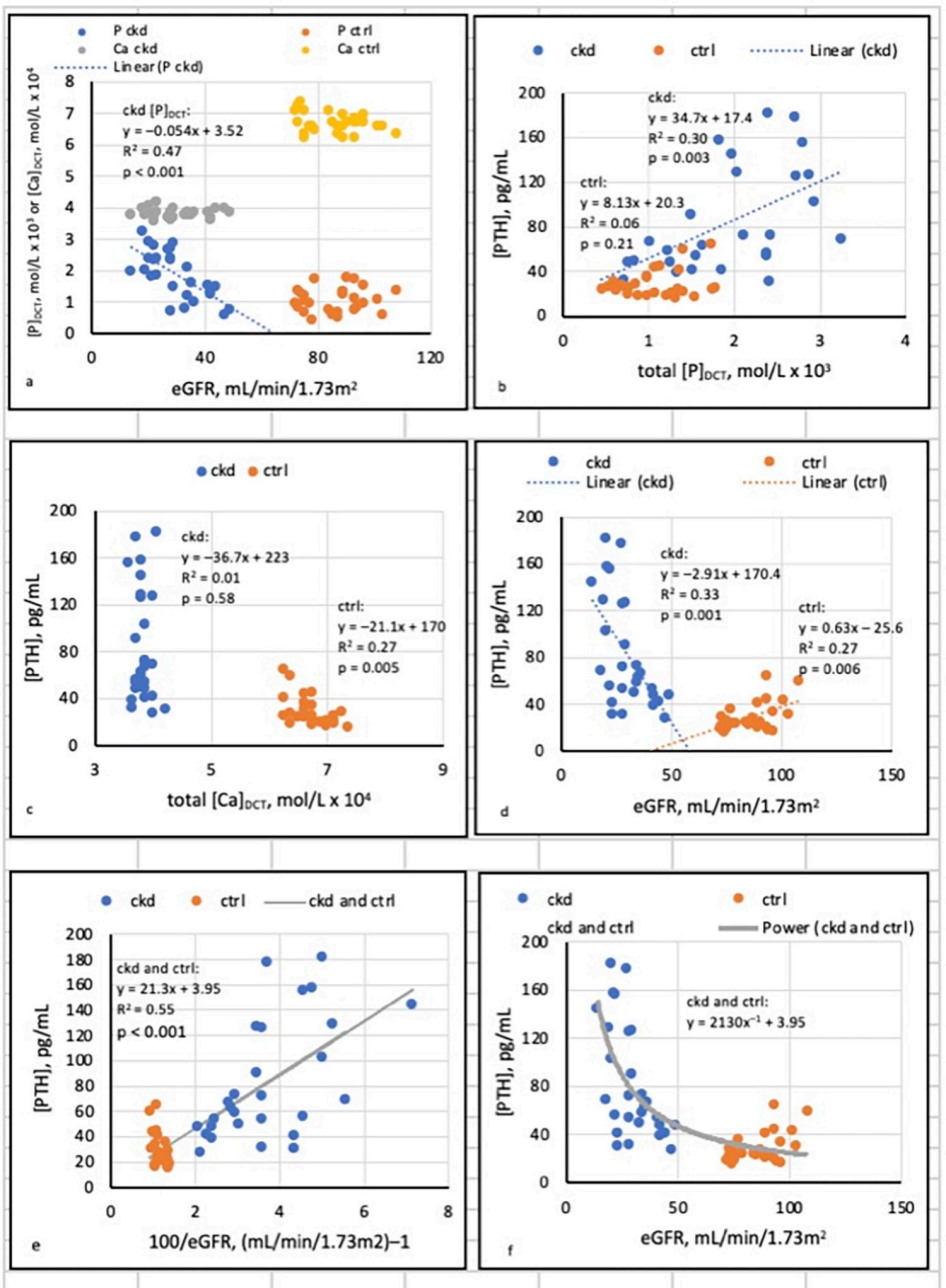
Linear regressions unaffected by pH or precipitation of Ca_3_(PO_4_)_2_ (am., s.).

The hypothesis investigated in the present study was that [Ca^++^]_DCT_ induced by [P]_DCT_ determines [PTH] in stages G_3_ and G_4_ CKD. For comparative purposes, we examined least-squares regressions of total [P]_DCT_ and total [Ca]_DCT_ on eGFR, and regressions of [PTH] on total [P]_DCT_, total [Ca]_DCT_, and eGFR (Fig 1). None of these regressions was affected by pH or the state of precipitation of Ca_3_(PO_4_)_2_ (am., s.).

**Fig 2:**
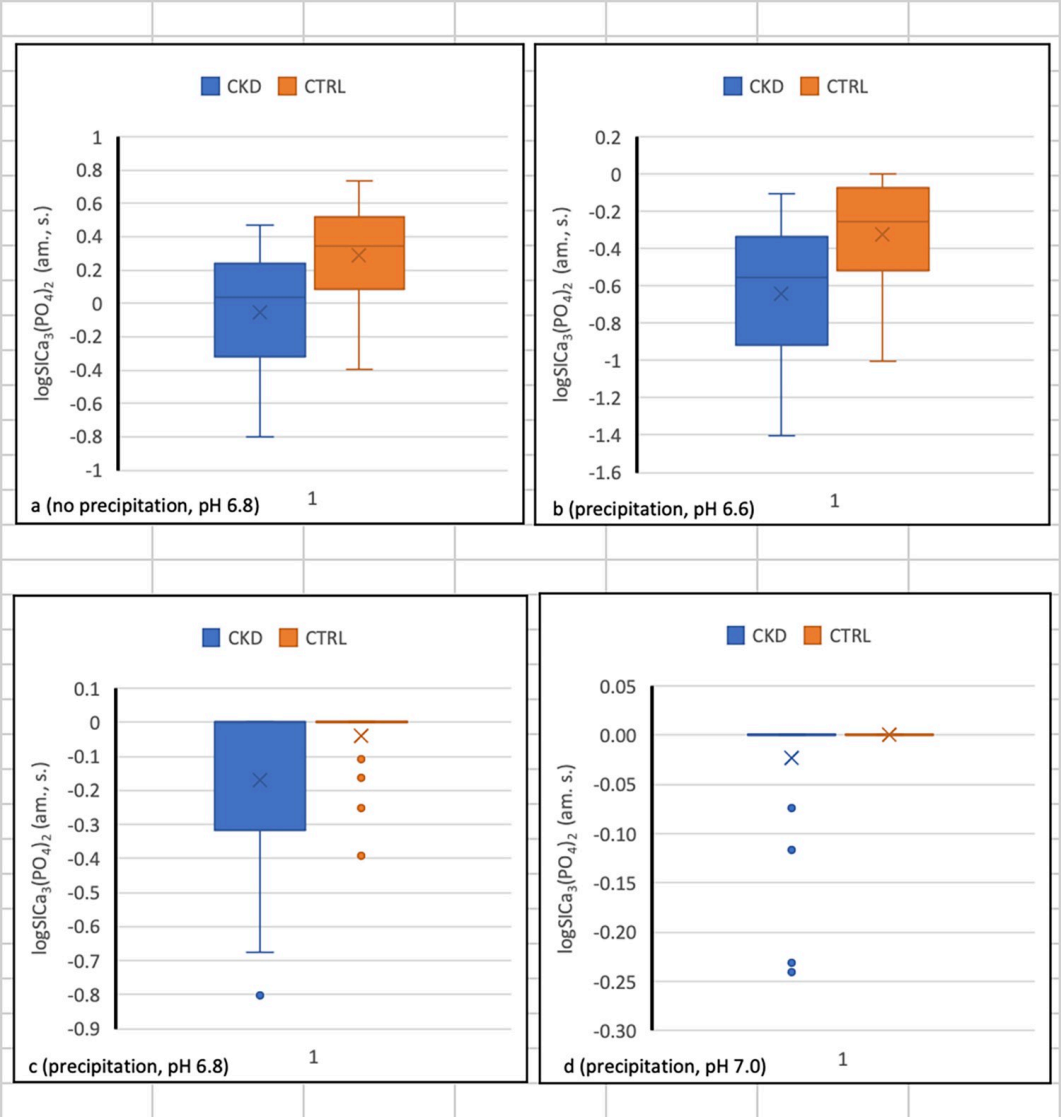
Dependence of logSICa_3_(PO_4_)_2_ (am., s.) on pH in the DCT.

Since JESS identified Ca_3_(PO_4_)_2_ (am., s.) as the Ca species most likely to precipitate in the DCT, we examined the maximum, minimum, median, mean, and 25^th^ to 75^th^ percentiles of logSICa_3_(PO_4_)_2_ (am., s.) at pH 6.8 with precipitation excluded, and at pH 6.6, 6.8, or 7.0 with precipitation assumed if logSICa_3_(PO_4_)_2_ (am., s.) equaled or exceeded zero (Fig 2).

**Fig 3:**
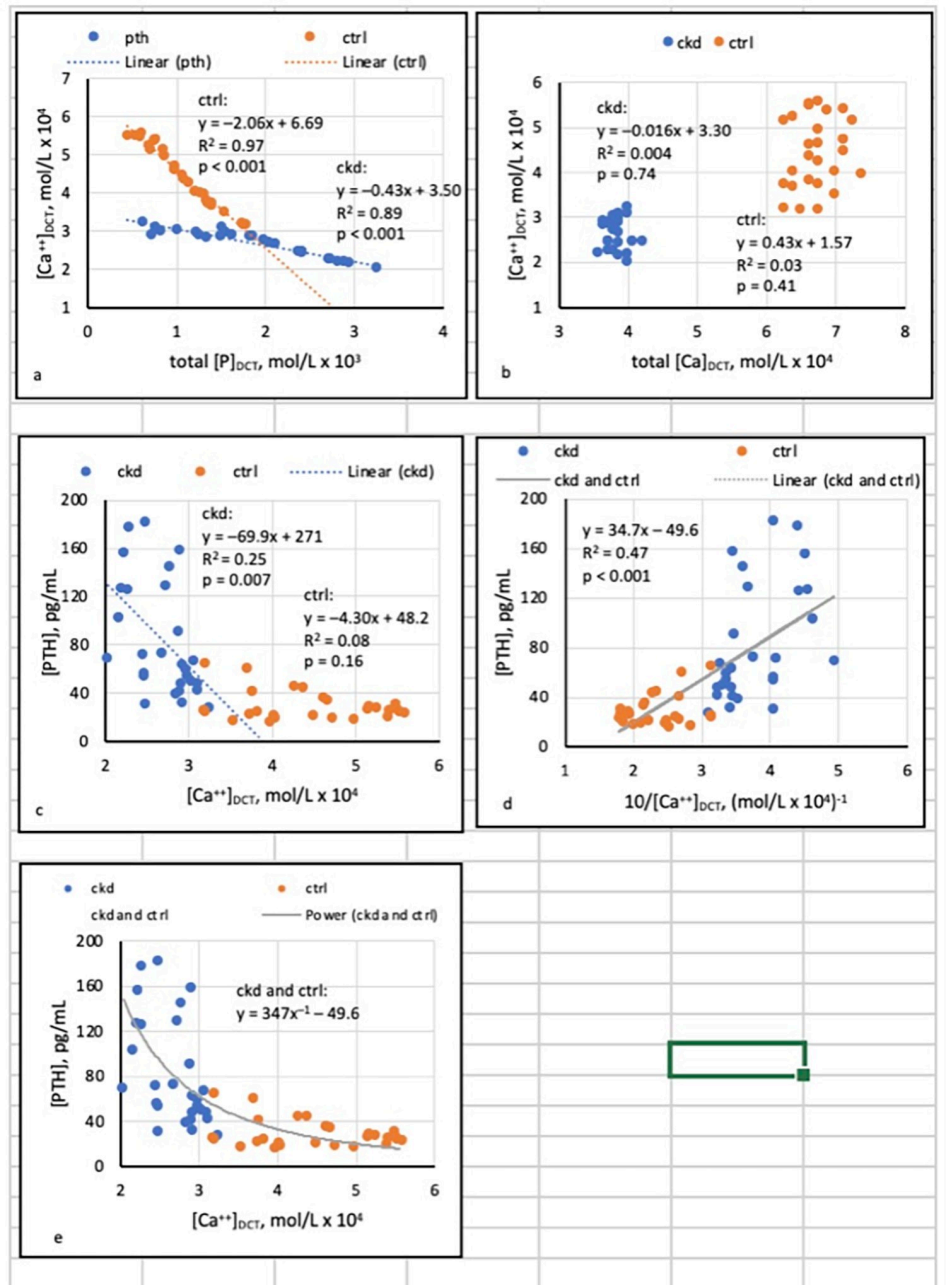
Regressions assuming pH 6.8 and precipitation of Ca_3_(PO_4_)_2_ (am., s.).

**Fig 4:**
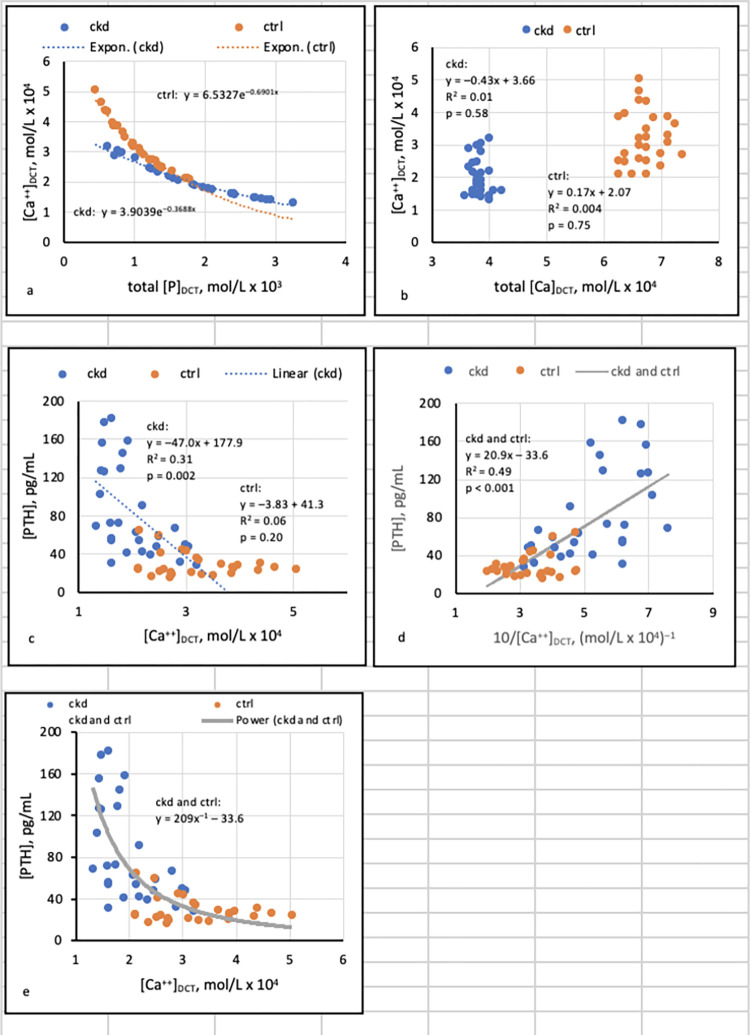
Regressions assuming pH 7.0 and precipitation of Ca_3_(PO_4_)_2_ (am., s.).

**Fig 5:**
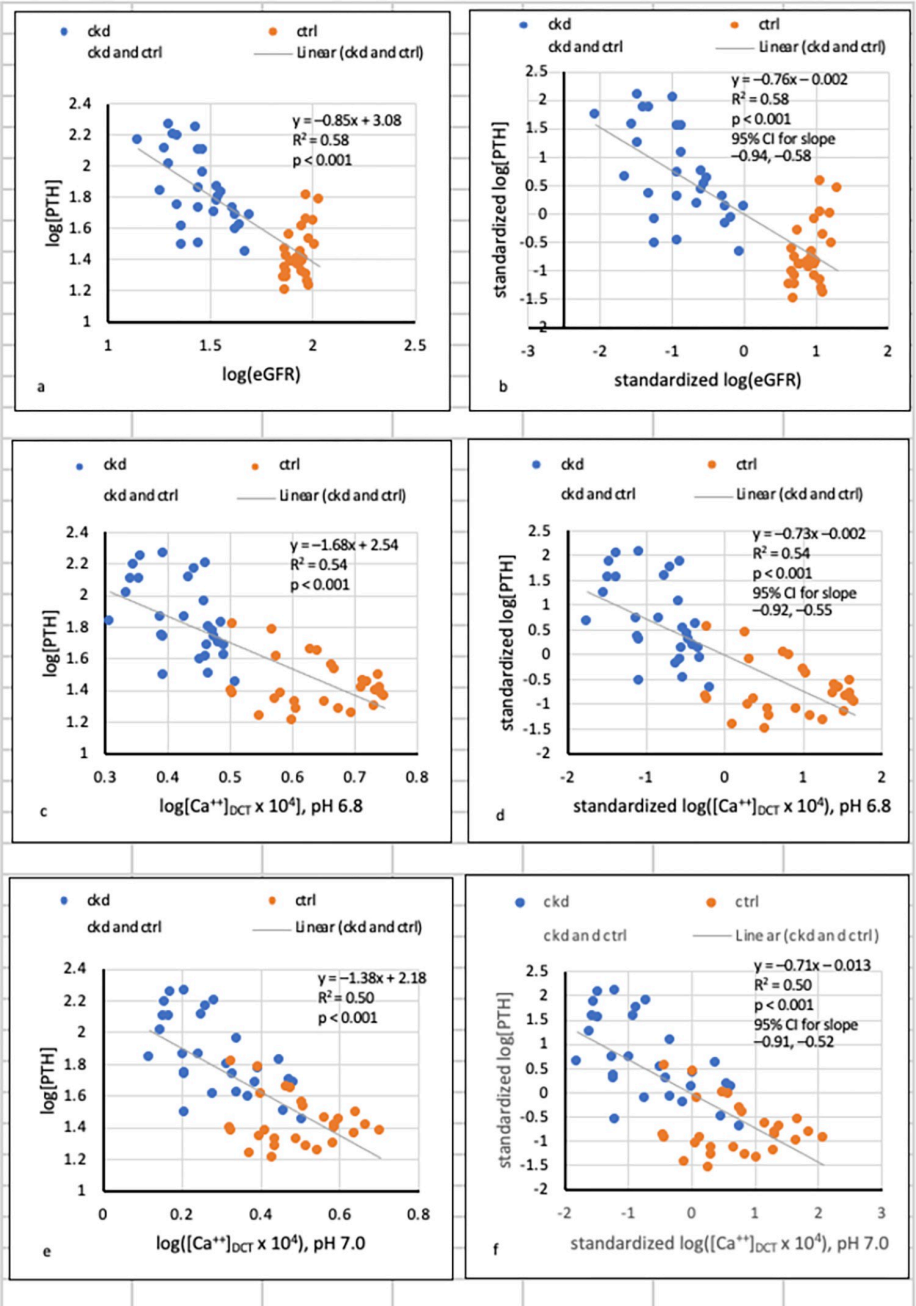
Regressions of [PTH] on eGFR and [Ca^++^]_DCT_ after log-transformation of variables and standardization of logarithmic values.

Factors determining [Ca^++^]_DCT_ (“determinants”) were sought with least-squares regressions of [Ca^++^]_DCT_ on total [P]_DCT_ and total [Ca]_DCT_. The tradeoff-in-the-nephron hypothesis was tested with regressions of [PTH] on [Ca^++^]_DCT_ at pH 6.6, 6.8, and 7.0, assuming precipitation of Ca_3_(PO_4_)_2_ (am., s.) if logSI was ≥ 0 (Figs 3,4, S2 and S3). In combined CKD and CTRL, inverse curvilinear relationships between [PTH] and either eGFR or [Ca^++^]_DCT_ were assessed as power functions in the form *y* = *kx*^*–1*^ + *c*, where *y* = [PTH] and *x* = eGFR (Fig 1) or [Ca^++^]_DCT_ (Figs 3 and 4). Relationships between [PTH] and both eGFR and [Ca^++^]_DCT_ were then investigated by log-log transformation of these variables and subsequent standardization of logarithmic values (Fig 5).

To investigate the possibility that CaHPO_4_^0^ was a mediator of the effect of total [P]_DCT_ on [Ca^++^]_DCT_, we produced scatterplots of [CaHPO_4_^0^]_DCT_ against total [P]_DCT_, and of [Ca^++^]_DCT_ against [CaHPO_4_^0^]_DCT_, at pH 6.6, 6.8, and 7.0 (S4 Fig). To determine whether other Ca species had affected [Ca^++^]_DCT_, we examined regressions of [Ca^++^]_DCT_ on [Cacit^–^]_DCT_, [Caox^0^]_DCT_, [CaHCO_3_^+^]_DCT_, and [CaSO_4_^0^]_DCT_, assuming pH 6.8 and precipitation of Ca_3_(PO_4_)_2_ (am., s.) if logSI was ≥ 0 (S5 Fig).

Although we assumed in the present work that fractional delivery of calcium to the DCT (FD_Ca_) was 0.1 in both CKD and CTRL, we also considered the possibility that in CKD, a higher FD_Ca_ might increase the effect of total [Ca]_DCT_ on [Ca^++^]_DCT_. To investigate that possibility, we performed regressions of [Ca^++^]_DCT_ on total [Ca]_DCT_, [Ca^++^]_DCT_ on [P]_DCT_, and [PTH] on [Ca^++^]_DCT_ at FD_Ca_ 0.15 and 0.2 and pH 6.6, 6.8, and 7.0 (S6 and S7 Figs).

Statistical analyses were carried out with Microsoft Excel and R version 4.0.3 (R Core Team, 2020) [47].

### Clinical research policies

The research project that provided the data reported herein [12,13] was approved and periodically reviewed by the Institutional Review Board (IRB) of the Stratton Veterans’ Affairs Medical Center, Albany, NY, USA. The project was conducted with adherence to the Declaration of Helsinki, and written informed consent was obtained from all participants. Data employed in the present study were obtained from tests on urine, serum and plasma obtained at an IRB-approved research clinic visit. IRB oversight over access to all data was in place.

## Results

We present our results in accordance with the order of questions posed in Table 1. Table 2 compares means of variables that were not affected by filtrate pH or precipitation of Ca_3_(PO_4_)_2_ (am., s.) in the DCT.

**Table 2:** Parameters unaffected by pH or precipitation of Ca_3_(PO_4_)_2_ (am., s.) in the DCT^a^.

Parameter	Subjects with CKD (n = 28)	Control subjects (n = 27)	p
eGFR, mL/min/1.73m^2^	29.9 (9.5)	86.2 (10.2)	< 0.001
[PTH], pg/mL	82.9 (47.6)	29.0 (12.4)	< 0.001
[Ca^++^]_s_, mol/L x 10^3^	1.24 (0.05)	1.26 (0.03)	0.1
[Ca_uf_]_s_, mol/L x 10^3^	1.34 (0.05)	1.34 (0.06)	0.9
[P]_s_, mol/L x 10^3^	1.15 (0.24)	1.11 (0.20)	0.7
E_P_, mol/24h x 10^2^	2.60 (0.83)	2.66 (1.03)	0.8
E_Ca_, mol/24h x 10^3^	1.05 (0.74)	3.31 (1.84)	< 0.001
Total [Ca]_DCT_, mol/L x 10^4^	3.82 (0.14)	6.71 (0.31)	< 0.001
Total [P]_DCT_, mol/L x 10^3^	1.89 (0.75)	1.07 (0.38)	< 0.001

^a^Values are mean (SD).

[Ca^++^]_s_, [Ca_uf_]_s_, [P]_s_, and 24-hour E_P_ were not different in CKD and CTRL. In CKD, eGFR was lower, [PTH] higher, 24h E_Ca_ lower, total [Ca]_DCT_ lower, and total [P]_DCT_ higher than in CTRL.

Fig 1 depicts regressions that were not affected by filtrate pH or precipitation of Ca_3_(PO_4_)_2_ (am., s.).

Fig 1A shows that total [P]_DCT_ varied inversely with eGFR in CKD but not CTRL, and total [Ca]_DCT_ was unrelated to eGFR in each group. [PTH] rose with [P]_DCT_ in CKD but not CTRL (Fig 1B); in contrast, [PTH] was inversely related to [Ca]_DCT_ in CTRL but not CKD (Fig 1C).

Fig 1D shows that [PTH] varied inversely with eGFR in CKD and directly with eGFR in CTRL. However, a scatterplot of [PTH] (*y*) against eGFR (*x*) in the combined groups appeared to depict a hyperbola described in part by the formula *xy* = *k* (Fig 1D). Accordingly, a least-squares regression of [PTH] (*y*) on 100/eGFR (100/*x*) displayed a linear relationship (Fig 1E). The associated equation was then modified to express [PTH] (*y*) as a power function of eGFR (*x*) in the form *y* = *kx*^*–1*^ *+ c*. The hyperbola described by the function is included in Fig 1F.

Fig 2 depicts the median, mean, 25^th^-75^th^ percentile range, and upper and lower limits of logSICa_3_(PO_4_)_2_ (am., s.) in CKD and CTRL under four combinations of conditions. LogSICa_3_(PO_4_)_2_ (am., s.) was 0 at saturation and > 0 at supersaturation. In Fig 2A, pH was 6.8 and precipitation was considered not to occur; in Fig 2B–2D, at pH 6.6, 6.8, and 7.0, precipitation was assumed to occur when DCT filtrate was saturated or supersaturated with Ca_3_(PO_4_)_2_ (am., s.).

JESS predicted that with no precipitation, the DCT would be saturated or supersaturated with this compound at pH 6.8 in half of CKD and a majority of CTRL (Fig 2A). In contrast, at pH 6.6, saturation with Ca_3_(PO_4_)_2_ (am., s.) was predicted in no subjects with CKD and in five CTRL (Fig 2B). At pH 6.8, saturation with and consequent precipitation of Ca_3_(PO_4_)_2_ (am., s.) were predicted in the top two quartiles of CKD and in all but four CTRL (Fig 2C). At pH 7.0, precipitation was predicted in all but four CKD and in all CTRL (Fig 2D). If [Ca]_DCT_ and [P]_DCT_ were fixed hypothetically at 0.5 mM and 1.5 mM respectively, saturation with Ca_3_(PO_4_)_2_ (am., s.) occurred at pH 6.73 (see S1 Fig and related discussion in SI).

Under conditions in which precipitation of Ca_3_(PO_4_)_2_ (am., s.) did not occur (Fig 2A and 2B), linear regressions showed that total [Ca]_DCT_ was a major and [P]_DCT_ was a minor determinant of [Ca^++^]_DCT_ (see S2 and S3 Figs and related discussion in SI). In contrast, when precipitation was assumed to occur in states of supersaturation (Fig 2C and 2D), regressions showed that [P]_DCT_ was the sole determinant of [Ca^++^]_DCT_ (Figs 3A, 3B, 4A and 4B).

At pH 6.8, [PTH] varied inversely with [Ca^++^]_DCT_ in CKD but not CTRL, and began to rise unequivocally in CKD at [Ca^++^]_DCT_ of approximately 2.8 x 10^−4^ mol/L (Fig 3C). In the combined groups, a scatterplot of [PTH] (*y*) against [Ca^++^]_DCT_ (*x*) appeared to depict a hyperbola described in part by the formula *xy* = *k* (Fig 3C). A significant least-squares regression of [PTH] (*y*) on 10/[Ca^++^]_DCT_ (10/*x*) was accordingly documented (Fig 3D), and the associated linear equation was modified to express [PTH] (*y*) as a power function of [Ca^++^]_DCT_ (*x*) in the form *y = kx*^*–1*^ *+ c*. The hyperbola described by the function is included in Fig 3E.

At pH 7.0, results resembled and accentuated those obtained at pH 6.8. [Ca^++^]_DCT_ was again entirely dependent on [P]_DCT_ in both groups, but relationships were curvilinear rather than linear (Fig 4A). [Ca^++^]_DCT_ remained independent of total [Ca]_DCT_ (Fig 4B). [PTH] rose as [Ca^++^]_DCT_ fell in CKD but not CTRL, and the trajectory of [PTH] became more positive at [Ca^++^]_DCT_ of approximately 2.0 mol/L x 10^−4^ (Fig 4C). Again, a scatterplot of [PTH] against [Ca^++^]_DCT_ in both groups appeared to depict a hyperbola described in part by the equation *xy* = *k* (Fig 4C). A significant linear regression of [PTH] (*y*) on 10/[Ca^++^]_DCT_ (*x*) was accordingly demonstrated (Fig 4D), and the associated equation was modified to express [PTH] (*y*) as a power function of [Ca^++^]_DCT_ (*x*) in the form *y = kx*^*–1*^ *+ c*. The hyperbola described by the function is included in Fig 4E.

Because relationships of [PTH] to eGFR and to [Ca^++^]_DCT_ were visually similar (Figs 1F, 3E, and 4E), we performed an additional test of the hypothesis that the first relationship resulted from the second. Whereas (*kx*^*–1*^ *+ c*) is not amenable to log transformation, the general formula *y* = *kx*^*n*^–a simpler power function of *x*–transforms to the linear equation log*y* = log*k* + *n*(log*x*).

Fig 5 presents results of this modification for *y* = [PTH] and *x* = eGFR or [Ca^++^]_DCT_ at pH 6.8 or 7.0.

Fig 5A, 5C, and 5E show that at either pH value, log transformations yielded significant linear regressions of log[PTH] on both log(eGFR) and log([Ca^++^]_DCT_ x 10^4^). These results indicate that in addition to the hyperbolic formulas in Figs 1F, 3E, and 4E, power functions of the form *y* = k*x*^n^ related [PTH] (*y*) to both eGFR and [Ca^++^]_DCT_ (*x*) in combined CKD and CTRL. In Fig 5B, 5D, and 5F, each value of log[PTH], log(eGFR) and log([Ca^++^]_DCT_ x 10^4^) was assigned a z-score. After this standardization procedure, slopes of lines relating log[PTH] to either log(eGFR) or log([Ca^++^]_DCT_ x 10^4^) were virtually identical, as is indicated by the extreme overlap in confidence intervals.

Other issues are examined in Supporting Information. Although precipitation of Ca_3_(PO_4_)_2_ (am., s.) was assumed in most scenarios, we also considered the possibility that formation of CaHPO_4_^0^ might cause a reduction of [Ca^++^]_DCT_ and an elevation of [PTH] as [P]_DCT_ rose (S4 Fig). In CKD and CTRL, [CaHPO_4_^0^]_DCT_ increased with total [P]_DCT_ at pH 6.6, 6.8, and 7.0 (S4A and S4B Fig). [Ca^++^]_DCT_ fell as [CaHPO_4_^0^]_DCT_ rose, but at a given [CaHPO_4_^0^]_DCT_, [Ca^++^]_DCT_ varied markedly with pH and therefore did not appear to be controlled by [CaHPO_4_^0^]_DCT_ *per se* (S4C and S4D Fig). The small calculated formation of CaHPO_4_^0^ as a percentage of total calcium argues strongly against a role for this species in determination of [Ca^++^]_DCT_.

Assuming pH 6.8 and precipitation of Ca_3_(PO_4_)_2_ (am., s.), we examined regressions of [Ca^++^]_DCT_ on concentrations of Cacit^−^and other Ca complexes to ascertain whether these compounds could reduce [Ca^++^]_DCT_ and raise [PTH] secondarily. [Ca^++^]_DCT_ varied inversely with [CaHPO_4_^0^]_DCT_, presumably because of the relationship of [CaHPO_4_]_DCT_ to [P]_DCT_ (S4 Fig), and directly with concentrations of all other complexes (S5 Fig). Citrate, oxalate, bicarbonate, and sulfate did not reduce [Ca^++^]_DCT_ significantly.

In principle, fractional delivery of filtered calcium to the DCT (FD_Ca_) could have been higher in CKD than the assigned value, 0.1, with the result that total [Ca]_DCT_ exerted an effect on [Ca^++^]_DCT_ that was not evident at FD_Ca_ 0.1. We therefore examined regressions of [Ca^++^]_DCT_ on total [Ca]_DCT_, [Ca^++^]_DCT_ on total [P]_DCT_, and [PTH] on [Ca^++^]_DCT_ at FD_Ca_ 0.15 or 0.2. At pH 6.6, 6.8, and 7.0, logSICa_3_(PO_4_)_2_ (am., s.) was essentially zero in all subjects at both FD_Ca_ values (data not shown), and therefore indicated universal precipitation of Ca_3_(PO_4_)_2_ (am., s.) at the increased values of FD_Ca_. At pH 6.6, a previously significant relationship of [Ca^++^]_DCT_ to total [Ca]_DCT_ disappeared (S3B Fig), and a strong relationship of [Ca^++^]_DCT_ to total [P]_DCT_ emerged (S6 and S7 Figs). At pH 6.8 and 7.0, [Ca^++^]_DCT_ remained independent of [Ca]_DCT_ and exclusively dependent on [P]_DCT_ (S6 and S7 Figs). Regressions of [PTH] on [Ca^++^]_DCT_ were significant at both values of FD_Ca_ and all three pH values; R^2^ for these regressions was similar to values obtained at FD_Ca_ 0.1 (Figs 3 and 4).

## Discussion

### Background and summary of principal findings

In stages G_3_ and G_4_ CKD, one of the cardinal features of SHPT is persistence of normal [Ca^++^]_s_ and [Ca_uf_]_s_ until CKD is far advanced [12]. If C_cr_ is assumed to approximate GFR, [Ca_uf_]_s_ equals E_Ca_/C_cr_ + TR_Ca_/C_cr_, *i*.*e*., the summed amounts of Ca excreted and reabsorbed per volume of filtrate [12,48]. Since flux of Ca into plasma determines and equals E_Ca_, the ratio E_Ca_/C_cr_, calculated as [Ca]_u_[cr]_p_/[cr]_u_, quantifies the contribution of net influx from all sources to [Ca_uf_]_s_ [48]. TR_Ca_/C_cr_, the difference between [Ca_uf_]_s_ and E_Ca_/C_cr_, describes the simultaneous contribution of tubular Ca reabsorption. Ordinarily, influx provides 1–2% and reabsorption 98–99% of the flux maintaining normal [Ca_uf_]_s_ [12].

PTH regulates reabsorption of the 10% of filtered Ca that reaches the DCT by controlling expression of the apical calcium channel transient receptor potential vanilloid 5 (TRPV5), the intracellular transporter calbindin-D_28K_, and the basolateral extrusion proteins sodium-calcium exchanger 1 (NCX1) and plasma membrane calcium ATPase 1b (PMCA1b) [10,11,30]. In primary hyperparathyroidism (PHPT), elevated [PTH] causes hypercalcemia by increasing TR_Ca_/C_cr_ [12,49]; in SHPT, comparable or higher [PTH] is associated with and presumably required to achieve normal TR_Ca_/C_cr_ and normocalcemia [12]. This presumption is consistent with the observation that cinacalcet, a calcimimetic that suppresses synthesis and secretion of PTH, reduced tubular Ca reabsorption and caused hypocalcemia as it lowered [PTH] in CKD stages G_3_ and G_4_ [50].

The tradeoff-in-the-nephron hypothesis states that as GFR falls, [Ca^++^]_DCT_ also falls in response to increased total [P]_DCT_; if [P]_DCT_ reduces [Ca^++^]_DCT_ sufficiently, [PTH] rises to preserve Ca reabsorption and maintain normocalcemia [8]. Whereas the hypothesis was previously supported by significant relationships of [PTH] to E_P_/C_cr_, a surrogate for [P]_DCT_ [12,13,24,25], the present study showed additionally that [Ca^++^]_DCT_ is related to total [P]_DCT_ in CKD and CTRL (Figs 3A and 4A). If precipitation of amorphous Ca_3_(PO_4_)_2_ is posited in states of supersaturation, the results imply that in CKD, [PTH] increases as [P]_DCT_ rises and [Ca^++^]_DCT_ falls. At FD_Ca_ 0.1 and pH 6.8 or 7.0, the upward trajectory of [PTH] is accentuated at a sufficiently reduced [Ca^++^]_DCT_ (Figs 3E and 4E). A critical observation is that in combined CKD and CTRL, curvilinear relationships of [PTH] to [Ca^++^]_DCT_ and to eGFR are essentially identical after log-transformation of variables and standardization of logarithmic values (Fig 5).

### Estimation of total [Ca]_DCT_ and [P]_DCT_

Our estimations of total [Ca]_DCT_ and [P]_DCT_ are based on published physiologic observations and measurements of [Ca_uf_]_s_, eGFR, and 24-hour E_P_. Given that micropuncture studies of normal rodents showed fractional Ca delivery to the DCT of 10% [30], we calculated total [Ca]_DCT_ as the rate of Ca delivery divided by the rate of filtrate delivery to the DCT, or (0.1)eGFR[Ca_uf_]_s_/{FD_f_(eGFR)}, where FD_f_ = fractional delivery of filtrate to that segment. This formula simplified to total [Ca]_DCT_ = (0.1)[Ca_uf_]_p_/FD_f_. We assumed FD_f_ of 0.2 in CTRL and 0.35 in CKD [31–33]; since [Ca_uf_]_s_ was not different in CKD and CTRL, FD_f_ was solely responsible for differences in total [Ca]_DCT_ in the two groups. In CKD, higher FD_f_ lowered total [Ca]_DCT_; this consequence could possibly have obscured an effect of higher FD_Ca_ on [Ca^++^]_DCT_, but when FD_Ca_ was increased to 0.15 or 0.2, [Ca^++^]_DCT_ remained independent of total [Ca]_DCT_, and total [P]_DCT_ continued to be the sole determinant of [Ca^++^]_DCT_ (S6 and S7 Figs).

Diurnal variation in [P]_s_ necessitated a different line of reasoning to estimate total [P]_DCT_ [51]. Because E_P_ approximates the rate at which phosphate is delivered to the DCT [29], we inferred, in accordance with classic micropuncture observations [9], that normal phosphate influx from the gut raises [P]_DCT_ as GFR falls. We also recognized that a correlation between [PTH] and [P]_DCT_ would explain why limiting intestinal phosphate influx has consistently prevented, mitigated, or reversed SHPT in animal and human studies [8,14–22].

### Determinants of [Ca^++^]_DCT_

According to JESS calculations, the principal determinant of [Ca^++^]_DCT_ depended on whether precipitation of Ca_3_(PO_4_)_2_ (am., s.) occurred in the DCT. When no precipitation, FD_Ca_ 0.1, and pH 6.8 were assumed (S2 Fig), total [Ca]_DCT_ was found to be the major factor and total [P]_DCT_ a minor factor determining [Ca^++^]_DCT_ in CKD and CTRL. At pH 6.6, mean logSICa_3_(PO_4_)_2_ (am., s.) was substantially negative, the DCT was not saturated with this solid (with five exceptions in CTRL), and relationships of [Ca^++^]_DCT_ to [Ca]_DCT_ and [P]_DCT_ resembled those predicted in the absence of precipitation at pH 6.8 (S2 and S3 Figs). When precipitation, FD_Ca_ 0.1, and pH 6.8 or 7.0 were assumed, supersaturation of the DCT with Ca_3_(PO_4_)_2_ (am., s.) was more prevalent, and [P]_DCT_ emerged as the sole determinant of [Ca^++^]_DCT_ (Figs 3 and 4). When precipitation and FD_Ca_ 0.15 or 0.20 were assumed, supersaturation with Ca_3_(PO_4_)_2_ (am., s.) was universal at pH 6.6, 6.8, and 7.0, and [Ca^++^]_DCT_ was determined exclusively by [P]_DCT_ (S6 and S7 Figs). We therefore conclude that the effect of [P]_DCT_ on [Ca^++^]_DCT_ is dominant when precipitation of Ca_3_(PO_4_)_2_ (am., s.) occurs and negligible when precipitation does not occur. By inference, tradeoff-in-the-nephron is not applicable when FD_Ca_ is ≤ 0.1 and pH is ≤ 6.6 simultaneously, but it is applicable under the more likely conditions that either pH is ≥ 6.7 (S1 Fig), or FD_Ca_ is ≥ 0.1 (S6 and S7 Figs), or both are true. In all scenarios, we presume that the solid phase of Ca_3_(PO_4_)_2_ (am., s.) may dissolve downstream in a more acidic milieu [52].

If precipitation of Ca_3_(PO_4_)_2_ (am., s.) was not assumed, [CaHPO_4_^0^]_DCT_ became the only plausible phosphate-containing determinant of [Ca^++^]_DCT_, but JESS calculations rendered this scenario unlikely; [Ca^++^]_DCT_ was consistently predicted to be an order of magnitude higher than [CaHPO_4_^0^]_DCT_, and [Ca^++^]_DCT_ varied substantially with pH at a given [CaHPO_4_^0^]_DCT_ (S4 Fig). We conclude that CaHPO_4_^0^ did not mediate the effect of [P]_DCT_ on [Ca^++^]_DCT_.

We also investigated the effect of anions other than phosphate on [Ca^++^]_DCT_. Regressions of [Ca^++^]_DCT_ on [Cacit^–^]_DCT_, [Caox^0^]_DCT_, [CaHCO_3_^+^]_DCT_, and [CaSO_4_^0^]_DCT_ were performed to ascertain whether these compounds had reduced [Ca^++^]_DCT_, but [Ca^++^]_DCT_ and concentrations of each complex varied in the same direction (S5 Fig). These results support the inference that precipitation of amorphous Ca_3_(PO_4_)_2_ determined [Ca^++^]_DCT_, and [Ca^++^]_DCT_ determined complex concentrations other than [CaHPO_4_^0^]_DCT_. Low relative concentrations of these complexes preclude the possibility that [Ca^++^]_DCT_ was suppressed by substances such as citrate or oxalate.

### Regressions of [PTH] on total [P]_DCT,_ [Ca^++^]_DCT_, and eGFR

[PTH] rose with total [P]_DCT_ in CKD (Fig 1B). Although [Ca^++^]_DCT_ was inversely related to [P]_DCT_ in CKD and CTRL, reductions in [Ca^++^]_DCT_ were apparently sufficient to raise [PTH] in CKD only. When precipitation of Ca_3_(PO_4_)_2_ (am., s.) was excluded from consideration or appeared not to have occurred, total [Ca]_DCT_ was the primary determinant of [Ca^++^]_DCT_, and substantial elevations of [PTH] were predicted over a narrow range of [Ca^++^]_DCT_ (S2 and S3 Figs). When precipitation of Ca_3_(PO_4_)_2_ (am., s.) occurred at pH 6.8 or 7.0, [P]_DCT_ was the sole determinant of [Ca^++^]_DCT_; [PTH] increased to abnormal levels over a wider and more plausible range of [Ca^++^]_DCT_, and a continuous hyperbolic relationship between [PTH] and [Ca^++^]_DCT_ emerged if CKD and CTRL were considered together (Figs 3 and 4). After log-log transformation of variables and standardization of logarithmic values, relationships of [PTH] to eGFR and to [Ca^++^]_DCT_ were found to be virtually identical (Fig 5).

At pH values typical of the DCT [28], we infer that increased [P]_DCT_ mediates a decline in [Ca^++^]_DCT_ through precipitation of Ca_3_(PO_4_)_2_ (am., s.). If [Ca^++^]_DCT_ is sufficiently reduced, [PTH] rises to maintain normal Ca^++^ reabsorption. The similarity of relationships of [PTH] to [Ca^++^]_DCT_ and [PTH] to eGFR suggests that the first relationship causes the second (Figs 1F, 3E, 4E, and 5).

### Strengths and limitations of the present study

In the present study, we employed laboratory data and evidence-based physiologic assumptions to estimate total [P]_DCT_ and [Ca]_DCT_ [29, 30], and we drew on published information to assign concentrations to other constituents of DCT filtrate [31–45]. JESS calculations using representative values of [P]_DCT_ and [Ca]_DCT_ predicted precipitation of Ca_3_(PO_4_)_2_ (am., s.) at pH ≥ 6.73 (S1 Fig); similarly, calculations based on measured values showed that at pH ≥ 6.8, [P]_DCT_ determined [Ca^++^]_DCT_ by inducing precipitation of Ca_3_(PO_4_)_2_ (am., s.). [PTH] rose in curvilinear fashion as eGFR and [Ca^++^]_DCT_ fell (Figs 1F, 3E, and 4E); moreover, after log-transformation of variables and standardization of logarithmic values, lines relating [PTH] to eGFR and [PTH] to [Ca^++^]_DCT_ had virtually identical slopes. A strength of our study is the coincidence of robust methodology with chemical evidence that in CKD, phosphate raises [PTH] by reducing [Ca^++^]_DCT_. The effect of [P]_DCT_ on [Ca^++^]_DCT_ is consistent at FD_Ca_ 0.1, 0.15, and 0.2, and it explains why cinacalcet reduced Ca reabsorption and caused hypocalcemia in CKD stages G_3_ and G_4_ [50].

Although our observations support the tradeoff-in-the-nephron hypothesis, it is reasonable to question why R^2^ values for some pertinent regressions are not higher. Several explanations present themselves. First, precipitation that reduces [Ca^++^]_DCT_ occurs only when the DCT is supersaturated with Ca_3_(PO_4_)_2_ (am., s.). Since supersaturation was not universal in CKD at FD_Ca_ of 0.1 (Fig 2C and 2D), [P]_DCT_ affected [Ca^++^]_DCT_ differently in individual subjects. A related consideration is that uromodulin, a protein secreted by the thick ascending limb of Henle’s loop, prevents aggregation of calcium phosphate crystals and may therefore have interfered with precipitation of Ca_3_(PO_4_)_2_ in the DCT (am., s.) [53].

A second potential limitation is that loop diuretic therapy could have contributed to SHPT in CKD [54]. However, patients were instructed to abstain from food or medicines for at least eight hours before plasma was obtained to measure [PTH], and 24-hour E_Ca_ was reduced in proportion to eGFR in CKD (Table 1). We suspect that the contribution of loop diuresis to SHPT was minimal.

A third limitation is that we associated [PTH] at a single moment with calculations of [P]_DCT_ and [Ca^++^]_DCT_ from 24-hour data. Since [P]_DCT_ varies through the day with consumption of food and changes in phosphate reabsorption [51], it is unlikely that our calculated concentrations were identical to those present in the fasting state when blood was sampled for PTH assays. Given that the half-life of PTH is a few minutes [55], we presume that [PTH] was more closely related to a contemporaneous than to an average [P]_DCT_.

Additional limitations arise from assumptions that FD_f_ to the DCT was fixed at 0.2 in CTRL and 0.35 in CKD, and filtrate pH was uniformly 6.6, 6.8, or 7.0. Errors were also inherent in estimations of GFR and determinations of 24-hour E_Ca_ and E_P_. Single-nephron GFR varies in animal models of CKD [9], and it is unclear how this variability affected our data. Although equilibrium constants, especially solubility products of solid phases, are somewhat uncertain, we doubt that these uncertainties undermine our results.

### Summary and conclusions

We describe an examination of the tradeoff-in-the-nephron hypothesis with the Joint Expert Speciation System. At pH values typical of the DCT, we present evidence that [P]_DCT_ determines [Ca^++^]_DCT_ by inducing precipitation of Ca_3_(PO_4_)_2_ (am., s.). Although this phenomenon occurs in both CKD and CTRL, [Ca^++^]_DCT_ appears to fall sufficiently to raise [PTH] in CKD only. Whether they are defined by the equation *y = kx*^*–1*^ *+ c* or the simpler power function *y = kx*^*n*^, relationships of [PTH] to [Ca^++^]_DCT_ and [PTH] to eGFR are so similar that the former seems likely to cause the latter. Our observations strongly suggest that the tradeoff-in-the-nephron hypothesis explains SHPT in stages G_3_ and G_4_ CKD. They also suggest that humans with SHPT may be treated successfully by reducing phosphate influx in proportion to the reduction in GFR.

## Supporting information

S1 Fig Plot of logSI(Ca_3_PO4_2_) (am.,s.) *vs*. pH. (TIFF)

S2 Fig Regressions assuming pH 6.8 and no precipitation of Ca_3_(PO_4_)_2_ (am., s.). (TIFF)

S3 Fig Regressions assuming pH 6.6 and precipitation of Ca_3_(PO_4_)_2_ (am., s.). (TIFF)

S4 Fig Relationship of [Ca^++^]_DCT_ to [CaHPO40]_DCT_ at pH 6.6, 6.8, and 7.0. (TIFF)

S5 Fig Effect of Ca-anion complexes on [Ca^++^]_DCT_. (TIFF)

S6 Fig Regressions at FD_Ca_ to DCT of 0.15 and pH of 6.6 (a-c), 6.8 (d-f), and 7.0 (g-i). (TIFF)

S7 Fig Regressions at FD_Ca_ of 0.2 and pH of 6.6 (a-c), 6.8 (d-f), and 7.0 (g-i). (TIFF)

S1 Table Assumed anion excretion rates at normal and reduced GFR. (TIFF)

S2 Table Estiimated total concentrations in the DCT. (TIFF)

S1 File Supplemental information. (PDF)

S2 File Raw data. (PDF)

S3 File (PDF)

S4 File (PDF)

S5 File (PDF)

S6 File (PDF)

S7 File (PDF)

S8 File (PDF)

S9 File (PDF)

S10 File (PDF)

## Abbreviations

[*x*]: concentration of *x*
[*x*]_p_: concentration of *x* in plasma
[*x*]_s_: concentration of *x* in serum
[*x*]_u_: concentration of *x* in urine
Ca: calcium
Ca^++^: ionized calcium
Ca_3_(PO_4_)_2_ (am., s.): calcium phosphate (amorphous, solid)
Cacit^–^: calcium citrate, transitory form
CaHCO3+: calcium bicarbonate, transitory form
CaHPO4.2H_2_O: calcium monohydrogen phosphate dihydrate; brushite in solid form
CaHPO40: calcium monohydrogen phosphate in solution
CaSO_4_: calcium sulfate
Ca_uf_: ultrafilterable calcium
C_cr_: creatinine clearance, units of volume/time
CKD: chronic kidney disease
CTRL: control
DCT: distal convoluted tubule
eGFR: estimated GFR, units of mL/min/1.73m^2^
E_*x*_: excretion rate of *x*, units of mass/time
E_*x*_/C_cr_: amount of *x* excreted per volume of filtrate (assuming C_cr_ ~ GFR)
FD_Ca_: fractional delivery of Ca (herein, to the DCT)
FD_f_: fractional delivery of filtrate (herein, to the DCT)
GFR: glomerular filtration rate, units of volume/time
IAP: ion activity product
JESS: Joint Expert Speciation System
K_sp_: solubility product constant
MDRD: modification of diet in renal disease
P: phosphorus or phosphate
PHPT: primary hyperparathyroidism
SHPT: secondary hyperparathyroidism
SI: solubility index
Stage G_3_: stage of CKD in which eGFR is 30–60 mL/min/1.73m^2^
Stage G_4_: stage of CKD in which eGFR is 15–30 mL/min/1.73m^2^
