# Supplementary figures and images for: Chemical evidence for the tradeoff-in-the-nephron hypothesis to explain secondary hyperparathyroidism
Source: PLoS One. 2022 Aug 1;17(8):e0272380. doi: 10.1371/journal.pone.0272380 (PMC9342777; doi:10.1371/journal.pone.0272380)

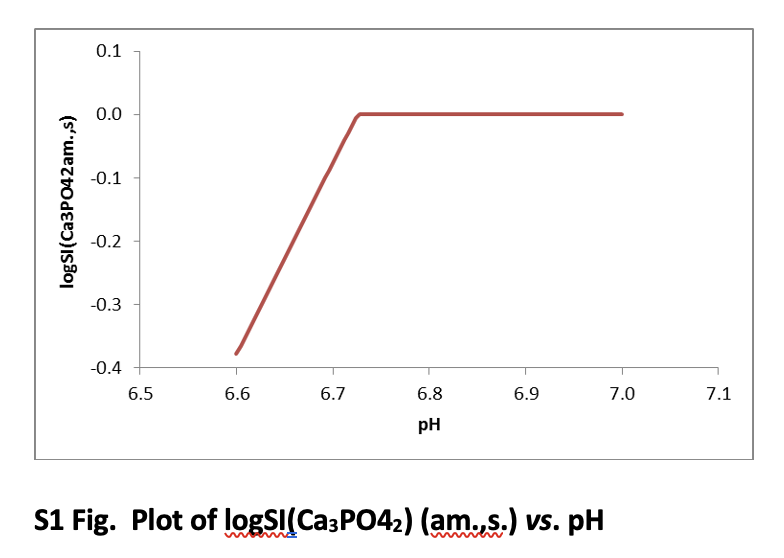

Supplement: S1 Fig — (TIFF) [file pone.0272380.s001.tiff]

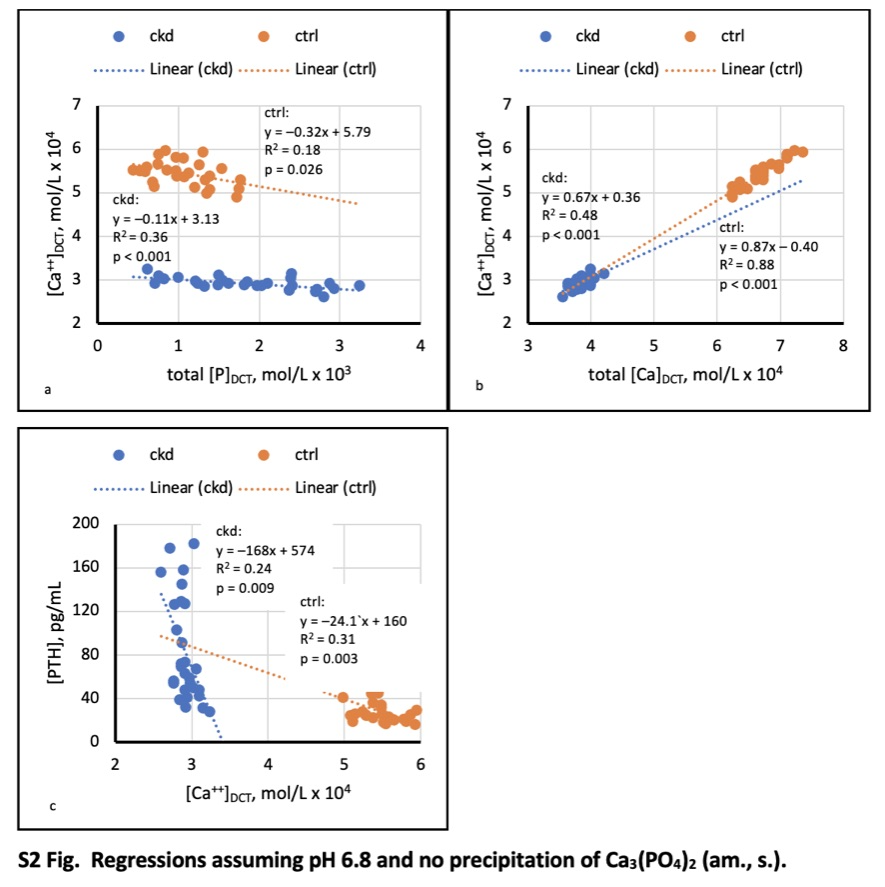

Supplement: S2 Fig — (TIFF) [file pone.0272380.s002.tiff]

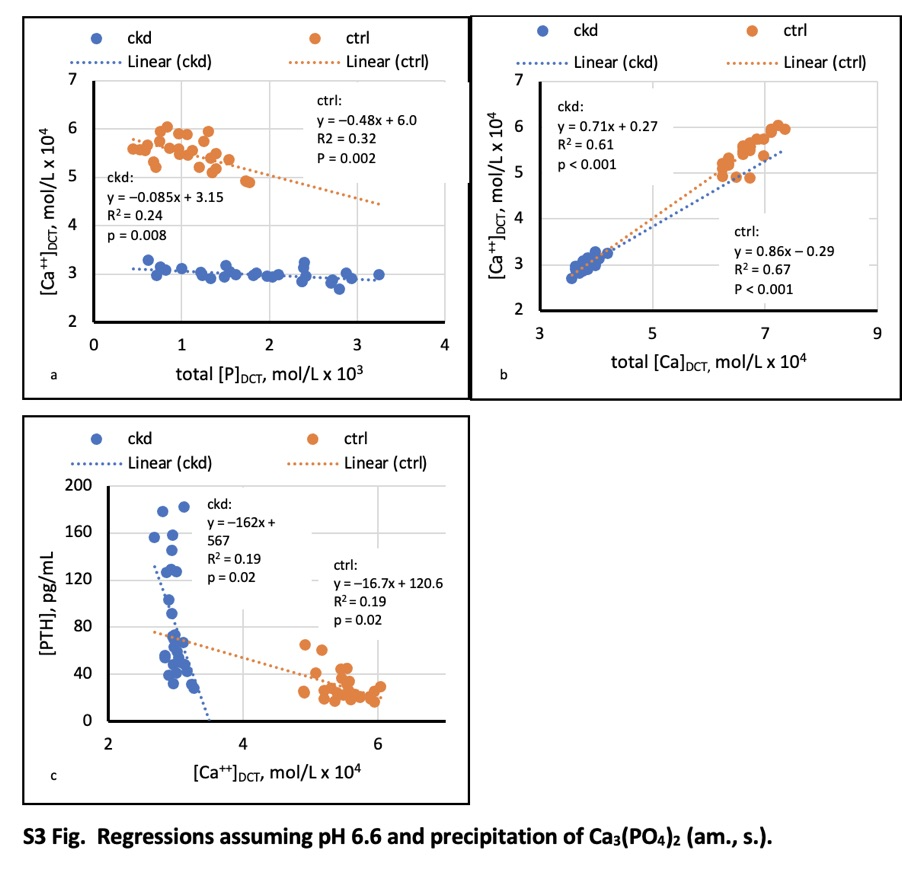

Supplement: S3 Fig — (TIFF) [file pone.0272380.s003.tiff]

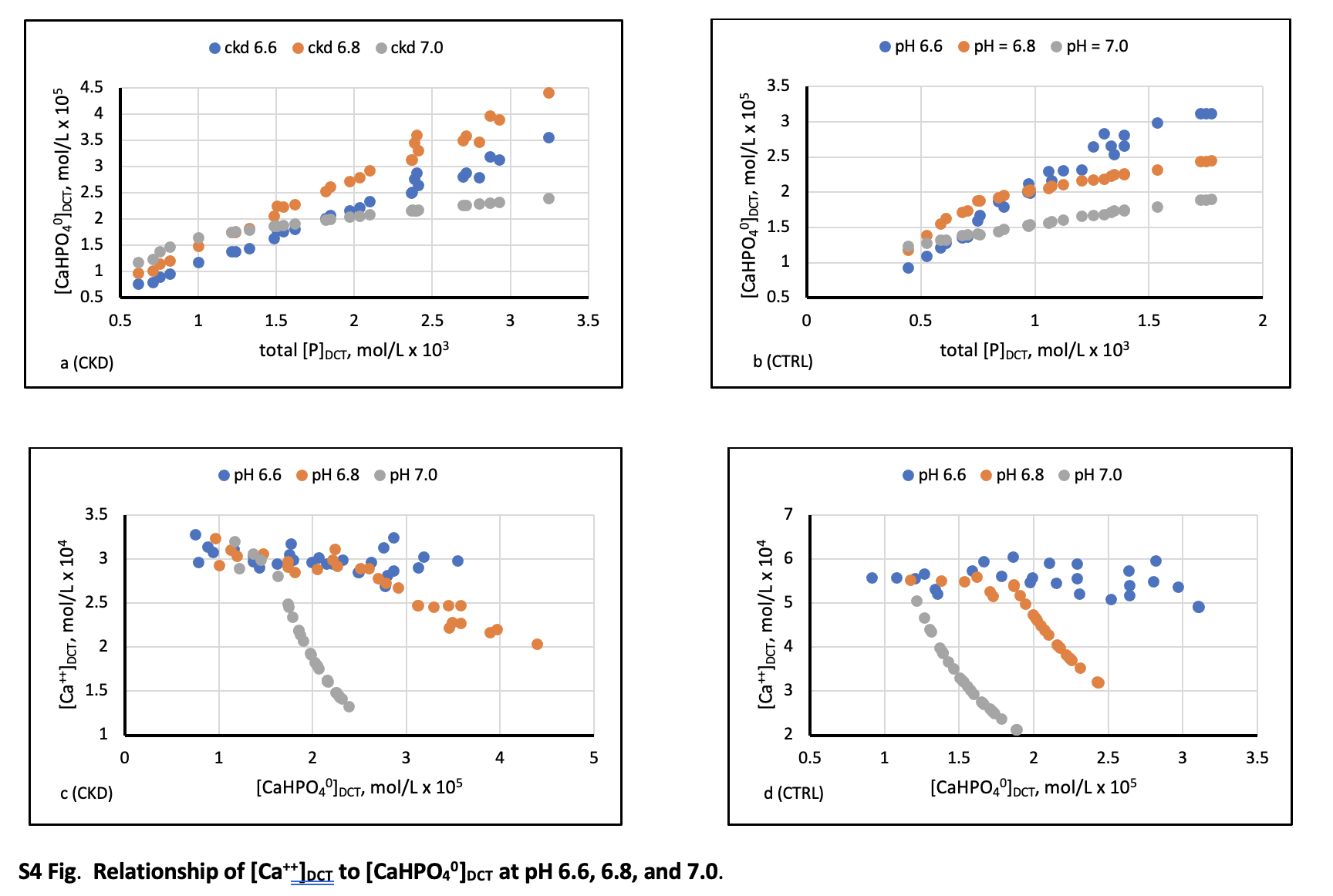

Supplement: S4 Fig — (TIFF) [file pone.0272380.s004.tiff]

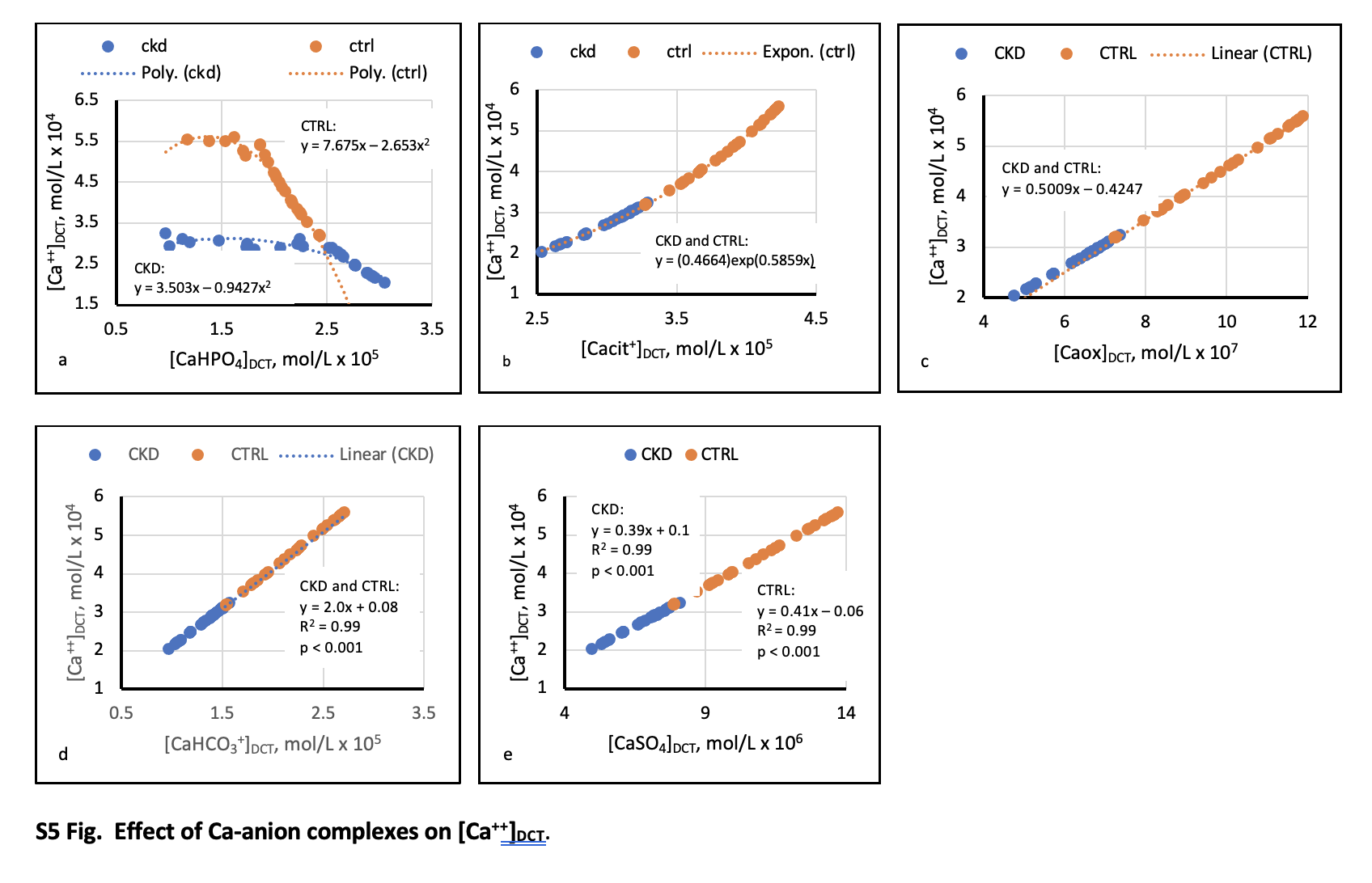

Supplement: S5 Fig — (TIFF) [file pone.0272380.s005.tiff]

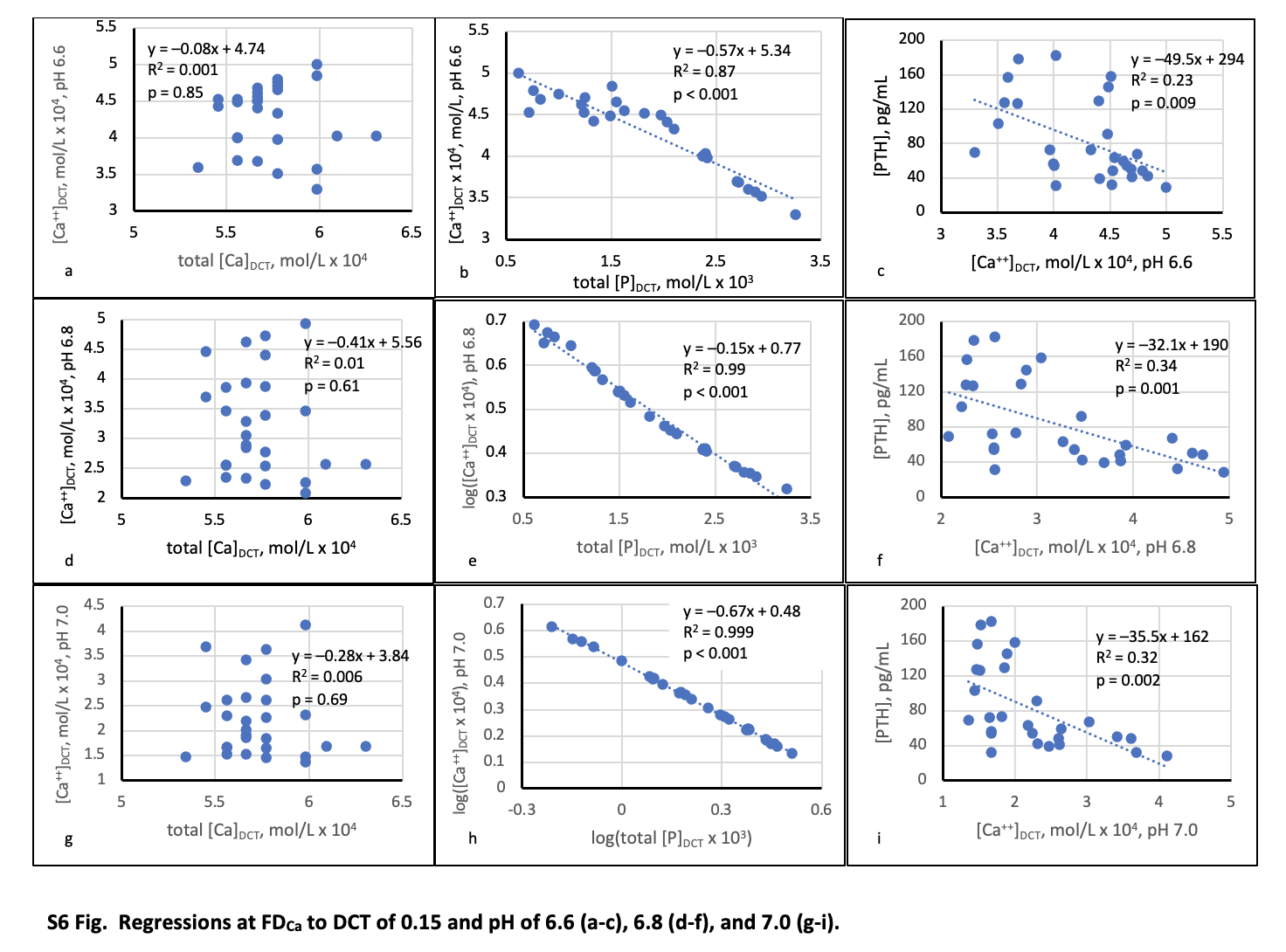

Supplement: S6 Fig — (TIFF) [file pone.0272380.s006.tiff]

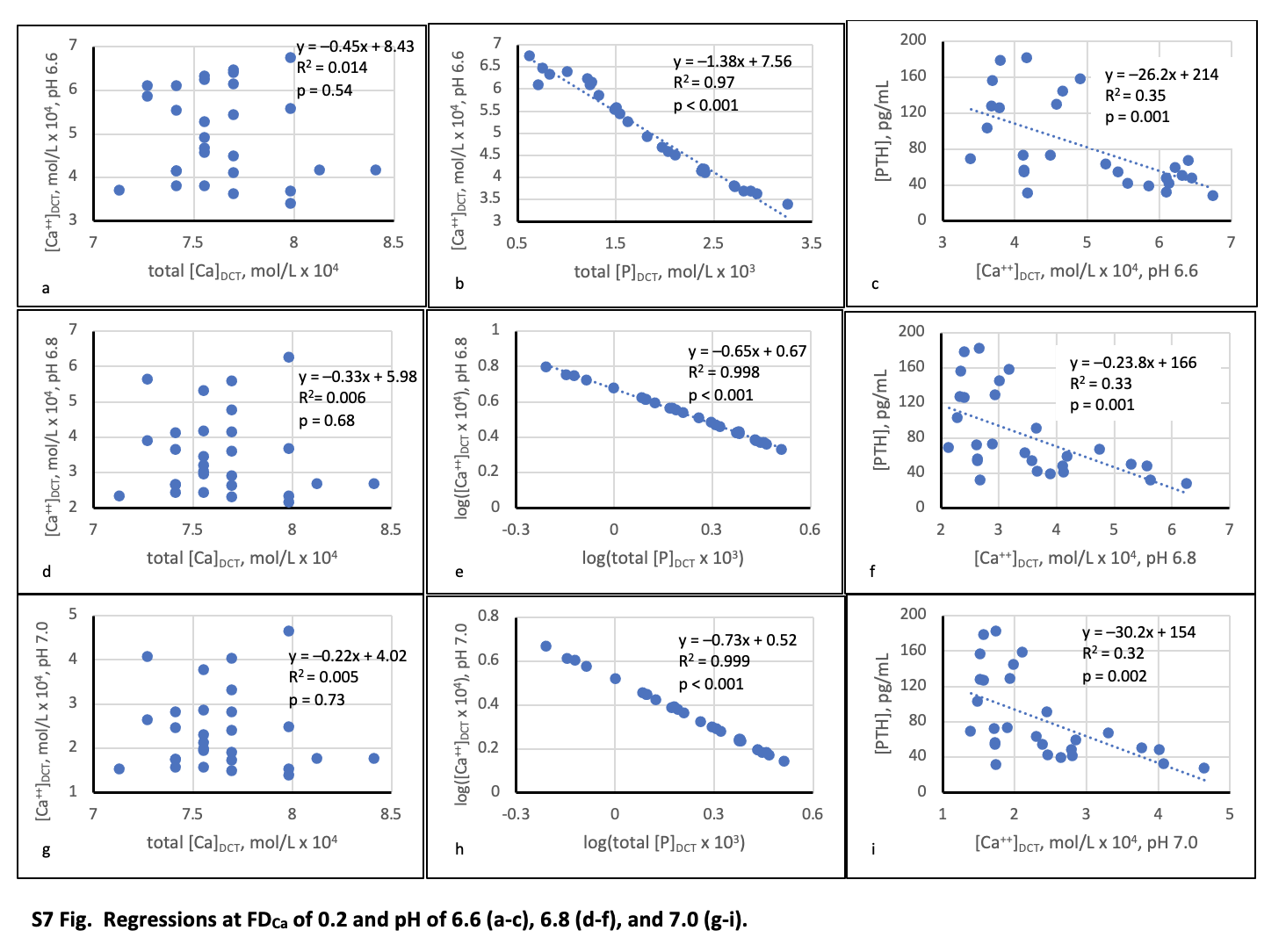

Supplement: S7 Fig — (TIFF) [file pone.0272380.s007.tiff]

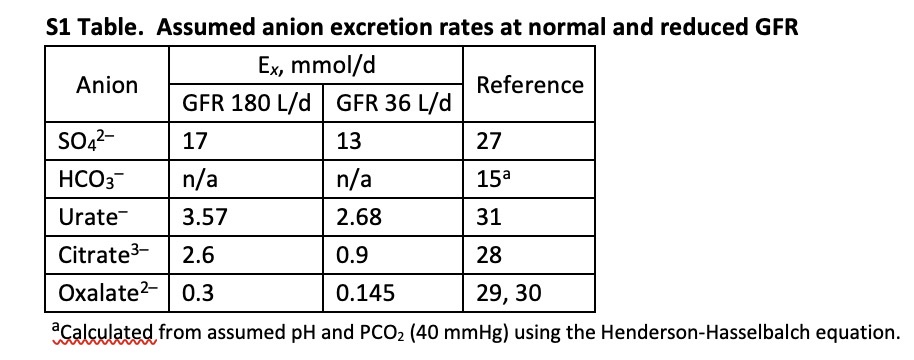

Supplement: S1 Table — (TIFF) [file pone.0272380.s008.tiff]

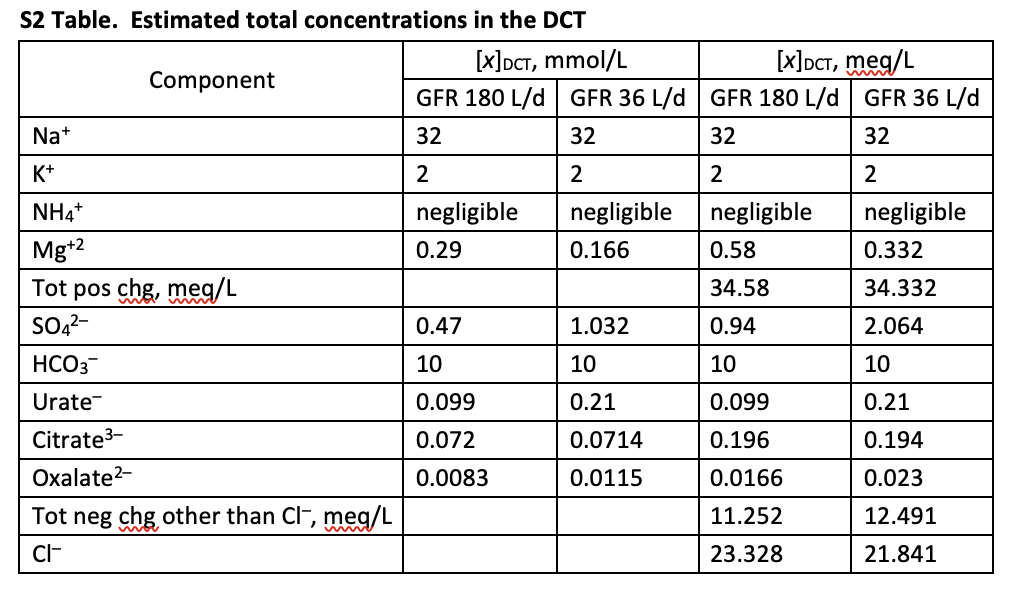

Supplement: S2 Table — (TIFF) [file pone.0272380.s009.tiff]
