# Supplementary material for: Chemical evidence for the tradeoff-in-the-nephron hypothesis to explain secondary hyperparathyroidism
Source: PLoS One. 2022 Aug 1;17(8):e0272380. doi: 10.1371/journal.pone.0272380 (PMC9342777; doi:10.1371/journal.pone.0272380)
Supplement: S1 File — (PDF) [file pone.0272380.s010.pdf]

SUPPORTING INFORMATION

Phelps KR, Gemoets DE, May PM: Chemical evidence for the tradeoff-in-the-nephron hypothesis to explain secondary hyperparathyroidism

I. Estimation of concentrations in the distal convoluted tubule

II. Effect of pH on saturation of DCT filtrate with $Ca_3(PO_4)_2$ (am., s.)

III. Determinants of $[Ca^{++}]_{DCT}$ and relationship of [PTH] to $[Ca^{++}]_{DCT}$, assuming pH 6.8 and no precipitation of $Ca_3(PO_4)_2$ (am., s.)

IV. Determinants of $[Ca^{++}]_{DCT}$ and relationship of [PTH] to $[Ca^{++}]_{DCT}$, assuming pH 6.6 and precipitation of $Ca_3(PO_4)_2$ (am., s.)

V. Possible role of $CaHPO_4^0$ in determination of $[Ca^{++}]_{DCT}$

VI. Possible role of anions in determination of $[Ca^{++}]_{DCT}$

VII. Relationships of $[Ca^{++}]_{DCT}$ to total $[Ca]_{DCT}$ and $[P]_{DCT,}$ and of [PTH] to $[Ca^{++}]_{DCT}$, at fractional delivery of calcium to the DCT (FD_{Ca}) of 0.15 and 0.2

(N.B.: Citations in Supplemental Material refer to those in the main text.)

I. Estimation of concentrations in the distal convoluted tubule (DCT)

To estimate filtrate volume and ionic activities of Ca and phosphate (P) species in the DCT, it was necessary to introduce assumptions concerning fractional delivery of filtrate to that segment (FD_f), and to assign concentrations to constituents of filtrate other than Ca and P. We employed several strategies to meet these requirements.

Estimation of FD_f to the DCT in CTRL and CKD

In micropuncture studies, FD_f to the DCT is deduced from the factor by which the inulin concentration in the DCT rises in comparison to the plasma inulin concentration established by a constant infusion. FD_f is consistently around 0.2 in normal animals. Using data from three published sources [31-33], we assigned an average value of 0.35 to FD_f in CKD. This value probably overstated FD_f in moderate CKD and understated it in advanced CKD, but we had no empiric basis for a more complex derivation of the parameter.

Estimation of [Ca] and [Mg] in the DCT

$[Ca]_{DCT}$ and $[Mg]_{DCT}$ are dependent on the rates of filtration of Ca and Mg, calculated as $eGFR[Ca_{uf}]_s$ and $eGFR[Mg_{uf}]_s$. In the present study, $[Ca_{uf}]_s$ was measured, and $[Mg_{uf}]_s$ was assumed to be 1.4 mg/dL, or 0.58 mmol/L. Since both concentrations are somewhat variable *in vivo*, delivery rates of Ca and Mg to the DCT are expressed as % of filtration rates of these substances (FD_{Ca} and FD_{Mg}), and both fractions are approximately 10% [30, 37]. Lacking evidence to the contrary, we assume that FD_{Ca} and FD_{Mg} are identical at normal and reduced GFR and are independent of fractional delivery of filtrate (FD_f). $[Ca]_{DCT}$ is therefore calculated as $0.1[Ca_{uf}]_s(eGFR)/FD_f(eGFR)$, or $0.1[Ca_{uf}]_s/FD_f$ after simplification. $[Mg_{uf}]_s$ is estimated as $0.7[Mg]_s$; FD_{Mg} is therefore calculated as $(0.1)(0.7)[Mg]_s(eGFR)/FD_f(eGFR)$, or $0.07[Mg]_s/FD_f$ after simplification. The assumptions underlying deductions of $[Ca]_{DCT}$ and $[Mg]_{DCT}$ are based on micropuncture data [30, 37].

Estimation of [Na], [K], pH, osmolality, and [urea] in the DCT

Micropuncture studies performed between 1960 and 1990 provided direct measurements of [Na], [K], and pH in the DCT [28, 31-35]. In the present study, $[osm]_{DCT}$ was equated with minimum urine [osm] attainable after water loading [39], which agrees with direct micropuncture measurements [36]. $[Urea]_{DCT}$ was estimated as $[osm]_{DCT} – 2([Na]_{DCT} + [K]_{DCT} + [Ca]_{DCT} + [Mg]_{DCT})$. $[NH_4^+]_{DCT}$ was assumed to be negligible.

Estimation of anion concentrations in the DCT

Whereas cations reaching the DCT undergo secretion into or absorption from filtrate in that segment and beyond, anions other than $Cl^−$ and $HCO_3^−$ are not transported in the distal nephron. Consequently, $[PO_4]_{DCT}$, $[SO_4]_{DCT}$, $[urate]_{DCT}$, $[citrate]_{DCT}$, and $[oxalate]_{DCT}$ can be estimated as (excretion rate)/(rate of filtrate delivery to DCT), or $E_x/(FD_f)eGFR$. $[Creatinine]_{DCT}$ and $[glucose]_{DCT}$ (if glucose is present in urine) are similarly computed because their tubular handling is limited to the proximal tubule. In the present study, published mean excretion rates for sulfate, urate, citrate, and oxalate were assigned to CKD and CTRL because those rates were not measured [40-45]. $[x^−]_{DCT}$ was estimated as described above, with FD_f of 0.2 in CTRL and 0.35 in CKD. $[Cl^−]_{DCT}$ was calculated as the difference between deduced concentrations of cations and anions other than $Cl^−$. Charge balance was not imposed on speciation calculations, but the resulting inconsistency was negligible (< 2%). Excretion rates in S1 Table were adopted from the literature. DCT concentrations in S2 Table were deduced as described above.

II. Effect of pH on saturation of the DCT with $Ca_3(PO_4)_2$

In S1 Fig, a plot of logSI($Ca_3(PO_4)_2$ (am., s.) is predicted for a hypothetical DCT filtrate with total $[Ca]_{DCT}$ = 0.5 mM and total $[P]_{DCT}$ = 1.5 mM. Other assumed total concentrations are as described in Item 1, and were chosen such that they could occur in either CKD or CTRL. The figure shows that saturation with and precipitation of amorphous calcium phosphate ((Ca_3PO4_2) (am.,s.)) commence at pH ~ 6.73 under these conditions. The calculation demonstrates the consequence of rising $[PO_4^{3−}]_{DCT}$ as pH increases; $[PO_4^{3−}]_{DCT}$ increases nearly

four-fold and more than offsets the falling $[Ca^{++}]_{DCT}$ (which is not even halved). Brushite is found to be distinctly undersaturated over this whole range of conditions with –0.67 > log SI > –0.76. Formation of the amorphous calcium phosphate phase appears to be responsible for the low $[Ca^{++}]_{DCT}$ that emerges from this work.

III. Determinants of $[Ca^{++}]_{DCT}$ and relationship of [PTH] to $[Ca^{++}]_{DCT}$, assuming pH 6.8 and no precipitation of $Ca_3(PO_4)_2$ (am., s.)

S2 Fig shows relationships of $[Ca^{++}]_{DCT}$ to its determinants and the relationship of [PTH] to $[Ca^{++}]_{DCT}$ if pH is 6.8 and precipitation of $Ca_3(PO_4)_2$ (am., s.) does not occur. Graphs a and b show that $[Ca^{++}]_{DCT}$ is more closely associated with $[Ca]_{DCT}$ than with $[P]_{DCT}$ in patients and controls. [PTH] is inversely related to $[Ca^{++}]_{DCT}$ in both groups (graph c), but the slope of the relationship is much steeper in CKD.

IV. Determinants of $[Ca^{++}]_{DCT}$ and relationship of [PTH] to $[Ca^{++}]_{DCT}$, assuming pH 6.6 and precipitation of $Ca_3(PO_4)_2$ (am., s.)

S3 Fig shows relationships of $[Ca^{++}]_{DCT}$ to its determinants and the relationship of [PTH] to $[Ca^{++}]_{DCT}$ if pH is 6.6 and precipitation of $Ca_3(PO_4)_2$ (am., s.) does occur in states of supersaturation. Again, graphs a and b show that $[Ca^{++}]_{DCT}$ is more closely associated with $[Ca]_{DCT}$ than with $[P]_{DCT}$ in patients and controls. [PTH] remains inversely related to $[Ca^{++}]_{DCT}$ in both groups (graph c), but the slope of the relationship is much steeper in CKD.

V. Possible role of $CaHPO_4^0$ in determination of $[Ca^{++}]_{DCT}$

S4 Fig plots $[CaHPO_4^0]_{DCT}$ against total $[P]_{DCT}$ (graphs a and b) and $[Ca^{++}]_{DCT}$ against $[CaHPO_4^0]_{DCT}$ (graphs c and d) in CKD and CTRL. In both groups, $[CaHPO_4^0]_{DCT}$ rises with total

$[P]_{DCT}$, and $[Ca^{++}]_{DCT}$ varies markedly with pH at a given $[CaHPO_4^0]_{DCT}$. $[Ca^{++}]_{DCT}$ is at least tenfold higher than $[CaHPO_4^0]_{DCT}$ at pH 6.6, 6.8, and 7.0. We conclude from these observations that $[CaHPO_4^0]_{DCT}$ does not affect $[Ca^{++}]_{DCT}$ directly at normal or reduced GFR.

VI. Possible role of anions in determination of $[Ca^{++}]_{DCT}$

S5 Fig plots $[Ca^{++}]_{DCT}$ against plausible Ca-anion complexes with pH 6.8 and precipitation of (Ca_3PO4_2) (am.,s.) assumed. $[Ca^{++}]_{DCT}$ is inversely related to $[CaHPO_4^0]_{DCT}$ (graph a), presumably because $[CaHPO_4^0]_{DCT}$ is directly related to $[P]_{DCT}$. In contrast, $[Ca^{++}]_{DCT}$ is directly related to $[Cacit^-]_{DCT}$, $[Caox]_{DCT}$, $[CaHCO_3^+]_{DCT}$, and $[CaSO_4^0]_{DCT}$ (graphs b-e). We conclude that $[Ca^{++}]_{DCT}$ determines concentrations of Ca-anion complexes other than $CaHPO_4^0$. Ca-anion complexes do not determine $[Ca^{++}]_{DCT}$.

VII. Relationships of $[Ca^{++}]_{DCT}$ to total $[Ca]_{DCT}$ and $[P]_{DCT,}$ and of [PTH] to $[Ca^{++}]_{DCT}$, at fractional delivery of calcium to the DCT (FD_{Ca}) of 0.15 and 0.2

We cannot find a reported micropuncture measurement of $[Ca^{++}]_{DCT}$ in experimental CKD. In the present study, we assumed that FD_{Ca} to the DCT was 0.1 in CTRL and CKD [30], and that fractional delivery of filtrate to the DCT (FD_f) was 0.2 in CTRL and 0.35 in CKD [31-33]. Together, these assumptions ensured that total $[Ca]_{DCT}$ would be lower in CKD than in CTRL. In CKD, this difference could have led to an underestimation of the effect of $[Ca]_{DCT}$ and an overestimation of the effect of $[P]_{DCT}$ on $[Ca^{++}]_{DCT}$.

To examine this possibility, we employed JESS to calculate $[Ca^{++}]_{DCT}$ in CKD at pH 6.6, 6.8, and 7.0 under assumptions that FD_{Ca} equaled 0.15 or 0.2 instead of 0.1. The value of 0.2 ensured that total $[Ca]_{DCT}$ would be higher in CKD than in CTRL. FD_f remained at 0.35 in CKD. We examined regressions of $[Ca^{++}]_{DCT}$ on total $[Ca]_{DCT}$ and $[P]_{DCT}$ and regressions of [PTH] on $[Ca^{++}]_{DCT}$. S6 and S7 Figs show that increasing FD_{Ca} to 0.15 or 0.20 did not enhance the effect of total $[Ca]_{DCT}$ on $[Ca^{++}]_{DCT}$. On the contrary, whereas total $[Ca]_{DCT}$ had been the principal determinant of $[Ca^{++}]_{DCT}$ at FD_{Ca} 0.1 and pH 6.6 (S3 Fig), the two concentrations were not

related at either increased FD_{Ca} and the same pH. Instead, $[Ca^{++}]_{DCT}$ was determined entirely by $[P]_{DCT}$ at FD_{Ca} 0.15 or 0.2 and pH 6.6, 6.8, or 7.0. At all three pH values and both increased values of FD_{Ca}, regressions of [PTH] on $[Ca^{++}]_{DCT}$ were similar to those obtained at FD_{Ca} = 0.1 (Figs 3 and 4; S3 Fig). The $[Ca^{++}]_{DCT}$ at which [PTH] rose unequivocally depended more on pH than on FD_{Ca} (graphs a, d, and g, S6 and S7 Figs).

In summary, we infer that at pH 6.6, the likelihood that $Ca_3(PO_4)_2$ (am., s.) will precipitate rises with FD_{Ca} in CKD. More importantly, at any of the three pH values and either increased FD_{Ca}, $[P]_{DCT}$ is the sole determinant of $[Ca^{++}]_{DCT}$, presumably because total $[P]_{DCT}$ is almost always a single-digit multiple of $[Ca]_{DCT}$ at any plausible FD_{Ca}. A comparison of graphs a, d, and g in S6 and S7 Figs suggests that $[Ca^{++}]_{DCT}$ is much more affected by pH than by FD_{Ca}. Consequently, the $[Ca^{++}]_{DCT}$ estimated to induce a clear elevation of [PTH] falls as pH rises (graphs c, f, and i in S6 and S7 Figs; Figs 3c and 4c). At a given pH, regressions of [PTH] on $[Ca^{++}]_{DCT}$ at FD_{Ca} 0.15 or 0.20 are similar to each other, and similar to regressions at pH 6.8 and 7.0 if FD_{Ca} = 0.1 (Figs 3c, 4c).
