# Supplementary material for: Chemical evidence for the tradeoff-in-the-nephron hypothesis to explain secondary hyperparathyroidism
Source: PLoS One. 2022 Aug 1;17(8):e0272380. doi: 10.1371/journal.pone.0272380 (PMC9342777; doi:10.1371/journal.pone.0272380)
Supplement: S2 File — (PDF) [file pone.0272380.s011.pdf]

code	[P]s	EP	Ecr	EP/Ecr	[P]u	[cr]s	[cr]u	EP/Ccr
CKD2	2.7	597	1024.3	0.582837	27.1	2.9	53.4	1.471723
CKD4	2.5	665.4	1458	0.456379	47.7	2	144.5	0.660208
CKD5	2.5	646.8	1468	0.440599	45.3	2.1	156.2	0.609027
CKD6	4.2	992.4	1157.2	0.857587	93.2	1.7	91.2	1.737281
CKD7	3.8	604.3	1228.9	0.491741	55.3	3.4	127.9	1.470055
CKD11	3.2	424.6	731	0.580848	42.2	2	99.5	0.848241
CKD13	3.7	816.4	1224.6	0.666667	29.5	2.8	49.2	1.678862
CKD14	5.3	431.5	1148.5	0.375707	43.3	4.8	101.7	2.043658
CKD15	4.4	963.6	1195.3	0.806157	29.5	2.9	40	2.13875
CKD16	3.8	617.6	865.6	0.713494	69.5	2.7	99	1.895455
CKD18	3.3	564	746	0.756032	40.5	1.8	61.5	1.185366
CKD20	3.7	747	1164	0.641753	34.6	3.1	63.2	1.697152
CKD21	4.4	1189	1372.6	0.866239	79	2.3	108.9	1.668503
CKD23	3.4	888.3	1076	0.825558	31	1.9	39.4	1.494924
CKD24	4.8	917	1311	0.699466	44.1	3	53.8	2.459108
CKD25	3.9	1036	1922.2	0.538966	22.6	1.6	34.6	1.045087
CKD26	4.5	914.3	1293.6	0.706787	47.6	3.5	64.7	2.574961
CKD27	2.7	1056	1339	0.788648	100.1	2.3	100.1	2.3
CKD31	4	863.2	1169.6	0.73803	65.2	2.7	81.9	2.149451
CKD32	3.9	674.5	853.6	0.790183	41	2.2	46.6	1.935622
CKD33	4.1	1037.3	1605.9	0.645931	52	2.3	97.8	1.222904
CKD41	2.7	904.5	1679.1	0.538681	14.5	2.4	53.2	0.654135
CKD45	2.7	1302.1	1710.4	0.761284	58.3	2.2	75.2	1.705585
CKD46	3.2	873.1	1674.2	0.521503	43.6	1.6	178.7	0.390375
CKD49	2.9	814.8	1146.5	0.710685	41	1.6	68	0.964706
CKD50	3.1	579.9	1055	0.549668	19.7	1.4	46.3	0.59568
CKD51	2.8	1117	1866	0.598607	43.4	2	126.8	0.684543
CKD55	2.6	312.4	1167.3	0.267626	10.6	2.8	70.3	0.422191
CKD59	3.5	454.7	1080.7	0.420746	38.5	1.7	104.2	0.628119
CKD62	3.6	1139.8	1536.3	0.741912	19.3	2.8	26.1	2.070498

TRP/Ccr	FEP	FTRP	pth 1-84	FGF23	1,25	eGFR	100/eGFR	25D
1.228277	0.545083	0.454917	158	35.403	25.1	21	4.761905	37.4
1.839792	0.264083	0.735917	41	13.383	38	23	4.347826	42.2
1.890973	0.243611	0.756389	59	13.543	55.9	34	2.941176	47.4
2.462719	0.413638	0.586362	54	26.494	74.7	41	2.439024	44.3
2.329945	0.386857	0.613143	129	30.706	39.6	19	5.263158	41.7
2.351759	0.265075	0.734925	50	17.384	89.6	33	3.030303	55.8
2.02114	0.453746	0.546254	56	23.805	20.4	22	4.545455	21
3.25634	0.385596	0.614404	145	68.316	64.2	14	7.142857	34.2
2.26125	0.48608	0.51392	156	26	27.6	22	4.545455	18.9
1.904545	0.498804	0.501196	169	36.399	58.6	22	4.545455	71.2
2.114634	0.359202	0.640798	67	17.415	57.2	36	2.777778	42.7
2.002848	0.45869	0.54131	182	43.713	33.7	20	5	49.1
2.731497	0.379205	0.620795	126	26.399	19.3	28	3.571429	20.5
1.905076	0.439683	0.560317	63	35.711	49.6	35	2.857143	27.7
2.340892	0.512314	0.487686	103	48.446	25.1	20	5	31
2.854913	0.267971	0.732029	42	12.507	52.6	44	2.272727	lost
1.925039	0.572214	0.427786	69	35.564	21.4	18	5.555556	25.6
1.610889	0.851852	0.148148	72	42.96	44.9	28	3.571429	26.4
1.85055	0.537363	0.462637	31	24.519		23	4.347826	27.5
1.964378	0.496313	0.503687	91	89.109	21.7	29	3.448276	32.2
2.877096	0.298269	0.701731	54	26.285	27.3	28	3.571429	49.5
2.04586	0.242272	0.757728	79	38.187	30	27	3.703704	17.4
0.994415	0.631698	0.368302	127	25.303	47.8	29	3.448276	30.1
2.80963	0.121992	0.878008	39	17.44	31.8	42	2.380952	24
1.935294	0.332657	0.667343	48	48.504	25.5	42	2.380952	24.6
2.50432	0.192155	0.807845	48	14.623	75.1	49	2.040816	36.9
2.11546	0.244479	0.755521	73	19.062	26.5	34	2.941176	17.8
2.17781	0.162381	0.837619	32	20.402	43.4	28	3.571429	35.9
2.871881	0.179463	0.820537	28	16.126	74.9	47	2.12766	51.9
1.529502	0.575138	0.424862	178	20.506	53.1	27	3.703704	21.5

Cai	ECa/Ccr	[Ca]uf	Cai molar	Cai M x 1000	Cauf M	Cauf M x 1000
4.61	0.168	5.3	0.001151	1.15077384	0.001323	1.323015477
5.09	0.039	5.4	0.001271	1.27059411	0.001348	1.347978033
4.81	0.027	5.3	0.001201	1.20069895	0.001323	1.323015477
5.09	0.097	5.4	0.001271	1.27059411	0.001348	1.347978033
4.93	0.053	5.3	0.001231	1.23065402	0.001323	1.323015477
4.93	0.046	5.3	0.001231	1.23065402	0.001323	1.323015477
5.05	0.114	5.2	0.001261	1.26060909	0.001298	1.298052921
4.53	0.094	5.3	0.001131	1.13080379	0.001323	1.323015477
4.73	0.145	5	0.001181	1.18072891	0.001248	1.248127808
5.41	0.055	6	0.00135	1.35047429	0.001498	1.49775337
4.85	0.059	5.4	0.001211	1.21068397	0.001348	1.347978033
5.05	0.123	5.7	0.001261	1.26060909	0.001423	1.422865701
4.77	0.034	5.3	0.001191	1.19071393	0.001323	1.323015477
5.13	0.019	5.3	0.001281	1.28057913	0.001323	1.323015477
4.73	0.028	5.4	0.001181	1.18072891	0.001348	1.347978033
5.25	0.069	5.6	0.001311	1.3105342	0.001398	1.397903145
5.45	0.119	5.6	0.00136	1.36045931	0.001398	1.397903145
5.13	0.018	5.4	0.001281	1.28057913	0.001348	1.347978033
5.13	0.105	5.9	0.001281	1.28057913	0.001473	1.472790814
4.81	0.038	5.2	0.001201	1.20069895	0.001298	1.298052921
5.01	0.019	5.2	0.001251	1.25062406	0.001298	1.298052921
4.89	0.014	4.3	0.001221	1.220669	0.001073	1.073389915
4.81	0.009	5.6	0.001201	1.20069895	0.001398	1.397903145
4.97	0.037	5.1	0.001241	1.24063904	0.001273	1.273090364
4.97	0.08	5.2	0.001241	1.24063904	0.001298	1.298052921
4.89	0.07	5.4	0.001221	1.220669	0.001348	1.347978033
5.09	0.002	5.4	0.001271	1.27059411	0.001348	1.347978033
5.01	0.092	5.1	0.001251	1.25062406	0.001273	1.273090364
5.01	0.011	5.6	0.001251	1.25062406	0.001398	1.397903145
5.17	0.21	5.2	0.001291	1.29056415	0.001298	1.298052921

CODE	[cr]s	eGFR	[P]s	[Ca]i	[Ca]uf	[PTH]1-84	1-84 & 7-84	[PTH]7-84
N2	0.9	89	3.1	5.00875	5.6	21	24	3
N3	0.8	101	3.5	4.88854	5.3	44	77	33
N4	0.7	93	3.5	5.12896	5.4	45	72	27
N6	0.8	103	3	4.96868	5.3	31	52	21
N7	0.7	94	3.5	5.04882	5.4	18	28	10
N8	1	79	2.8	4.92861	5.2	24	29	5
N9	0.8	77	3.2	5.08889	5.3	36	57	21
N10	0.8	73	3.4	5.12896	5.4	22	31	9
N11	0.8	108	2.9	4.96868	5.1	60	120	60
N13	0.7	87	4.9	4.8084	5.1	28	50	22
N14	0.9	93	2.1	5.04882	5.5	20	30	10
N15	0.7	96	3.1	5.08889	5.4	34	59	25
N16	0.9	96	3.4	5.16903	5.6	17	22	5
N17	1.1	73	3.4	4.84847	5.8	29	49	20
N18	0.7	90	4	5.08889	5.2	25	45	20
N19	1	78	3.2	5.24917	5.4	23	38	13
N20	0.8	75	4.1	5.12896	5.1	19	32	13
N21	0.8	75	2.9	5.00875	5.00875	26	51	25
N24	0.8	75	4	5.08889	5.7	21	36	15
N25	1.1	89	3.2	5.00875	5.00875	41	82	41
N27	0.8	74	3.1	5.24917	5.9	16	27	11
N29	0.7	85	4.2	5.00875	5.4	23	46	23
N31	0.9	72	3.4	5.04882	5.7	19	30	11
N32	1.3	89	3.6	5.08889	5.3	24	36	12
N33	0.9	93	4.9	5.00875	5.00875	65	93	28
N35	0.9	78	4	4.96868	5.3	24	51	27
N36	1	84	2.7	4.96868	5.7	25	47	22
N38	0.7	87	3.2	5.2091	5.3	26	49	23

25D	1,25D	FGF23	24h EP	24h Ecr	24h EP/Ecr	24h EP/Ccr	spot EP/Ccr
40.5	66.7	11.018	999.6	1493.8	0.66916589	0.602249297	0.44787
27.7	38.7	4.5325	969.6	1717.8	0.56444289	0.451554314	0.41669
35.3	71.8	23.379	936	1173	0.79795396	0.558567775	0.470909
55.8	66.1	117.63	542.5	1354.8	0.40042811	0.320342486	0.186121
33.5	60	9.3336	727.2	937.3	0.77584551	0.54309186	0.438913
30.8	35	14.798	1235.2	2084.7	0.59250732	0.592507315	0.278234
46.2	55.3	17.591	675.5	1196.4	0.5646105	0.451688399	0.3855
47.8	47	12.978	908.7	1238.4	0.73376938	0.587015504	0.380812
20	90.9	15.294	1342	1105.4	1.21404017	0.971232133	0.45463
25.4	23.4	12.749	532	910	0.58461538	0.409230769	0.583639
24.9	25.8	34.843	625	1416.1	0.44135301	0.397217711	0.21772
22.1	83.3	16.026	832.6	1279.5	0.65072294	0.455506057	0.445848
30.7	68.5	32.967	1319.3	1731.6	0.76189651	0.685706861	0.589039
21.2	53.9	8.3066	548.3	2387.5	0.22965445	0.252619895	0.287347
27.4	28.1	16.158	1427.2	1272.9	1.12121926	0.784853484	0.43
17	38.5	14.5962	n/a	n/a	n/a	n/a	0.8197941
32.5	35.1	13.3182	807.8	1426.5	0.56628111	0.453024886	0.343947
n/a	36.4	12.5182	472.6	867.8	0.54459553	0.435676423	0.292998
19.8	52.8	12.1842	711	1138.5	0.62450593	0.499604743	0.38037
17.5	31.7	7.932	1072.7	2022.9	0.53027831	0.583306145	0.668646
98.1	136.3	10.6568	862.4	1252.8	0.68837803	0.550702427	0.366213
31.1	91	11.7211	463.5	1039.5	0.44588745	0.312121212	0.225379
22.9	94.2	12.6715	626.1	1058.3	0.59160918	0.532448266	0.38008
40.4	77.1	10.5577	882	2416	0.36506623	0.474586093	0.31234
15.2	25	19.7492	1436	2001.7	0.71739022	0.645651196	0.46075
29.6	36.7	15.9907	311	861.7	0.36091447	0.324823024	0.58508
21.9	41.6	5.8652	572	805.2	0.71038251	0.710382514	0.5
30.3	64.6	23.7653	410.3	801.5	0.51191516	0.358340611	0.110526

spot TRP/Ccr	24h ECa	24h ECa/Ecr	24h ECa/Ccr	spot ECa/Ccr	spot TRCa/Ccr	100/eGFR
2.65213	127.5	0.085352792	0.076817512	0.08872	5.51128	1.123596
3.08331	139.2	0.081033881	0.064827104	0.02369	5.27631	0.990099
3.029091	71.2	0.060699062	0.042489344	0.082197	5.317803	1.075269
2.813879	49	0.0361677	0.02893416	0.008181	5.281819	0.970874
3.061087	96.3	0.102741918	0.071919343	0.091483	5.308517	1.06383
2.521766	110.9	0.053197103	0.053197103	0.067762	5.132238	1.265823
2.8145	104.5	0.087345369	0.069876296	0.04264	5.25736	1.298701
3.019188	138.5	0.111837855	0.089470284	0.066421	5.333579	1.369863
2.44537	263.4	0.238284784	0.190627827	0.11654	4.98	0.925926
4.316361	210	0.230769231	0.161538462	0.116361	4.983639	1.149425
1.88228	103.7	0.073229292	0.065906363	0.044376	5.455624	1.075269
2.654152	75.9	0.059320047	0.041524033	0.022022	5.377978	1.041667
2.810961	158.2	0.091360591	0.082224532	0.084353	5.515647	1.041667
3.112653	122.4	0.051267016	0.056393717	0.044898	5.755102	1.369863
3.57	236.8	0.186031896	0.130222327	0.05125	5.14875	1.111111
3.0102059	n/a	n/a	n/a	0.0734109	5.3265891	1.282051
3.756053	100.9	0.070732562	0.05658605	0.014674	5.085326	1.333333
2.607002	60.9	0.07017746	0.056141968	0.010054	4.998696	1.333333
3.61963	344.4	0.302503294	0.242002635	0.07507	5.62493	1.333333
2.531354	169.6	0.083840032	0.092224035	0.042271	4.966479	1.123596
2.733787	227.9	0.181912516	0.145530013	0.13279	5.76703	1.351351
3.974621	37	0.035594036	0.024915825	0.031818	5.368182	1.176471
3.01992	54.1	0.05111972	0.046007748	0.024502	5.675498	1.388889
3.28766	97	0.040149007	0.052193709	0.0726	5.2274	1.123596
4.43925	113.1	0.056501973	0.050851776	0.01593	4.99282	1.075269
3.41492	116.6	0.135313914	0.121782523	0.01924	5.28076	1.282051
2.2	53.9	0.066939891	0.066939891	n/a	5.7	1.190476
3.089474	200	0.249532127	0.174672489	0.090526	5.209474	1.149425

Cai M	Cai M x 1000	Cauf M	Cauf M x 1000
0.00125	1.25031203	0.001398	1.397903145
0.00122	1.22030454	0.001323	1.323015477
0.00128	1.28031952	0.001348	1.347978033
0.00124	1.24030954	0.001323	1.323015477
0.00126	1.26031453	0.001348	1.347978033
0.00123	1.23030704	0.001298	1.298052921
0.00127	1.27031702	0.001323	1.323015477
0.00128	1.28031952	0.001348	1.347978033
0.00124	1.24030954	0.001273	1.273090364
0.0012	1.20029955	0.001273	1.273090364
0.00126	1.26031453	0.001373	1.372940589
0.00127	1.27031702	0.001348	1.347978033
0.00129	1.29032202	0.001398	1.397903145
0.00121	1.21030205	0.001448	1.447828258
0.00127	1.27031702	0.001298	1.298052921
0.00131	1.31032701	0.001348	1.347978033
0.00128	1.28031952	0.001273	1.273090364
0.00125	1.25031203	0.00125	1.250312032
0.00127	1.27031702	0.001423	1.422865701
0.00125	1.25031203	0.00125	1.250312032
0.00131	1.31032701	0.001473	1.472790814
0.00125	1.25031203	0.001348	1.347978033
0.00126	1.26031453	0.001423	1.422865701
0.00127	1.27031702	0.001323	1.323015477
0.00125	1.25031203	0.00125	1.250312032
0.00124	1.24030954	0.001323	1.323015477
0.00124	1.24030954	0.001423	1.422865701
0.0013	1.30032451	0.001323	1.323015477

code	PDCT, mol/L	[Ca]DCT, mol/L	IStr	Tot(P)	Tot(Ca)
CKD2	0.00181955	0.00037783	0.0351000	0.00182	0.0003778
CKD4	0.00185167	0.00038496	0.0351600	0.001852	0.000385
CKD5	0.00121758	0.00037783	0.0345700	0.001218	0.0003778
CKD6	0.00154921	0.00038496	0.0348000	0.001549	0.000385
CKD7	0.00203567	0.00037783	0.0353200	0.002036	0.0003778
CKD11	0.00082352	0.00037783	0.0342100	0.0008235	0.0003778
CKD13	0.00237513	0.00037071	0.0356000	0.002375	0.0003707
CKD14	0.0019727	0.00037783	0.0353800	0.001973	0.0003778
CKD15	0.00280338	0.00035645	0.0360800	0.002803	0.0003564
CKD16	0.00179677	0.00042774	0.0352500	0.001797	0.0004277
CKD18	0.00100273	0.00038496	0.0343900	0.001003	0.000385
CKD20	0.00239055	0.00040635	0.0356700	0.002391	0.0004064
CKD21	0.00271789	0.00037783	0.0359600	0.002718	0.0003778
CKD23	0.00162442	0.00037783	0.0350100	0.001624	0.0003778
CKD24	0.00293459	0.00038496	0.0362100	0.002935	0.000385
CKD25	0.00150701	0.00039922	0.0349400	0.001507	0.0003992
CKD26	0.00325105	0.00039922	0.0365300	0.003251	0.0003992
CKD27	0.00241387	0.00038496	0.0358100	0.002414	0.000385
CKD31	0.0024021	0.00042061	0.0358000	0.002402	0.0004206
CKD32	0.00148865	0.00037071	0.0348600	0.001489	0.0003707
CKD33	0.00237112	0.00037071	0.0356400	0.002371	0.0003707
CKD41	0.00214414	0.00030655	0.0354500	0.002144	0.0003065
CKD45	0.00287378	0.00039922	0.0362000	0.002874	0.0003992
CKD46	0.00133052	0.00036358	0.0347300	0.001331	0.0003636
CKD49	0.00124168	0.00037071	0.0345900	0.001242	0.0003707
CKD50	0.00075747	0.00038496	0.0342100	0.0007575	0.000385
CKD51	0.00210272	0.00038496	0.0353600	0.002103	0.000385
CKD55	0.0007141	0.00036358	0.0340900	0.0007141	0.0003636
CKD59	0.00061921	0.00039922	0.0339900	0.0006192	0.0003992
CKD62	0.00270192	0.00037071	0.0360000	0.002702	0.0003707

Ca+2	CaCitric	CaHPO4	lgSI(Ca3PO42am.,s)	lgSI(Brushite)
0.0002789	0.00003453	0.00002475	-0.04335	-0.6245
0.000284	0.00003484	0.00002563	-0.005035	-0.6093
0.0002865	0.00003504	0.00001711	-0.3517	-0.7848
0.0002879	0.00003511	0.00002181	-0.139	-0.6794
0.0002762	0.00003434	0.00002736	0.03944	-0.5809
0.0002917	0.00003537	0.00001183	-0.6647	-0.9453
0.0002667	0.00003372	0.00003074	0.1253	-0.5303
0.000277	0.00003437	0.00002658	0.01568	-0.5934
0.0002512	0.00003264	0.00003403	0.1875	-0.486
0.000318	0.00003677	0.00002776	0.1135	-0.5745
0.0002951	0.00003556	0.00001454	-0.4803	-0.8556
0.0002934	0.00003535	0.00003396	0.2533	-0.487
0.000268	0.00003376	0.0000352	0.2448	-0.4714
0.0002814	0.00003468	0.00002231	-0.1293	-0.6695
0.0002707	0.0000339	0.00003829	0.3221	-0.4348
0.0002997	0.00003577	0.00002204	-0.1124	-0.6747
0.0002773	0.00003427	0.00004327	0.4385	-0.3817
0.0002771	0.00003433	0.00003237	0.1867	-0.5078
0.0003041	0.00003593	0.00003529	0.3021	-0.4702
0.0002775	0.00003445	0.00002021	-0.2214	-0.7126
0.0002668	0.00003371	0.00003068	0.1238	-0.531
0.0002209	0.00003049	0.00002309	-0.2051	-0.6546
0.0002819	0.00003459	0.00003903	0.3563	-0.4265
0.0002739	0.00003423	0.00001785	-0.3345	-0.7663
0.0002806	0.00003467	0.00001709	-0.362	-0.7854
0.0002984	0.00003576	0.00001113	-0.7076	-0.9717
0.0002808	0.00003463	0.00002871	0.08868	-0.5599
0.0002815	0.00003476	9.918E-06	-0.8328	-1.022
0.0003119	0.00003655	9.522E-06	-0.8236	-1.039
0.0002629	0.00003342	0.00003433	0.2149	-0.4822

tot Pdct M x 1000	tot Cadct M x 10000
1.819545022	3.77834651
1.851666333	3.849636067
1.217583808	3.77834651
1.549211345	3.849636067
2.035667125	3.77834651
0.823519372	3.77834651
2.375133827	3.707056953
1.972697681	3.77834651
2.803379416	3.56447784
1.796769539	4.277373408
1.002730841	3.849636067
2.390552995	4.063504737
2.717888231	3.77834651
1.624423963	3.77834651
2.934587814	3.849636067
1.507005539	3.99221518
3.251052512	3.99221518
2.413868773	3.849636067
2.402101561	4.206083851
1.488646998	3.707056953
2.37112318	3.707056953
2.144137225	3.065450942
2.873783922	3.99221518
1.330523249	3.635767396
1.241679467	3.707056953
0.757468886	3.849636067
2.10272281	3.849636067
0.714102845	3.635767396
0.619205587	3.99221518
2.701921071	3.707056953

All201208

code	IStr	Tot(P)	Tot(Ca)	Ca+2	CaCitric	CaOxalic	CaHPO4	CaHCO3+	CaSO4	lgSI(Ca3PO42am.,s)	tot P x 1000	tot Ca x 10000	Ca+2 x 10000	Cacit x 100000	Caox x 10^7	CaHPO4 x 10^5	CaHCO3+ x 10^5	CaSO4 x 10^6
CKD2	0.0362	0.00182	0.000378	0.000289	3.11E-05	6.64E-07	2.52E-05	1.4E-05	7.16E-06	-0.0126	1.82	3.778	2.891	3.11	6.64	2.52	1.40	7.16
CKD4	0.0363	0.001852	0.000385	0.000289	3.11E-05	6.64E-07	2.56E-05	1.4E-05	7.16E-06	2.07E-07	1.852	3.85	2.891	3.11	6.64	2.56	1.40	7.16
CKD5	0.0357	0.001218	0.000378	0.000297	3.15E-05	6.82E-07	1.74E-05	1.44E-05	7.4E-06	-0.3205	1.218	3.778	2.973	3.15	6.82	1.74	1.44	7.40
CKD6	0.036	0.001549	0.000385	0.000298	3.17E-05	6.85E-07	2.22E-05	1.45E-05	7.41E-06	-0.1085	1.549	3.85	2.984	3.17	6.85	2.22	1.45	7.41
CKD7	0.0365	0.002036	0.000378	0.000272	3.01E-05	6.28E-07	2.64E-05	1.32E-05	6.73E-06	4.14E-07	2.036	3.778	2.722	3.01	6.28	2.64	1.32	6.73
CKD11	0.0354	0.000824	0.000378	0.000303	3.18E-05	6.94E-07	1.21E-05	1.47E-05	7.56E-06	-0.633	0.8235	3.778	3.027	3.18	6.94	1.21	1.47	7.56
CKD13	0.0368	0.002375	0.000371	0.000247	2.85E-05	5.73E-07	2.77E-05	1.19E-05	6.09E-06	0	2.375	3.707	2.467	2.85	5.73	2.77	1.19	6.09
CKD14	0.0364	0.001973	0.000378	0.000278	3.04E-05	6.4E-07	2.61E-05	1.34E-05	6.87E-06	-4.14E-07	1.973	3.778	2.776	3.04	6.40	2.61	1.34	6.87
CKD15	0.0372	0.002803	0.000356	0.000222	2.68E-05	5.18E-07	2.92E-05	1.07E-05	5.45E-06	2.07E-07	2.803	3.564	2.216	2.68	5.18	2.92	1.07	5.45
CKD16	0.0363	0.001797	0.000428	0.000298	3.16E-05	6.82E-07	2.52E-05	1.44E-05	7.36E-06	4.14E-07	1.797	4.277	2.975	3.16	6.82	2.52	1.44	7.36
CKD18	0.0355	0.001003	0.000385	0.000306	3.2E-05	7.01E-07	1.48E-05	1.49E-05	7.63E-06	-0.4493	1.003	3.85	3.061	3.20	7.01	1.48	1.49	7.63
CKD20	0.0368	0.002391	0.000406	0.000247	2.85E-05	5.74E-07	2.77E-05	1.19E-05	6.11E-06	4.14E-07	2.391	4.064	2.473	2.85	5.74	2.77	1.19	6.11
CKD21	0.0371	0.002718	0.000378	0.000227	2.71E-05	5.29E-07	2.89E-05	1.09E-05	5.59E-06	0	2.718	3.778	2.268	2.71	5.29	2.89	1.09	5.59
CKD23	0.0362	0.001624	0.000378	0.000292	3.13E-05	6.7E-07	2.27E-05	1.41E-05	7.23E-06	-0.09839	1.624	3.778	2.918	3.13	6.70	2.27	1.41	7.23
CKD24	0.0373	0.002935	0.000385	0.000216	2.64E-05	5.05E-07	2.96E-05	1.04E-05	5.31E-06	1.24E-06	2.935	3.85	2.161	2.64	5.05	2.96	1.04	5.31
CKD25	0.0361	0.001507	0.000399	0.000311	3.23E-05	7.09E-07	2.24E-05	1.5E-05	7.7E-06	-0.08215	1.507	3.992	3.106	3.23	7.09	2.24	1.50	7.70
CKD26	0.0376	0.003251	0.000399	0.000203	2.53E-05	4.75E-07	3.06E-05	9.72E-06	4.97E-06	8.28E-07	3.251	3.992	2.028	2.53	4.75	3.06	0.97	4.97
CKD27	0.0368	0.002414	0.000385	0.000245	2.84E-05	5.69E-07	2.78E-05	1.18E-05	6.04E-06	2.07E-07	2.414	3.85	2.449	2.84	5.69	2.78	1.18	6.04
CKD31	0.0368	0.002402	0.000421	0.000247	2.85E-05	5.74E-07	2.77E-05	1.19E-05	6.1E-06	-6.21E-07	2.402	4.206	2.472	2.85	5.74	2.77	1.19	6.10
CKD32	0.036	0.001489	0.000371	0.000288	3.1E-05	6.62E-07	2.06E-05	1.39E-05	7.15E-06	-0.1902	1.489	3.707	2.878	3.10	6.62	2.06	1.39	7.15
CKD33	0.0368	0.002371	0.000371	0.000247	2.85E-05	5.74E-07	2.77E-05	1.19E-05	6.1E-06	0	2.371	3.707	2.47	2.85	5.74	2.77	1.19	6.10
CKD41	0.0366	0.002144	0.000307	0.000229	2.73E-05	5.36E-07	2.36E-05	1.11E-05	5.68E-06	-0.1717	2.144	3.065	2.294	2.73	5.36	2.36	1.11	5.68
CKD45	0.0372	0.002874	0.000399	0.00022	2.66E-05	5.13E-07	2.94E-05	1.06E-05	5.4E-06	0	2.874	3.992	2.196	2.66	5.13	2.94	1.06	5.40
CKD46	0.0359	0.001331	0.000364	0.000284	3.08E-05	6.54E-07	1.82E-05	1.38E-05	7.07E-06	-0.303	1.331	3.636	2.842	3.08	6.54	1.82	1.38	7.07
CKD49	0.0357	0.001242	0.000371	0.000291	3.12E-05	6.69E-07	1.74E-05	1.41E-05	7.25E-06	-0.3306	1.242	3.707	2.911	3.12	6.69	1.74	1.41	7.25
CKD50	0.0354	0.000758	0.000385	0.00031	3.22E-05	7.09E-07	1.13E-05	1.51E-05	7.73E-06	-0.6764	0.7575	3.85	3.097	3.22	7.09	1.13	1.51	7.73
CKD51	0.0365	0.002103	0.000385	0.000267	2.98E-05	6.17E-07	2.66E-05	1.29E-05	6.61E-06	2.07E-07	2.103	3.85	2.671	2.98	6.17	2.66	1.29	6.61
CKD55	0.0353	0.000714	0.000364	0.000292	3.12E-05	6.73E-07	1.01E-05	1.42E-05	7.32E-06	-0.8007	0.7141	3.636	2.923	3.12	6.73	1.01	1.42	7.32
CKD59	0.0352	0.000619	0.000399	0.000324	3.29E-05	7.38E-07	9.7E-06	1.58E-05	8.1E-06	-0.7926	0.6192	3.992	3.236	3.29	7.38	0.97	1.58	8.10
CKD62	0.0371	0.002702	0.000371	0.000227	2.72E-05	5.3E-07	2.89E-05	1.09E-05	5.6E-06	-6.21E-07	2.702	3.707	2.274	2.72	5.30	2.89	1.09	5.60
N2	0.0358	0.001258	0.000699	0.000402	3.68E-05	8.94E-07	2.17E-05	1.95E-05	9.94E-06	2.07E-07	1.258	6.986	4.02	3.68	8.94	2.17	1.95	9.94
N3	0.0357	0.001075	0.000661	0.000437	3.82E-05	9.63E-07	2.08E-05	2.12E-05	1.08E-05	-6.21E-07	1.075	6.612	4.371	3.82	9.63	2.08	2.12	10.80
N4	0.0358	0.001127	0.000674	0.000427	3.78E-05	9.43E-07	2.1E-05	2.07E-05	1.05E-05	6.21E-07	1.127	6.737	4.267	3.78	9.43	2.10	2.07	10.50
N6	0.0356	0.00059	0.000661	0.000549	4.21E-05	1.17E-06	1.54E-05	2.66E-05	1.35E-05	-0.1622	0.5899	6.612	5.485	4.21	11.70	1.54	2.66	13.50
N7	0.0357	0.000867	0.000674	0.000497	4.04E-05	1.08E-06	1.95E-05	2.41E-05	1.23E-05	-1.24E-06	0.8665	6.737	4.971	4.04	10.80	1.95	2.41	12.30
N8	0.0361	0.001751	0.000649	0.000319	3.28E-05	7.27E-07	2.43E-05	1.54E-05	7.9E-06	-4.14E-07	1.751	6.487	3.191	3.28	7.27	2.43	1.54	7.90
N9	0.0357	0.000983	0.000661	0.000461	3.91E-05	1.01E-06	2.03E-05	2.24E-05	1.14E-05	0	0.9826	6.612	4.608	3.91	10.10	2.03	2.24	11.40
N10	0.0359	0.001394	0.000674	0.000373	3.55E-05	8.37E-07	2.25E-05	1.81E-05	9.24E-06	4.14E-07	1.394	6.737	3.733	3.55	8.37	2.25	1.81	9.24
N11	0.0359	0.001392	0.000636	0.000369	3.53E-05	8.3E-07	2.26E-05	1.79E-05	9.14E-06	2.07E-07	1.392	6.363	3.694	3.53	8.30	2.26	1.79	9.14
N13	0.0357	0.000685	0.000636	0.000525	4.13E-05	1.13E-06	1.71E-05	2.54E-05	1.29E-05	-0.09023	0.6849	6.363	5.246	4.13	11.30	1.71	2.54	12.90
N14	0.0357	0.000753	0.000686	0.000539	4.18E-05	1.15E-06	1.87E-05	2.61E-05	1.32E-05	0	0.7527	6.862	5.385	4.18	11.50	1.87	2.61	13.20
N15	0.0357	0.000971	0.000674	0.000466	3.93E-05	1.02E-06	2.01E-05	2.26E-05	1.15E-05	-8.28E-07	0.9714	6.737	4.661	3.93	10.20	2.01	2.26	11.50
N16	0.036	0.001539	0.000699	0.000353	3.45E-05	7.95E-07	2.32E-05	1.71E-05	8.72E-06	1.04E-06	1.539	6.986	3.525	3.45	7.95	2.32	1.71	8.72
N17	0.0357	0.000841	0.000724	0.000516	4.1E-05	1.11E-06	1.91E-05	2.5E-05	1.27E-05	-2.07E-07	0.8413	7.236	5.162	4.10	11.10	1.91	2.50	12.70
N18	0.0362	0.001776	0.000674	0.000318	3.27E-05	7.25E-07	2.44E-05	1.54E-05	7.87E-06	1.24E-06	1.776	6.737	3.181	3.27	7.25	2.44	1.54	7.87
N20	0.0358	0.001206	0.000636	0.000404	3.69E-05	8.98E-07	2.16E-05	1.96E-05	9.99E-06	2.07E-07	1.206	6.363	4.04	3.69	8.98	2.16	1.96	9.99
N21	0.0356	0.000706	0.000625	0.000514	4.1E-05	1.11E-06	1.73E-05	2.5E-05	1.27E-05	-0.08931	0.7058	6.249	5.143	4.10	11.10	1.73	2.50	12.70
N24	0.0357	0.001062	0.000711	0.000449	3.87E-05	9.85E-07	2.05E-05	2.18E-05	1.11E-05	0	1.062	7.111	4.487	3.87	9.85	2.05	2.18	11.10
N25	0.0359	0.00135	0.000625	0.000375	3.56E-05	8.41E-07	2.24E-05	1.82E-05	9.29E-06	-6.21E-07	1.35	6.249	3.754	3.56	8.41	2.24	1.82	9.29
N27	0.0358	0.001305	0.000736	0.000398	3.66E-05	8.85E-07	2.18E-05	1.93E-05	9.83E-06	0	1.305	7.361	3.975	3.66	8.85	2.18	1.93	9.83
N29	0.0356	0.000611	0.000674	0.000559	4.24E-05	1.19E-06	1.62E-05	2.71E-05	1.37E-05	-0.1089	0.6108	6.737	5.587	4.24	11.90	1.62	2.71	13.70
N31	0.0357	0.000974	0.000711	0.000472	3.95E-05	1.03E-06	2E-05	2.29E-05	1.17E-05	-2.07E-07	0.974	7.111	4.724	3.95	10.30	2.00	2.29	11.70
N32	0.0359	0.001335	0.000661	0.000382	3.59E-05	8.55E-07	2.22E-05	1.85E-05	9.46E-06	4.14E-07	1.335	6.612	3.823	3.59	8.55	2.22	1.85	9.46
N33	0.0361	0.001729	0.000625	0.00032	3.28E-05	7.28E-07	2.43E-05	1.55E-05	7.92E-06	1.24E-06	1.729	6.249	3.198	3.28	7.28	2.43	1.55	7.92
N35	0.0354	0.000447	0.000661	0.000552	4.22E-05	1.18E-06	1.18E-05	2.68E-05	1.36E-05	-0.3938	0.4466	6.612	5.522	4.22	11.80	1.18	2.68	13.60
N36	0.0357	0.000763	0.000711	0.000541	4.18E-05	1.16E-06	1.87E-05	2.62E-05	1.33E-05	4.14E-07	0.7627	7.111	5.41	4.18	11.60	1.87	2.62	13.30
N38	0.0355	0.000528	0.000661	0.00055	4.21E-05	1.17E-06	1.38E-05	2.67E-05	1.35E-05	-0.2537	0.5282	6.612	5.501	4.21	11.70	1.38	2.67	13.50

CODE	[P]DCT, mol/L	[Ca]DCT, mol/L	IStr	Tot(P)	Tot(Ca)
N2	0.001258	0.00069864	0.03618	0.001258	0.0006986
N3	0.00107527	0.00066121	0.03604	0.001075	0.0006612
N4	0.0011273	0.00067369	0.03605	0.001127	0.0006737
N6	0.00058994	0.00066121	0.03566	0.0005899	0.0006612
N7	0.00086651	0.00067369	0.03577	0.0008665	0.0006737
N8	0.00175128	0.00064873	0.03656	0.001751	0.0006487
N9	0.00098261	0.00066121	0.03594	0.0009826	0.0006612
N10	0.00139426	0.00067369	0.03622	0.001394	0.0006737
N11	0.00139179	0.00063626	0.03624	0.001392	0.0006363
N13	0.00068492	0.00063626	0.03562	0.0006849	0.0006363
N14	0.00075274	0.00068616	0.0357	0.0007527	0.0006862
N15	0.00097143	0.00067369	0.03585	0.0009714	0.0006737
N16	0.00153928	0.00069864	0.03645	0.001539	0.0006986
N17	0.00084128	0.00072359	0.03591	0.0008413	0.0007236
N18	0.00177618	0.00067369	0.03663	0.001776	0.0006737
N20	0.00120639	0.00063626	0.03611	0.001206	0.0006363
N21	0.00070579	0.00062488	0.03572	0.0007058	0.0006249
N24	0.00106183	0.00071111	0.03597	0.001062	0.0007111
N25	0.00135	0.00062488	0.03614	0.00135	0.0006249
N27	0.00130534	0.00073606	0.03628	0.001305	0.0007361
N29	0.00061077	0.00067369	0.03562	0.0006108	0.0006737
N31	0.000974	0.00071111	0.03588	0.000974	0.0007111
N32	0.001335	0.00066121	0.03614	0.001335	0.0006612
N33	0.00172949	0.00062488	0.03645	0.00173	0.0006249
N35	0.00044659	0.00066121	0.03546	0.0004466	0.0006612
N36	0.00076272	0.00071111	0.03569	0.0007627	0.0007111
N38	0.00052824	0.00066121	0.03553	0.0005282	0.0006612

Ca+2	CaCitric	CaHPO4	lgSI(Ca3PO42a	lgSI(Brushite)		tot Pdct x 1000
0.0005625	0.00004251	0.0000334	0.5212	-0.4941		1.258004108
0.000536	0.00004168	0.00002729	0.3249	-0.5819		1.075268817
0.0005451	0.00004198	0.00002908	0.3872	-0.5543		1.127297954
0.0005485	0.00004204	0.00001538	-0.1628	-0.8309		0.589940669
0.0005519	0.00004218	0.0000227	0.1775	-0.662		0.86650652
0.0005086	0.00004083	0.00004198	0.6757	-0.3948		1.751281702
0.0005383	0.00004175	0.00002507	0.2532	-0.6187		0.982608341
0.0005381	0.00004178	0.00003544	0.5534	-0.4684		1.394259096
0.0005072	0.00004077	0.0000334	0.4761	-0.4942		1.391792778
0.0005246	0.0000413	0.00001711	-0.08988	-0.7847		0.684917398
0.0005656	0.00004259	0.00002021	0.08748	-0.7124		0.752736347
0.0005492	0.0000421	0.00002529	0.2694	-0.6149		0.971428838
0.0005549	0.00004228	0.00004021	0.6762	-0.4135		1.539281847
0.0005953	0.00004343	0.00002368	0.2472	-0.6435		0.841281239
0.0005283	0.00004147	0.00004414	0.7359	-0.373		1.776184787
0.0005117	0.00004091	0.00002925	0.3649	-0.5518		1.206391876
0.0005143	0.00004095	0.00001728	-0.08983	-0.7804		0.705794504
0.0005784	0.00004298	0.00002903	0.4116	-0.555		1.061827957
0.0004987	0.00004049	0.00003191	0.4291	-0.514		1.350001007
0.0005926	0.00004337	0.00003641	0.6186	-0.4567		1.305337596
0.0005587	0.00004236	0.00001622	-0.1087	-0.8078		0.610768501
0.0005808	0.00004305	0.00002677	0.343	-0.5903		0.973995669
0.0005292	0.0000415	0.00003342	0.4952	-0.4939		1.335004359
0.0004897	0.0000402	0.00004	0.6175	-0.4158		1.729487031
0.0005522	0.00004215	0.00001175	-0.394	-0.948		0.446592684
0.0005867	0.00004321	0.00002122	0.1456	-0.6913		0.76271548
0.00055	0.00004209	0.00001383	-0.2539	-0.8771		0.528236106

tot Cadct x 10000
6.986376566
6.612106393
6.736863117
6.612106393
6.736863117
6.487349668
6.612106393
6.736863117
6.362592944
6.362592944
6.861619841
6.736863117
6.986376566
7.235890014
6.736863117
6.362592944
6.248752433
7.11113329
6.248752433
7.360646739
6.736863117
7.11113329
6.612106393
6.248752433
6.612106393
7.11113329
6.612106393

code	eGFR	CODE	eGFR
CKD2	21	N2	89
CKD4	23	N3	101
CKD5	34	N4	93
CKD6	41	N6	103
CKD7	19	N7	94
CKD11	33	N8	79
CKD13	22	N9	77
CKD14	14	N10	73
CKD15	22	N11	108
CKD18	36	N13	87
CKD20	20	N14	93
CKD21	28	N15	96
CKD23	35	N16	96
CKD24	20	N17	73
CKD25	44	N18	90
CKD26	18	N20	75
CKD27	28	N21	75
CKD31	23	N24	75
CKD32	29	N25	89
CKD33	28	N27	74
CKD45	29	N29	85
CKD46	42	N31	72
CKD49	42	N32	89
CKD50	49	N33	93
CKD51	34	N35	78
CKD55	28	N36	84
CKD59	47	N38	87
CKD62	27		

code	pth 1-84	CODE	[PTH]1-84
CKD2	158	N2	21
CKD4	41	N3	44
CKD5	59	N4	45
CKD6	54	N6	31
CKD7	129	N7	18
CKD11	50	N8	24
CKD13	56	N9	36
CKD14	145	N10	22
CKD15	156	N11	60
CKD18	67	N13	28
CKD20	182	N14	20
CKD21	126	N15	34
CKD23	63	N16	17
CKD24	103	N17	29
CKD25	42	N18	25
CKD26	69	N20	19
CKD27	72	N21	26
CKD31	31	N24	21
CKD32	91	N25	41
CKD33	54	N27	16
CKD45	127	N29	23
CKD46	39	N31	19
CKD49	48	N32	24
CKD50	48	N33	65
CKD51	73	N35	24
CKD55	32	N36	25
CKD59	28	N38	26
CKD62	178		

code	Cai M x 1000	CODE	Cai M x 1000
CKD2	1.15077384	N2	1.25031203
CKD4	1.27059411	N3	1.22030454
CKD5	1.20069895	N4	1.28031952
CKD6	1.27059411	N6	1.24030954
CKD7	1.23065402	N7	1.26031453
CKD11	1.23065402	N8	1.23030704
CKD13	1.26060909	N9	1.27031702
CKD14	1.13080379	N10	1.28031952
CKD15	1.18072891	N11	1.24030954
CKD18	1.21068397	N13	1.20029955
CKD20	1.26060909	N14	1.26031453
CKD21	1.19071393	N15	1.27031702
CKD23	1.28057913	N16	1.29032202
CKD24	1.18072891	N17	1.21030205
CKD25	1.3105342	N18	1.27031702
CKD26	1.36045931	N20	1.28031952
CKD27	1.28057913	N21	1.25031203
CKD31	1.28057913	N24	1.27031702
CKD32	1.20069895	N25	1.25031203
CKD33	1.25062406	N27	1.31032701
CKD45	1.20069895	N29	1.25031203
CKD46	1.24063904	N31	1.26031453
CKD49	1.24063904	N32	1.27031702
CKD50	1.220669	N33	1.25031203
CKD51	1.27059411	N35	1.24030954
CKD55	1.25062406	N36	1.24030954
CKD59	1.25062406	N38	1.30032451
CKD62	1.29056415		

code	Cauf M x 1000	CODE	Cauf M x 1000
CKD2	1.32301548	N2	1.39790315
CKD4	1.34797803	N3	1.32301548
CKD5	1.32301548	N4	1.34797803
CKD6	1.34797803	N6	1.32301548
CKD7	1.32301548	N7	1.34797803
CKD11	1.32301548	N8	1.29805292
CKD13	1.29805292	N9	1.32301548
CKD14	1.32301548	N10	1.34797803
CKD15	1.24812781	N11	1.27309036
CKD18	1.34797803	N13	1.27309036
CKD20	1.4228657	N14	1.37294059
CKD21	1.32301548	N15	1.34797803
CKD23	1.32301548	N16	1.39790315
CKD24	1.34797803	N17	1.44782826
CKD25	1.39790315	N18	1.29805292
CKD26	1.39790315	N20	1.27309036
CKD27	1.34797803	N21	1.25031203
CKD31	1.47279081	N24	1.4228657
CKD32	1.29805292	N25	1.25031203
CKD33	1.29805292	N27	1.47279081
CKD45	1.39790315	N29	1.34797803
CKD46	1.27309036	N31	1.4228657
CKD49	1.29805292	N32	1.32301548
CKD50	1.34797803	N33	1.25031203
CKD51	1.34797803	N35	1.32301548
CKD55	1.27309036	N36	1.4228657
CKD59	1.39790315	N38	1.32301548
CKD62	1.29805292		

code	[P]s	CODE	[P]s
CKD2	2.7	N2	3.1
CKD4	2.5	N3	3.5
CKD5	2.5	N4	3.5
CKD6	4.2	N6	3
CKD7	3.8	N7	3.5
CKD11	3.2	N8	2.8
CKD13	3.7	N9	3.2
CKD14	5.3	N10	3.4
CKD15	4.4	N11	2.9
CKD18	3.3	N13	4.9
CKD20	3.7	N14	2.1
CKD21	4.4	N15	3.1
CKD23	3.4	N16	3.4
CKD24	4.8	N17	3.4
CKD25	3.9	N18	4
CKD26	4.5	N20	4.1
CKD27	2.7	N21	2.9
CKD31	4	N24	4
CKD32	3.9	N25	3.2
CKD33	4.1	N27	3.1
CKD45	2.7	N29	4.2
CKD46	3.2	N31	3.4
CKD49	2.9	N32	3.6
CKD50	3.1	N33	4.9
CKD51	2.8	N35	4
CKD55	2.6	N36	2.7
CKD59	3.5	N38	3.2
CKD62	3.6		

code	tot Cadct M x 10000	CODE	tot Cadct M x 10000
CKD2	3.77834651	N2	6.986376566
CKD4	3.849636067	N3	6.612106393
CKD5	3.77834651	N4	6.736863117
CKD6	3.849636067	N6	6.612106393
CKD7	3.77834651	N7	6.736863117
CKD11	3.77834651	N8	6.487349668
CKD13	3.707056953	N9	6.612106393
CKD14	3.77834651	N10	6.736863117
CKD15	3.56447784	N11	6.362592944
CKD18	3.849636067	N13	6.362592944
CKD20	4.063504737	N14	6.861619841
CKD21	3.77834651	N15	6.736863117
CKD23	3.77834651	N16	6.986376566
CKD24	3.849636067	N17	7.235890014
CKD25	3.99221518	N18	6.736863117
CKD26	3.99221518	N20	6.362592944
CKD27	3.849636067	N21	6.248752433
CKD31	4.206083851	N24	7.11113329
CKD32	3.707056953	N25	6.248752433
CKD33	3.707056953	N27	7.360646739
CKD45	3.99221518	N29	6.736863117
CKD46	3.635767396	N31	7.11113329
CKD49	3.707056953	N32	6.612106393
CKD50	3.849636067	N33	6.248752433
CKD51	3.849636067	N35	6.612106393
CKD55	3.635767396	N36	7.11113329
CKD59	3.99221518	N38	6.612106393
CKD62	3.707056953		

code	tot Pdct M x 1000	CODE	tot Pdct x 1000
CKD2	1.819545022	N2	1.258004108
CKD4	1.851666333	N3	1.075268817
CKD5	1.217583808	N4	1.127297954
CKD6	1.549211345	N6	0.589940669
CKD7	2.035667125	N7	0.86650652
CKD11	0.823519372	N8	1.751281702
CKD13	2.375133827	N9	0.982608341
CKD14	1.972697681	N10	1.394259096
CKD15	2.803379416	N11	1.391792778
CKD18	1.002730841	N13	0.684917398
CKD20	2.390552995	N14	0.752736347
CKD21	2.717888231	N15	0.971428838
CKD23	1.624423963	N16	1.539281847
CKD24	2.934587814	N17	0.841281239
CKD25	1.507005539	N18	1.776184787
CKD26	3.251052512	N20	1.206391876
CKD27	2.413868773	N21	0.705794504
CKD31	2.402101561	N24	1.061827957
CKD32	1.488646998	N25	1.350001007
CKD33	2.37112318	N27	1.305337596
CKD45	2.873783922	N29	0.610768501
CKD46	1.330523249	N31	0.973995669
CKD49	1.241679467	N32	1.335004359
CKD50	0.757468886	N33	1.729487031
CKD51	2.10272281	N35	0.446592684
CKD55	0.714102845	N36	0.76271548
CKD59	0.619205587	N38	0.528236106
CKD62	2.701921071		

code	Ca+2 x 10000
CKD2	2.891
CKD4	2.891
CKD5	2.973
CKD6	2.984
CKD7	2.722
CKD11	3.027
CKD13	2.467
CKD14	2.776
CKD15	2.216
CKD18	3.061
CKD20	2.473
CKD21	2.268
CKD23	2.918
CKD24	2.161
CKD25	3.106
CKD26	2.028
CKD27	2.449
CKD31	2.472
CKD32	2.878
CKD33	2.47
CKD45	2.196
CKD46	2.842
CKD49	2.911
CKD50	3.097
CKD51	2.671
CKD55	2.923
CKD59	3.236
CKD62	2.274

code	Ca+2 x 10000
N2	4.02
N3	4.371
N4	4.267
N6	5.485
N7	4.971
N8	3.191
N9	4.608
N10	3.733
N11	3.694
N13	5.246
N14	5.385
N15	4.661
N16	3.525
N17	5.162
N18	3.181
N20	4.04
N21	5.143
N24	4.487
N25	3.754
N27	3.975
N29	5.587
N31	4.724
N32	3.823
N33	3.198
N35	5.522
N36	5.41
N38	5.501

code	tot Cadct M x 10000	Ca+2 x 10000	% of tot Ca ckd		CODE
CKD2	3.77834651	2.891	76.51495151		N2
CKD4	3.849636067	2.891	75.09800796		N3
CKD5	3.77834651	2.973	78.68521302		N4
CKD6	3.849636067	2.984	77.51382074		N6
CKD7	3.77834651	2.722	72.04209547		N7
CKD11	3.77834651	3.027	80.11440962		N8
CKD13	3.707056953	2.467	66.54874827		N9
CKD14	3.77834651	2.776	73.47129208		N10
CKD15	3.56447784	2.216	62.1689936		N11
CKD18	3.849636067	3.061	79.51400981		N13
CKD20	4.063504737	2.473	60.85879456		N14
CKD21	3.77834651	2.268	60.02625736		N15
CKD23	3.77834651	2.918	77.22954981		N16
CKD24	3.849636067	2.161	56.13517648		N17
CKD25	3.99221518	3.106	77.8014175		N18
CKD26	3.99221518	2.028	50.798865		N20
CKD27	3.849636067	2.449	63.61640315		N21
CKD31	4.206083851	2.472	58.77200949		N24
CKD32	3.707056953	2.878	77.63571038		N25
CKD33	3.707056953	2.47	66.629675		N27
CKD45	3.99221518	2.196	55.007055		N29
CKD46	3.635767396	2.842	78.16781686		N31
CKD49	3.707056953	2.911	78.52590442		N32
CKD50	3.849636067	3.097	80.44916315		N33
CKD51	3.849636067	2.671	69.38318204		N35
CKD55	3.635767396	2.923	80.39568216		N36
CKD59	3.99221518	3.236	81.057755		N38
CKD62	3.707056953	2.274	61.34246192		

tot Cadct M x 10000	Ca+2 x 10000	% of tot Ca cont
6.986376566	4.02	57.54055714
6.612106393	4.371	66.10601434
6.736863117	4.267	63.3380837
6.612106393	5.485	82.95389811
6.736863117	4.971	73.78805111
6.487349668	3.191	49.18803769
6.612106393	4.608	69.69034868
6.736863117	3.733	55.41154593
6.362592944	3.694	58.05809098
6.362592944	5.246	82.45066196
6.861619841	5.385	78.48001091
6.736863117	4.661	69.18650296
6.986376566	3.525	50.45533929
7.235890014	5.162	71.33884
6.736863117	3.181	47.21782148
6.362592944	4.04	63.49612549
6.248752433	5.143	82.30442885
7.11113329	4.487	63.0982407
6.248752433	3.754	60.07599181
7.360646739	3.975	54.00340678
6.736863117	5.587	82.93177259
7.11113329	4.724	66.43104281
6.612106393	3.823	57.81818642
6.248752433	3.198	51.17821572
6.612106393	5.522	83.51347774
7.11113329	5.41	76.07788772
6.612106393	5.501	83.19587849

code	Cacit x 100000 ckd
CKD2	3.11
CKD4	3.11
CKD5	3.15
CKD6	3.17
CKD7	3.01
CKD11	3.18
CKD13	2.85
CKD14	3.04
CKD15	2.68
CKD18	3.2
CKD20	2.85
CKD21	2.71
CKD23	3.13
CKD24	2.64
CKD25	3.23
CKD26	2.53
CKD27	2.84
CKD31	2.85
CKD32	3.1
CKD33	2.85
CKD45	2.66
CKD46	3.08
CKD49	3.12
CKD50	3.22
CKD51	2.98
CKD55	3.12
CKD59	3.29
CKD62	2.72

code	Cacit x 100000 cont
N2	3.68
N3	3.82
N4	3.78
N6	4.21
N7	4.04
N8	3.28
N9	3.91
N10	3.55
N11	3.53
N13	4.13
N14	4.18
N15	3.93
N16	3.45
N17	4.1
N18	3.27
N20	3.69
N21	4.1
N24	3.87
N25	3.56
N27	3.66
N29	4.24
N31	3.95
N32	3.59
N33	3.28
N35	4.22
N36	4.18
N38	4.21

code	tot Cadct M x 10000	Cacit x 100000 ckd	Cacit % of tot Ca ckd
CKD2	3.77834651	3.11	8.231113774
CKD4	3.849636067	3.11	8.078685741
CKD5	3.77834651	3.15	8.336980189
CKD6	3.849636067	3.17	8.23454463
CKD7	3.77834651	3.01	7.966447736
CKD11	3.77834651	3.18	8.41638
CKD13	3.707056953	2.85	7.688039423
CKD14	3.77834651	3.04	8.045847547
CKD15	3.56447784	2.68	7.5186328
CKD18	3.849636067	3.2	8.312474074
CKD20	4.063504737	2.85	7.01365
CKD21	3.77834651	2.71	7.172449623
CKD23	3.77834651	3.13	8.284046981
CKD24	3.849636067	2.64	6.857791111
CKD25	3.99221518	3.23	8.09074625
CKD26	3.99221518	2.53	6.33733375
CKD27	3.849636067	2.84	7.377320741
CKD31	4.206083851	2.85	6.775899153
CKD32	3.707056953	3.1	8.362428846
CKD33	3.707056953	2.85	7.688039423
CKD45	3.99221518	2.66	6.6629675
CKD46	3.635767396	3.08	8.47138902
CKD49	3.707056953	3.12	8.41638
CKD50	3.849636067	3.22	8.364427037
CKD51	3.849636067	2.98	7.740991481
CKD55	3.635767396	3.12	8.581407059
CKD59	3.99221518	3.29	8.24103875
CKD62	3.707056953	2.72	7.337356923

CODE	tot Cadct M x 10000	Cacit x 100000 cont	Cacit % tot Ca cont
N2	6.986376566	3.68	5.267394286
N3	6.612106393	3.82	5.777281509
N4	6.736863117	3.78	5.61092
N6	6.612106393	4.21	6.367108679
N7	6.736863117	4.04	5.996856296
N8	6.487349668	3.28	5.055993846
N9	6.612106393	3.91	5.913395472
N10	6.736863117	3.55	5.269514815
N11	6.362592944	3.53	5.548052549
N13	6.362592944	4.13	6.491064314
N14	6.861619841	4.18	6.091856
N15	6.736863117	3.93	5.833575556
N16	6.986376566	3.45	4.938182143
N17	7.235890014	4.1	5.6662
N18	6.736863117	3.27	4.853891111
N20	6.362592944	3.69	5.799522353
N21	6.248752433	4.1	6.561309708
N24	7.11113329	3.87	5.442170526
N25	6.248752433	3.56	5.69713721
N27	7.360646739	3.66	4.972389153
N29	6.736863117	4.24	6.29373037
N31	7.11113329	3.95	5.554670175
N32	6.612106393	3.59	5.429434717
N33	6.248752433	3.28	5.249047766
N35	6.612106393	4.22	6.382232453
N36	7.11113329	4.18	5.878106667
N38	6.612106393	4.21	6.367108679

code	Caox x 10^7
CKD2	6.64
CKD4	6.64
CKD5	6.82
CKD6	6.85
CKD7	6.28
CKD11	6.94
CKD13	5.73
CKD14	6.4
CKD15	5.18
CKD18	7.01
CKD20	5.74
CKD21	5.29
CKD23	6.7
CKD24	5.05
CKD25	7.09
CKD26	4.75
CKD27	5.69
CKD31	5.74
CKD32	6.62
CKD33	5.74
CKD45	5.13
CKD46	6.54
CKD49	6.69
CKD50	7.09
CKD51	6.17
CKD55	6.73
CKD59	7.38
CKD62	5.3

code	Caox x 10^7
N2	8.94
N3	9.63
N4	9.43
N6	11.7
N7	10.8
N8	7.27
N9	10.1
N10	8.37
N11	8.3
N13	11.3
N14	11.5
N15	10.2
N16	7.95
N17	11.1
N18	7.25
N20	8.98
N21	11.1
N24	9.85
N25	8.41
N27	8.85
N29	11.9
N31	10.3
N32	8.55
N33	7.28
N35	11.8
N36	11.6
N38	11.7

code	tot Cadct M x 10000	Caox x 10^7 ckd	Caox % tot Ca ckd	CODE
CKD2	3.77834651	6.64	0.175738249	N2
CKD4	3.849636067	6.64	0.172483837	N3
CKD5	3.77834651	6.82	0.180502238	N4
CKD6	3.849636067	6.85	0.177938898	N6
CKD7	3.77834651	6.28	0.166210272	N7
CKD11	3.77834651	6.94	0.18367823	N8
CKD13	3.707056953	5.73	0.154570056	N9
CKD14	3.77834651	6.4	0.169386264	N10
CKD15	3.56447784	5.18	0.145322828	N11
CKD18	3.849636067	7.01	0.182095135	N13
CKD20	4.063504737	5.74	0.141257372	N14
CKD21	3.77834651	5.29	0.140008334	N15
CKD23	3.77834651	6.7	0.177326245	N16
CKD24	3.849636067	5.05	0.131181231	N17
CKD25	3.99221518	7.09	0.177595638	N18
CKD26	3.99221518	4.75	0.118981563	N20
CKD27	3.849636067	5.69	0.14780618	N21
CKD31	4.206083851	5.74	0.136468986	N24
CKD32	3.707056953	6.62	0.178578319	N25
CKD33	3.707056953	5.74	0.154839812	N27
CKD45	3.99221518	5.13	0.128500088	N29
CKD46	3.635767396	6.54	0.179879494	N31
CKD49	3.707056953	6.69	0.18046661	N32
CKD50	3.849636067	7.09	0.184173254	N33
CKD51	3.849636067	6.17	0.160274891	N35
CKD55	3.635767396	6.73	0.185105351	N36
CKD59	3.99221518	7.38	0.184859775	N38
CKD62	3.707056953	5.3	0.142970558	

tot Cadct M x 10000	Caox x 10^7 ckd	Ca ox % tot Ca cont
6.986376566	8.94	0.127963329
6.612106393	9.63	0.14564194
6.736863117	9.43	0.139976126
6.612106393	11.7	0.176948151
6.736863117	10.8	0.160312
6.487349668	7.27	0.112064254
6.612106393	10.1	0.152750113
6.736863117	8.37	0.1242418
6.362592944	8.3	0.130449961
6.362592944	11.3	0.177600549
6.861619841	11.5	0.167598909
6.736863117	10.2	0.151405778
6.986376566	7.95	0.113792893
7.235890014	11.1	0.153402
6.736863117	7.25	0.107616852
6.362592944	8.98	0.141137427
6.248752433	11.1	0.177635458
7.11113329	9.85	0.138515193
6.248752433	8.41	0.134586865
7.360646739	8.85	0.120234
6.736863117	11.9	0.176640074
7.11113329	10.3	0.144843298
6.612106393	8.55	0.129308264
6.248752433	7.28	0.116503255
6.612106393	11.8	0.178460528
7.11113329	11.6	0.163124491
6.612106393	11.7	0.176948151

code	$CaHPO_4 \times 10^5$
CKD2	2.52
CKD4	2.56
CKD5	1.74
CKD6	2.22
CKD7	2.64
CKD11	1.21
CKD13	2.77
CKD14	2.61
CKD15	2.92
CKD18	1.48
CKD20	2.77
CKD21	2.89
CKD23	2.27
CKD24	2.96
CKD25	2.24
CKD26	3.06
CKD27	2.78
CKD31	2.77
CKD32	2.06
CKD33	2.77
CKD45	2.94
CKD46	1.82
CKD49	1.74
CKD50	1.13
CKD51	2.66
CKD55	1.01
CKD59	0.97
CKD62	2.89

code	$CaHPO_4 \times 10^5$
N2	2.17
N3	2.08
N4	2.1
N6	1.54
N7	1.95
N8	2.43
N9	2.03
N10	2.25
N11	2.26
N13	1.71
N14	1.87
N15	2.01
N16	2.32
N17	1.91
N18	2.44
N20	2.16
N21	1.73
N24	2.05
N25	2.24
N27	2.18
N29	1.62
N31	2
N32	2.22
N33	2.43
N35	1.18
N36	1.87
N38	1.38

code	tot Cadct M x 10000	CaHPO4 x 10^5	% tot Ca CKD	CODE
CKD2	3.77834651	2.52	6.66958415	N2
CKD4	3.849636067	2.56	6.64997926	N3
CKD5	3.77834651	1.74	4.60518906	N4
CKD6	3.849636067	2.22	5.76677889	N6
CKD7	3.77834651	2.64	6.9871834	N7
CKD11	3.77834651	1.21	3.20245906	N8
CKD13	3.707056953	2.77	7.47223481	N9
CKD14	3.77834651	2.61	6.90778358	N10
CKD15	3.56447784	2.92	8.1919432	N11
CKD18	3.849636067	1.48	3.84451926	N13
CKD20	4.063504737	2.77	6.81677561	N14
CKD21	3.77834651	2.89	7.64884849	N15
CKD23	3.77834651	2.27	6.00791906	N16
CKD24	3.849636067	2.96	7.68903852	N17
CKD25	3.99221518	2.24	5.61092	N18
CKD26	3.99221518	3.06	7.6649175	N20
CKD27	3.849636067	2.78	7.22146185	N21
CKD31	4.206083851	2.77	6.58569847	N24
CKD32	3.707056953	2.06	5.55696885	N25
CKD33	3.707056953	2.77	7.47223481	N27
CKD45	3.99221518	2.94	7.3643325	N29
CKD46	3.635767396	1.82	5.00582078	N31
CKD49	3.707056953	1.74	4.69375038	N32
CKD50	3.849636067	1.13	2.93534241	N33
CKD51	3.849636067	2.66	6.90974407	N35
CKD55	3.635767396	1.01	2.77795549	N36
CKD59	3.99221518	0.97	2.42972875	N38
CKD62	3.707056953	2.89	7.79594173	

tot Cadct M x 10000	CaHPO4 x 10^5	% tot Ca cont
6.986376566	2.17	3.106045
6.612106393	2.08	3.14574491
6.736863117	2.1	3.11717778
6.612106393	1.54	2.32906113
6.736863117	1.95	2.89452222
6.487349668	2.43	3.74575154
6.612106393	2.03	3.07012604
6.736863117	2.25	3.33983333
6.362592944	2.26	3.55201098
6.362592944	1.71	2.68758353
6.861619841	1.87	2.725304
6.736863117	2.01	2.98358444
6.986376566	2.32	3.32074857
7.235890014	1.91	2.63962
6.736863117	2.44	3.6218637
6.362592944	2.16	3.39484235
6.248752433	1.73	2.76855263
7.11113329	2.05	2.88280351
6.248752433	2.24	3.58471555
7.360646739	2.18	2.96169627
6.736863117	1.62	2.40468
7.11113329	2	2.81249123
6.612106393	2.22	3.35747774
6.248752433	2.43	3.88877624
6.612106393	1.18	1.78460528
7.11113329	1.87	2.6296793
6.612106393	1.38	2.08708075

code	CaHCO3+ x 10^5
CKD2	1.4
CKD4	1.4
CKD5	1.44
CKD6	1.45
CKD7	1.32
CKD11	1.47
CKD13	1.19
CKD14	1.34
CKD15	1.07
CKD18	1.49
CKD20	1.19
CKD21	1.09
CKD23	1.41
CKD24	1.04
CKD25	1.5
CKD26	0.972
CKD27	1.18
CKD31	1.19
CKD32	1.39
CKD33	1.19
CKD45	1.06
CKD46	1.38
CKD49	1.41
CKD50	1.51
CKD51	1.29
CKD55	1.42
CKD59	1.58
CKD62	1.09

code	CaHCO3+ x 10^5
N2	1.95
N3	2.12
N4	2.07
N6	2.66
N7	2.41
N8	1.54
N9	2.24
N10	1.81
N11	1.79
N13	2.54
N14	2.61
N15	2.26
N16	1.71
N17	2.5
N18	1.54
N20	1.96
N21	2.5
N24	2.18
N25	1.82
N27	1.93
N29	2.71
N31	2.29
N32	1.85
N33	1.55
N35	2.68
N36	2.62
N38	2.67

code	tot Cadct M x 10000	CaHCO3+ x 10^5 ckd	CaHCO3+ % of tot Ca ckd
CKD2	3.77834651	1.4	3.705324528
CKD4	3.849636067	1.4	3.636707407
CKD5	3.77834651	1.44	3.811190943
CKD6	3.849636067	1.45	3.766589815
CKD7	3.77834651	1.32	3.493591698
CKD11	3.77834651	1.47	3.890590755
CKD13	3.707056953	1.19	3.210093654
CKD14	3.77834651	1.34	3.546524906
CKD15	3.56447784	1.07	3.0018422
CKD18	3.849636067	1.49	3.870495741
CKD20	4.063504737	1.19	2.928506491
CKD21	3.77834651	1.09	2.884859811
CKD23	3.77834651	1.41	3.731791132
CKD24	3.849636067	1.04	2.701554074
CKD25	3.99221518	1.5	3.7573125
CKD26	3.99221518	0.972	2.4347385
CKD27	3.849636067	1.18	3.065224815
CKD31	4.206083851	1.19	2.829235085
CKD32	3.707056953	1.39	3.749605192
CKD33	3.707056953	1.19	3.210093654
CKD45	3.99221518	1.06	2.6551675
CKD46	3.635767396	1.38	3.795622353
CKD49	3.707056953	1.41	3.803556346
CKD50	3.849636067	1.51	3.922448704
CKD51	3.849636067	1.29	3.350966111
CKD55	3.635767396	1.42	3.905640392
CKD59	3.99221518	1.58	3.9577025
CKD62	3.707056953	1.09	2.940337885

CODE	tot Cadct M x 10000	CaHCO3+ x 10^5	CaHCO3+ % of tot Ca cont
N2	6.986376566	1.95	2.791146429
N3	6.612106393	2.12	3.20624
N4	6.736863117	2.07	3.072646667
N6	6.612106393	2.66	4.022923774
N7	6.736863117	2.41	3.577332593
N8	6.487349668	1.54	2.373850769
N9	6.612106393	2.24	3.387725283
N10	6.736863117	1.81	2.68671037
N11	6.362592944	1.79	2.813318431
N13	6.362592944	2.54	3.992083137
N14	6.861619841	2.61	3.803766545
N15	6.736863117	2.26	3.354677037
N16	6.986376566	1.71	2.447620714
N17	7.235890014	2.5	3.455
N18	6.736863117	1.54	2.28593037
N20	6.362592944	1.96	3.080505098
N21	6.248752433	2.5	4.000798602
N24	7.11113329	2.18	3.065615439
N25	6.248752433	1.82	2.912581383
N27	7.360646739	1.93	2.622052203
N29	6.736863117	2.71	4.022643704
N31	7.11113329	2.29	3.220302456
N32	6.612106393	1.85	2.797898113
N33	6.248752433	1.55	2.480495134
N35	6.612106393	2.68	4.053171321
N36	7.11113329	2.62	3.684363509
N38	6.612106393	2.67	4.038047547

code	CaSO4 x 10^5
CKD2	7.16
CKD4	7.16
CKD5	7.4
CKD6	7.41
CKD7	6.73
CKD11	7.56
CKD13	6.09
CKD14	6.87
CKD15	5.45
CKD18	7.63
CKD20	6.11
CKD21	5.59
CKD23	7.23
CKD24	5.31
CKD25	7.7
CKD26	4.97
CKD27	6.04
CKD31	6.1
CKD32	7.15
CKD33	6.1
CKD45	5.4
CKD46	7.07
CKD49	7.25
CKD50	7.73
CKD51	6.61
CKD55	7.32
CKD59	8.1
CKD62	5.6

code	CaSO4 x 10^5
N2	9.94
N3	10.8
N4	10.5
N6	13.5
N7	12.3
N8	7.9
N9	11.4
N10	9.24
N11	9.14
N13	12.9
N14	13.2
N15	11.5
N16	8.72
N17	12.7
N18	7.87
N20	9.99
N21	12.7
N24	11.1
N25	9.29
N27	9.83
N29	13.7
N31	11.7
N32	9.46
N33	7.92
N35	13.6
N36	13.3
N38	13.5

code	tot Cadct M x 10000	CaSO4 x 10^5 ckd	CaSO4% of tot Ca ckd
CKD2	3.77834651	7.16	18.9500883
CKD4	3.849636067	7.16	18.59916074
CKD5	3.77834651	7.4	19.58528679
CKD6	3.849636067	7.41	19.24857278
CKD7	3.77834651	6.73	17.81202434
CKD11	3.77834651	7.56	20.00875245
CKD13	3.707056953	6.09	16.42812635
CKD14	3.77834651	6.87	18.18255679
CKD15	3.56447784	5.45	15.289757
CKD18	3.849636067	7.63	19.82005537
CKD20	4.063504737	6.11	15.03628123
CKD21	3.77834651	5.59	14.79483151
CKD23	3.77834651	7.23	19.13535453
CKD24	3.849636067	5.31	13.79351167
CKD25	3.99221518	7.7	19.2875375
CKD26	3.99221518	4.97	12.44922875
CKD27	3.849636067	6.04	15.68979481
CKD31	4.206083851	6.1	14.50280169
CKD32	3.707056953	7.15	19.2875375
CKD33	3.707056953	6.1	16.45510192
CKD45	3.99221518	5.4	13.526325
CKD46	3.635767396	7.07	19.44568843
CKD49	3.707056953	7.25	19.55729327
CKD50	3.849636067	7.73	20.07982019
CKD51	3.849636067	6.61	17.17045426
CKD55	3.635767396	7.32	20.13330118
CKD59	3.99221518	8.1	20.2894875
CKD62	3.707056953	5.6	15.10632308

CODE	tot Cadct M x 10000	CaSO4 x 10^5 ckd	CaSO4% of tot Ca cont
N2	6.986376566	9.94	
N3	6.612106393	10.8	
N4	6.736863117	10.5	
N6	6.612106393	13.5	
N7	6.736863117	12.3	
N8	6.487349668	7.9	
N9	6.612106393	11.4	
N10	6.736863117	9.24	
N11	6.362592944	9.14	
N13	6.362592944	12.9	
N14	6.861619841	13.2	
N15	6.736863117	11.5	
N16	6.986376566	8.72	
N17	7.235890014	12.7	
N18	6.736863117	7.87	
N20	6.362592944	9.99	
N21	6.248752433	12.7	
N24	7.11113329	11.1	
N25	6.248752433	9.29	
N27	7.360646739	9.83	
N29	6.736863117	13.7	
N31	7.11113329	11.7	
N32	6.612106393	9.46	
N33	6.248752433	7.92	
N35	6.612106393	13.6	
N36	7.11113329	13.3	
N38	6.612106393	13.5	
