# Supplementary material for: Chemical evidence for the tradeoff-in-the-nephron hypothesis to explain secondary hyperparathyroidism
Source: PLoS One. 2022 Aug 1;17(8):e0272380. doi: 10.1371/journal.pone.0272380 (PMC9342777; doi:10.1371/journal.pone.0272380)
Supplement: S3 File — (PDF) [file pone.0272380.s012.pdf]

code	IStr	Tot(P)	Tot(Ca)	Ca+2	CaCitric	CaOxalic	CaHPO4	CaHCO3+
CKD2	0.03563	0.00182	0.000378	0.000296	3.09E-05	6.81E-07	2.00E-05	1.30E-05
CKD4	0.03569	0.001852	0.000385	0.000302	3.12E-05	6.92E-07	2.07E-05	1.32E-05
CKD5	0.03519	0.001218	0.000378	0.000303	3.12E-05	6.96E-07	1.38E-05	1.33E-05
CKD6	0.03538	0.001549	0.000385	0.000305	3.14E-05	7.00E-07	1.76E-05	1.34E-05
CKD7	0.03583	0.002036	0.000378	0.000294	3.08E-05	6.76E-07	2.22E-05	1.29E-05
CKD11	0.03488	0.000824	0.000378	0.000307	3.14E-05	7.05E-07	9.46E-06	1.35E-05
CKD13	0.03606	0.002375	0.000371	0.000285	3.03E-05	6.56E-07	2.50E-05	1.24E-05
CKD14	0.03587	0.001973	0.000378	0.000295	3.08E-05	6.77E-07	2.15E-05	1.29E-05
CKD15	0.03646	0.002803	0.000356	0.000269	2.93E-05	6.22E-07	2.78E-05	1.17E-05
CKD18	0.03503	0.001003	0.000385	0.000311	3.16E-05	7.13E-07	1.17E-05	1.37E-05
CKD20	0.03614	0.002391	0.000406	0.000313	3.19E-05	7.14E-07	2.76E-05	1.37E-05
CKD21	0.03638	0.002718	0.000378	0.000287	3.04E-05	6.59E-07	2.87E-05	1.25E-05
CKD23	0.03556	0.001624	0.000378	0.000298	3.10E-05	6.85E-07	1.80E-05	1.31E-05
CKD24	0.03659	0.002935	0.000385	0.00029	3.06E-05	6.65E-07	3.13E-05	1.26E-05
CKD25	0.03551	0.001507	0.000399	0.000317	3.20E-05	7.25E-07	1.78E-05	1.39E-05
CKD26	0.03686	0.003251	0.000399	0.000298	3.11E-05	6.81E-07	3.55E-05	1.29E-05
CKD27	0.03625	0.002414	0.000385	0.000296	3.09E-05	6.78E-07	2.63E-05	1.29E-05
CKD31	0.03625	0.002402	0.000421	0.000324	3.25E-05	7.37E-07	2.87E-05	1.41E-05
CKD32	0.03543	0.001489	0.000371	0.000294	3.07E-05	6.76E-07	1.63E-05	1.29E-05
CKD33	0.0361	0.002371	0.000371	0.000285	3.03E-05	6.56E-07	2.50E-05	1.24E-05
CKD45	0.03658	0.002874	0.000399	0.000302	3.13E-05	6.90E-07	3.19E-05	1.31E-05
CKD46	0.03532	0.001331	0.000364	0.00029	3.04E-05	6.68E-07	1.44E-05	1.27E-05
CKD49	0.0352	0.001242	0.000371	0.000297	3.08E-05	6.83E-07	1.37E-05	1.30E-05
CKD50	0.03488	0.000758	0.000385	0.000314	3.17E-05	7.19E-07	8.89E-06	1.38E-05
CKD51	0.03586	0.002103	0.000385	0.000299	3.11E-05	6.86E-07	2.33E-05	1.31E-05
CKD55	0.03477	0.000714	0.000364	0.000296	3.07E-05	6.83E-07	7.92E-06	1.31E-05
CKD59	0.0347	0.000619	0.000399	0.000328	3.25E-05	7.49E-07	7.60E-06	1.44E-05
CKD62	0.03641	0.002702	0.000371	0.000281	3.01E-05	6.47E-07	2.80E-05	1.23E-05
N2	0.03575	0.001258	0.000699	0.000573	4.22E-05	1.22E-06	2.64E-05	2.50E-05
N3	0.03556	0.001075	0.000661	0.000545	4.13E-05	1.17E-06	2.15E-05	2.38E-05
N4	0.03555	0.001127	0.000674	0.000555	4.16E-05	1.18E-06	2.30E-05	2.43E-05
N6	0.03521	0.00059	0.000661	0.000555	4.16E-05	1.18E-06	1.21E-05	2.43E-05
N7	0.0353	0.000867	0.000674	0.00056	4.18E-05	1.19E-06	1.79E-05	2.45E-05
N8	0.03597	0.001751	0.000649	0.000491	3.96E-05	1.07E-06	3.11E-05	2.14E-05
N9	0.03545	0.000983	0.000661	0.000547	4.14E-05	1.17E-06	1.98E-05	2.39E-05
N10	0.03569	0.001394	0.000674	0.000549	4.15E-05	1.17E-06	2.81E-05	2.40E-05
N11	0.03571	0.001392	0.000636	0.000517	4.05E-05	1.12E-06	2.65E-05	2.26E-05
N13	0.03516	0.000685	0.000636	0.000531	4.09E-05	1.14E-06	1.34E-05	2.33E-05
N14	0.03524	0.000753	0.000686	0.000573	4.22E-05	1.22E-06	1.59E-05	2.51E-05
N15	0.03537	0.000971	0.000674	0.000558	4.17E-05	1.19E-06	1.99E-05	2.44E-05
N16	0.03587	0.001539	0.000699	0.000536	4.11E-05	1.15E-06	2.98E-05	2.34E-05
N17	0.03552	0.000841	0.000724	0.000604	4.30E-05	1.27E-06	1.86E-05	2.64E-05
N18	0.03597	0.001776	0.000674	0.00049	3.96E-05	1.06E-06	3.11E-05	2.14E-05
N20	0.03566	0.001206	0.000636	0.000521	4.06E-05	1.12E-06	2.31E-05	2.28E-05
N21	0.03516	0.000706	0.000625	0.000521	4.05E-05	1.12E-06	1.36E-05	2.29E-05
N24	0.03546	0.001062	0.000711	0.000588	4.26E-05	1.24E-06	2.29E-05	2.57E-05
N25	0.03561	0.00135	0.000625	0.000509	4.02E-05	1.10E-06	2.53E-05	2.22E-05

N27	0.03578	0.001305	0.000736	0.000595	4.28E-05	1.25E-06	2.83E-05	2.60E-05
N29	0.03517	0.000611	0.000674	0.000565	4.19E-05	1.20E-06	1.27E-05	2.48E-05
N31	0.0354	0.000974	0.000711	0.00059	4.27E-05	1.25E-06	2.11E-05	2.58E-05
N32	0.03563	0.001335	0.000661	0.00054	4.12E-05	1.16E-06	2.65E-05	2.36E-05
N33	0.03595	0.001729	0.000625	0.000492	3.96E-05	1.07E-06	3.11E-05	2.15E-05
N35	0.03503	0.000447	0.000661	0.000558	4.17E-05	1.19E-06	9.20E-06	2.45E-05
N36	0.03524	0.000763	0.000711	0.000595	4.28E-05	1.26E-06	1.67E-05	2.61E-05
N38	0.03509	0.000528	0.000661	0.000556	4.16E-05	1.19E-06	1.09E-05	2.44E-05
CKD16	0.03578	0.001797	0.000428	0.000337	3.31E-05	7.65E-07	2.24E-05	1.48E-05
CKD41	0.03592	0.002144	0.000307	0.000236	2.71E-05	5.51E-07	1.88E-05	1.03E-05

CaSO4	lgSI(Ca3PO42am.,s)	lgSI(Brushite)
7.38E-06	-0.6021	-0.7167
7.51E-06	-0.5638	-0.7016
7.58E-06	-0.9184	-0.8799
7.62E-06	-0.7018	-0.773
7.31E-06	-0.5165	-0.6722
7.72E-06	-1.237	-1.042
7.07E-06	-0.4263	-0.62
7.33E-06	-0.541	-0.6849
6.66E-06	-0.3582	-0.5736
7.80E-06	-1.05	-0.9518
7.75E-06	-0.2992	-0.5769
7.09E-06	-0.3026	-0.5596
7.44E-06	-0.6905	-0.7626
7.16E-06	-0.223	-0.5222
7.91E-06	-0.6758	-0.7686
7.32E-06	-0.1028	-0.4677
7.32E-06	-0.3648	-0.5974
8.01E-06	-0.2502	-0.5601
7.35E-06	-0.7843	-0.8063
7.06E-06	-0.4278	-0.6208
7.44E-06	-0.1896	-0.5142
7.25E-06	-0.8993	-0.8607
7.43E-06	-0.9282	-0.8803
7.89E-06	-1.281	-1.069
7.43E-06	-0.4669	-0.651
7.46E-06	-1.406	-1.119
8.24E-06	-1.399	-1.138
6.96E-06	-0.3325	-0.5704
1.41E-05	-0.07434	-0.5961
1.34E-05	-0.2733	-0.6848
1.36E-05	-0.21	-0.6569
1.37E-05	-0.7678	-0.9361
1.38E-05	-0.4236	-0.7659
1.21E-05	-8.28E-07	-0.5252
1.35E-05	-0.3461	-0.722
1.35E-05	-0.03996	-0.5696
1.27E-05	-0.1175	-0.5955
1.31E-05	-0.6939	-0.8897
1.41E-05	-0.5152	-0.8168
1.37E-05	-0.3301	-0.7183
1.32E-05	8.28E-07	-0.5444
1.48E-05	-0.3544	-0.7476
1.20E-05	-2.07E-07	-0.5246
1.28E-05	-0.232	-0.6543
1.29E-05	-0.6929	-0.885
1.45E-05	-0.186	-0.6577
1.25E-05	-0.1652	-0.6157

1.46E-05	6.21E-07	-0.567
1.40E-05	-0.7135	-0.913
1.45E-05	-0.2562	-0.6935
1.33E-05	-0.09908	-0.5955
1.21E-05	6.21E-07	-0.5257
1.38E-05	-1.001	-1.054
1.46E-05	-0.4567	-0.7956
1.38E-05	-0.8599	-0.9827
8.37E-06	-0.4468	-0.6672
5.88E-06	-0.7572	-0.7446

code	[P]s	EP	Ecr	EP/Ecr	[P]u	[cr]s	[cr]u	EP/Ccr	TRP/Ccr	FEP	FTRP	pth 1-84
CKD2	2.7	597	1024.3	0.582837	27.1	2.9	53.4	1.471723	1.228277	0.545083	0.454917	158
CKD4	2.5	665.4	1458	0.456379	47.7	2	144.5	0.660208	1.839792	0.264083	0.735917	41
CKD5	2.5	646.8	1468	0.440599	45.3	2.1	156.2	0.609027	1.890973	0.243611	0.756389	59
CKD6	4.2	992.4	1157.2	0.857587	93.2	1.7	91.2	1.737281	2.462719	0.413638	0.586362	54
CKD7	3.8	604.3	1228.9	0.491741	55.3	3.4	127.9	1.470055	2.329945	0.386857	0.613143	129
CKD11	3.2	424.6	731	0.580848	42.2	2	99.5	0.848241	2.351759	0.265075	0.734925	50
CKD13	3.7	816.4	1224.6	0.666667	29.5	2.8	49.2	1.678862	2.02114	0.453746	0.546254	56
CKD14	5.3	431.5	1148.5	0.375707	43.3	4.8	101.7	2.043658	3.25634	0.385596	0.614404	145
CKD15	4.4	963.6	1195.3	0.806157	29.5	2.9	40	2.13875	2.26125	0.48608	0.51392	156
CKD18	3.3	564	746	0.756032	40.5	1.8	61.5	1.185366	2.114634	0.359202	0.640798	67
CKD20	3.7	747	1164	0.641753	34.6	3.1	63.2	1.697152	2.002848	0.45869	0.54131	182
CKD21	4.4	1189	1372.6	0.866239	79	2.3	108.9	1.668503	2.731497	0.379205	0.620795	126
CKD23	3.4	888.3	1076	0.825558	31	1.9	39.4	1.494924	1.905076	0.439683	0.560317	63
CKD24	4.8	917	1311	0.699466	44.1	3	53.8	2.459108	2.340892	0.512314	0.487686	103
CKD25	3.9	1036	1922.2	0.538966	22.6	1.6	34.6	1.045087	2.854913	0.267971	0.732029	42
CKD26	4.5	914.3	1293.6	0.706787	47.6	3.5	64.7	2.574961	1.925039	0.572214	0.427786	69
CKD27	2.7	1056	1339	0.788648	100.1	2.3	100.1	2.3	1.610889	0.851852	0.148148	72
CKD31	4	863.2	1169.6	0.73803	65.2	2.7	81.9	2.149451	1.85055	0.537363	0.462637	31
CKD32	3.9	674.5	853.6	0.790183	41	2.2	46.6	1.935622	1.964378	0.496313	0.503687	91
CKD33	4.1	1037.3	1605.9	0.645931	52	2.3	97.8	1.222904	2.877096	0.298269	0.701731	54
CKD45	2.7	1302.1	1710.4	0.761284	58.3	2.2	75.2	1.705585	0.994415	0.631698	0.368302	127
CKD46	3.2	873.1	1674.2	0.521503	43.6	1.6	178.7	0.390375	2.80963	0.121992	0.878008	39
CKD49	2.9	814.8	1146.5	0.710685	41	1.6	68	0.964706	1.935294	0.332657	0.667343	48
CKD50	3.1	579.9	1055	0.549668	19.7	1.4	46.3	0.59568	2.50432	0.192155	0.807845	48
CKD51	2.8	1117	1866	0.598607	43.4	2	126.8	0.684543	2.11546	0.244479	0.755521	73
CKD55	2.6	312.4	1167.3	0.267626	10.6	2.8	70.3	0.422191	2.17781	0.162381	0.837619	32
CKD59	3.5	454.7	1080.7	0.420746	38.5	1.7	104.2	0.628119	2.871881	0.179463	0.820537	28
CKD62	3.6	1139.8	1536.3	0.741912	19.3	2.8	26.1	2.070498	1.529502	0.575138	0.424862	178
CKD16	3.8	617.6	865.6	0.713494	69.5	2.7	99	1.895455	1.904545	0.498804	0.501196	169
CKD41	2.7	904.5	1679.1	0.538681	14.5	2.4	53.2	0.654135	2.04586	0.242272	0.757728	79

FGF23	1,25	eGFR	100/eGFR	25D	Cai	ECa/Ccr	[Ca]uf
35.403	25.1	21	4.761905	37.4	4.61	0.168	5.3
13.383	38	23	4.347826	42.2	5.09	0.039	5.4
13.543	55.9	34	2.941176	47.4	4.81	0.027	5.3
26.494	74.7	41	2.439024	44.3	5.09	0.097	5.4
30.706	39.6	19	5.263158	41.7	4.93	0.053	5.3
17.384	89.6	33	3.030303	55.8	4.93	0.046	5.3
23.805	20.4	22	4.545455	21	5.05	0.114	5.2
68.316	64.2	14	7.142857	34.2	4.53	0.094	5.3
26	27.6	22	4.545455	18.9	4.73	0.145	5
17.415	57.2	36	2.777778	42.7	4.85	0.059	5.4
43.713	33.7	20	5	49.1	5.05	0.123	5.7
26.399	19.3	28	3.571429	20.5	4.77	0.034	5.3
35.711	49.6	35	2.857143	27.7	5.13	0.019	5.3
48.446	25.1	20	5	31	4.73	0.028	5.4
12.507	52.6	44	2.272727	lost	5.25	0.069	5.6
35.564	21.4	18	5.555556	25.6	5.45	0.119	5.6
42.96	44.9	28	3.571429	26.4	5.13	0.018	5.4
24.519		23	4.347826	27.5	5.13	0.105	5.9
89.109	21.7	29	3.448276	32.2	4.81	0.038	5.2
26.285	27.3	28	3.571429	49.5	5.01	0.019	5.2
25.303	47.8	29	3.448276	30.1	4.81	0.009	5.6
17.44	31.8	42	2.380952	24	4.97	0.037	5.1
48.504	25.5	42	2.380952	24.6	4.97	0.08	5.2
14.623	75.1	49	2.040816	36.9	4.89	0.07	5.4
19.062	26.5	34	2.941176	17.8	5.09	0.002	5.4
20.402	43.4	28	3.571429	35.9	5.01	0.092	5.1
16.126	74.9	47	2.12766	51.9	5.01	0.011	5.6
20.506	53.1	27	3.703704	21.5	5.17	0.21	5.2
36.399	58.6	22	4.545455	71.2	5.41	0.055	6
38.187	30	27	3.703704	17.4	4.89	0.014	4.3

CODE	[cr]s	eGFR	[P]s	[Ca]i	[Ca]uf	[PTH]1-84	1-84 & 7-84	[PTH]7-84
N2	0.9	89	3.1	5.00875	5.6	21	24	3
N3	0.8	101	3.5	4.88854	5.3	44	77	33
N4	0.7	93	3.5	5.12896	5.4	45	72	27
N6	0.8	103	3	4.96868	5.3	31	52	21
N7	0.7	94	3.5	5.04882	5.4	18	28	10
N8	1	79	2.8	4.92861	5.2	24	29	5
N9	0.8	77	3.2	5.08889	5.3	36	57	21
N10	0.8	73	3.4	5.12896	5.4	22	31	9
N11	0.8	108	2.9	4.96868	5.1	60	120	60
N13	0.7	87	4.9	4.8084	5.1	28	50	22
N14	0.9	93	2.1	5.04882	5.5	20	30	10
N15	0.7	96	3.1	5.08889	5.4	34	59	25
N16	0.9	96	3.4	5.16903	5.6	17	22	5
N17	1.1	73	3.4	4.84847	5.8	29	49	20
N18	0.7	90	4	5.08889	5.2	25	45	20
N20	0.8	75	4.1	5.12896	5.1	19	32	13
N21	0.8	75	2.9	5.00875	5.00875	26	51	25
N24	0.8	75	4	5.08889	5.7	21	36	15
N25	1.1	89	3.2	5.00875	5.00875	41	82	41
N27	0.8	74	3.1	5.24917	5.9	16	27	11
N29	0.7	85	4.2	5.00875	5.4	23	46	23
N31	0.9	72	3.4	5.04882	5.7	19	30	11
N32	1.3	89	3.6	5.08889	5.3	24	36	12
N33	0.9	93	4.9	5.00875	5.00875	65	93	28
N35	0.9	78	4	4.96868	5.3	24	51	27
N36	1	84	2.7	4.96868	5.7	25	47	22
N38	0.7	87	3.2	5.2091	5.3	26	49	23
N19	1	78	3.2	5.24917	5.4	23	38	13

25D	1,25D	FGF23	24h EP	24h Ecr	24h EP/Ecr	24h EP/Ccr	spot EP/Ccr
40.5	66.7	11.018	999.6	1493.8	0.66916589	0.602249297	0.44787
27.7	38.7	4.5325	969.6	1717.8	0.56444289	0.451554314	0.41669
35.3	71.8	23.379	936	1173	0.79795396	0.558567775	0.470909
55.8	66.1	117.63	542.5	1354.8	0.40042811	0.320342486	0.186121
33.5	60	9.3336	727.2	937.3	0.77584551	0.54309186	0.438913
30.8	35	14.798	1235.2	2084.7	0.59250732	0.592507315	0.278234
46.2	55.3	17.591	675.5	1196.4	0.5646105	0.451688399	0.3855
47.8	47	12.978	908.7	1238.4	0.73376938	0.587015504	0.380812
20	90.9	15.294	1342	1105.4	1.21404017	0.971232133	0.45463
25.4	23.4	12.749	532	910	0.58461538	0.409230769	0.583639
24.9	25.8	34.843	625	1416.1	0.44135301	0.397217711	0.21772
22.1	83.3	16.026	832.6	1279.5	0.65072294	0.455506057	0.445848
30.7	68.5	32.967	1319.3	1731.6	0.76189651	0.685706861	0.589039
21.2	53.9	8.3066	548.3	2387.5	0.22965445	0.252619895	0.287347
27.4	28.1	16.158	1427.2	1272.9	1.12121926	0.784853484	0.43
32.5	35.1	13.3182	807.8	1426.5	0.56628111	0.453024886	0.343947
n/a	36.4	12.5182	472.6	867.8	0.54459553	0.435676423	0.292998
19.8	52.8	12.1842	711	1138.5	0.62450593	0.499604743	0.38037
17.5	31.7	7.932	1072.7	2022.9	0.53027831	0.583306145	0.668646
98.1	136.3	10.6568	862.4	1252.8	0.68837803	0.550702427	0.366213
31.1	91	11.7211	463.5	1039.5	0.44588745	0.312121212	0.225379
22.9	94.2	12.6715	626.1	1058.3	0.59160918	0.532448266	0.38008
40.4	77.1	10.5577	882	2416	0.36506623	0.474586093	0.31234
15.2	25	19.7492	1436	2001.7	0.71739022	0.645651196	0.46075
29.6	36.7	15.9907	311	861.7	0.36091447	0.324823024	0.58508
21.9	41.6	5.8652	572	805.2	0.71038251	0.710382514	0.5
30.3	64.6	23.7653	410.3	801.5	0.51191516	0.358340611	0.110526
17	38.5	14.5962	n/a	n/a	n/a	n/a	0.8197941

spot TRP/Ccr	24h ECa	24h ECa/Ecr	24h ECa/Ccr	spot ECa/Ccr	spot TRCa/Ccr	100/eGFR
2.65213	127.5	0.085352792	0.076817512	0.08872	5.51128	1.123596
3.08331	139.2	0.081033881	0.064827104	0.02369	5.27631	0.990099
3.029091	71.2	0.060699062	0.042489344	0.082197	5.317803	1.075269
2.813879	49	0.0361677	0.02893416	0.008181	5.281819	0.970874
3.061087	96.3	0.102741918	0.071919343	0.091483	5.308517	1.06383
2.521766	110.9	0.053197103	0.053197103	0.067762	5.132238	1.265823
2.8145	104.5	0.087345369	0.069876296	0.04264	5.25736	1.298701
3.019188	138.5	0.111837855	0.089470284	0.066421	5.333579	1.369863
2.44537	263.4	0.238284784	0.190627827	0.11654	4.98	0.925926
4.316361	210	0.230769231	0.161538462	0.116361	4.983639	1.149425
1.88228	103.7	0.073229292	0.065906363	0.044376	5.455624	1.075269
2.654152	75.9	0.059320047	0.041524033	0.022022	5.377978	1.041667
2.810961	158.2	0.091360591	0.082224532	0.084353	5.515647	1.041667
3.112653	122.4	0.051267016	0.056393717	0.044898	5.755102	1.369863
3.57	236.8	0.186031896	0.130222327	0.05125	5.14875	1.111111
3.756053	100.9	0.070732562	0.05658605	0.014674	5.085326	1.333333
2.607002	60.9	0.07017746	0.056141968	0.010054	4.998696	1.333333
3.61963	344.4	0.302503294	0.242002635	0.07507	5.62493	1.333333
2.531354	169.6	0.083840032	0.092224035	0.042271	4.966479	1.123596
2.733787	227.9	0.181912516	0.145530013	0.13279	5.76703	1.351351
3.974621	37	0.035594036	0.024915825	0.031818	5.368182	1.176471
3.01992	54.1	0.05111972	0.046007748	0.024502	5.675498	1.388889
3.28766	97	0.040149007	0.052193709	0.0726	5.2274	1.123596
4.43925	113.1	0.056501973	0.050851776	0.01593	4.99282	1.075269
3.41492	116.6	0.135313914	0.121782523	0.01924	5.28076	1.282051
2.2	53.9	0.066939891	0.066939891	n/a	5.7	1.190476
3.089474	200	0.249532127	0.174672489	0.090526	5.209474	1.149425
3.0102059	n/a	n/a	n/a	0.0734109	5.3265891	1.282051

code	eGFR	Tot(P)	Tot(Ca)
CKD2	21	0.00182	0.0003778
CKD4	23	0.001852	0.000385
CKD5	34	0.001218	0.0003778
CKD6	41	0.001549	0.000385
CKD7	19	0.002036	0.0003778
CKD11	33	0.0008235	0.0003778
CKD13	22	0.002375	0.0003707
CKD14	14	0.001973	0.0003778
CKD15	22	0.002803	0.0003564
CKD18	36	0.001003	0.000385
CKD20	20	0.002391	0.0004063
CKD21	28	0.002718	0.0003778
CKD23	35	0.001624	0.0003778
CKD24	20	0.002935	0.000385
CKD25	44	0.001507	0.0003992
CKD26	18	0.003251	0.0003992
CKD27	28	0.002414	0.000385
CKD31	23	0.002402	0.0004206
CKD32	29	0.001489	0.0003707
CKD33	28	0.002371	0.0003707
CKD45	29	0.002874	0.0003992
CKD46	42	0.001331	0.0003636
CKD49	42	0.001242	0.0003707
CKD50	49	0.0007575	0.000385
CKD51	34	0.002103	0.000385
CKD55	28	0.0007141	0.0003636
CKD59	47	0.0006192	0.0003992
CKD62	27	0.002702	0.0003707
N2	89	0.001258	0.0006986
N3	101	0.001075	0.0006612
N4	93	0.001127	0.0006737
N6	103	0.0005899	0.0006612
N7	94	0.0008665	0.0006737
N8	79	0.001751	0.0006487
N9	77	0.0009826	0.0006612
N10	73	0.001394	0.0006737
N11	108	0.001392	0.0006363
N13	87	0.0006849	0.0006363
N14	93	0.0007527	0.0006862
N15	96	0.0009714	0.0006737
N16	96	0.001539	0.0006986
N17	73	0.0008413	0.0007236
N18	90	0.001776	0.0006737
N20	75	0.001206	0.0006363
N21	75	0.0007058	0.0006249
N24	75	0.001062	0.0007111

code	eGFR
CKD2	21
CKD4	23
CKD5	34
CKD6	41
CKD7	19
CKD11	33
CKD13	22
CKD14	14
CKD15	22
CKD18	36
CKD20	20
CKD21	28
CKD23	35
CKD24	20
CKD25	44
CKD26	18
CKD27	28
CKD31	23
CKD32	29
CKD33	28
CKD45	29
CKD46	42
CKD49	42
CKD50	49
CKD51	34
CKD55	28
CKD59	47
CKD62	27
N2	89
N3	101
N4	93
N6	103
N7	94
N8	79
N9	77
N10	73
N11	108
N13	87
N14	93
N15	96
N16	96
N17	73
N18	90
N20	75
N21	75
N24	75

N25	89	0.00135	0.0006249	N25	89
N27	74	0.001305	0.0007361	N27	74
N29	85	0.0006108	0.0006737	N29	85
N31	72	0.000974	0.0007111	N31	72
N32	89	0.001335	0.0006612	N32	89
N33	93	0.001729	0.0006249	N33	93
N35	78	0.0004466	0.0006612	N35	78
N36	84	0.0007627	0.0007111	N36	84
N38	87	0.0005282	0.0006612	N38	87

P ckd	P ctrl	Ca ckd	Ca ctrl
1.82		3.778	
1.852		3.85	
1.218		3.778	
1.549		3.85	
2.036		3.778	
0.8235		3.778	
2.375		3.707	
1.973		3.778	
2.803		3.564	
1.003		3.85	
2.391		4.063	
2.718		3.778	
1.624		3.778	
2.935		3.85	
1.507		3.992	
3.251		3.992	
2.414		3.85	
2.402		4.206	
1.489		3.707	
2.371		3.707	
2.874		3.992	
1.331		3.636	
1.242		3.707	
0.7575		3.85	
2.103		3.85	
0.7141		3.636	
0.6192		3.992	
2.702		3.707	
	1.258		6.986
	1.075		6.612
	1.127		6.737
	0.5899		6.612
	0.8665		6.737
	1.751		6.487
	0.9826		6.612
	1.394		6.737
	1.392		6.363
	0.6849		6.363
	0.7527		6.862
	0.9714		6.737
	1.539		6.986
	0.8413		7.236
	1.776		6.737
	1.206		6.363
	0.7058		6.249
	1.062		7.111

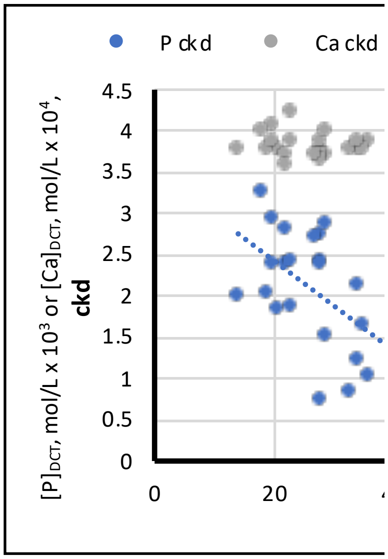

SUMMARY OUTPUT

Regression Statistics	
Multiple R	0.68359294
R Square	0.46729931
Adjusted R Sq	0.44681082
Standard Erro	0.5600378
Observations	28

ANOVA

	df
Regression	1
Residual	26
Total	27

	Coefficients
Intercept	3.5153782
X Variable 1	-0.0544657

SUMMARY OUTPUT

Regression Statistics	
Multiple R	0.09776243
R Square	0.00955749
Adjusted R Sq	-0.0300602
Standard Erro	0.38828079
Observations	27

1.35	6.249		
1.305	7.361	ANOVA	
0.6108	6.737		*df*
0.974	7.111	Regression	1
1.335	6.612	Residual	25
1.729	6.249	Total	26
0.4466	6.612		
0.7627	7.111		*Coefficients*
0.5282	6.612	Intercept	0.75779685
		X Variable 1	0.00367521

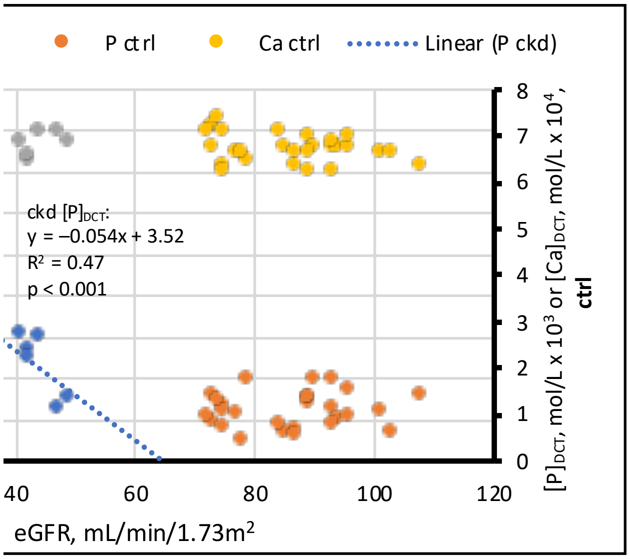

ckd total [P]DCT

SUMMARY OUTPUT

Regression Statistics	
Multiple R	0.00349298
R Square	1.2201E-05
Adjusted R Sq	-0.0384489
Standard Erro	0.14438813
Observations	28

ANOVA

	df	*SS*	*MS*	*F*	*Significance F*
Regression	1	7.153521801	7.1535218	22.807896	6.0744E-05
Residual	26	8.154700746	0.31364234		
Total	27	15.30822255			

	Coefficients	*Standard Error*	*t Stat*	*P-value*	*Lower 95%*	*Upper 95%*	*Lower 95.0%*	*Upper 95.0%*
Intercept	3.82000782	0.356577872	9.85865495	2.8492E-10	2.78242189	4.24833452	2.78242189	4.24833452
X Variable 1	5.237E-05	0.011404602	-4.7757613	6.0744E-05	-0.0779082	-0.0310232	-0.0779082	-0.0310232

ctrl total [P]DCT

SS	MS	F	Significance F
0.036370271	0.03637027	0.241243	0.62759406
3.76904933	0.15076197		
3.805419601			

Standard Error	t Stat	P-value	Lower 95%	Upper 95%	Lower 95.0%	Upper 95.0%
0.649482446	1.16677033	0.25431262	-0.5798373	2.09543098	-0.5798373	2.09543098
0.007482636	0.49116494	0.62759406	-0.0117356	0.01908599	-0.0117356	0.01908599

ctrl total [P]DCT

SS	MS	F	Significance F
6.61354E-06	6.6135E-06	0.00031723	0.9859257
0.542046244	0.02084793		
0.542052857			

Standard Error	t Stat	P-value	Lower 95%	Upper 95%	Lower 95.0%	Upper 95.0%
0.091932389	41.5523612	2.6307E-25	3.63103809	4.008977551	3.63103809	4.00897755
0.002940318	0.01781089	0.9859257	-0.0059915	0.00609628	-0.0059915	0.00609628

SUMMARY OUTPUT ctrl total [Ca]DCT

Regression Statistics	
Multiple R	0.29958615
R Square	0.08975186
Adjusted R Sq	0.05334194
Standard Erro	0.2998176
Observations	27

ANOVA

	df	SS	MS	F
Regression	1	0.221583754	0.22158375	2.46503836
Residual	25	2.247264765	0.08989059	
Total	26	2.468848519		

	Coefficients	Standard Error	t Stat	P-value
Intercept	7.4925697	0.501508881	14.9400539	5.7413E-14
X Variable 1	-0.0090715	0.005777844	-1.5700441	0.12897659

Significance F
0.12897659

Lower 95%	*Upper 95%*	*Lower 95.0%*	*Upper 95.0%*
6.45969282	8.52544657	6.45969282	8.52544657
-0.0209712	0.00282822	-0.0209712	0.00282822

code	eGFR	P ckd	P ctrl	Ca ckd	Ca ctrl
CKD2	21	1.82		3.778	
CKD4	23	1.852		3.85	
CKD5	34	1.218		3.778	
CKD6	41	1.549		3.85	
CKD7	19	2.036		3.778	
CKD11	33	0.8235		3.778	
CKD13	22	2.375		3.707	
CKD14	14	1.973		3.778	
CKD15	22	2.803		3.564	
CKD18	36	1.003		3.85	
CKD20	20	2.391		4.063	
CKD21	28	2.718		3.778	
CKD23	35	1.624		3.778	
CKD24	20	2.935		3.85	
CKD25	44	1.507		3.992	
CKD26	18	3.251		3.992	
CKD27	28	2.414		3.85	
CKD31	23	2.402		4.206	
CKD32	29	1.489		3.707	
CKD33	28	2.371		3.707	
CKD45	29	2.874		3.992	
CKD46	42	1.331		3.636	
CKD49	42	1.242		3.707	
CKD50	49	0.7575		3.85	
CKD51	34	2.103		3.85	
CKD55	28	0.7141		3.636	
CKD59	47	0.6192		3.992	
CKD62	27	2.702		3.707	
N2	89		1.258		6.986
N3	101		1.075		6.612
N4	93		1.127		6.737
N6	103		0.5899		6.612
N7	94		0.8665		6.737
N8	79		1.751		6.487
N9	77		0.9826		6.612
N10	73		1.394		6.737
N11	108		1.392		6.363
N13	87		0.6849		6.363
N14	93		0.7527		6.862
N15	96		0.9714		6.737
N16	96		1.539		6.986
N17	73		0.8413		7.236
N18	90		1.776		6.737
N20	75		1.206		6.363
N21	75		0.7058		6.249
N24	75		1.062		7.111
N25	89		1.35		6.249

N27	74	1.305	7.361
N29	85	0.6108	6.737
N31	72	0.974	7.111
N32	89	1.335	6.612
N33	93	1.729	6.249
N35	78	0.4466	6.612
N36	84	0.7627	7.111
N38	87	0.5282	6.612

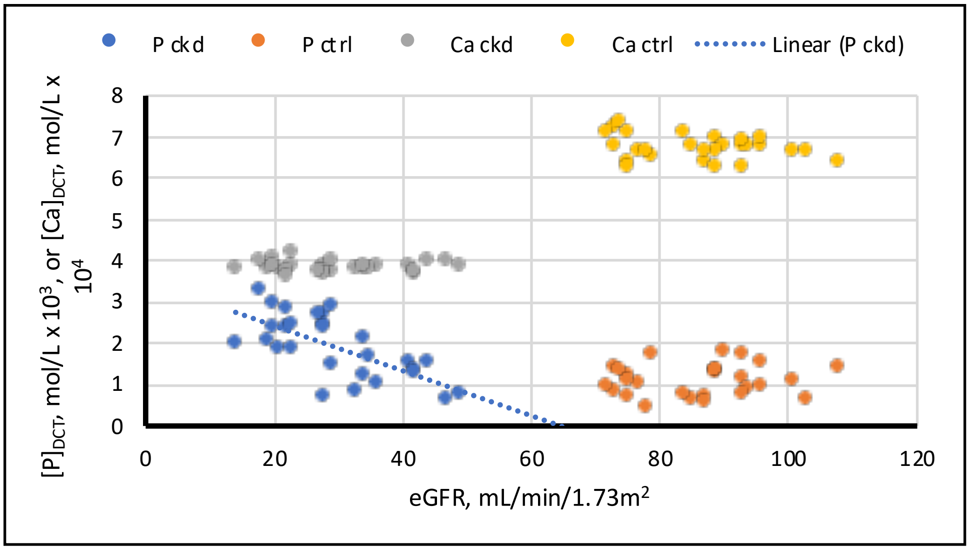

P ckd
P ctrl
Ca ckd
Ca ctrl
Linear (P ckd)
[P]DCT, mol/L x 10^3, or [Ca]DCT, mol/L x 10^4
eGFR, mL/min/1.73m^2
0
20
40
60
80
100
120
0
1
2
3
4
5
6
7
8

code	Tot(Ca)	Ca++	tot Ca x 10^4	ckd	ctrl
CKD2	0.0003778	0.0002962	3.778	2.962	
CKD4	0.000385	0.0003017	3.85	3.017	
CKD5	0.0003778	0.0003028	3.778	3.028	
CKD6	0.000385	0.000305	3.85	3.05	
CKD7	0.0003778	0.0002939	3.778	2.939	
CKD11	0.0003778	0.0003072	3.778	3.072	
CKD13	0.0003707	0.0002846	3.707	2.846	
CKD14	0.0003778	0.0002946	3.778	2.946	
CKD15	0.0003564	0.0002691	3.564	2.691	
CKD18	0.000385	0.0003112	3.85	3.112	
CKD20	0.0004063	0.0003129	4.063	3.129	
CKD21	0.0003778	0.0002867	3.778	2.867	
CKD23	0.0003778	0.0002984	3.778	2.984	
CKD24	0.000385	0.00029	3.85	2.9	
CKD25	0.0003992	0.0003173	3.992	3.173	
CKD26	0.0003992	0.0002977	3.992	2.977	
CKD27	0.000385	0.0002956	3.85	2.956	
CKD31	0.0004206	0.0003242	4.206	3.242	
CKD32	0.0003707	0.000294	3.707	2.94	
CKD33	0.0003707	0.0002847	3.707	2.847	
CKD45	0.0003992	0.0003018	3.992	3.018	
CKD46	0.0003636	0.0002898	3.636	2.898	
CKD49	0.0003707	0.0002966	3.707	2.966	
CKD50	0.000385	0.000314	3.85	3.14	
CKD51	0.000385	0.0002989	3.85	2.989	
CKD55	0.0003636	0.0002963	3.636	2.963	
CKD59	0.0003992	0.0003277	3.992	3.277	
CKD62	0.0003707	0.0002813	3.707	2.813	
N2	0.0006986	0.0005731	6.986		5.731
N3	0.0006612	0.000545	6.612		5.45
N4	0.0006737	0.0005545	6.737		5.545
N6	0.0006612	0.0005549	6.612		5.549
N7	0.0006737	0.0005599	6.737		5.599
N8	0.0006487	0.0004909	6.487		4.909
N9	0.0006612	0.0005468	6.612		5.468
N10	0.0006737	0.0005489	6.737		5.489
N11	0.0006363	0.0005173	6.363		5.173
N13	0.0006363	0.0005312	6.363		5.312
N14	0.0006862	0.0005732	6.862		5.732
N15	0.0006737	0.0005577	6.737		5.577
N16	0.0006986	0.0005361	6.986		5.361
N17	0.0007236	0.0006038	7.236		6.038
N18	0.0006737	0.0004895	6.737		4.895
N20	0.0006363	0.000521	6.363		5.21
N21	0.0006249	0.0005209	6.249		5.209
N24	0.0007111	0.000588	7.111		5.88
N25	0.0006249	0.0005085	6.249		5.085

N27	0.0007361	0.0005947	7.361		5.947
N29	0.0006737	0.0005654	6.737		5.654
N31	0.0007111	0.0005899	7.111		5.899
N32	0.0006612	0.0005395	6.612		5.395
N33	0.0006249	0.0004918	6.249		4.918
N35	0.0006612	0.0005578	6.612		5.578
N36	0.0007111	0.0005946	7.111		5.946
N38	0.0006612	0.0005562	6.612		5.562
CKD16	0.0004277	0.0003373	4.277	3.373	
CKD41	0.0003065	0.0002357	3.065	2.357	

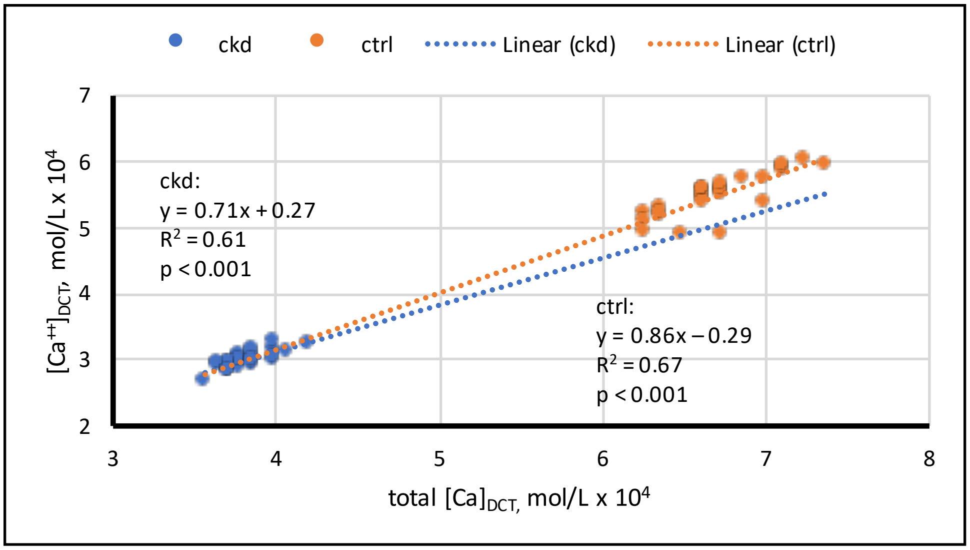

SUMMARY OUTPUT ckd

Regression Statistics	
Multiple R	0.7790662
R Square	0.60694415
Adjusted R Sq	0.59182661
Standard Erro	0.08279307
Observations	28

ANOVA

	df	*SS*	*MS*	*F*	*Significance F*
Regression	1	0.27520469	0.27520469	40.14836	1.04E-06
Residual	26	0.17822202	0.00685469		
Total	27	0.45342671			

	Coefficients	*Standard Error*	*t Stat*	*P-value*	*Lower 95%*
Intercept	0.2677767	0.43003403	0.62268724	0.53891371	-0.6161709
X Variable 1	0.71253647	0.11245356	6.33627335	1.04E-06	0.48138487

SUMMARY OUTPUT ctrl

Regression Statistics	
Multiple R	0.81995693
R Square	0.67232937
Adjusted R Sq	0.65922254
Standard Erro	0.17988543
Observations	27

ANOVA

	df	*SS*	*MS*	*F*	*Significance F*
Regression	1	1.65987936	1.65987936	51.2961262	1.6606E-07
Residual	25	0.80896916	0.03235877		
Total	26	2.46884852			

	Coefficients	*Standard Error*	*t Stat*	*P-value*	*Lower 95%*
Intercept	2.4239245	0.59949304	4.04329045	0.00044328	1.18924547
X Variable 1	0.78140745	0.10910264	7.1621314	1.6606E-07	0.55670635

Upper 95%	*Lower 95.0%*	*Upper 95.0%*
1.15172431	-0.6161709	1.15172431
0.94368806	0.48138487	0.94368806

Upper 95%	*Lower 95.0%*	*Upper 95.0%*
3.65860354	1.18924547	3.65860354
1.00610854	0.55670635	1.00610854

code	Tot(P)	Ca++		tot P x 10^3	ckd	ctrl
CKD2	0.00182	0.0002962		1.82	2.962	
CKD4	0.001852	0.0003017		1.852	3.017	
CKD5	0.001218	0.0003028		1.218	3.028	
CKD6	0.001549	0.000305		1.549	3.05	
CKD7	0.002036	0.0002939		2.036	2.939	
CKD11	0.0008235	0.0003072		0.8235	3.072	
CKD13	0.002375	0.0002846		2.375	2.846	
CKD14	0.001973	0.0002946		1.973	2.946	
CKD15	0.002803	0.0002691		2.803	2.691	
CKD18	0.001003	0.0003112		1.003	3.112	
CKD20	0.002391	0.0003129		2.391	3.129	
CKD21	0.002718	0.0002867		2.718	2.867	
CKD23	0.001624	0.0002984		1.624	2.984	
CKD24	0.002935	0.00029		2.935	2.9	
CKD25	0.001507	0.0003173		1.507	3.173	
CKD26	0.003251	0.0002977		3.251	2.977	
CKD27	0.002414	0.0002956		2.414	2.956	
CKD31	0.002402	0.0003242		2.402	3.242	
CKD32	0.001489	0.000294		1.489	2.94	
CKD33	0.002371	0.0002847		2.371	2.847	
CKD45	0.002874	0.0003018		2.874	3.018	
CKD46	0.001331	0.0002898		1.331	2.898	
CKD49	0.001242	0.0002966		1.242	2.966	
CKD50	0.0007575	0.000314		0.7575	3.14	
CKD51	0.002103	0.0002989		2.103	2.989	
CKD55	0.0007141	0.0002963		0.7141	2.963	
CKD59	0.0006192	0.0003277		0.6192	3.277	
CKD62	0.002702	0.0002813		2.702	2.813	
N2	0.001258	0.0005731		1.258		5.731
N3	0.001075	0.000545		1.075		5.45
N4	0.001127	0.0005545		1.127		5.545
N6	0.0005899	0.0005549		0.5899		5.549
N7	0.0008665	0.0005599		0.8665		5.599
N8	0.001751	0.0004909		1.751		4.909
N9	0.0009826	0.0005468		0.9826		5.468
N10	0.001394	0.0005489		1.394		5.489
N11	0.001392	0.0005173		1.392		5.173
N13	0.0006849	0.0005312		0.6849		5.312
N14	0.0007527	0.0005732		0.7527		5.732
N15	0.0009714	0.0005577		0.9714		5.577
N16	0.001539	0.0005361		1.539		5.361
N17	0.0008413	0.0006038		0.8413		6.038
N18	0.001776	0.0004895		1.776		4.895
N20	0.001206	0.000521		1.206		5.21
N21	0.0007058	0.0005209		0.7058		5.209
N24	0.001062	0.000588		1.062		5.88
N25	0.00135	0.0005085		1.35		5.085

N27	0.001305	0.0005947	1.305	5.947
N29	0.0006108	0.0005654	0.6108	5.654
N31	0.000974	0.0005899	0.974	5.899
N32	0.001335	0.0005395	1.335	5.395
N33	0.001729	0.0004918	1.729	4.918
N35	0.0004466	0.0005578	0.4466	5.578
N36	0.0007627	0.0005946	0.7627	5.946
N38	0.0005282	0.0005562	0.5282	5.562

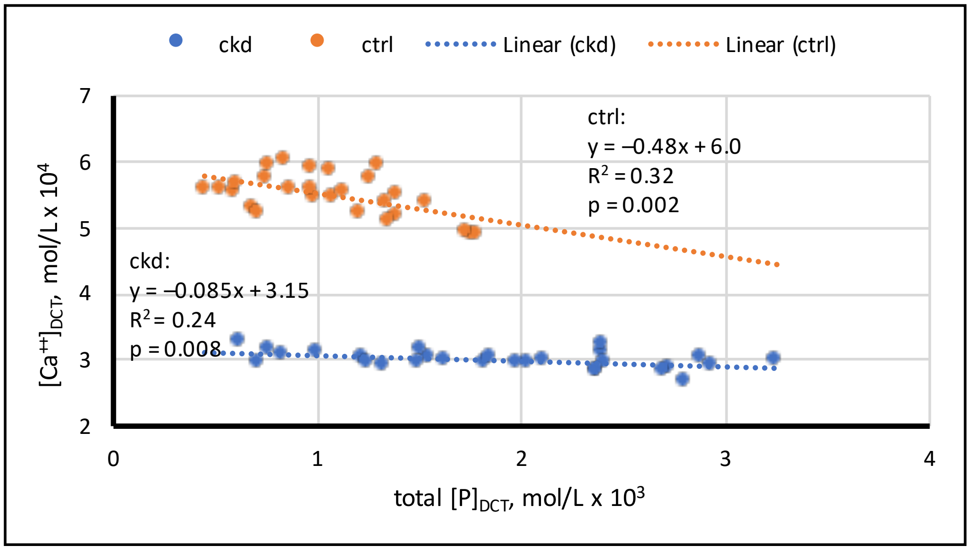

SUMMARY OUTPUT ckd

Regression Statistics	
Multiple R	0.49223743
R Square	0.24229769
Adjusted R Sq	0.21315529
Standard Erro	0.11495191
Observations	28

ANOVA

	df	*SS*	*MS*	*F*	*Significance F*
Regression	1	0.10986425	0.10986425	8.31426779	0.00779529
Residual	26	0.34356247	0.01321394		
Total	27	0.45342671			

	Coefficients	*tandard Error*	*t Stat*	*P-value*	*Lower 95%*
Intercept	3.15083041	0.05960446	52.8623288	5.4039E-28	3.02831169
X Variable 1	-0.0847161	0.02938014	-2.8834472	0.00779529	-0.1451078

SUMMARY OUTPUT ctrl

Regression Statistics	
Multiple R	0.56465334
R Square	0.3188334
Adjusted R Sq	0.29158674
Standard Erro	0.27215567
Observations	27

ANOVA

	df	*SS*	*MS*	*F*	*Significance F*
Regression	1	0.86673282	0.86673282	11.7017408	0.00215239
Residual	25	1.8517177	0.07406871		
Total	26	2.71845052			

	Coefficients	*Standard Error*	*t Stat*	*P-value*	*Lower 95%*
Intercept	5.99847891	0.15881766	37.769596	1.424E-23	5.67138781
X Variable 1	-0.477245	0.13951346	-3.4207807	0.00215239	-0.7645783

Upper 95%	*Lower 95.0%*	*Upper 95.0%*
3.27334912	3.02831169	3.27334912
-0.0243243	-0.1451078	-0.0243243

Upper 95%	*Lower 95.0%*	*Upper 95.0%*
6.32557	5.67138781	6.32557
-0.1899116	-0.7645783	-0.1899116

code	Tot(Ca)	CaHPO4	tot ca x 10^4	ckd	ctrl
CKD2	0.0003778	2.00E-05	3.778	2.001	
CKD4	0.000385	2.07E-05	3.85	2.072	
CKD5	0.0003778	1.38E-05	3.778	1.375	
CKD6	0.000385	1.76E-05	3.85	1.758	
CKD7	0.0003778	2.22E-05	3.778	2.217	
CKD11	0.0003778	9.46E-06	3.778	0.9459	
CKD13	0.0003707	2.50E-05	3.707	2.5	
CKD14	0.0003778	2.15E-05	3.778	2.153	
CKD15	0.0003564	2.78E-05	3.564	2.781	
CKD18	0.000385	1.17E-05	3.85	1.165	
CKD20	0.0004063	2.76E-05	4.063	2.76	
CKD21	0.0003778	2.87E-05	3.778	2.873	
CKD23	0.0003778	1.80E-05	3.778	1.801	
CKD24	0.000385	3.13E-05	3.85	3.131	
CKD25	0.0003992	1.78E-05	3.992	1.776	
CKD26	0.0003992	3.55E-05	3.992	3.549	
CKD27	0.000385	2.63E-05	3.85	2.633	
CKD31	0.0004206	2.87E-05	4.206	2.869	
CKD32	0.0003707	1.63E-05	3.707	1.628	
CKD33	0.0003707	2.50E-05	3.707	2.495	
CKD45	0.0003992	3.19E-05	3.992	3.189	
CKD46	0.0003636	1.44E-05	3.636	1.436	
CKD49	0.0003707	1.37E-05	3.707	1.373	
CKD50	0.000385	8.89E-06	3.85	0.8891	
CKD51	0.000385	2.33E-05	3.85	2.328	
CKD55	0.0003636	7.92E-06	3.636	0.7924	
CKD59	0.0003992	7.60E-06	3.992	0.7595	
CKD62	0.0003707	2.80E-05	3.707	2.802	
N2	0.0006986	2.64E-05	6.986		2.641
N3	0.0006612	2.15E-05	6.612		2.154
N4	0.0006737	2.30E-05	6.737		2.297
N6	0.0006612	1.21E-05	6.612		1.208
N7	0.0006737	1.79E-05	6.737		1.787
N8	0.0006487	3.11E-05	6.487		3.109
N9	0.0006612	1.98E-05	6.612		1.977
N10	0.0006737	2.81E-05	6.737		2.808
N11	0.0006363	2.65E-05	6.363		2.645
N13	0.0006363	1.34E-05	6.363		1.344
N14	0.0006862	1.59E-05	6.862		1.589
N15	0.0006737	1.99E-05	6.737		1.994
N16	0.0006986	2.98E-05	6.986		2.975
N17	0.0007236	1.86E-05	7.236		1.864
N18	0.0006737	3.11E-05	6.737		3.114
N20	0.0006363	2.31E-05	6.363		2.31
N21	0.0006249	1.36E-05	6.249		1.359
N24	0.0007111	2.29E-05	7.111		2.293
N25	0.0006249	2.53E-05	6.249		2.525

N27	0.0007361	2.83E-05	7.361	2.825
N29	0.0006737	1.27E-05	6.737	1.273
N31	0.0007111	2.11E-05	7.111	2.111
N32	0.0006612	2.65E-05	6.612	2.646
N33	0.0006249	3.11E-05	6.249	3.106
N35	0.0006612	9.20E-06	6.612	0.9204
N36	0.0007111	1.67E-05	7.111	1.669
N38	0.0006612	1.09E-05	6.612	1.085

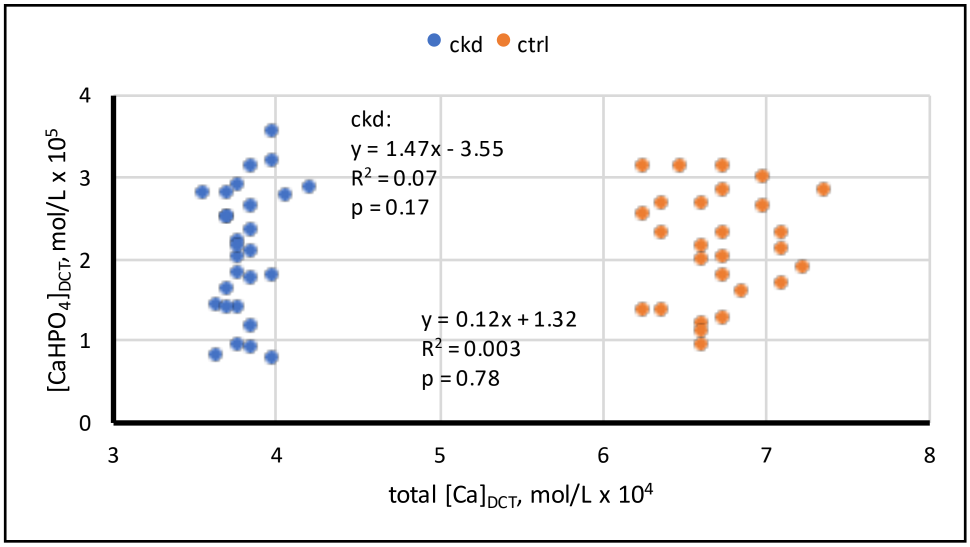

SUMMARY OUTPUT ckd

Regression Statistics	
Multiple R	0.26436717
R Square	0.06989
Adjusted R Sq	0.03411654
Standard Erro	0.77517156
Observations	28

ANOVA

	df	*SS*	*MS*	*F*	*Significance F*
Regression	1	1.17395034	1.17395034	1.95368285	0.17400337
Residual	26	15.6231647	0.60089095		
Total	27	16.797115			

	Coefficients	*Standard Error*	*t Stat*	*P-value*	*Lower 95%*
Intercept	-3.5507271	4.02630476	-0.8818823	0.385921	-11.826915
X Variable 1	1.47164834	1.05287548	1.39774205	0.17400337	-0.6925682

SUMMARY OUTPUT ctrl

Regression Statistics	
Multiple R	0.05605395
R Square	0.00314204
Adjusted R Sq	-0.0367323
Standard Erro	0.68184206
Observations	27

ANOVA

	df	*SS*	*MS*	*F*	*Significance F*

Regression	1	0.0366342	0.0366342	0.07879871	0.78124203
Residual	25	11.6227148	0.46490859		
Total	26	11.659349			

	Coefficients	*Standard Error*	*t Stat*	*P-value*	*Lower 95%*
Intercept	1.31696566	2.91491539	0.45180236	0.65530683	-4.686415
X Variable 1	0.1218137	0.43394687	0.28071108	0.78124203	-0.7719166

Upper 95%	*Lower 95.0%*	*Upper 95.0%*
4.72546087	-11.826915	4.72546087
3.63586488	-0.6925682	3.63586488

Upper 95%	*Lower 95.0%*	*Upper 95.0%*
7.32034627	-4.686415	7.32034627
1.01554401	-0.7719166	1.01554401

code	Tot(P)	CaHPO4	Tot P x 10^3	ckd	ctrl
CKD2	0.00182	2.00E-05	1.82	2.001	
CKD4	0.001852	2.07E-05	1.852	2.072	
CKD5	0.001218	1.38E-05	1.218	1.375	
CKD6	0.001549	1.76E-05	1.549	1.758	
CKD7	0.002036	2.22E-05	2.036	2.217	
CKD11	0.0008235	9.46E-06	0.8235	0.9459	
CKD13	0.002375	2.50E-05	2.375	2.5	
CKD14	0.001973	2.15E-05	1.973	2.153	
CKD15	0.002803	2.78E-05	2.803	2.781	
CKD18	0.001003	1.17E-05	1.003	1.165	
CKD20	0.002391	2.76E-05	2.391	2.76	
CKD21	0.002718	2.87E-05	2.718	2.873	
CKD23	0.001624	1.80E-05	1.624	1.801	
CKD24	0.002935	3.13E-05	2.935	3.131	
CKD25	0.001507	1.78E-05	1.507	1.776	
CKD26	0.003251	3.55E-05	3.251	3.549	
CKD27	0.002414	2.63E-05	2.414	2.633	
CKD31	0.002402	2.87E-05	2.402	2.869	
CKD32	0.001489	1.63E-05	1.489	1.628	
CKD33	0.002371	2.50E-05	2.371	2.495	
CKD45	0.002874	3.19E-05	2.874	3.189	
CKD46	0.001331	1.44E-05	1.331	1.436	
CKD49	0.001242	1.37E-05	1.242	1.373	
CKD50	0.0007575	8.89E-06	0.7575	0.8891	
CKD51	0.002103	2.33E-05	2.103	2.328	
CKD55	0.0007141	7.92E-06	0.7141	0.7924	
CKD59	0.0006192	7.60E-06	0.6192	0.7595	
CKD62	0.002702	2.80E-05	2.702	2.802	
N2	0.001258	2.64E-05	1.258		2.641
N3	0.001075	2.15E-05	1.075		2.154
N4	0.001127	2.30E-05	1.127		2.297
N6	0.0005899	1.21E-05	0.5899		1.208
N7	0.0008665	1.79E-05	0.8665		1.787
N8	0.001751	3.11E-05	1.751		3.109
N9	0.0009826	1.98E-05	0.9826		1.977
N10	0.001394	2.81E-05	1.394		2.808
N11	0.001392	2.65E-05	1.392		2.645
N13	0.0006849	1.34E-05	0.6849		1.344
N14	0.0007527	1.59E-05	0.7527		1.589
N15	0.0009714	1.99E-05	0.9714		1.994
N16	0.001539	2.98E-05	1.539		2.975
N17	0.0008413	1.86E-05	0.8413		1.864
N18	0.001776	3.11E-05	1.776		3.114
N20	0.001206	2.31E-05	1.206		2.31
N21	0.0007058	1.36E-05	0.7058		1.359
N24	0.001062	2.29E-05	1.062		2.293
N25	0.00135	2.53E-05	1.35		2.525

N27	0.001305	2.83E-05	1.305	2.825
N29	0.0006108	1.27E-05	0.6108	1.273
N31	0.000974	2.11E-05	0.974	2.111
N32	0.001335	2.65E-05	1.335	2.646
N33	0.001729	3.11E-05	1.729	3.106
N35	0.0004466	9.20E-06	0.4466	0.9204
N36	0.0007627	1.67E-05	0.7627	1.669
N38	0.0005282	1.09E-05	0.5282	1.085

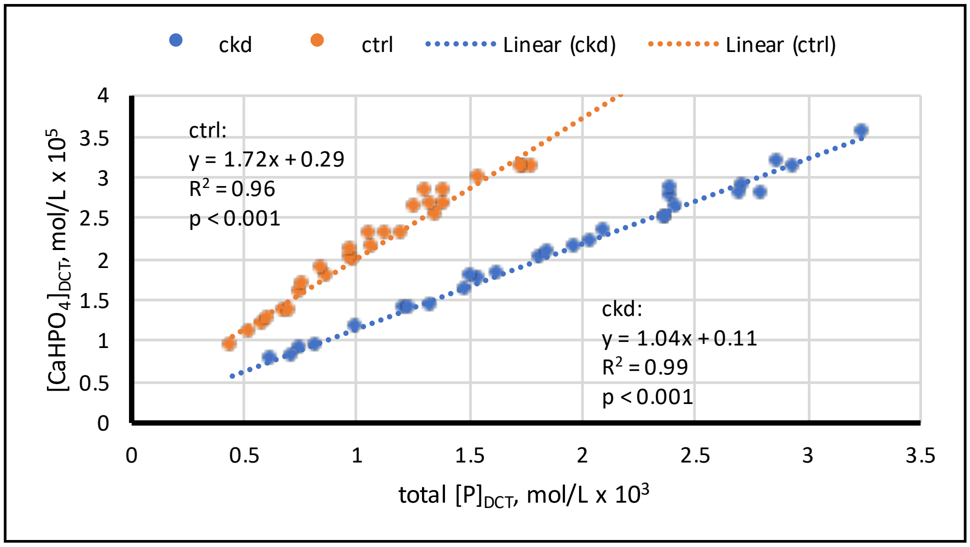

SUMMARY OUTPUT ckd

Regression Statistics	
Multiple R	0.99340422
R Square	0.98685195
Adjusted R Sq	0.98634626
Standard Erro	0.09216401
Observations	28

ANOVA

	df	*SS*	*MS*	*F*	*Significance F*
Regression	1	16.5762657	16.5762657	1951.47961	5.472E-26
Residual	26	0.2208493	0.0084942		
Total	27	16.797115			

	Coefficients	*Standard Error*	*t Stat*	*P-value*	*Lower 95%*
Intercept	0.1074047	0.04778855	2.24749837	0.03331506	0.00917392
X Variable 1	1.04059316	0.02355586	44.1755545	5.472E-26	0.9921734

SUMMARY OUTPUT ctrl

Regression Statistics	
Multiple R	0.98151734
R Square	0.9633763
Adjusted R Sq	0.96191135
Standard Erro	0.13069178
Observations	27

ANOVA

	df	*SS*	*MS*	*F*	*Significance F*
Regression	1	11.2323404	11.2323404	657.618014	1.7912E-19
Residual	25	0.42700854	0.01708034		
Total	26	11.659349			

	Coefficients	*Standard Error*	*t Stat*	*P-value*	*Lower 95%*
Intercept	0.28803694	0.07626578	3.77675225	0.000877	0.13096463
X Variable 1	1.7180423	0.06699571	25.6440639	1.7912E-19	1.58006205

Upper 95%	*Lower 95.0%*	*Upper 95.0%*
0.20563548	0.00917392	0.20563548
1.08901292	0.9921734	1.08901292

Upper 95%	*Lower 95.0%*	*Upper 95.0%*
0.44510924	0.13096463	0.44510924
1.85602255	1.58006205	1.85602255

code	CaHPO4	Ca++	CaHPO4 x 10^5	ckd	ctrl
CKD2	2.00E-05	0.0002962	2.001	2.962	
CKD4	2.07E-05	0.0003017	2.072	3.017	
CKD5	1.38E-05	0.0003028	1.375	3.028	
CKD6	1.76E-05	0.000305	1.758	3.05	
CKD7	2.22E-05	0.0002939	2.217	2.939	
CKD11	9.46E-06	0.0003072	0.9459	3.072	
CKD13	2.50E-05	0.0002846	2.5	2.846	
CKD14	2.15E-05	0.0002946	2.153	2.946	
CKD15	2.78E-05	0.0002691	2.781	2.691	
CKD18	1.17E-05	0.0003112	1.165	3.112	
CKD20	2.76E-05	0.0003129	2.76	3.129	
CKD21	2.87E-05	0.0002867	2.873	2.867	
CKD23	1.80E-05	0.0002984	1.801	2.984	
CKD24	3.13E-05	0.00029	3.131	2.9	
CKD25	1.78E-05	0.0003173	1.776	3.173	
CKD26	3.55E-05	0.0002977	3.549	2.977	
CKD27	2.63E-05	0.0002956	2.633	2.956	
CKD31	2.87E-05	0.0003242	2.869	3.242	
CKD32	1.63E-05	0.000294	1.628	2.94	
CKD33	2.50E-05	0.0002847	2.495	2.847	
CKD45	3.19E-05	0.0003018	3.189	3.018	
CKD46	1.44E-05	0.0002898	1.436	2.898	
CKD49	1.37E-05	0.0002966	1.373	2.966	
CKD50	8.89E-06	0.000314	0.8891	3.14	
CKD51	2.33E-05	0.0002989	2.328	2.989	
CKD55	7.92E-06	0.0002963	0.7924	2.963	
CKD59	7.60E-06	0.0003277	0.7595	3.277	
CKD62	2.80E-05	0.0002813	2.802	2.813	
N2	2.64E-05	0.0005731	2.641		5.731
N3	2.15E-05	0.000545	2.154		5.45
N4	2.30E-05	0.0005545	2.297		5.545
N6	1.21E-05	0.0005549	1.208		5.549
N7	1.79E-05	0.0005599	1.787		5.599
N8	3.11E-05	0.0004909	3.109		4.909
N9	1.98E-05	0.0005468	1.977		5.468
N10	2.81E-05	0.0005489	2.808		5.489
N11	2.65E-05	0.0005173	2.645		5.173
N13	1.34E-05	0.0005312	1.344		5.312
N14	1.59E-05	0.0005732	1.589		5.732
N15	1.99E-05	0.0005577	1.994		5.577
N16	2.98E-05	0.0005361	2.975		5.361
N17	1.86E-05	0.0006038	1.864		6.038
N18	3.11E-05	0.0004895	3.114		4.895
N20	2.31E-05	0.000521	2.31		5.21
N21	1.36E-05	0.0005209	1.359		5.209
N24	2.29E-05	0.000588	2.293		5.88
N25	2.53E-05	0.0005085	2.525		5.085

N27	2.83E-05	0.0005947	2.825	5.947
N29	1.27E-05	0.0005654	1.273	5.654
N31	2.11E-05	0.0005899	2.111	5.899
N32	2.65E-05	0.0005395	2.646	5.395
N33	3.11E-05	0.0004918	3.106	4.918
N35	9.20E-06	0.0005578	0.9204	5.578
N36	1.67E-05	0.0005946	1.669	5.946
N38	1.09E-05	0.0005562	1.085	5.562

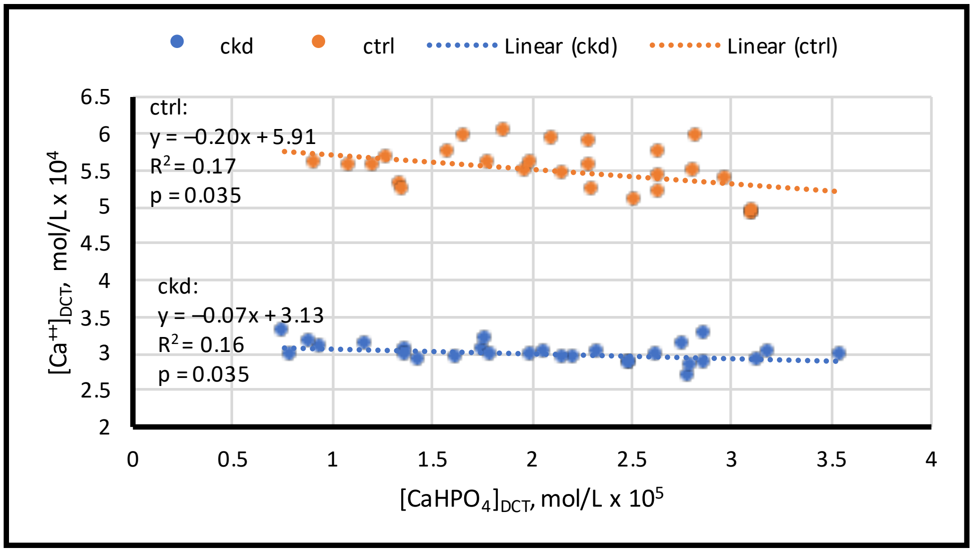

SUMMARY OUTPUT ckd

Regression Statistics	
Multiple R	0.39854254
R Square	0.15883616
Adjusted R Sq	0.1264837
Standard Erro	0.12111758
Observations	28

ANOVA

	df	*SS*	*MS*	*F*	*Significance F*
Regression	1	0.07202056	0.07202056	4.9095549	0.03567031
Residual	26	0.38140616	0.01466947		
Total	27	0.45342671			

	Coefficients	*Standard Error*	*t Stat*	*P-value*	*Lower 95%*
Intercept	3.12654492	0.06540588	47.8022025	7.2059E-27	2.99210122
X Variable 1	-0.0654803	0.0295522	-2.2157515	0.03567031	-0.1262258

SUMMARY OUTPUT ctrl

Regression Statistics	
Multiple R	0.40718624
R Square	0.16580063
Adjusted R Sq	0.13243266
Standard Erro	0.30117966

Observations	27

ANOVA

	df	*SS*	*MS*	*F*	*Significance F*
Regression	1	0.45072082	0.45072082	4.96885522	0.03502981
Residual	25	2.2677297	0.09070919		
Total	26	2.71845052			

	Coefficients	*Standard Error*	*t Stat*	*P-value*	*Lower 95%*
Intercept	5.90524475	0.19698212	29.9785829	4.066E-21	5.49955248
X Variable 1	-0.196615	0.08820404	-2.2290929	0.03502981	-0.3782746

Upper 95%	*Lower 95.0%*	*Upper 95.0%*
3.26098863	2.99210122	3.26098863
-0.0047349	-0.1262258	-0.0047349

Upper 95%	*Lower 95.0%*	*Upper 95.0%*
6.31093701	5.49955248	6.31093701
-0.0149554	-0.3782746	-0.0149554

code	CaCitric	Ca++		Cacit x 10^5	ckd	ctrl
CKD2	3.09E-05	0.0002962		3.088	2.962	
CKD4	3.12E-05	0.0003017		3.119	3.017	
CKD5	3.12E-05	0.0003028		3.118	3.028	
CKD6	3.14E-05	0.000305		3.136	3.05	
CKD7	3.08E-05	0.0002939		3.076	2.939	
CKD11	3.14E-05	0.0003072		3.137	3.072	
CKD13	3.03E-05	0.0002846		3.026	2.846	
CKD14	3.08E-05	0.0002946		3.078	2.946	
CKD15	2.93E-05	0.0002691		2.933	2.691	
CKD18	3.16E-05	0.0003112		3.162	3.112	
CKD20	3.19E-05	0.0003129		3.188	3.129	
CKD21	3.04E-05	0.0002867		3.041	2.867	
CKD23	3.10E-05	0.0002984		3.096	2.984	
CKD24	3.06E-05	0.00029		3.062	2.9	
CKD25	3.20E-05	0.0003173		3.201	3.173	
CKD26	3.11E-05	0.0002977		3.109	2.977	
CKD27	3.09E-05	0.0002956		3.088	2.956	
CKD31	3.25E-05	0.0003242		3.247	3.242	
CKD32	3.07E-05	0.000294		3.069	2.94	
CKD33	3.03E-05	0.0002847		3.025	2.847	
CKD45	3.13E-05	0.0003018		3.129	3.018	
CKD46	3.04E-05	0.0002898		3.042	2.898	
CKD49	3.08E-05	0.0002966		3.082	2.966	
CKD50	3.17E-05	0.000314		3.173	3.14	
CKD51	3.11E-05	0.0002989		3.107	2.989	
CKD55	3.07E-05	0.0002963		3.073	2.963	
CKD59	3.25E-05	0.0003277		3.247	3.277	
CKD62	3.01E-05	0.0002813		3.007	2.813	
N2	4.22E-05	0.0005731		4.219		5.731
N3	4.13E-05	0.000545		4.132		5.45
N4	4.16E-05	0.0005545		4.163		5.545
N6	4.16E-05	0.0005549		4.158		5.549
N7	4.18E-05	0.0005599		4.178		5.599
N8	3.96E-05	0.0004909		3.96		4.909
N9	4.14E-05	0.0005468		4.138		5.468
N10	4.15E-05	0.0005489		4.15		5.489
N11	4.05E-05	0.0005173		4.048		5.173
N13	4.09E-05	0.0005312		4.086		5.312
N14	4.22E-05	0.0005732		4.216		5.732
N15	4.17E-05	0.0005577		4.173		5.577
N16	4.11E-05	0.0005361		4.109		5.361
N17	4.30E-05	0.0006038		4.302		6.038
N18	3.96E-05	0.0004895		3.955		4.895
N20	4.06E-05	0.000521		4.056		5.21
N21	4.05E-05	0.0005209		4.053		5.209
N24	4.26E-05	0.000588		4.263		5.88
N25	4.02E-05	0.0005085		4.019		5.085

N27	4.28E-05	0.0005947	4.282	5.947
N29	4.19E-05	0.0005654	4.191	5.654
N31	4.27E-05	0.0005899	4.268	5.899
N32	4.12E-05	0.0005395	4.12	5.395
N33	3.96E-05	0.0004918	3.964	4.918
N35	4.17E-05	0.0005578	4.166	5.578
N36	4.28E-05	0.0005946	4.279	5.946
N38	4.16E-05	0.0005562	4.162	5.562

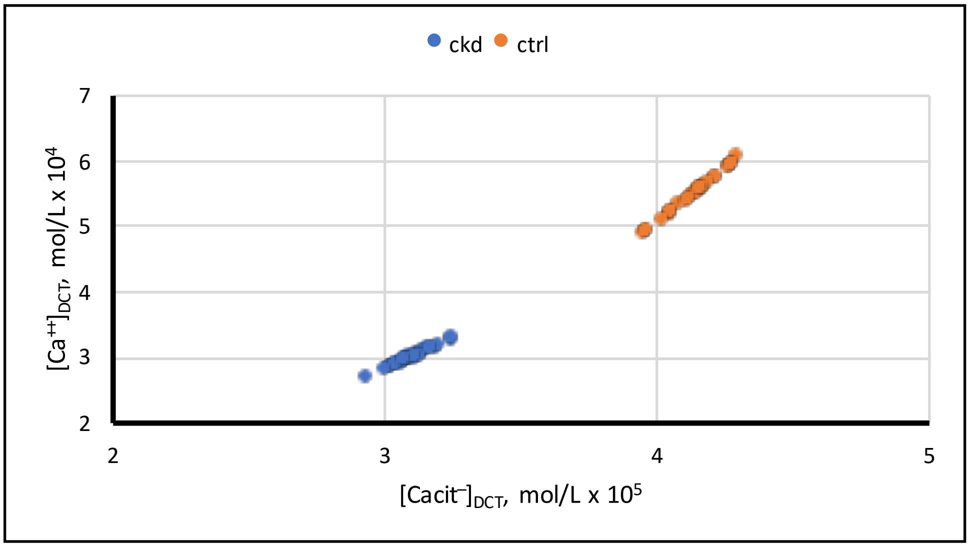

ckd
ctrl
7
6
5
4
3
2
2
3
4
5
[Ca++]DCT, mol/L x 10^4
[Cacit⁻]DCT, mol/L x 10^5

code	Tot(Ca)	tot Ca x 10^4	ckd	ctrl
CKD2	0.0003778	3.778	158	
CKD4	0.000385	3.85	41	
CKD5	0.0003778	3.778	59	
CKD6	0.000385	3.85	54	
CKD7	0.0003778	3.778	129	
CKD11	0.0003778	3.778	50	
CKD13	0.0003707	3.707	56	
CKD14	0.0003778	3.778	145	
CKD15	0.0003564	3.564	156	
CKD18	0.000385	3.85	67	
CKD20	0.0004063	4.063	182	
CKD21	0.0003778	3.778	126	
CKD23	0.0003778	3.778	63	
CKD24	0.000385	3.85	103	
CKD25	0.0003992	3.992	42	
CKD26	0.0003992	3.992	69	
CKD27	0.000385	3.85	72	
CKD31	0.0004206	4.206	31	
CKD32	0.0003707	3.707	91	
CKD33	0.0003707	3.707	54	
CKD45	0.0003992	3.992	127	
CKD46	0.0003636	3.636	39	
CKD49	0.0003707	3.707	48	
CKD50	0.000385	3.85	48	
CKD51	0.000385	3.85	73	
CKD55	0.0003636	3.636	32	
CKD59	0.0003992	3.992	28	
CKD62	0.0003707	3.707	178	
N2	0.0006986	6.986		21
N3	0.0006612	6.612		44
N4	0.0006737	6.737		45
N6	0.0006612	6.612		31
N7	0.0006737	6.737		18
N8	0.0006487	6.487		24
N9	0.0006612	6.612		36
N10	0.0006737	6.737		22
N11	0.0006363	6.363		60
N13	0.0006363	6.363		28
N14	0.0006862	6.862		20
N15	0.0006737	6.737		34
N16	0.0006986	6.986		17
N17	0.0007236	7.236		29
N18	0.0006737	6.737		25
N20	0.0006363	6.363		19
N21	0.0006249	6.249		26
N24	0.0007111	7.111		21
N25	0.0006249	6.249		41

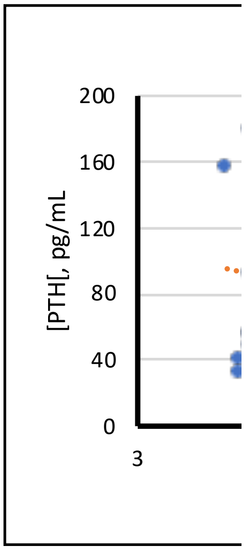

SUMMARY OU

Regression

Multiple R
R Square
Adjusted R Sq
Standard Erro
Observations

ANOVA

Regression
Residual
Total

Intercept
X Variable 1

SUMMARY OU

Regression

Multiple R
R Square
Adjusted R Sq
Standard Erro
Observations

N27	0.0007361	7.361	16	ANOVA
N29	0.0006737	6.737	23	
N31	0.0007111	7.111	19	Regression
N32	0.0006612	6.612	24	Residual
N33	0.0006249	6.249	65	Total
N35	0.0006612	6.612	24	
N36	0.0007111	7.111	25	
N38	0.0006612	6.612	26	Intercept
				X Variable 1

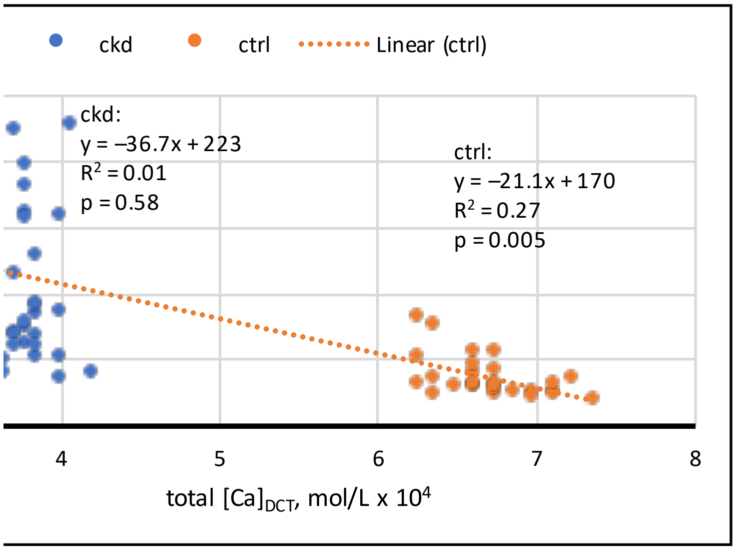

TPUT ckd

Statistics
0.10902978
0.01188749
-0.0261168
48.2660858
28

df	*SS*	*MS*	*F*	*Significance F*
1	728.687668	728.687668	0.31279317	0.58075742
26	60569.9909	2329.61503		
27	61298.6786			

Coefficients	*Standard Error*	*t Stat*	*P-value*	*Lower 95%*	*Upper 95%*	*Lower 95.0%*
223.010193	250.698014	0.88955708	0.38186086	-292.30695	738.327342	-292.30695
-36.664848	65.5573306	-0.5592792	0.58075742	-171.41987	98.0901747	-171.41987

TPUT ctrl

Statistics
0.52245748
0.27296181
0.24388029
10.8043163
27

df	*SS*	*MS*	*F*	*Significance F*
1	1095.66872	1095.66872	9.38608932	0.00517895
25	2918.33128	116.733251		
26	4014			

Coefficients	*Standard Error*	*t Stat*	*P-value*	*Lower 95%*	*Upper 95%*	*Lower 95.0%*
170.364805	46.1890955	3.68842046	0.00109768	75.2365821	265.493028	75.2365821
-21.066501	6.87622484	-3.0636725	0.00517895	-35.228351	-6.9046509	-35.228351

Upper 95.0%
738.327342
98.0901747

Upper 95.0%
265.493028
-6.9046509

code	Ca+2	Ca++ x 10^4	ckd	ctrl
CKD2	0.0002962	2.962	158	
CKD4	0.0003017	3.017	41	
CKD5	0.0003028	3.028	59	
CKD6	0.000305	3.05	54	
CKD7	0.0002939	2.939	129	
CKD11	0.0003072	3.072	50	
CKD13	0.0002846	2.846	56	
CKD14	0.0002946	2.946	145	
CKD15	0.0002691	2.691	156	
CKD18	0.0003112	3.112	67	
CKD20	0.0003129	3.129	182	
CKD21	0.0002867	2.867	126	
CKD23	0.0002984	2.984	63	
CKD24	0.00029	2.9	103	
CKD25	0.0003173	3.173	42	
CKD26	0.0002977	2.977	69	
CKD27	0.0002956	2.956	72	
CKD31	0.0003242	3.242	31	
CKD32	0.000294	2.94	91	
CKD33	0.0002847	2.847	54	
CKD45	0.0003018	3.018	127	
CKD46	0.0002898	2.898	39	
CKD49	0.0002966	2.966	48	
CKD50	0.000314	3.14	48	
CKD51	0.0002989	2.989	73	
CKD55	0.0002963	2.963	32	
CKD59	0.0003277	3.277	28	
CKD62	0.0002813	2.813	178	
N2	0.0005731	5.731		21
N3	0.000545	5.45		44
N4	0.0005545	5.545		45
N6	0.0005549	5.549		31
N7	0.0005599	5.599		18
N8	0.0004909	4.909		24
N9	0.0005468	5.468		36
N10	0.0005489	5.489		22
N11	0.0005173	5.173		60
N13	0.0005312	5.312		28
N14	0.0005732	5.732		20
N15	0.0005577	5.577		34
N16	0.0005361	5.361		17
N17	0.0006038	6.038		29
N18	0.0004895	4.895		25
N20	0.000521	5.21		19
N21	0.0005209	5.209		26
N24	0.000588	5.88		21
N25	0.0005085	5.085		41

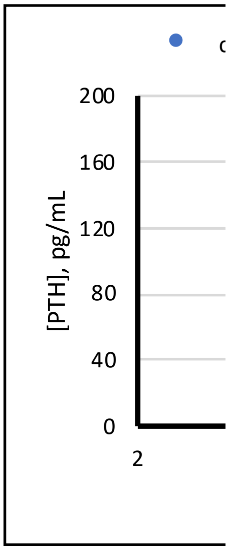

SUMMARY OU

Regression
Multiple R
R Square
Adjusted R Sq
Standard Erro
Observations

ANOVA

Regression
Residual
Total

Intercept
X Variable 1

SUMMARY OU

Regression
Multiple R
R Square
Adjusted R Sq
Standard Erro
Observations

N27	0.0005947	5.947	16	ANOVA
N29	0.0005654	5.654	23	
N31	0.0005899	5.899	19	Regression
N32	0.0005395	5.395	24	Residual
N33	0.0004918	4.918	65	Total
N35	0.0005578	5.578	24	
N36	0.0005946	5.946	25	
N38	0.0005562	5.562	26	Intercept
				X Variable 1

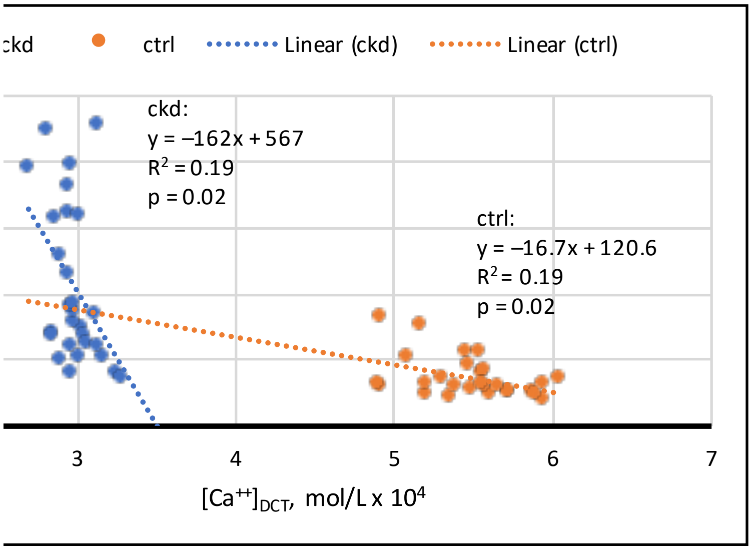

TPUT ckd

Statistics
0.44032567
0.19388669
0.16288234
43.5950246
28

df	*SS*	*MS*	*F*	*Significance F*
1	11884.9982	11884.9982	6.25353039	0.01902705
26	49413.6804	1900.52617		
27	61298.6786			

Coefficients	*Standard Error*	*t Stat*	*P-value*	*Lower 95%*	*Upper 95%*	*Lower 95.0%*
567.100126	193.803423	2.92616155	0.00703559	168.731484	965.468769	168.731484
-161.89969	64.7415921	-2.500706	0.01902705	-294.97793	-28.821438	-294.97793

TPUT ctrl

Statistics
0.43446357
0.1887586
0.15630894
11.4128401
27

df	*SS*	*MS*	*F*	*Significance F*
1	757.677008	757.677008	5.81696756	0.0235424
25	3256.32299	130.25292		
26	4014			

Coefficients	*Standard Error*	*t Stat*	*P-value*	*Lower 95%*	*Upper 95%*	*Lower 95.0%*
120.580896	38.0348671	3.17027257	0.00399639	42.2466208	198.915171	42.2466208
-16.694805	6.92202267	-2.411839	0.0235424	-30.950977	-2.438632	-30.950977

Upper 95.0%
965.468769
-28.821438

Upper 95.0%
198.915171
-2.438632

code	Ca+2	Ca++ x 10^4	10/Ca++	ckd and ctrl
CKD2	0.0002962	2.962	3.37609723	158
CKD4	0.0003017	3.017	3.31455088	41
CKD5	0.0003028	3.028	3.30250991	59
CKD6	0.000305	3.05	3.27868852	54
CKD7	0.0002939	2.939	3.40251786	129
CKD11	0.0003072	3.072	3.25520833	50
CKD13	0.0002846	2.846	3.51370344	56
CKD14	0.0002946	2.946	3.39443313	145
CKD15	0.0002691	2.691	3.71609067	156
CKD18	0.0003112	3.112	3.21336761	67
CKD20	0.0003129	3.129	3.19590924	182
CKD21	0.0002867	2.867	3.48796652	126
CKD23	0.0002984	2.984	3.35120643	63
CKD24	0.00029	2.9	3.44827586	103
CKD25	0.0003173	3.173	3.15159155	42
CKD26	0.0002977	2.977	3.35908633	69
CKD27	0.0002956	2.956	3.38294993	72
CKD31	0.0003242	3.242	3.08451573	31
CKD32	0.000294	2.94	3.40136054	91
CKD33	0.0002847	2.847	3.51246927	54
CKD45	0.0003018	3.018	3.31345262	127
CKD46	0.0002898	2.898	3.45065562	39
CKD49	0.0002966	2.966	3.37154417	48
CKD50	0.000314	3.14	3.18471338	48
CKD51	0.0002989	2.989	3.34560054	73
CKD55	0.0002963	2.963	3.37495781	32
CKD59	0.0003277	3.277	3.05157156	28
CKD62	0.0002813	2.813	3.55492357	178
N2	0.0005731	5.731	1.74489618	21
N3	0.000545	5.45	1.83486239	44
N4	0.0005545	5.545	1.80342651	45
N6	0.0005549	5.549	1.80212651	31
N7	0.0005599	5.599	1.78603322	18
N8	0.0004909	4.909	2.03707476	24
N9	0.0005468	5.468	1.82882224	36
N10	0.0005489	5.489	1.82182547	22
N11	0.0005173	5.173	1.93311425	60
N13	0.0005312	5.312	1.88253012	28
N14	0.0005732	5.732	1.74459177	20
N15	0.0005577	5.577	1.79307872	34
N16	0.0005361	5.361	1.86532363	17
N17	0.0006038	6.038	1.65617754	29
N18	0.0004895	4.895	2.04290092	25
N20	0.000521	5.21	1.9193858	19
N21	0.0005209	5.209	1.91975427	26
N24	0.000588	5.88	1.70068027	21
N25	0.0005085	5.085	1.96656834	41

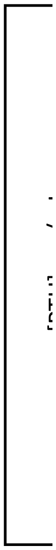

N27	0.0005947	5.947	1.68152009	16
N29	0.0005654	5.654	1.76865936	23
N31	0.0005899	5.899	1.69520258	19
N32	0.0005395	5.395	1.85356812	24
N33	0.0004918	4.918	2.03334689	65
N35	0.0005578	5.578	1.79275726	24
N36	0.0005946	5.946	1.68180289	25
N38	0.0005562	5.562	1.79791442	26

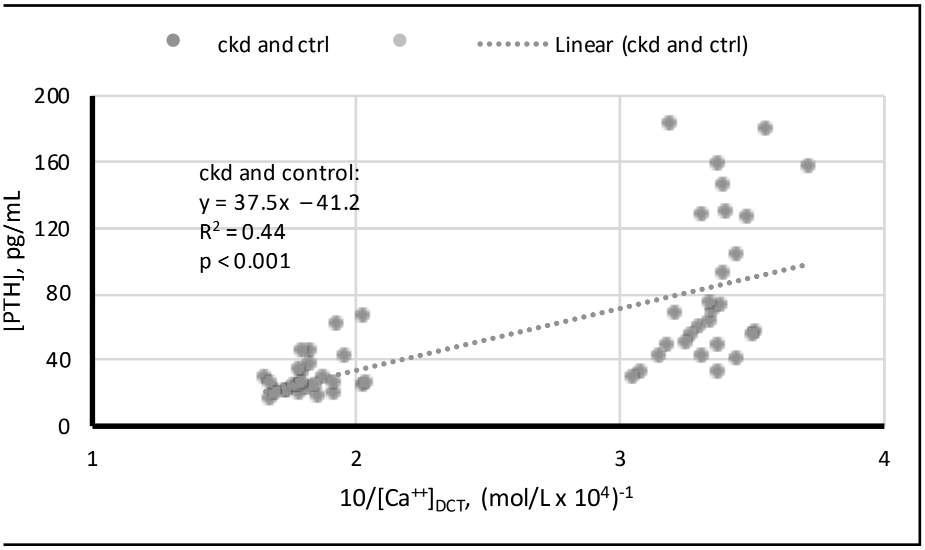

SUMMARY OUTPUT

Regression Statistics	
Multiple R	0.66060513
R Square	0.43639914
Adjusted R Sq	0.42576516
Standard Erro	33.4525384
Observations	55

ANOVA

	df	*SS*	*MS*	*F*	*Significance F*
Regression	1	45924.6939	45924.6939	41.0381821	4.0633E-08
Residual	53	59310.8334	1119.07233		
Total	54	105235.527			

	Coefficients	*Standard Error*	*t Stat*	*P-value*	*Lower 95%*	*Upper 95%*
Intercept	-41.186279	15.8925746	-2.5915423	0.01231718	-73.062747	-9.3098115
X Variable 1	37.5005274	5.85387331	6.40610506	4.0633E-08	25.7591445	49.2419104

Lower 95.0%	*Upper 95.0%*
-73.062747	-9.3098115
25.7591445	49.2419104

code	Ca+2	Ca++ x 10^4	ckd	ctrl	ckd and ctrl
CKD2	0.0002962	2.962	158		85.4036462
CKD4	0.0003017	3.017	41		83.0956579
CKD5	0.0003028	3.028	59		82.6441215
CKD6	0.000305	3.05	54		81.7508197
CKD7	0.0002939	2.939	129		86.3944199
CKD11	0.0003072	3.072	50		80.8703125
CKD13	0.0002846	2.846	56		90.5638791
CKD14	0.0002946	2.946	145		86.0912424
CKD15	0.0002691	2.691	156		98.1534002
CKD18	0.0003112	3.112	67		79.3012853
CKD20	0.0003129	3.129	182		78.6465964
CKD21	0.0002867	2.867	126		89.5987443
CKD23	0.0002984	2.984	63		84.4702413
CKD24	0.00029	2.9	103		88.1103448
CKD25	0.0003173	3.173	42		76.9846833
CKD26	0.0002977	2.977	69		84.7657373
CKD27	0.0002956	2.956	72		85.6606225
CKD31	0.0003242	3.242	31		74.4693399
CKD32	0.000294	2.94	91		86.3510204
CKD33	0.0002847	2.847	54		90.5175975
CKD45	0.0003018	3.018	127		83.0544732
CKD46	0.0002898	2.898	39		88.1995859
CKD49	0.0002966	2.966	48		85.2329063
CKD50	0.000314	3.14	48		78.2267516
CKD51	0.0002989	2.989	73		84.2600201
CKD55	0.0002963	2.963	32		85.360918
CKD59	0.0003277	3.277	28		73.2339335
CKD62	0.0002813	2.813	178		92.1096338
N2	0.0005731	5.731		21	24.2336067
N3	0.000545	5.45		44	27.6073394
N4	0.0005545	5.545		45	26.4284941
N6	0.0005549	5.549		31	26.3797441
N7	0.0005599	5.599		18	25.7762458
N8	0.0004909	4.909		24	35.1903035
N9	0.0005468	5.468		36	27.3808339
N10	0.0005489	5.489		22	27.1184551
N11	0.0005173	5.173		60	31.2917843
N13	0.0005312	5.312		28	29.3948795
N14	0.0005732	5.732		20	24.2221912
N15	0.0005577	5.577		34	26.0404519
N16	0.0005361	5.361		17	28.7496363
N17	0.0006038	6.038		29	20.9066578
N18	0.0004895	4.895		25	35.4087845
N20	0.000521	5.21		19	30.7769674
N21	0.0005209	5.209		26	30.7907852
N24	0.000588	5.88		21	22.5755102
N25	0.0005085	5.085		41	32.5463127

N27	0.0005947	5.947	16	21.8570035
N29	0.0005654	5.654	23	25.1247259
N31	0.0005899	5.899	19	22.3700966
N32	0.0005395	5.395	24	28.3088044
N33	0.0004918	4.918	65	35.0505083
N35	0.0005578	5.578	24	26.0283973
N36	0.0005946	5.946	25	21.8676085
N38	0.0005562	5.562	26	26.2217907

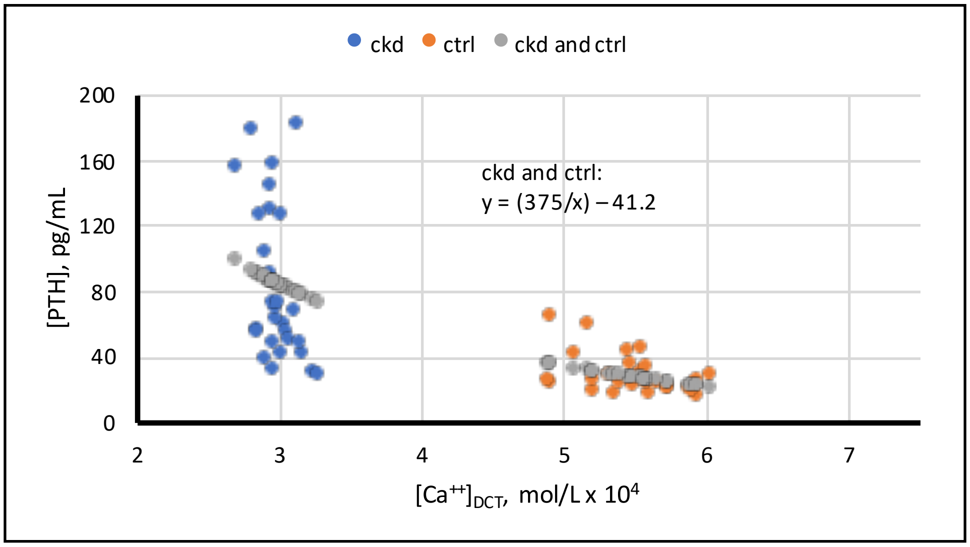

ckd
ctrl
ckd and ctrl
200
160
120
80
40
0
[PTH], pg/mL
2
3
4
5
6
7
[Ca++]DCT, mol/L x 10^4
ckd and ctrl:
y = (375/x) – 41.2

code	Tot(P)	tot P x 10^3	ckd	ctrl
CKD2	0.00182	1.82	158	
CKD4	0.001852	1.852	41	
CKD5	0.001218	1.218	59	
CKD6	0.001549	1.549	54	
CKD7	0.002036	2.036	129	
CKD11	0.0008235	0.8235	50	
CKD13	0.002375	2.375	56	
CKD14	0.001973	1.973	145	
CKD15	0.002803	2.803	156	
CKD18	0.001003	1.003	67	
CKD20	0.002391	2.391	182	
CKD21	0.002718	2.718	126	
CKD23	0.001624	1.624	63	
CKD24	0.002935	2.935	103	
CKD25	0.001507	1.507	42	
CKD26	0.003251	3.251	69	
CKD27	0.002414	2.414	72	
CKD31	0.002402	2.402	31	
CKD32	0.001489	1.489	91	
CKD33	0.002371	2.371	54	
CKD45	0.002874	2.874	127	
CKD46	0.001331	1.331	39	
CKD49	0.001242	1.242	48	
CKD50	0.0007575	0.7575	48	
CKD51	0.002103	2.103	73	
CKD55	0.0007141	0.7141	32	
CKD59	0.0006192	0.6192	28	
CKD62	0.002702	2.702	178	
N2	0.001258	1.258		21
N3	0.001075	1.075		44
N4	0.001127	1.127		45
N6	0.0005899	0.5899		31
N7	0.0008665	0.8665		18
N8	0.001751	1.751		24
N9	0.0009826	0.9826		36
N10	0.001394	1.394		22
N11	0.001392	1.392		60
N13	0.0006849	0.6849		28
N14	0.0007527	0.7527		20
N15	0.0009714	0.9714		34
N16	0.001539	1.539		17
N17	0.0008413	0.8413		29
N18	0.001776	1.776		25
N20	0.001206	1.206		19
N21	0.0007058	0.7058		26
N24	0.001062	1.062		21
N25	0.00135	1.35		41

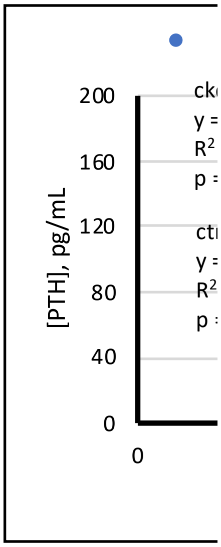

SUMMARY OU

Regression
Multiple R
R Square
Adjusted R Sq
Standard Erro
Observations

ANOVA

Regression
Residual
Total

Intercept
X Variable 1

SUMMARY OU

Regression
Multiple R
R Square
Adjusted R Sq
Standard Erro
Observations

N27	0.001305	1.305	16	ANOVA
N29	0.0006108	0.6108	23	
N31	0.000974	0.974	19	Regression
N32	0.001335	1.335	24	Residual
N33	0.001729	1.729	65	Total
N35	0.0004466	0.4466	24	
N36	0.0007627	0.7627	25	
N38	0.0005282	0.5282	26	Intercept
				X Variable 1

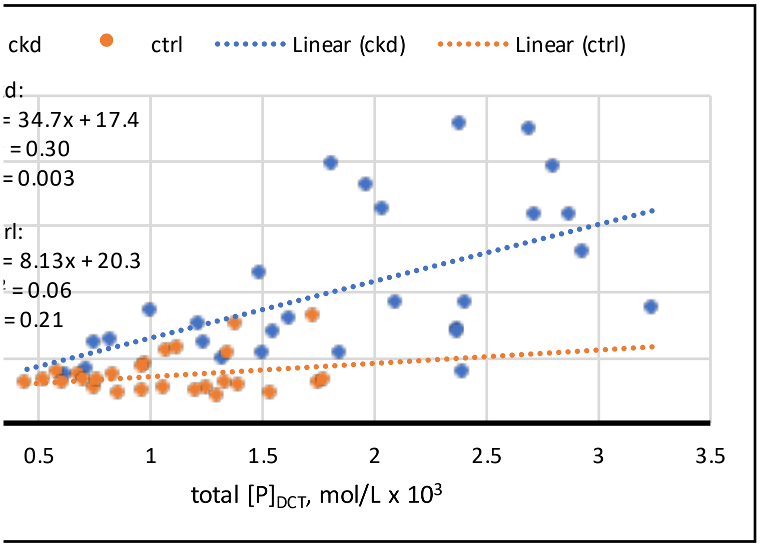

TPUT ckd

Statistics
0.54792622
0.30022314
0.27330865
40.6180126
28

df	*SS*	*MS*	*F*	*Significance F*
1	18403.282	18403.282	11.1547012	0.00254162
26	42895.3966	1649.82295		
27	61298.6786			

Coefficients	*Standard Error*	*t Stat*	*P-value*	*Lower 95%*	*Upper 95%*	*Lower 95.0%*
17.3899266	21.0611084	0.82568905	0.41649288	-25.901802	60.6816549	-25.901802
34.6725079	10.3814086	3.33986544	0.00254162	13.3332169	56.011799	13.3332169

TPUT ctrl

Statistics
0.25035381
0.06267703
0.02518411
12.2677046
27

df	*SS*	*MS*	*F*	*Significance F*
1	251.585607	251.585607	1.6717032	0.20785198
25	3762.41439	150.496576		
26	4014			

Coefficients	*Standard Error*	*t Stat*	*P-value*	*Lower 95%*	*Upper 95%*	*Lower 95.0%*
20.261812	7.15887404	2.83030709	0.00904204	5.51783489	35.0057891	5.51783489
8.13095617	6.28871675	1.29294362	0.20785198	-4.8208984	21.0828108	-4.8208984

Upper 95.0%
60.6816549
56.011799

Upper 95.0%
35.0057891
21.0828108

code	CaHPO4	CaHPO4 x 10^5	ckd	ctrl
CKD2	2.00E-05	2.001	158	
CKD4	2.07E-05	2.072	41	
CKD5	1.38E-05	1.375	59	
CKD6	1.76E-05	1.758	54	
CKD7	2.22E-05	2.217	129	
CKD11	9.46E-06	0.9459	50	
CKD13	2.50E-05	2.5	56	
CKD14	2.15E-05	2.153	145	
CKD15	2.78E-05	2.781	156	
CKD18	1.17E-05	1.165	67	
CKD20	2.76E-05	2.76	182	
CKD21	2.87E-05	2.873	126	
CKD23	1.80E-05	1.801	63	
CKD24	3.13E-05	3.131	103	
CKD25	1.78E-05	1.776	42	
CKD26	3.55E-05	3.549	69	
CKD27	2.63E-05	2.633	72	
CKD31	2.87E-05	2.869	31	
CKD32	1.63E-05	1.628	91	
CKD33	2.50E-05	2.495	54	
CKD45	3.19E-05	3.189	127	
CKD46	1.44E-05	1.436	39	
CKD49	1.37E-05	1.373	48	
CKD50	8.89E-06	0.8891	48	
CKD51	2.33E-05	2.328	73	
CKD55	7.92E-06	0.7924	32	
CKD59	7.60E-06	0.7595	28	
CKD62	2.80E-05	2.802	178	
N2	2.64E-05	2.641		21
N3	2.15E-05	2.154		44
N4	2.30E-05	2.297		45
N6	1.21E-05	1.208		31
N7	1.79E-05	1.787		18
N8	3.11E-05	3.109		24
N9	1.98E-05	1.977		36
N10	2.81E-05	2.808		22
N11	2.65E-05	2.645		60
N13	1.34E-05	1.344		28
N14	1.59E-05	1.589		20
N15	1.99E-05	1.994		34
N16	2.98E-05	2.975		17
N17	1.86E-05	1.864		29
N18	3.11E-05	3.114		25
N20	2.31E-05	2.31		19
N21	1.36E-05	1.359		26
N24	2.29E-05	2.293		21
N25	2.53E-05	2.525		41

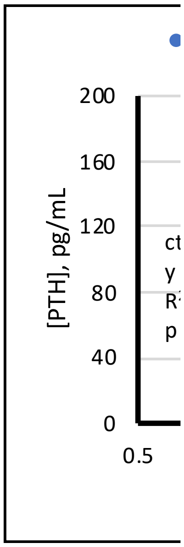

SUMMARY OU

Regression
Multiple R
R Square
Adjusted R Sq
Standard Erro
Observations

ANOVA

Regression
Residual
Total

Intercept
X Variable 1

SUMMARY OU

Regression
Multiple R
R Square
Adjusted R Sq
Standard Erro
Observations

N27	2.83E-05	2.825	16	ANOVA
N29	1.27E-05	1.273	23	
N31	2.11E-05	2.111	19	Regression
N32	2.65E-05	2.646	24	Residual
N33	3.11E-05	3.106	65	Total
N35	9.20E-06	0.9204	24	
N36	1.67E-05	1.669	25	
N38	1.09E-05	1.085	26	Intercept
				X Variable 1

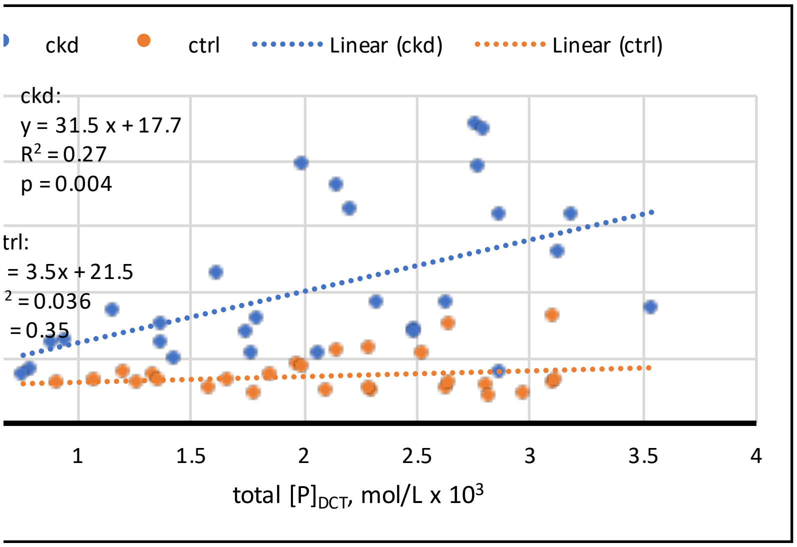

TPUT

Statistics
0.52083115
0.27126508
0.24323682
41.4499176
28

df	*SS*	*MS*	*F*	*Significance F*
1	16628.1912	16628.1912	9.67826852	0.00448785
26	44670.4874	1718.09567		
27	61298.6786			

Coefficients	*Standard Error*	*t Stat*	*P-value*	*Lower 95%*	*Upper 95%*	*Lower 95.0%*
17.660426	22.3837714	0.78898349	0.43725747	-28.350075	63.670927	-28.350075
31.4633642	10.1136128	3.11099156	0.00448785	10.6745353	52.2521931	10.6745353

TPUT ctrl

Statistics
0.18884642
0.03566297
-0.0029105
12.4432292
27

df	SS	MS	F	Significance F
1	143.151159	143.151159	0.9245463	0.34549262
25	3870.84884	154.833954		
26	4014			

Coefficients	Standard Error	t Stat	P-value	Lower 95%	Upper 95%	Lower 95.0%
21.52118	8.13831068	2.64442841	0.01393389	4.76001539	38.2823446	4.76001539
3.50396923	3.64414754	0.96153331	0.34549262	-4.0012931	11.0092316	-4.0012931

Upper 95.0%
63.670927
52.2521931

Upper 95.0%
38.2823446
11.0092316

code	eGFR	ckd	ctrl
CKD2	21	158	
CKD4	23	41	
CKD5	34	59	
CKD6	41	54	
CKD7	19	129	
CKD11	33	50	
CKD13	22	56	
CKD14	14	145	
CKD15	22	156	
CKD18	36	67	
CKD20	20	182	
CKD21	28	126	
CKD23	35	63	
CKD24	20	103	
CKD25	44	42	
CKD26	18	69	
CKD27	28	72	
CKD31	23	31	
CKD32	29	91	
CKD33	28	54	
CKD45	29	127	
CKD46	42	39	
CKD49	42	48	
CKD50	49	48	
CKD51	34	73	
CKD55	28	32	
CKD59	47	28	
CKD62	27	178	
N2	89		21
N3	101		44
N4	93		45
N6	103		31
N7	94		18
N8	79		24
N9	77		36
N10	73		22
N11	108		60
N13	87		28
N14	93		20
N15	96		34
N16	96		17
N17	73		29
N18	90		25
N20	75		19
N21	75		26
N24	75		21
N25	89		41

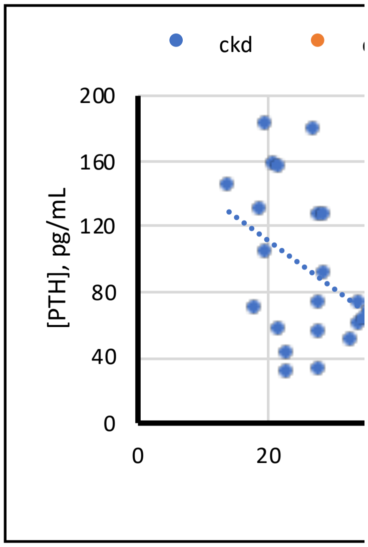

SUMMARY OUTPUT

Regression Statistics	
Multiple R	0.57726753
R Square	0.3332378
Adjusted R Sq	0.3075931
Standard Erro	39.6482816
Observations	28

ANOVA

	df
Regression	1
Residual	26
Total	27

	Coefficients
Intercept	169.791647
X Variable 1	-2.9104858

SUMMARY OUTPUT

Regression Statistics	
Multiple R	0.5186142
R Square	0.26896069
Adjusted R Sq	0.23971911
Standard Erro	10.8340054
Observations	27

N27	74	16
N29	85	23
N31	72	19
N32	89	24
N33	93	65
N35	78	24
N36	84	25
N38	87	26

ANOVA

	df
Regression	1
Residual	25
Total	26

	Coefficients
Intercept	-25.596022
X Variable 1	0.63320129

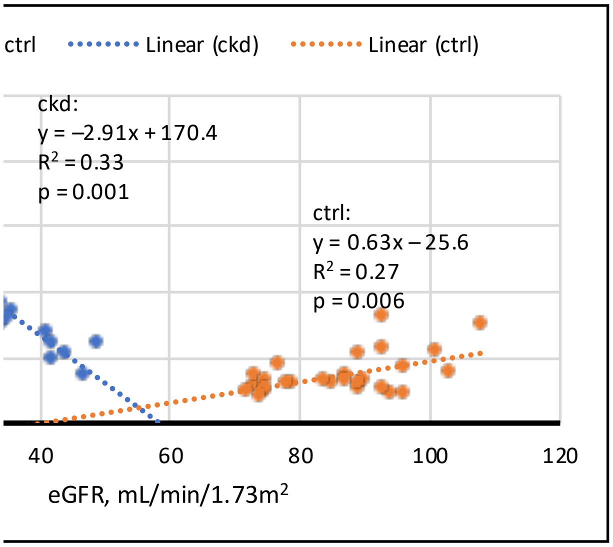

ckd

SS	MS	F	Significance F
20427.0366	20427.0366	12.9944119	0.0012986
40871.642	1571.98623		
61298.6786			

Standard Error	t Stat	P-value	Lower 95%	Upper 95%	Lower 95.0%	Upper 95.0%
25.2441887	6.72596965	3.8878E-07	117.901474	221.68182	117.901474	221.68182
0.80739707	-3.6047763	0.0012986	-4.5701142	-1.2508573	-4.5701142	-1.2508573

ctrl

SS	MS	F	Significance F
1079.6082	1079.6082	9.19788722	0.0055798
2934.3918	117.375672		
4014			

Standard Error	t Stat	P-value	Lower 95%	Upper 95%	Lower 95.0%	Upper 95.0%
18.1221849	-1.4124137	0.17015635	-62.919361	11.7273163	-62.919361	11.7273163
0.20878426	3.03280188	0.0055798	0.20320206	1.06320051	0.20320206	1.06320051

code	eGFR	100/eGFR	ckd and ctrl
CKD2	21	4.76190476	158
CKD4	23	4.34782609	41
CKD5	34	2.94117647	59
CKD6	41	2.43902439	54
CKD7	19	5.26315789	129
CKD11	33	3.03030303	50
CKD13	22	4.54545455	56
CKD14	14	7.14285714	145
CKD15	22	4.54545455	156
CKD18	36	2.77777778	67
CKD20	20	5	182
CKD21	28	3.57142857	126
CKD23	35	2.85714286	63
CKD24	20	5	103
CKD25	44	2.27272727	42
CKD26	18	5.55555556	69
CKD27	28	3.57142857	72
CKD31	23	4.34782609	31
CKD32	29	3.44827586	91
CKD33	28	3.57142857	54
CKD45	29	3.44827586	127
CKD46	42	2.38095238	39
CKD49	42	2.38095238	48
CKD50	49	2.04081633	48
CKD51	34	2.94117647	73
CKD55	28	3.57142857	32
CKD59	47	2.12765957	28
CKD62	27	3.7037037	178
N2	89	1.12359551	21
N3	101	0.99009901	44
N4	93	1.07526882	45
N6	103	0.97087379	31
N7	94	1.06382979	18
N8	79	1.26582278	24
N9	77	1.2987013	36
N10	73	1.36986301	22
N11	108	0.92592593	60
N13	87	1.14942529	28
N14	93	1.07526882	20
N15	96	1.04166667	34
N16	96	1.04166667	17
N17	73	1.36986301	29
N18	90	1.11111111	25
N20	75	1.33333333	19
N21	75	1.33333333	26
N24	75	1.33333333	21
N25	89	1.12359551	41

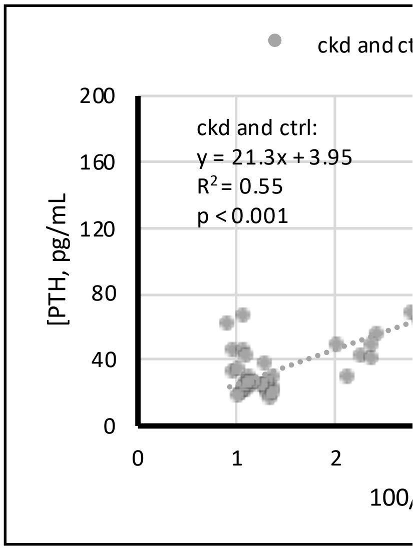

SUMMARY OUTPUT

Regression Statistics	
Multiple R	0.74479267
R Square	0.55471611
Adjusted R Sq	0.54631453
Standard Erro	29.7345757
Observations	55

ANOVA

	df
Regression	1
Residual	53
Total	54

	Coefficients
Intercept	3.94957599
X Variable 1	21.3329675

N27	74	1.35135135	16
N29	85	1.17647059	23
N31	72	1.38888889	19
N32	89	1.12359551	24
N33	93	1.07526882	65
N35	78	1.28205128	24
N36	84	1.19047619	25
N38	87	1.14942529	26

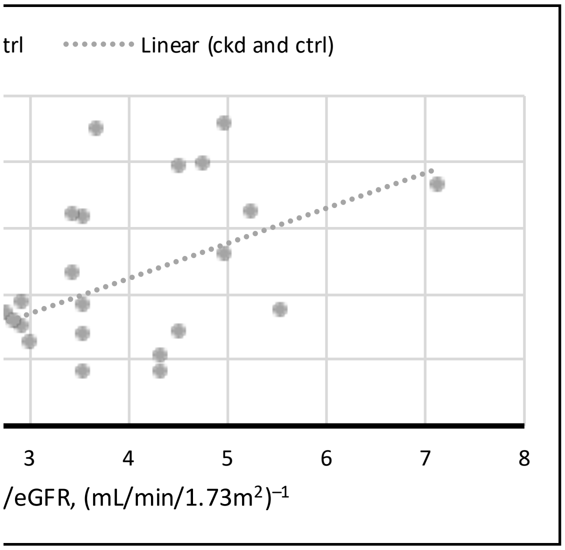

both

SS	MS	F	Significance F
58375.8427	58375.8427	66.0251919	7.0545E-11
46859.6845	884.144991		
105235.527			

Standard Error	t Stat	P-value	Lower 95%	Upper 95%	Lower 95.0%	Upper 95.0%
7.60261692	0.51950217	0.60557351	-11.299342	19.1984944	-11.299342	19.1984944
2.62540577	8.12558871	7.0545E-11	16.0670704	26.5988646	16.0670704	26.5988646

code	eGFR	100/eGFR	ckd	ctrl	ckd and ctrl
CKD2	21	4.76190476	158		105.378571
CKD4	23	4.34782609	41		96.5586957
CKD5	34	2.94117647	59		66.5970588
CKD6	41	2.43902439	54		55.9012195
CKD7	19	5.26315789	129		116.055263
CKD11	33	3.03030303	50		68.4954545
CKD13	22	4.54545455	56		100.768182
CKD14	14	7.14285714	145		156.092857
CKD15	22	4.54545455	156		100.768182
CKD18	36	2.77777778	67		63.1166667
CKD20	20	5	182		110.45
CKD21	28	3.57142857	126		80.0214286
CKD23	35	2.85714286	63		64.8071429
CKD24	20	5	103		110.45
CKD25	44	2.27272727	42		52.3590909
CKD26	18	5.55555556	69		122.283333
CKD27	28	3.57142857	72		80.0214286
CKD31	23	4.34782609	31		96.5586957
CKD32	29	3.44827586	91		77.3982759
CKD33	28	3.57142857	54		80.0214286
CKD45	29	3.44827586	127		77.3982759
CKD46	42	2.38095238	39		54.6642857
CKD49	42	2.38095238	48		54.6642857
CKD50	49	2.04081633	48		47.4193878
CKD51	34	2.94117647	73		66.5970588
CKD55	28	3.57142857	32		80.0214286
CKD59	47	2.12765957	28		49.2691489
CKD62	27	3.7037037	178		82.8388889
N2	89	1.12359551		21	27.8825843
N3	101	0.99009901		44	25.0391089
N4	93	1.07526882		45	26.8532258
N6	103	0.97087379		31	24.6296117
N7	94	1.06382979		18	26.6095745
N8	79	1.26582278		24	30.9120253
N9	77	1.2987013		36	31.6123377
N10	73	1.36986301		22	33.1280822
N11	108	0.92592593		60	23.6722222
N13	87	1.14942529		28	28.4327586
N14	93	1.07526882		20	26.8532258
N15	96	1.04166667		34	26.1375
N16	96	1.04166667		17	26.1375
N17	73	1.36986301		29	33.1280822
N18	90	1.11111111		25	27.6166667
N20	75	1.33333333		19	32.35
N21	75	1.33333333		26	32.35
N24	75	1.33333333		21	32.35
N25	89	1.12359551		41	27.8825843

N27	74	1.35135135	16	32.7337838
N29	85	1.17647059	23	29.0088235
N31	72	1.38888889	19	33.5333333
N32	89	1.12359551	24	27.8825843
N33	93	1.07526882	65	26.8532258
N35	78	1.28205128	24	31.2576923
N36	84	1.19047619	25	29.3071429
N38	87	1.14942529	26	28.4327586

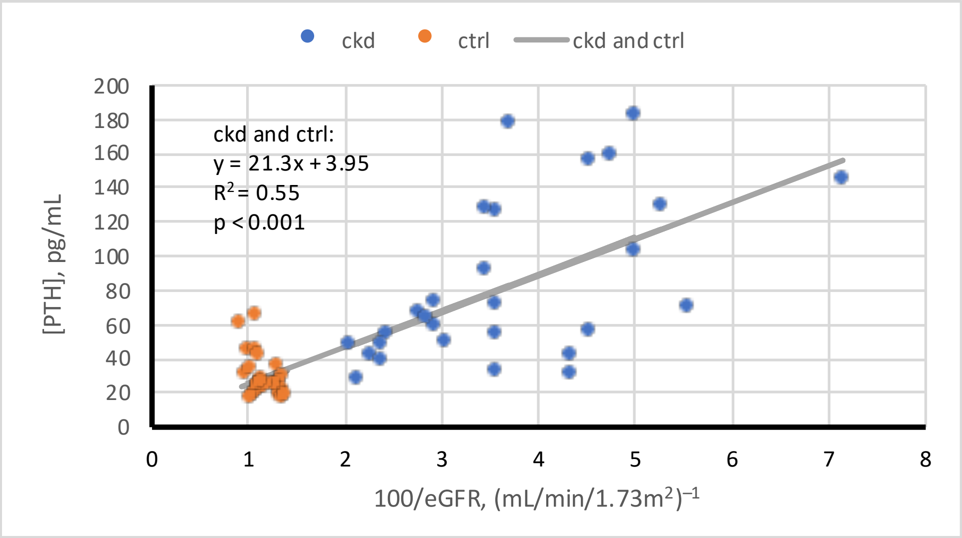

ckd
ctrl
ckd and ctrl
ckd and ctrl:
y = 21.3x + 3.95
R^2 = 0.55
p < 0.001
[PTH], pg/mL
100/eGFR, (mL/min/1.73m^2)$^{-1}$
0
20
40
60
80
100
120
140
160
180
200
0
1
2
3
4
5
6
7
8

code	eGFR	ckd	ctrl	ckd and ctrl
CKD2	21	158		105.378571
CKD4	23	41		96.5586957
CKD5	34	59		66.5970588
CKD6	41	54		55.9012195
CKD7	19	129		116.055263
CKD11	33	50		68.4954545
CKD13	22	56		100.768182
CKD14	14	145		156.092857
CKD15	22	156		100.768182
CKD18	36	67		63.1166667
CKD20	20	182		110.45
CKD21	28	126		80.0214286
CKD23	35	63		64.8071429
CKD24	20	103		110.45
CKD25	44	42		52.3590909
CKD26	18	69		122.283333
CKD27	28	72		80.0214286
CKD31	23	31		96.5586957
CKD32	29	91		77.3982759
CKD33	28	54		80.0214286
CKD45	29	127		77.3982759
CKD46	42	39		54.6642857
CKD49	42	48		54.6642857
CKD50	49	48		47.4193878
CKD51	34	73		66.5970588
CKD55	28	32		80.0214286
CKD59	47	28		49.2691489
CKD62	27	178		82.8388889
N2	89		21	27.8825843
N3	101		44	25.0391089
N4	93		45	26.8532258
N6	103		31	24.6296117
N7	94		18	26.6095745
N8	79		24	30.9120253
N9	77		36	31.6123377
N10	73		22	33.1280822
N11	108		60	23.6722222
N13	87		28	28.4327586
N14	93		20	26.8532258
N15	96		34	26.1375
N16	96		17	26.1375
N17	73		29	33.1280822
N18	90		25	27.6166667
N20	75		19	32.35
N21	75		26	32.35
N24	75		21	32.35
N25	89		41	27.8825843

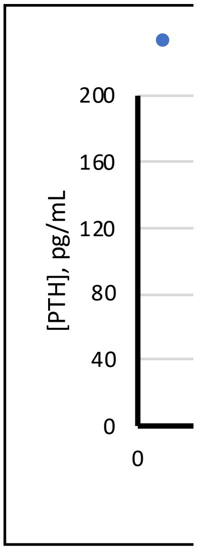

N27	74	16	32.7337838
N29	85	23	29.0088235
N31	72	19	33.5333333
N32	89	24	27.8825843
N33	93	65	26.8532258
N35	78	24	31.2576923
N36	84	25	29.3071429
N38	87	26	28.4327586

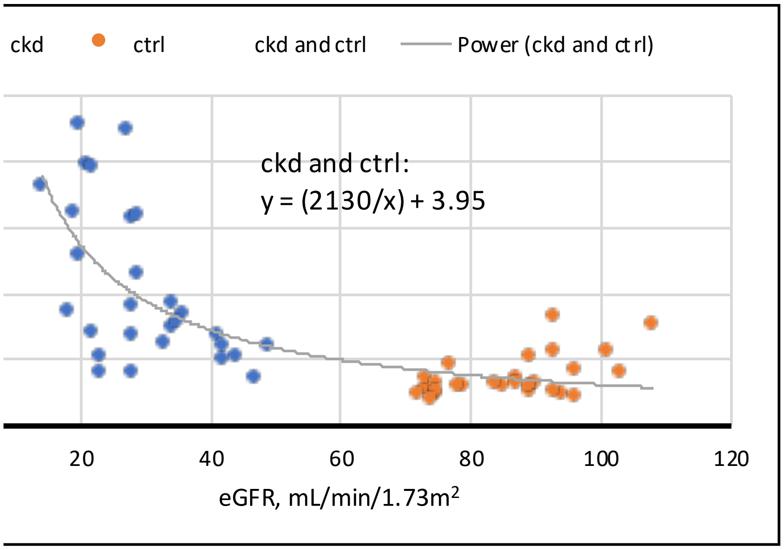

ckd
ctrl
ckd and ctrl
Power (ckd and ctrl)
ckd and ctrl:
y = (2130/x) + 3.95
20
40
60
80
100
120
eGFR, mL/min/1.73m2

Figure 1. Regressions unaffected by pH or precipitation

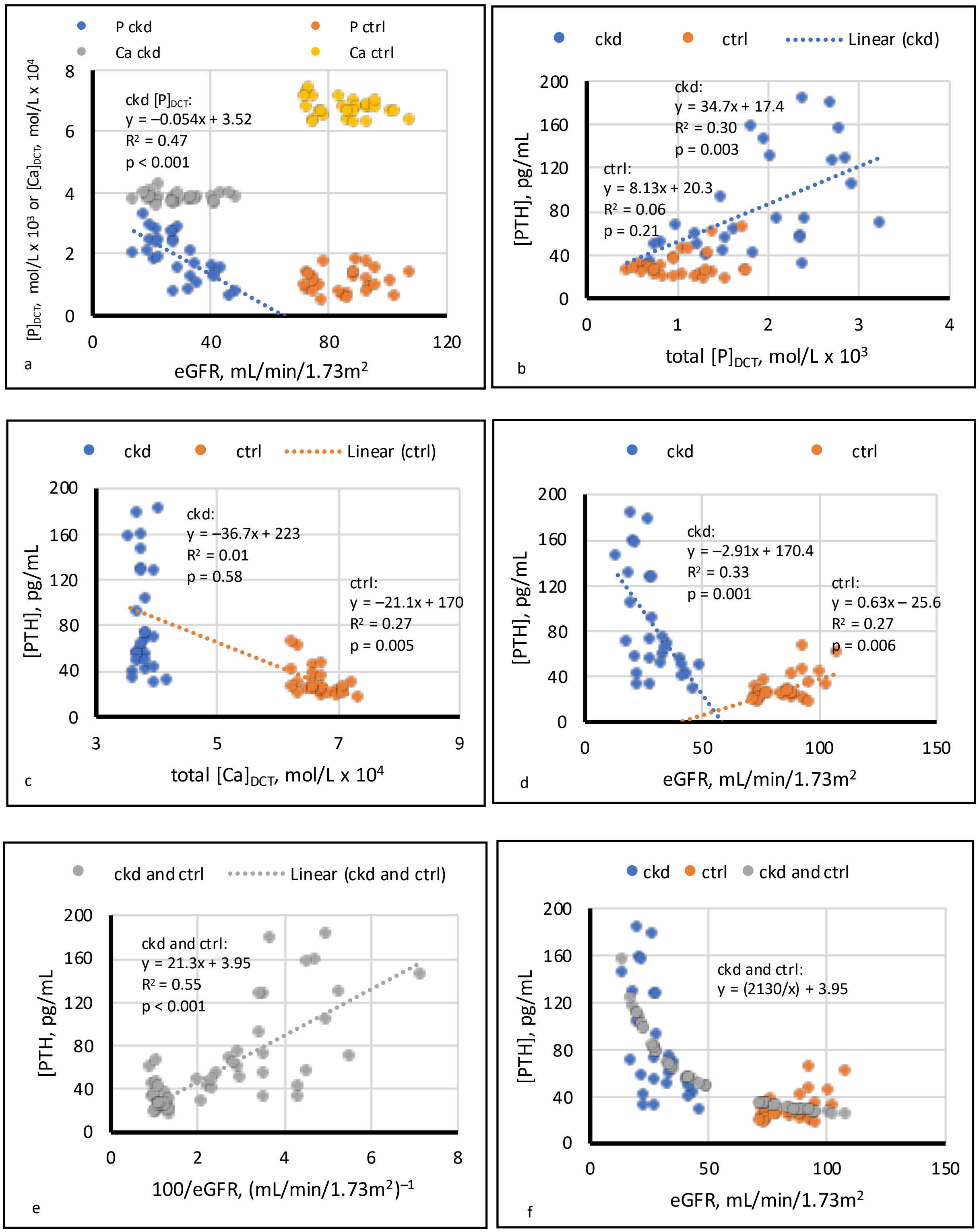

Figure 1. Regressions unaffected by pH or precipitation
