# Supplementary material for: Chemical evidence for the tradeoff-in-the-nephron hypothesis to explain secondary hyperparathyroidism
Source: PLoS One. 2022 Aug 1;17(8):e0272380. doi: 10.1371/journal.pone.0272380 (PMC9342777; doi:10.1371/journal.pone.0272380)
Supplement: S4 File — (PDF) [file pone.0272380.s013.pdf]

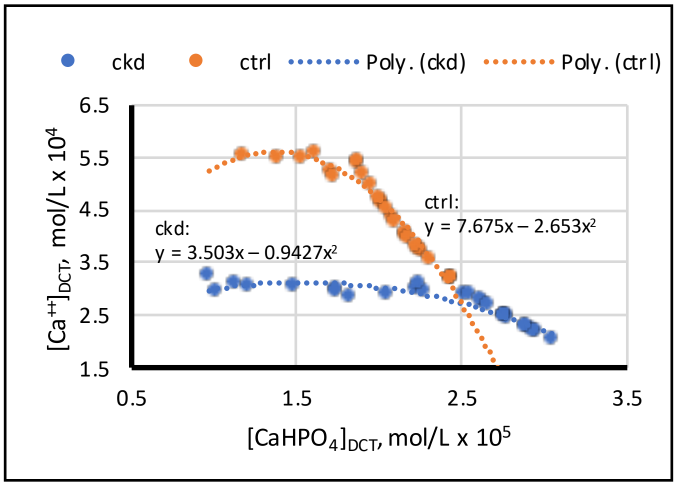

ckd
ctrl
Poly. (ckd)
Poly. (ctrl)
ckd:
y = 3.503x – 0.9427x²
ctrl:
y = 7.675x – 2.653x²
[Ca++]DCT, mol/L x 10^4
[CaHPO4]DCT, mol/L x 10^5

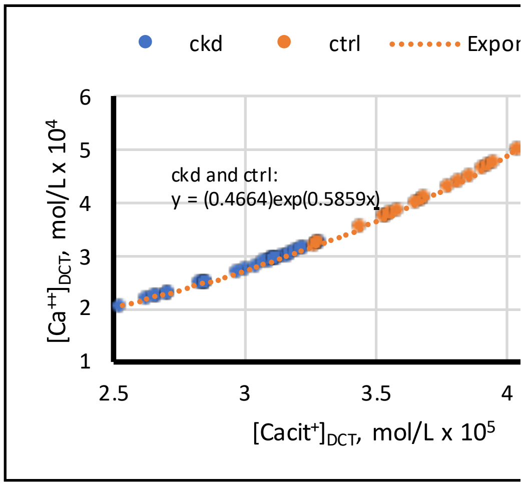

ckd
ctrl
Expor
ckd and ctrl:
y = (0.4664)exp(0.5859x)
[Ca++]DCT, mol/L x 10^4
[Cacit+]DCT, mol/L x 10^5

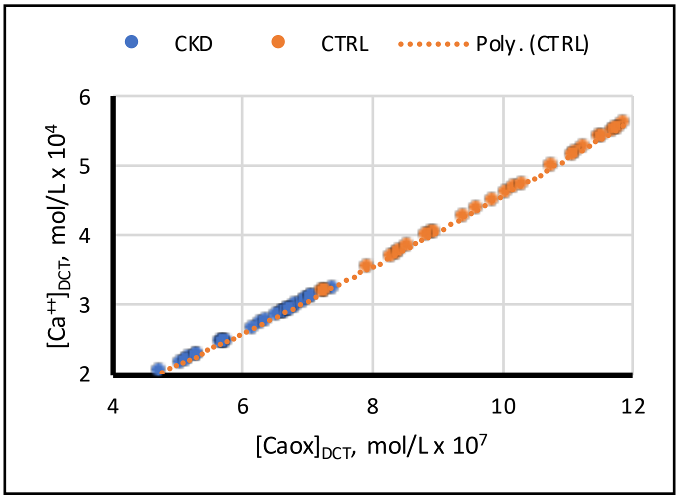

CKD
CTRL
Poly. (CTRL)
[Ca++]DCT, mol/L x 10^4
[Caox]DCT, mol/L x 10^7

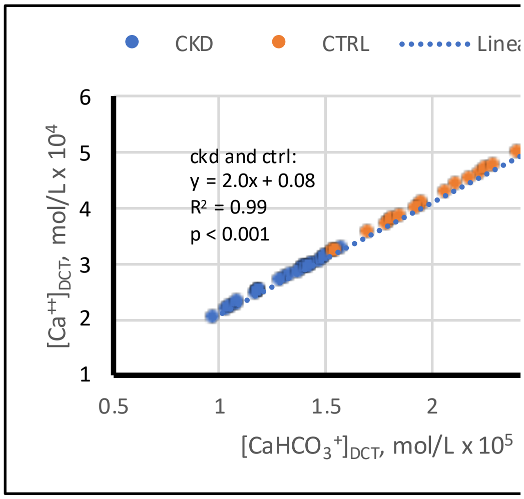

CKD
CTRL
Linea
ckd and ctrl:
y = 2.0x + 0.08
R² = 0.99
p < 0.001
[Ca++]DCT, mol/L x 10^4
[CaHCO3+]DCT, mol/L x 10^5

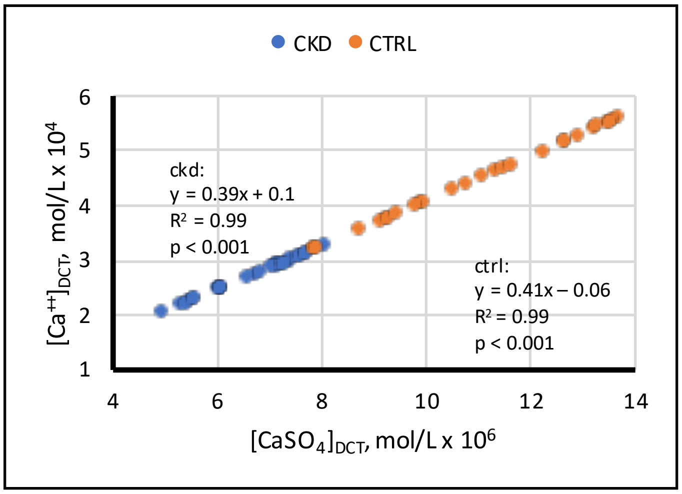

CKD
CTRL
ckd:
y = 0.39x + 0.1
R² = 0.99
p < 0.001
ctrl:
y = 0.41x – 0.06
R² = 0.99
p < 0.001
[Ca++]DCT, mol/L x 10^4
[CaSO4]DCT, mol/L x 10^6

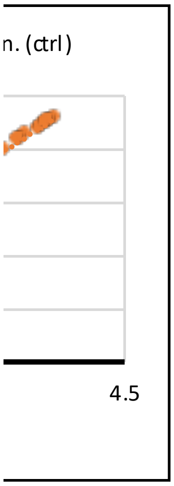
n. (ctrl)
4.5

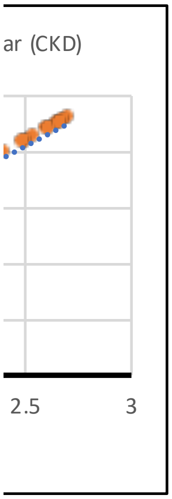
ar (CKD)
2.5
3

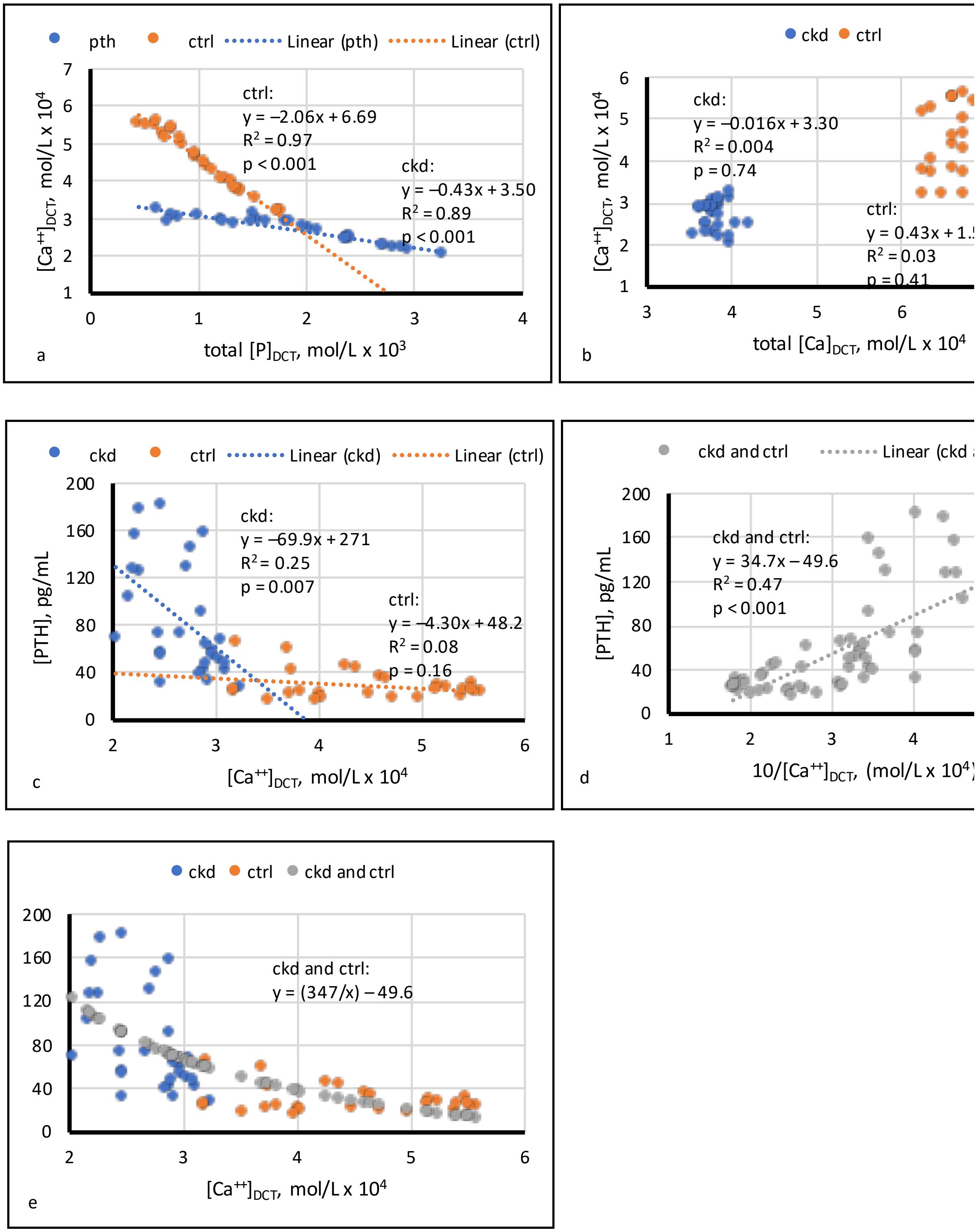
pth
ctrl
Linear (pth)
Linear (ctrl)
[Ca++]DCT, mol/L x 10^4
ctrl:
y = −2.06x + 6.69
R2 = 0.97
p < 0.001
ckd:
y = −0.43x + 3.50
R2 = 0.89
p < 0.001
total [P]DCT, mol/L x 10^3
a
ckd
ctrl
[Ca++]DCT, mol/L x 10^4
ckd:
y = −0.016x + 3.30
R2 = 0.004
p = 0.74
ctrl:
R2 = 0.03
p = 0.41
total [Ca]DCT, mol/L x 10^4
b
ckd
ctrl
Linear (ckd)
Linear (ctrl)
[PTH], pg/mL
ckd:
y = −69.9x + 271
R2 = 0.25
p = 0.007
ctrl:
y = −4.30x + 48.2
R2 = 0.08
p = 0.16
[Ca++]DCT, mol/L x 10^4
c
ckd and ctrl
[PTH], pg/mL
ckd and ctrl:
y = 34.7x − 49.6
R2 = 0.47
p < 0.001
d
ckd
ctrl
ckd and ctrl
ckd and ctrl:
y = (347/x) − 49.6
[Ca++]DCT, mol/L x 10^4
e

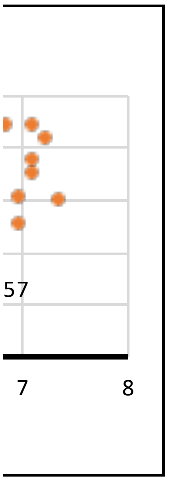
57
7
8

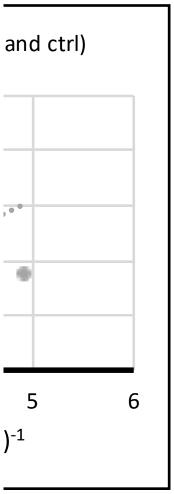
and ctrl)
5
6
)-1

code	IStr	Tot(P)	Tot(Ca)	Ca+2	CaCitric	CaOxalic	CaHPO4	CaHCO3+	CaSO4	lgSI(Ca3PO[illegible]	lgSI(Brushite)
CKD2	0.03624	0.00182	0.000378	0.000289	3.11E-05	6.64E-07	2.52E-05	1.40E-05	7.16E-06	-0.0126	-0.6165
CKD4	0.03631	0.001852	0.000385	0.000289	3.11E-05	6.64E-07	2.56E-05	1.40E-05	7.16E-06	2.07E-07	-0.6102
CKD5	0.03572	0.001218	0.000378	0.000297	3.15E-05	6.82E-07	1.74E-05	1.44E-05	7.40E-06	-0.3205	-0.7767
CKD6	0.03595	0.001549	0.000385	0.000298	3.17E-05	6.85E-07	2.22E-05	1.45E-05	7.41E-06	-0.1085	-0.6714
CKD7	0.03646	0.002036	0.000378	0.000272	3.01E-05	6.28E-07	2.64E-05	1.32E-05	6.73E-06	4.14E-07	-0.597
CKD11	0.03537	0.000824	0.000378	0.000303	3.18E-05	6.94E-07	1.21E-05	1.47E-05	7.56E-06	-0.633	-0.937
CKD13	0.03675	0.002375	0.000371	0.000247	2.85E-05	5.73E-07	2.77E-05	1.19E-05	6.09E-06	0	-0.5755
CKD14	0.03642	0.001973	0.000378	0.000278	3.04E-05	6.40E-07	2.61E-05	1.34E-05	6.87E-06	-4.14E-07	-0.6013
CKD15	0.03716	0.002803	0.000356	0.000222	2.68E-05	5.18E-07	2.92E-05	1.07E-05	5.45E-06	2.07E-07	-0.552
CKD18	0.03553	0.001003	0.000385	0.000306	3.20E-05	7.01E-07	1.48E-05	1.49E-05	7.63E-06	-0.4493	-0.8475
CKD20	0.03676	0.002391	0.000406	0.000247	2.85E-05	5.74E-07	2.77E-05	1.19E-05	6.11E-06	4.14E-07	-0.576
CKD21	0.03706	0.002718	0.000378	0.000227	2.71E-05	5.29E-07	2.89E-05	1.09E-05	5.59E-06	0	-0.5571
CKD23	0.03615	0.001624	0.000378	0.000292	3.13E-05	6.70E-07	2.27E-05	1.41E-05	7.23E-06	-0.09839	-0.6614
CKD24	0.03727	0.002935	0.000385	0.000216	2.64E-05	5.05E-07	2.96E-05	1.04E-05	5.31E-06	1.24E-06	-0.5466
CKD25	0.03609	0.001507	0.000399	0.000311	3.23E-05	7.09E-07	2.24E-05	1.50E-05	7.70E-06	-0.08215	-0.6669
CKD26	0.03757	0.003251	0.000399	0.000203	2.53E-05	4.75E-07	3.06E-05	9.72E-06	4.97E-06	8.28E-07	-0.5326
CKD27	0.03679	0.002414	0.000385	0.000245	2.84E-05	5.69E-07	2.78E-05	1.18E-05	6.04E-06	2.07E-07	-0.5739
CKD31	0.03679	0.002402	0.000421	0.000247	2.85E-05	5.74E-07	2.77E-05	1.19E-05	6.10E-06	-6.21E-07	-0.576
CKD32	0.036	0.001489	0.000371	0.000288	3.10E-05	6.62E-07	2.06E-05	1.39E-05	7.15E-06	-0.1902	-0.7044
CKD33	0.03676	0.002371	0.000371	0.000247	2.85E-05	5.74E-07	2.77E-05	1.19E-05	6.10E-06	0	-0.5758
CKD45	0.03722	0.002874	0.000399	0.00022	2.66E-05	5.13E-07	2.94E-05	1.06E-05	5.40E-06	0	-0.55
CKD46	0.03587	0.001331	0.000364	0.000284	3.08E-05	6.54E-07	1.82E-05	1.38E-05	7.07E-06	-0.303	-0.7581
CKD49	0.03574	0.001242	0.000371	0.000291	3.12E-05	6.69E-07	1.74E-05	1.41E-05	7.25E-06	-0.3306	-0.7772
CKD50	0.03537	0.000758	0.000385	0.00031	3.22E-05	7.09E-07	1.13E-05	1.51E-05	7.73E-06	-0.6764	-0.9637
CKD51	0.03651	0.002103	0.000385	0.000267	2.98E-05	6.17E-07	2.66E-05	1.29E-05	6.61E-06	2.07E-07	-0.5929
CKD55	0.03525	0.000714	0.000364	0.000292	3.12E-05	6.73E-07	1.01E-05	1.42E-05	7.32E-06	-0.8007	-1.013
CKD59	0.03516	0.000619	0.000399	0.000324	3.29E-05	7.38E-07	9.70E-06	1.58E-05	8.10E-06	-0.7926	-1.031
CKD62	0.03707	0.002702	0.000371	0.000227	2.72E-05	5.30E-07	2.89E-05	1.09E-05	5.60E-06	-6.21E-07	-0.5577
N2	0.03582	0.001258	0.000699	0.000402	3.68E-05	8.94E-07	2.17E-05	1.95E-05	9.94E-06	2.07E-07	-0.6819
N3	0.03574	0.001075	0.000661	0.000437	3.82E-05	9.63E-07	2.08E-05	2.12E-05	1.08E-05	-6.21E-07	-0.7001
N4	0.03575	0.001127	0.000674	0.000427	3.78E-05	9.43E-07	2.10E-05	2.07E-05	1.05E-05	6.21E-07	-0.6949
N6	0.03559	0.00059	0.000661	0.000549	4.21E-05	1.17E-06	1.54E-05	2.66E-05	1.35E-05	-0.1622	-0.8306
N7	0.03567	0.000867	0.000674	0.000497	4.04E-05	1.08E-06	1.95E-05	2.41E-05	1.23E-05	-1.24E-06	-0.7281
N8	0.03614	0.001751	0.000649	0.000319	3.28E-05	7.27E-07	2.43E-05	1.54E-05	7.90E-06	-4.14E-07	-0.6316

N9	0.03571	0.000983	0.000661	0.000461	3.91E-05	1.01E-06	2.03E-05	2.24E-05	1.14E-05	0	-0.7116
N10	0.03589	0.001394	0.000674	0.000373	3.55E-05	8.37E-07	2.25E-05	1.81E-05	9.24E-06	4.14E-07	-0.6658
N11	0.0359	0.001392	0.000636	0.000369	3.53E-05	8.30E-07	2.26E-05	1.79E-05	9.14E-06	2.07E-07	-0.6635
N13	0.03566	0.000685	0.000636	0.000525	4.13E-05	1.13E-06	1.71E-05	2.54E-05	1.29E-05	-0.09023	-0.7849
N14	0.03565	0.000753	0.000686	0.000539	4.18E-05	1.15E-06	1.87E-05	2.61E-05	1.32E-05	0	-0.7455
N15	0.0357	0.000971	0.000674	0.000466	3.93E-05	1.02E-06	2.01E-05	2.26E-05	1.15E-05	-8.28E-07	-0.7141
N16	0.03598	0.001539	0.000699	0.000353	3.45E-05	7.95E-07	2.32E-05	1.71E-05	8.72E-06	1.04E-06	-0.6533
N17	0.03567	0.000841	0.000724	0.000516	4.10E-05	1.11E-06	1.91E-05	2.50E-05	1.27E-05	-2.07E-07	-0.7363
N18	0.03615	0.001776	0.000674	0.000318	3.27E-05	7.25E-07	2.44E-05	1.54E-05	7.87E-06	1.24E-06	-0.631
N20	0.0358	0.001206	0.000636	0.000404	3.69E-05	8.98E-07	2.16E-05	1.96E-05	9.99E-06	2.07E-07	-0.683
N21	0.03564	0.000706	0.000625	0.000514	4.10E-05	1.11E-06	1.73E-05	2.50E-05	1.27E-05	-0.08931	-0.7801
N24	0.03572	0.001062	0.000711	0.000449	3.87E-05	9.85E-07	2.05E-05	2.18E-05	1.11E-05	0	-0.7058
N25	0.03592	0.00135	0.000625	0.000375	3.56E-05	8.41E-07	2.24E-05	1.82E-05	9.29E-06	-6.21E-07	-0.667
N27	0.03583	0.001305	0.000736	0.000398	3.66E-05	8.85E-07	2.18E-05	1.93E-05	9.83E-06	0	-0.6795
N29	0.03564	0.000611	0.000674	0.000559	4.24E-05	1.19E-06	1.62E-05	2.71E-05	1.37E-05	-0.1089	-0.8079
N31	0.03569	0.000974	0.000711	0.000472	3.95E-05	1.03E-06	2.00E-05	2.29E-05	1.17E-05	-2.07E-07	-0.717
N32	0.03586	0.001335	0.000661	0.000382	3.59E-05	8.55E-07	2.22E-05	1.85E-05	9.46E-06	4.14E-07	-0.671
N33	0.03613	0.001729	0.000625	0.00032	3.28E-05	7.28E-07	2.43E-05	1.55E-05	7.92E-06	1.24E-06	-0.6321
N35	0.03544	0.000447	0.000661	0.000552	4.22E-05	1.18E-06	1.18E-05	2.68E-05	1.36E-05	-0.3938	-0.9479
N36	0.03566	0.000763	0.000711	0.000541	4.18E-05	1.16E-06	1.87E-05	2.62E-05	1.33E-05	4.14E-07	-0.7465
N38	0.03552	0.000528	0.000661	0.00055	4.21E-05	1.17E-06	1.38E-05	2.67E-05	1.35E-05	-0.2537	-0.877

CKD16	0.03626	0.001797	0.000428	0.000298	3.16E-05	6.82E-07	2.52E-05	1.44E-05	7.36E-06	4.14E-07	-0.6164
CKD41	0.03657	0.002144	0.000307	0.000229	2.73E-05	5.36E-07	2.36E-05	1.11E-05	5.68E-06	-0.1717	-0.6456

code	Ca+2	Ca++ x 10^4	inv C3	ckd	ctrl
CKD2	0.0002891	2.891	3.45901072	158	
CKD4	0.0002891	2.891	3.45901072	41	
CKD5	0.0002973	2.973	3.36360579	59	
CKD6	0.0002984	2.984	3.35120643	54	
CKD7	0.0002722	2.722	3.67376929	129	
CKD11	0.0003027	3.027	3.30360093	50	
CKD13	0.0002467	2.467	4.05350628	56	
CKD14	0.0002776	2.776	3.60230548	145	
CKD15	0.0002216	2.216	4.51263538	156	
CKD18	0.0003061	3.061	3.26690624	67	
CKD20	0.0002473	2.473	4.04367165	182	
CKD21	0.0002268	2.268	4.40917108	126	
CKD23	0.0002918	2.918	3.4270048	63	
CKD24	0.0002161	2.161	4.62748727	103	
CKD25	0.0003106	3.106	3.21957502	42	
CKD26	0.0002028	2.028	4.93096647	69	
CKD27	0.0002449	2.449	4.08329931	72	
CKD31	0.0002472	2.472	4.04530744	31	
CKD32	0.0002878	2.878	3.47463516	91	
CKD33	0.000247	2.47	4.048583	54	
CKD45	0.0002196	2.196	4.55373406	127	
CKD46	0.0002842	2.842	3.51864884	39	
CKD49	0.0002911	2.911	3.43524562	48	
CKD50	0.0003097	3.097	3.22893122	48	
CKD51	0.0002671	2.671	3.74391614	73	
CKD55	0.0002923	2.923	3.42114266	32	
CKD59	0.0003236	3.236	3.09023486	28	
CKD62	0.0002274	2.274	4.39753738	178	
N2	0.000402	4.02	2.48756219		21
N3	0.0004371	4.371	2.28780599		44
N4	0.0004267	4.267	2.34356691		45
N6	0.0005485	5.485	1.82315406		31
N7	0.0004971	4.971	2.01166767		18
N8	0.0003191	3.191	3.13381385		24
N9	0.0004608	4.608	2.17013889		36
N10	0.0003733	3.733	2.67881061		22
N11	0.0003694	3.694	2.70709258		60
N13	0.0005246	5.246	1.90621426		28
N14	0.0005385	5.385	1.85701021		20
N15	0.0004661	4.661	2.14546235		34
N16	0.0003525	3.525	2.83687943		17
N17	0.0005162	5.162	1.93723363		29
N18	0.0003181	3.181	3.14366551		25
N20	0.000404	4.04	2.47524752		19
N21	0.0005143	5.143	1.94439043		26
N24	0.0004487	4.487	2.22866057		21
N25	0.0003754	3.754	2.66382525		41

N27	0.0003975	3.975	2.51572327	16
N29	0.0005587	5.587	1.78986934	23
N31	0.0004724	4.724	2.11685013	19
N32	0.0003823	3.823	2.6157468	24
N33	0.0003198	3.198	3.12695435	65
N35	0.0005522	5.522	1.81093807	24
N36	0.000541	5.41	1.84842884	25
N38	0.0005501	5.501	1.8178513	26

ckd and ctrl
70.4276721
70.4276721
67.1171208
66.6868633
77.8797943
65.0349521
91.056668
75.4
106.988448
63.7616465
90.7154064
103.398236
69.3170665
110.973808
62.1192531
121.504536
92.0904859
90.7721683
70.9698402
90.88583
108.414572
72.4971147
69.603023
62.4439135
80.3138899
69.1136504
57.6311496
102.994547
36.718408
29.786868
31.7217717
13.6634458
20.2048682
59.1433406
25.7038194
43.3547281
44.3361126
16.5456348
14.8382544
24.8475434
48.8397163
17.622007
59.4851933
36.2910891
17.870348
27.734522
42.8347363

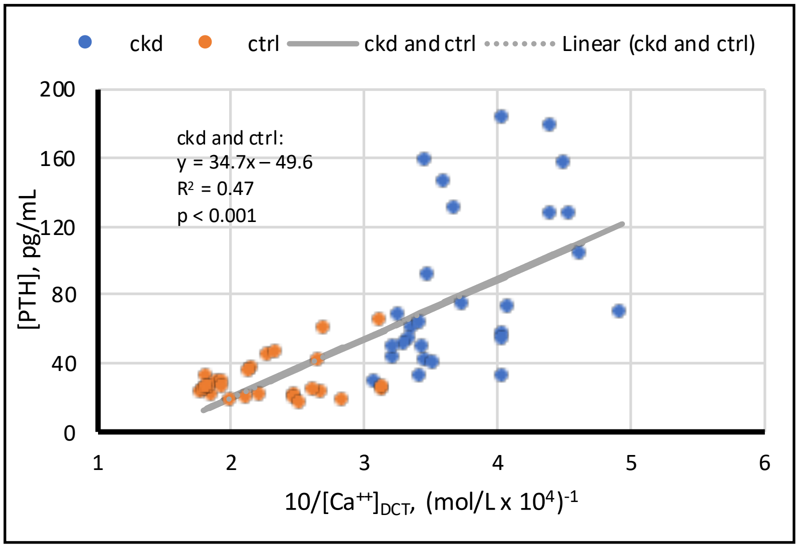

37.6955975
12.5084661
23.8546994
41.1664138
58.9053158
13.2395509
14.5404806
13.4794401

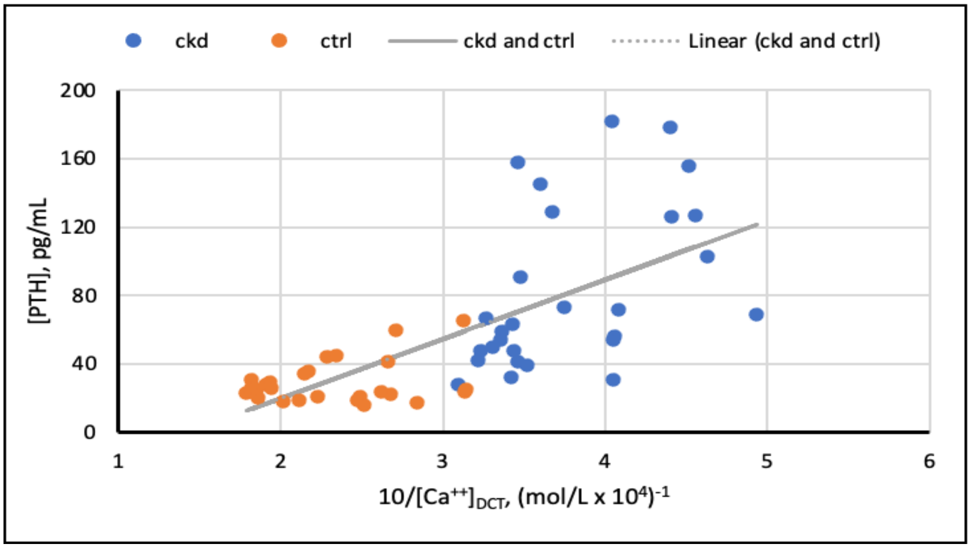
ckd
ctrl
ckd and ctrl
Linear (ckd and ctrl)
200
160
120
80
40
0
[PTH], pg/mL
1
2
3
4
5
6
10/[Ca++]DCT, (mol/L x 10^4)^-1

code	[P]s	EP	Ecr	EP/Ecr	[P]u	[cr]s	[cr]u	spot EP/Ccr
CKD2	2.7	597	1024.3	0.582837	27.1	2.9	53.4	1.471722846
CKD4	2.5	665.4	1458	0.456379	47.7	2	144.5	0.660207612
CKD5	2.5	646.8	1468	0.440599	45.3	2.1	156.2	0.609026889
CKD6	4.2	992.4	1157.2	0.857587	93.2	1.7	91.2	1.737280702
CKD7	3.8	604.3	1228.9	0.491741	55.3	3.4	127.9	1.47005473
CKD11	3.2	424.6	731	0.580848	42.2	2	99.5	0.848241206
CKD13	3.7	816.4	1224.6	0.666667	29.5	2.8	49.2	1.678861789
CKD14	5.3	431.5	1148.5	0.375707	43.3	4.8	101.7	2.043657817
CKD15	4.4	963.6	1195.3	0.806157	29.5	2.9	40	2.13875
CKD18	3.3	564	746	0.756032	40.5	1.8	61.5	1.185365854
CKD20	3.7	747	1164	0.641753	34.6	3.1	63.2	1.697151899
CKD21	4.4	1189	1372.6	0.866239	79	2.3	108.9	1.668503214
CKD23	3.4	888.3	1076	0.825558	31	1.9	39.4	1.494923858
CKD24	4.8	917	1311	0.699466	44.1	3	53.8	2.459107807
CKD25	3.9	1036	1922.2	0.538966	22.6	1.6	34.6	1.045086705
CKD26	4.5	914.3	1293.6	0.706787	47.6	3.5	64.7	2.57496136
CKD27	2.7	1056	1339	0.788648	100.1	2.3	100.1	2.3
CKD31	4	863.2	1169.6	0.73803	65.2	2.7	81.9	2.149450549
CKD32	3.9	674.5	853.6	0.790183	41	2.2	46.6	1.935622318
CKD33	4.1	1037.3	1605.9	0.645931	52	2.3	97.8	1.222903885
CKD45	2.7	1302.1	1710.4	0.761284	58.3	2.2	75.2	1.705585106
CKD46	3.2	873.1	1674.2	0.521503	43.6	1.6	178.7	0.39037493
CKD49	2.9	814.8	1146.5	0.710685	41	1.6	68	0.964705882
CKD50	3.1	579.9	1055	0.549668	19.7	1.4	46.3	0.595680346
CKD51	2.8	1117	1866	0.598607	43.4	2	126.8	0.684542587
CKD55	2.6	312.4	1167.3	0.267626	10.6	2.8	70.3	0.422190612
CKD59	3.5	454.7	1080.7	0.420746	38.5	1.7	104.2	0.628119002
CKD62	3.6	1139.8	1536.3	0.741912	19.3	2.8	26.1	2.070498084
CKD16	3.8	617.6	865.6	0.713494	69.5	2.7	99	1.895454545
CKD41	2.7	904.5	1679.1	0.538681	14.5	2.4	53.2	0.654135338

TRP/Ccr	FEP	FTRP	PTH 1-84	FGF23	eGFR	100/eGFR	1,25	spot ECa/Ccr
1.228277	0.545083	0.454917	158	35.403	21	4.761905	25.1	0.168352
1.839792	0.264083	0.735917	41	13.383	23	4.347826	38	0.038754
1.890973	0.243611	0.756389	59	13.543	34	2.941176	55.9	0.026888604
2.462719	0.413638	0.586362	54	26.494	41	2.439024	74.7	0.09693
2.329945	0.386857	0.613143	129	30.706	19	5.263158	39.6	0.053167
2.351759	0.265075	0.734925	50	17.384	33	3.030303	89.6	0.046231
2.02114	0.453746	0.546254	56	23.805	22	4.545455	20.4	0.11382
3.25634	0.385596	0.614404	145	68.316	14	7.142857	64.2	0.0944
2.26125	0.48608	0.51392	156	26	22	4.545455	27.6	0.145
2.114634	0.359202	0.640798	67	17.415	36	2.777778	57.2	0.058537
2.002848	0.45869	0.54131	182	43.713	20	5	33.7	0.122627
2.731497	0.379205	0.620795	126	26.399	28	3.571429	19.3	0.033792
1.905076	0.439683	0.560317	63	35.711	35	2.857143	49.6	0.0192893
2.340892	0.512314	0.487686	103	48.446	20	5	25.1	0.027881
2.854913	0.267971	0.732029	42	12.507	44	2.272727	52.6	0.0693642
1.925039	0.572214	0.427786	69	35.564	18	5.555556	21.4	0.1990108
1.610889	0.851852	0.148148	72	42.96	28	3.571429	44.9	0.018382
1.85055	0.537363	0.462637	31	24.519	23	4.347826		0.1054945
1.964378	0.496313	0.503687	91	89.109	29	3.448276	21.7	0.037768
2.877096	0.298269	0.701731	54	26.285	28	3.571429	27.3	0.0188139
0.994415	0.631698	0.368302	127	25.303	29	3.448276	47.8	0.008777
2.80963	0.121992	0.878008	39	17.44	42	2.380952	31.8	0.03671
1.935294	0.332657	0.667343	48	48.504	42	2.380952	25.5	0.08
2.50432	0.192155	0.807845	48	14.623	49	2.040816	75.1	0.06955
2.11546	0.244479	0.755521	73	19.062	34	2.941176	26.5	0.00158
2.17781	0.162381	0.837619	32	20.402	28	3.571429	43.4	0.09161
2.871881	0.179463	0.820537	28	16.126	47	2.12766	74.9	0.01142
1.529502	0.575138	0.424862	178	20.506	27	3.703704	53.1	0.214559
1.904545	0.498804	0.501196	169	36.399	22	4.545455	58.6	0.054545
2.04586	0.242272	0.757728	79	38.187	27	3.703704	30	0.01353

24h Eca, mg	24h Eca/Ccr	Cai	24h EP/Ccr
49.1	0.13901201	4.60805	1.6902275
23.3	0.03196159	5.08889	0.9127572
155.1	0.2218733	4.8084	0.9252589
63.6	0.09343242	5.00875	1.4578984
38.9	0.10762471	4.92861	1.671918
45.1	0.12339261	4.92861	1.1616963
10.4	0.02377919	5.04882	1.8666667
**23.2**	**0.09696125**	4.52791	1.8033957
**43.6**	**0.10578098**	4.72826	2.3378566
20	0.04825737	4.84847	1.3608579
58.5	0.15579897	5.04882	1.989433
57	0.09551217	4.76833	1.9923503
18.9	0.03337361	5.12896	1.5685595
19	0.04347826	4.72826	2.0983982
39.2	0.03262928	5.24917	0.8623452
45.1	0.12202381	5.44952	2.4737554
68.8	0.11817774	5.12896	1.813891
64.7	0.14935876	5.12896	1.9926813
32.8	0.08453608	4.8084	1.7384021
25.3	0.03623513	5.00875	1.4856405
11.6	0.01492049	4.8084	1.6748246
55.8	0.05332696	4.96868	0.8344045
77.6	0.10829481	4.96868	1.1370955
30	0.03981043	4.88854	0.7695355
19.2	0.02057878	5.08889	1.1972133
16.5	0.03957851	5.00875	0.7493532
7.4	0.0116406	5.00875	0.7152679
53.3	0.09714249	5.16903	2.0773547
36.5	0.11385166	5.40945	1.9264325
12.1	0.01729498	4.88854	1.2928354

code	24h Eca, mg
CKD2	49.1
CKD4	23.3
CKD5	155.1
CKD6	63.6
CKD7	38.9
CKD11	45.1
CKD13	10.4
CKD14	**23.2**
CKD15	**43.6**
CKD18	20
CKD20	58.5
CKD21	57
CKD23	18.9
CKD24	19
CKD25	39.2
CKD26	45.1
CKD27	68.8
CKD31	64.7
CKD32	32.8
CKD33	25.3
CKD45	11.6
CKD46	55.8
CKD49	77.6
CKD50	30
CKD51	19.2
CKD55	16.5
CKD59	7.4
CKD62	53.3

Column1	
Mean	41.89285714
Standard Error	5.600335233
Median	39.05
Mode	45.1
Standard Deviation	29.63418857
Sample Variance	878.1851323
Kurtosis	6.961796166
Skewness	2.096365559
Range	147.7
Minimum	7.4
Maximum	155.1
Sum	1173
Count	28

CODE
N2
N3
N4
N6
N7
N8
N9
N10
N11
N13
N14
N15
N16
N17
N18
N20
N21
N24
N25
N27
N29
N31
N32
N33
N35
N36
N38

24h ECa

127.5
139.2
71.2
49
96.3
110.9
104.5
138.5
263.4
210
103.7
75.9
158.2
122.4
236.8
100.9
60.9
344.4
169.6
227.9
37
54.1
97
113.1
116.6
53.9
200

Column1	
Mean	132.7
Standard Error	14.20272134
Median	113.1
Mode	#N/A
Standard Deviation	73.7995049
Sample Variance	5446.366923
Kurtosis	1.236310083
Skewness	1.149776631
Range	307.4
Minimum	37
Maximum	344.4
Sum	3582.9
Count	27

t-Test: Two-Sample Assuming Unequal Variances

	Variable 1	*Variable 2*
Mean	41.89285714	132.7
Variance	878.1851323	5446.366923
Observations	28	27
Hypothesized Mean Difference	0	
df	34	
t Stat	-5.947939331	
P(T<=t) one-tail	5.03098E-07	
t Critical one-tail	1.690924255	
P(T<=t) two-tail	1.0062E-06	
t Critical two-tail	2.032244509	

code	[P]s	EP	Ecr	EP/Ecr	[P]u	[cr]s	[cr]u	EP/Ccr	TRP/Ccr	FEP	FTRP	pth 1-84
CKD2	2.7	597	1024.3	0.582837	27.1	2.9	53.4	1.471723	1.228277	0.545083	0.454917	158
CKD4	2.5	665.4	1458	0.456379	47.7	2	144.5	0.660208	1.839792	0.264083	0.735917	41
CKD5	2.5	646.8	1468	0.440599	45.3	2.1	156.2	0.609027	1.890973	0.243611	0.756389	59
CKD6	4.2	992.4	1157.2	0.857587	93.2	1.7	91.2	1.737281	2.462719	0.413638	0.586362	54
CKD7	3.8	604.3	1228.9	0.491741	55.3	3.4	127.9	1.470055	2.329945	0.386857	0.613143	129
CKD11	3.2	424.6	731	0.580848	42.2	2	99.5	0.848241	2.351759	0.265075	0.734925	50
CKD13	3.7	816.4	1224.6	0.666667	29.5	2.8	49.2	1.678862	2.02114	0.453746	0.546254	56
CKD14	5.3	431.5	1148.5	0.375707	43.3	4.8	101.7	2.043658	3.25634	0.385596	0.614404	145
CKD15	4.4	963.6	1195.3	0.806157	29.5	2.9	40	2.13875	2.26125	0.48608	0.51392	156
CKD18	3.3	564	746	0.756032	40.5	1.8	61.5	1.185366	2.114634	0.359202	0.640798	67
CKD20	3.7	747	1164	0.641753	34.6	3.1	63.2	1.697152	2.002848	0.45869	0.54131	182
CKD21	4.4	1189	1372.6	0.866239	79	2.3	108.9	1.668503	2.731497	0.379205	0.620795	126
CKD23	3.4	888.3	1076	0.825558	31	1.9	39.4	1.494924	1.905076	0.439683	0.560317	63
CKD24	4.8	917	1311	0.699466	44.1	3	53.8	2.459108	2.340892	0.512314	0.487686	103
CKD25	3.9	1036	1922.2	0.538966	22.6	1.6	34.6	1.045087	2.854913	0.267971	0.732029	42
CKD26	4.5	914.3	1293.6	0.706787	47.6	3.5	64.7	2.574961	1.925039	0.572214	0.427786	69
CKD27	2.7	1056	1339	0.788648	100.1	2.3	100.1	2.3	1.610889	0.851852	0.148148	72
CKD31	4	863.2	1169.6	0.73803	65.2	2.7	81.9	2.149451	1.85055	0.537363	0.462637	31
CKD32	3.9	674.5	853.6	0.790183	41	2.2	46.6	1.935622	1.964378	0.496313	0.503687	91
CKD33	4.1	1037.3	1605.9	0.645931	52	2.3	97.8	1.222904	2.877096	0.298269	0.701731	54
CKD45	2.7	1302.1	1710.4	0.761284	58.3	2.2	75.2	1.705585	0.994415	0.631698	0.368302	127
CKD46	3.2	873.1	1674.2	0.521503	43.6	1.6	178.7	0.390375	2.80963	0.121992	0.878008	39
CKD49	2.9	814.8	1146.5	0.710685	41	1.6	68	0.964706	1.935294	0.332657	0.667343	48
CKD50	3.1	579.9	1055	0.549668	19.7	1.4	46.3	0.59568	2.50432	0.192155	0.807845	48
CKD51	2.8	1117	1866	0.598607	43.4	2	126.8	0.684543	2.11546	0.244479	0.755521	73
CKD55	2.6	312.4	1167.3	0.267626	10.6	2.8	70.3	0.422191	2.17781	0.162381	0.837619	32
CKD59	3.5	454.7	1080.7	0.420746	38.5	1.7	104.2	0.628119	2.871881	0.179463	0.820537	28
CKD62	3.6	1139.8	1536.3	0.741912	19.3	2.8	26.1	2.070498	1.529502	0.575138	0.424862	178
CKD16	3.8	617.6	865.6	0.713494	69.5	2.7	99	1.895455	1.904545	0.498804	0.501196	169
CKD41	2.7	904.5	1679.1	0.538681	14.5	2.4	53.2	0.654135	2.04586	0.242272	0.757728	79

FGF23	1,25	eGFR	100/eGFR	25D	Cai	ECa/Ccr	[Ca]uf
35.403	25.1	21	4.761905	37.4	4.61	0.168	5.3
13.383	38	23	4.347826	42.2	5.09	0.039	5.4
13.543	55.9	34	2.941176	47.4	4.81	0.027	5.3
26.494	74.7	41	2.439024	44.3	5.09	0.097	5.4
30.706	39.6	19	5.263158	41.7	4.93	0.053	5.3
17.384	89.6	33	3.030303	55.8	4.93	0.046	5.3
23.805	20.4	22	4.545455	21	5.05	0.114	5.2
68.316	64.2	14	7.142857	34.2	4.53	0.094	5.3
26	27.6	22	4.545455	18.9	4.73	0.145	5
17.415	57.2	36	2.777778	42.7	4.85	0.059	5.4
43.713	33.7	20	5	49.1	5.05	0.123	5.7
26.399	19.3	28	3.571429	20.5	4.77	0.034	5.3
35.711	49.6	35	2.857143	27.7	5.13	0.019	5.3
48.446	25.1	20	5	31	4.73	0.028	5.4
12.507	52.6	44	2.272727	lost	5.25	0.069	5.6
35.564	21.4	18	5.555556	25.6	5.45	0.119	5.6
42.96	44.9	28	3.571429	26.4	5.13	0.018	5.4
24.519		23	4.347826	27.5	5.13	0.105	5.9
89.109	21.7	29	3.448276	32.2	4.81	0.038	5.2
26.285	27.3	28	3.571429	49.5	5.01	0.019	5.2
25.303	47.8	29	3.448276	30.1	4.81	0.009	5.6
17.44	31.8	42	2.380952	24	4.97	0.037	5.1
48.504	25.5	42	2.380952	24.6	4.97	0.08	5.2
14.623	75.1	49	2.040816	36.9	4.89	0.07	5.4
19.062	26.5	34	2.941176	17.8	5.09	0.002	5.4
20.402	43.4	28	3.571429	35.9	5.01	0.092	5.1
16.126	74.9	47	2.12766	51.9	5.01	0.011	5.6
20.506	53.1	27	3.703704	21.5	5.17	0.21	5.2
36.399	58.6	22	4.545455	71.2	5.41	0.055	6
38.187	30	27	3.703704	17.4	4.89	0.014	4.3

CODE	[cr]s	eGFR	[P]s	[Ca]i	[Ca]uf	[PTH]1-84	1-84 & 7-84	[PTH]7-84
N2	0.9	89	3.1	5.00875	5.6	21	24	3
N3	0.8	101	3.5	4.88854	5.3	44	77	33
N4	0.7	93	3.5	5.12896	5.4	45	72	27
N6	0.8	103	3	4.96868	5.3	31	52	21
N7	0.7	94	3.5	5.04882	5.4	18	28	10
N8	1	79	2.8	4.92861	5.2	24	29	5
N9	0.8	77	3.2	5.08889	5.3	36	57	21
N10	0.8	73	3.4	5.12896	5.4	22	31	9
N11	0.8	108	2.9	4.96868	5.1	60	120	60
N13	0.7	87	4.9	4.8084	5.1	28	50	22
N14	0.9	93	2.1	5.04882	5.5	20	30	10
N15	0.7	96	3.1	5.08889	5.4	34	59	25
N16	0.9	96	3.4	5.16903	5.6	17	22	5
N17	1.1	73	3.4	4.84847	5.8	29	49	20
N18	0.7	90	4	5.08889	5.2	25	45	20
N20	0.8	75	4.1	5.12896	5.1	19	32	13
N21	0.8	75	2.9	5.00875	5.00875	26	51	25
N24	0.8	75	4	5.08889	5.7	21	36	15
N25	1.1	89	3.2	5.00875	5.00875	41	82	41
N27	0.8	74	3.1	5.24917	5.9	16	27	11
N29	0.7	85	4.2	5.00875	5.4	23	46	23
N31	0.9	72	3.4	5.04882	5.7	19	30	11
N32	1.3	89	3.6	5.08889	5.3	24	36	12
N33	0.9	93	4.9	5.00875	5.00875	65	93	28
N35	0.9	78	4	4.96868	5.3	24	51	27
N36	1	84	2.7	4.96868	5.7	25	47	22
N38	0.7	87	3.2	5.2091	5.3	26	49	23
N19	1	78	3.2	5.24917	5.4	23	38	13

25D	1,25D	FGF23	24h EP	24h Ecr	24h EP/Ecr	24h EP/Ccr	spot EP/Ccr
40.5	66.7	11.018	999.6	1493.8	0.66916589	0.602249297	0.44787
27.7	38.7	4.5325	969.6	1717.8	0.56444289	0.451554314	0.41669
35.3	71.8	23.379	936	1173	0.79795396	0.558567775	0.470909
55.8	66.1	117.63	542.5	1354.8	0.40042811	0.320342486	0.186121
33.5	60	9.3336	727.2	937.3	0.77584551	0.54309186	0.438913
30.8	35	14.798	1235.2	2084.7	0.59250732	0.592507315	0.278234
46.2	55.3	17.591	675.5	1196.4	0.5646105	0.451688399	0.3855
47.8	47	12.978	908.7	1238.4	0.73376938	0.587015504	0.380812
20	90.9	15.294	1342	1105.4	1.21404017	0.971232133	0.45463
25.4	23.4	12.749	532	910	0.58461538	0.409230769	0.583639
24.9	25.8	34.843	625	1416.1	0.44135301	0.397217711	0.21772
22.1	83.3	16.026	832.6	1279.5	0.65072294	0.455506057	0.445848
30.7	68.5	32.967	1319.3	1731.6	0.76189651	0.685706861	0.589039
21.2	53.9	8.3066	548.3	2387.5	0.22965445	0.252619895	0.287347
27.4	28.1	16.158	1427.2	1272.9	1.12121926	0.784853484	0.43
32.5	35.1	13.3182	807.8	1426.5	0.56628111	0.453024886	0.343947
n/a	36.4	12.5182	472.6	867.8	0.54459553	0.435676423	0.292998
19.8	52.8	12.1842	711	1138.5	0.62450593	0.499604743	0.38037
17.5	31.7	7.932	1072.7	2022.9	0.53027831	0.583306145	0.668646
98.1	136.3	10.6568	862.4	1252.8	0.68837803	0.550702427	0.366213
31.1	91	11.7211	463.5	1039.5	0.44588745	0.312121212	0.225379
22.9	94.2	12.6715	626.1	1058.3	0.59160918	0.532448266	0.38008
40.4	77.1	10.5577	882	2416	0.36506623	0.474586093	0.31234
15.2	25	19.7492	1436	2001.7	0.71739022	0.645651196	0.46075
29.6	36.7	15.9907	311	861.7	0.36091447	0.324823024	0.58508
21.9	41.6	5.8652	572	805.2	0.71038251	0.710382514	0.5
30.3	64.6	23.7653	410.3	801.5	0.51191516	0.358340611	0.110526
17	38.5	14.5962	n/a	n/a	n/a	n/a	0.8197941

spot TRP/Ccr	24h ECa	24h ECa/Ecr	24h ECa/Ccr	spot ECa/Ccr	spot TRCa/Ccr	100/eGFR
2.65213	127.5	0.085352792	0.076817512	0.08872	5.51128	1.123596
3.08331	139.2	0.081033881	0.064827104	0.02369	5.27631	0.990099
3.029091	71.2	0.060699062	0.042489344	0.082197	5.317803	1.075269
2.813879	49	0.0361677	0.02893416	0.008181	5.281819	0.970874
3.061087	96.3	0.102741918	0.071919343	0.091483	5.308517	1.06383
2.521766	110.9	0.053197103	0.053197103	0.067762	5.132238	1.265823
2.8145	104.5	0.087345369	0.069876296	0.04264	5.25736	1.298701
3.019188	138.5	0.111837855	0.089470284	0.066421	5.333579	1.369863
2.44537	263.4	0.238284784	0.190627827	0.11654	4.98	0.925926
4.316361	210	0.230769231	0.161538462	0.116361	4.983639	1.149425
1.88228	103.7	0.073229292	0.065906363	0.044376	5.455624	1.075269
2.654152	75.9	0.059320047	0.041524033	0.022022	5.377978	1.041667
2.810961	158.2	0.091360591	0.082224532	0.084353	5.515647	1.041667
3.112653	122.4	0.051267016	0.056393717	0.044898	5.755102	1.369863
3.57	236.8	0.186031896	0.130222327	0.05125	5.14875	1.111111
3.756053	100.9	0.070732562	0.05658605	0.014674	5.085326	1.333333
2.607002	60.9	0.07017746	0.056141968	0.010054	4.998696	1.333333
3.61963	344.4	0.302503294	0.242002635	0.07507	5.62493	1.333333
2.531354	169.6	0.083840032	0.092224035	0.042271	4.966479	1.123596
2.733787	227.9	0.181912516	0.145530013	0.13279	5.76703	1.351351
3.974621	37	0.035594036	0.024915825	0.031818	5.368182	1.176471
3.01992	54.1	0.05111972	0.046007748	0.024502	5.675498	1.388889
3.28766	97	0.040149007	0.052193709	0.0726	5.2274	1.123596
4.43925	113.1	0.056501973	0.050851776	0.01593	4.99282	1.075269
3.41492	116.6	0.135313914	0.121782523	0.01924	5.28076	1.282051
2.2	53.9	0.066939891	0.066939891	n/a	5.7	1.190476
3.089474	200	0.249532127	0.174672489	0.090526	5.209474	1.149425
3.0102059	n/a	n/a	n/a	0.0734109	5.3265891	1.282051

CODE	[cr]s	eGFR	[P]s	[Ca]i	[Ca]uf	[PTH]1-84	1-84 & 7-84	[PTH]7-84
N2	0.9	89	3.1	5.00875	5.6	21	24	3
N3	0.8	101	3.5	4.88854	5.3	44	77	33
N4	0.7	93	3.5	5.12896	5.4	45	72	27
N6	0.8	103	3	4.96868	5.3	31	52	21
N7	0.7	94	3.5	5.04882	5.4	18	28	10
N8	1	79	2.8	4.92861	5.2	24	29	5
N9	0.8	77	3.2	5.08889	5.3	36	57	21
N10	0.8	73	3.4	5.12896	5.4	22	31	9
N11	0.8	108	2.9	4.96868	5.1	60	120	60
N13	0.7	87	4.9	4.8084	5.1	28	50	22
N14	0.9	93	2.1	5.04882	5.5	20	30	10
N15	0.7	96	3.1	5.08889	5.4	34	59	25
N16	0.9	96	3.4	5.16903	5.6	17	22	5
N17	1.1	73	3.4	4.84847	5.8	29	49	20
N18	0.7	90	4	5.08889	5.2	25	45	20
N20	0.8	75	4.1	5.12896	5.1	19	32	13
N21	0.8	75	2.9	5.00875	5.00875	26	51	25
N24	0.8	75	4	5.08889	5.7	21	36	15
N25	1.1	89	3.2	5.00875	5.00875	41	82	41
N27	0.8	74	3.1	5.24917	5.9	16	27	11
N29	0.7	85	4.2	5.00875	5.4	23	46	23
N31	0.9	72	3.4	5.04882	5.7	19	30	11
N32	1.3	89	3.6	5.08889	5.3	24	36	12
N33	0.9	93	4.9	5.00875	5.00875	65	93	28
N35	0.9	78	4	4.96868	5.3	24	51	27
N36	1	84	2.7	4.96868	5.7	25	47	22
N38	0.7	87	3.2	5.2091	5.3	26	49	23
N19	1	78	3.2	5.24917	5.4	23	38	13

25D	1,25D	FGF23	24h EP	24h Ecr	24h EP/Ecr	24h EP/Ccr	spot EP/Ccr
40.5	66.7	11.018	999.6	1493.8	0.66916589	0.602249297	0.44787
27.7	38.7	4.5325	969.6	1717.8	0.56444289	0.451554314	0.41669
35.3	71.8	23.379	936	1173	0.79795396	0.558567775	0.470909
55.8	66.1	117.63	542.5	1354.8	0.40042811	0.320342486	0.186121
33.5	60	9.3336	727.2	937.3	0.77584551	0.54309186	0.438913
30.8	35	14.798	1235.2	2084.7	0.59250732	0.592507315	0.278234
46.2	55.3	17.591	675.5	1196.4	0.5646105	0.451688399	0.3855
47.8	47	12.978	908.7	1238.4	0.73376938	0.587015504	0.380812
20	90.9	15.294	1342	1105.4	1.21404017	0.971232133	0.45463
25.4	23.4	12.749	532	910	0.58461538	0.409230769	0.583639
24.9	25.8	34.843	625	1416.1	0.44135301	0.397217711	0.21772
22.1	83.3	16.026	832.6	1279.5	0.65072294	0.455506057	0.445848
30.7	68.5	32.967	1319.3	1731.6	0.76189651	0.685706861	0.589039
21.2	53.9	8.3066	548.3	2387.5	0.22965445	0.252619895	0.287347
27.4	28.1	16.158	1427.2	1272.9	1.12121926	0.784853484	0.43
32.5	35.1	13.3182	807.8	1426.5	0.56628111	0.453024886	0.343947
n/a	36.4	12.5182	472.6	867.8	0.54459553	0.435676423	0.292998
19.8	52.8	12.1842	711	1138.5	0.62450593	0.499604743	0.38037
17.5	31.7	7.932	1072.7	2022.9	0.53027831	0.583306145	0.668646
98.1	136.3	10.6568	862.4	1252.8	0.68837803	0.550702427	0.366213
31.1	91	11.7211	463.5	1039.5	0.44588745	0.312121212	0.225379
22.9	94.2	12.6715	626.1	1058.3	0.59160918	0.532448266	0.38008
40.4	77.1	10.5577	882	2416	0.36506623	0.474586093	0.31234
15.2	25	19.7492	1436	2001.7	0.71739022	0.645651196	0.46075
29.6	36.7	15.9907	311	861.7	0.36091447	0.324823024	0.58508
21.9	41.6	5.8652	572	805.2	0.71038251	0.710382514	0.5
30.3	64.6	23.7653	410.3	801.5	0.51191516	0.358340611	0.110526
17	38.5	14.5962	n/a	n/a	n/a	n/a	0.8197941

spot TRP/Ccr	24h ECa	24h ECa/Ecr	24h ECa/Ccr	spot ECa/Ccr	spot TRCa/Ccr	100/eGFR
2.65213	127.5	0.085352792	0.076817512	0.08872	5.51128	1.123596
3.08331	139.2	0.081033881	0.064827104	0.02369	5.27631	0.990099
3.029091	71.2	0.060699062	0.042489344	0.082197	5.317803	1.075269
2.813879	49	0.0361677	0.02893416	0.008181	5.281819	0.970874
3.061087	96.3	0.102741918	0.071919343	0.091483	5.308517	1.06383
2.521766	110.9	0.053197103	0.053197103	0.067762	5.132238	1.265823
2.8145	104.5	0.087345369	0.069876296	0.04264	5.25736	1.298701
3.019188	138.5	0.111837855	0.089470284	0.066421	5.333579	1.369863
2.44537	263.4	0.238284784	0.190627827	0.11654	4.98	0.925926
4.316361	210	0.230769231	0.161538462	0.116361	4.983639	1.149425
1.88228	103.7	0.073229292	0.065906363	0.044376	5.455624	1.075269
2.654152	75.9	0.059320047	0.041524033	0.022022	5.377978	1.041667
2.810961	158.2	0.091360591	0.082224532	0.084353	5.515647	1.041667
3.112653	122.4	0.051267016	0.056393717	0.044898	5.755102	1.369863
3.57	236.8	0.186031896	0.130222327	0.05125	5.14875	1.111111
3.756053	100.9	0.070732562	0.05658605	0.014674	5.085326	1.333333
2.607002	60.9	0.07017746	0.056141968	0.010054	4.998696	1.333333
3.61963	344.4	0.302503294	0.242002635	0.07507	5.62493	1.333333
2.531354	169.6	0.083840032	0.092224035	0.042271	4.966479	1.123596
2.733787	227.9	0.181912516	0.145530013	0.13279	5.76703	1.351351
3.974621	37	0.035594036	0.024915825	0.031818	5.368182	1.176471
3.01992	54.1	0.05111972	0.046007748	0.024502	5.675498	1.388889
3.28766	97	0.040149007	0.052193709	0.0726	5.2274	1.123596
4.43925	113.1	0.056501973	0.050851776	0.01593	4.99282	1.075269
3.41492	116.6	0.135313914	0.121782523	0.01924	5.28076	1.282051
2.2	53.9	0.066939891	0.066939891	n/a	5.7	1.190476
3.089474	200	0.249532127	0.174672489	0.090526	5.209474	1.149425
3.0102059	n/a	n/a	n/a	0.0734109	5.3265891	1.282051

code	[P]s	EP	Ecr	EP/Ecr	[P]u	[cr]s	[cr]u	EP/Ccr	TRP/Ccr	FEP	FTRP	pth 1-84
CKD2	2.7	597	1024.3	0.582837	27.1	2.9	53.4	1.471723	1.228277	0.545083	0.454917	158
CKD4	2.5	665.4	1458	0.456379	47.7	2	144.5	0.660208	1.839792	0.264083	0.735917	41
CKD5	2.5	646.8	1468	0.440599	45.3	2.1	156.2	0.609027	1.890973	0.243611	0.756389	59
CKD6	4.2	992.4	1157.2	0.857587	93.2	1.7	91.2	1.737281	2.462719	0.413638	0.586362	54
CKD7	3.8	604.3	1228.9	0.491741	55.3	3.4	127.9	1.470055	2.329945	0.386857	0.613143	129
CKD11	3.2	424.6	731	0.580848	42.2	2	99.5	0.848241	2.351759	0.265075	0.734925	50
CKD13	3.7	816.4	1224.6	0.666667	29.5	2.8	49.2	1.678862	2.02114	0.453746	0.546254	56
CKD14	5.3	431.5	1148.5	0.375707	43.3	4.8	101.7	2.043658	3.25634	0.385596	0.614404	145
CKD15	4.4	963.6	1195.3	0.806157	29.5	2.9	40	2.13875	2.26125	0.48608	0.51392	156
CKD18	3.3	564	746	0.756032	40.5	1.8	61.5	1.185366	2.114634	0.359202	0.640798	67
CKD20	3.7	747	1164	0.641753	34.6	3.1	63.2	1.697152	2.002848	0.45869	0.54131	182
CKD21	4.4	1189	1372.6	0.866239	79	2.3	108.9	1.668503	2.731497	0.379205	0.620795	126
CKD23	3.4	888.3	1076	0.825558	31	1.9	39.4	1.494924	1.905076	0.439683	0.560317	63
CKD24	4.8	917	1311	0.699466	44.1	3	53.8	2.459108	2.340892	0.512314	0.487686	103
CKD25	3.9	1036	1922.2	0.538966	22.6	1.6	34.6	1.045087	2.854913	0.267971	0.732029	42
CKD26	4.5	914.3	1293.6	0.706787	47.6	3.5	64.7	2.574961	1.925039	0.572214	0.427786	69
CKD27	2.7	1056	1339	0.788648	100.1	2.3	100.1	2.3	1.610889	0.851852	0.148148	72
CKD31	4	863.2	1169.6	0.73803	65.2	2.7	81.9	2.149451	1.85055	0.537363	0.462637	31
CKD32	3.9	674.5	853.6	0.790183	41	2.2	46.6	1.935622	1.964378	0.496313	0.503687	91
CKD33	4.1	1037.3	1605.9	0.645931	52	2.3	97.8	1.222904	2.877096	0.298269	0.701731	54
CKD45	2.7	1302.1	1710.4	0.761284	58.3	2.2	75.2	1.705585	0.994415	0.631698	0.368302	127
CKD46	3.2	873.1	1674.2	0.521503	43.6	1.6	178.7	0.390375	2.80963	0.121992	0.878008	39
CKD49	2.9	814.8	1146.5	0.710685	41	1.6	68	0.964706	1.935294	0.332657	0.667343	48
CKD50	3.1	579.9	1055	0.549668	19.7	1.4	46.3	0.59568	2.50432	0.192155	0.807845	48
CKD51	2.8	1117	1866	0.598607	43.4	2	126.8	0.684543	2.11546	0.244479	0.755521	73
CKD55	2.6	312.4	1167.3	0.267626	10.6	2.8	70.3	0.422191	2.17781	0.162381	0.837619	32
CKD59	3.5	454.7	1080.7	0.420746	38.5	1.7	104.2	0.628119	2.871881	0.179463	0.820537	28
CKD62	3.6	1139.8	1536.3	0.741912	19.3	2.8	26.1	2.070498	1.529502	0.575138	0.424862	178
CKD16	3.8	617.6	865.6	0.713494	69.5	2.7	99	1.895455	1.904545	0.498804	0.501196	169
CKD41	2.7	904.5	1679.1	0.538681	14.5	2.4	53.2	0.654135	2.04586	0.242272	0.757728	79

FGF23	1,25	eGFR	100/eGFR	25D	Cai	ECa/Ccr	[Ca]uf
35.403	25.1	21	4.761905	37.4	4.61	0.168	5.3
13.383	38	23	4.347826	42.2	5.09	0.039	5.4
13.543	55.9	34	2.941176	47.4	4.81	0.027	5.3
26.494	74.7	41	2.439024	44.3	5.09	0.097	5.4
30.706	39.6	19	5.263158	41.7	4.93	0.053	5.3
17.384	89.6	33	3.030303	55.8	4.93	0.046	5.3
23.805	20.4	22	4.545455	21	5.05	0.114	5.2
68.316	64.2	14	7.142857	34.2	4.53	0.094	5.3
26	27.6	22	4.545455	18.9	4.73	0.145	5
17.415	57.2	36	2.777778	42.7	4.85	0.059	5.4
43.713	33.7	20	5	49.1	5.05	0.123	5.7
26.399	19.3	28	3.571429	20.5	4.77	0.034	5.3
35.711	49.6	35	2.857143	27.7	5.13	0.019	5.3
48.446	25.1	20	5	31	4.73	0.028	5.4
12.507	52.6	44	2.272727	lost	5.25	0.069	5.6
35.564	21.4	18	5.555556	25.6	5.45	0.119	5.6
42.96	44.9	28	3.571429	26.4	5.13	0.018	5.4
24.519		23	4.347826	27.5	5.13	0.105	5.9
89.109	21.7	29	3.448276	32.2	4.81	0.038	5.2
26.285	27.3	28	3.571429	49.5	5.01	0.019	5.2
25.303	47.8	29	3.448276	30.1	4.81	0.009	5.6
17.44	31.8	42	2.380952	24	4.97	0.037	5.1
48.504	25.5	42	2.380952	24.6	4.97	0.08	5.2
14.623	75.1	49	2.040816	36.9	4.89	0.07	5.4
19.062	26.5	34	2.941176	17.8	5.09	0.002	5.4
20.402	43.4	28	3.571429	35.9	5.01	0.092	5.1
16.126	74.9	47	2.12766	51.9	5.01	0.011	5.6
20.506	53.1	27	3.703704	21.5	5.17	0.21	5.2
36.399	58.6	22	4.545455	71.2	5.41	0.055	6
38.187	30	27	3.703704	17.4	4.89	0.014	4.3

code	Tot(Ca)	Ca+2	tot Ca x 10^4	ckd	ctrl
CKD2	0.0003778	0.0002891	3.778	2.891	
CKD4	0.000385	0.0002891	3.85	2.891	
CKD5	0.0003778	0.0002973	3.778	2.973	
CKD6	0.000385	0.0002984	3.85	2.984	
CKD7	0.0003778	0.0002722	3.778	2.722	
CKD11	0.0003778	0.0003027	3.778	3.027	
CKD13	0.0003707	0.0002467	3.707	2.467	
CKD14	0.0003778	0.0002776	3.778	2.776	
CKD15	0.0003564	0.0002216	3.564	2.216	
CKD18	0.000385	0.0003061	3.85	3.061	
CKD20	0.0004064	0.0002473	4.064	2.473	
CKD21	0.0003778	0.0002268	3.778	2.268	
CKD23	0.0003778	0.0002918	3.778	2.918	
CKD24	0.000385	0.0002161	3.85	2.161	
CKD25	0.0003992	0.0003106	3.992	3.106	
CKD26	0.0003992	0.0002028	3.992	2.028	
CKD27	0.000385	0.0002449	3.85	2.449	
CKD31	0.0004206	0.0002472	4.206	2.472	
CKD32	0.0003707	0.0002878	3.707	2.878	
CKD33	0.0003707	0.000247	3.707	2.47	
CKD45	0.0003992	0.0002196	3.992	2.196	
CKD46	0.0003636	0.0002842	3.636	2.842	
CKD49	0.0003707	0.0002911	3.707	2.911	
CKD50	0.000385	0.0003097	3.85	3.097	
CKD51	0.000385	0.0002671	3.85	2.671	
CKD55	0.0003636	0.0002923	3.636	2.923	
CKD59	0.0003992	0.0003236	3.992	3.236	
CKD62	0.0003707	0.0002274	3.707	2.274	
N2	0.0006986	0.000402	6.986		4.02
N3	0.0006612	0.0004371	6.612		4.371
N4	0.0006737	0.0004267	6.737		4.267
N6	0.0006612	0.0005485	6.612		5.485
N7	0.0006737	0.0004971	6.737		4.971
N8	0.0006487	0.0003191	6.487		3.191
N9	0.0006612	0.0004608	6.612		4.608
N10	0.0006737	0.0003733	6.737		3.733
N11	0.0006363	0.0003694	6.363		3.694
N13	0.0006363	0.0005246	6.363		5.246
N14	0.0006862	0.0005385	6.862		5.385
N15	0.0006737	0.0004661	6.737		4.661
N16	0.0006986	0.0003525	6.986		3.525
N17	0.0007236	0.0005162	7.236		5.162
N18	0.0006737	0.0003181	6.737		3.181
N20	0.0006363	0.000404	6.363		4.04
N21	0.0006249	0.0005143	6.249		5.143
N24	0.0007111	0.0004487	7.111		4.487
N25	0.0006249	0.0003754	6.249		3.754

N27	0.0007361	0.0003975	7.361	3.975
N29	0.0006737	0.0005587	6.737	5.587
N31	0.0007111	0.0004724	7.111	4.724
N32	0.0006612	0.0003823	6.612	3.823
N33	0.0006249	0.0003198	6.249	3.198
N35	0.0006612	0.0005522	6.612	5.522
N36	0.0007111	0.000541	7.111	5.41
N38	0.0006612	0.0005501	6.612	5.501

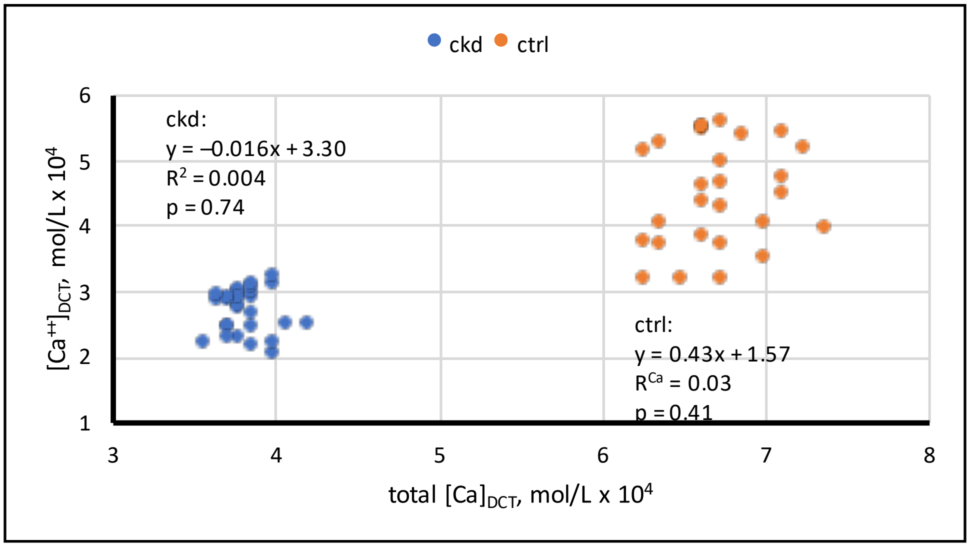

SUMMARY OUTPUT ckd

Regression Statistics	
Multiple R	0.06580683
R Square	0.00433054
Adjusted R Sq	-0.0339644
Standard Erro	0.34711463
Observations	28

ANOVA

	df	*SS*	*MS*	*F*	*Significance F*
Regression	1	0.0136253	0.0136253	0.11308372	0.73935929
Residual	26	3.13270281	0.12048857		
Total	27	3.14632811			

	Coefficients	*Standard Error*	*t Stat*	*P-value*	*Lower 95%*
Intercept	3.29780449	1.80215572	1.82992205	0.07875066	-0.4065796
X Variable 1	-0.1584741	0.47125761	-0.3362792	0.73935929	-1.127158

SUMMARY OUTPUT ctrl

Regression Statistics	
Multiple R	0.16641612
R Square	0.02769432
Adjusted R Sq	-0.0111979
Standard Erro	0.80451733
Observations	27

ANOVA

	df	*SS*	*MS*	*F*	*Significance F*
Regression	1	0.46089158	0.46089158	0.71207865	0.406757
Residual	25	16.1812034	0.64724814		
Total	26	16.642095			

	Coefficients	*Standard Error*	*t Stat*	*P-value*	*Lower 95%*
Intercept	1.56968432	3.43935949	0.45638856	0.65205091	-5.5138091
X Variable 1	0.43206806	0.51202148	0.84384753	0.406757	-0.6224599

Upper 95%	*Lower 95.0%*	*Upper 95.0%*
7.00218863	-0.4065796	7.00218863
0.81020974	-1.127158	0.81020974

Upper 95%	*Lower 95.0%*	*Upper 95.0%*
8.65317778	-5.5138091	8.65317778
1.48659604	-0.6224599	1.48659604

code	Tot(P)	Ca+2	tot P x 10^3	pth	ctrl
CKD2	0.00182	0.0002891	1.82	2.891	
CKD4	0.001852	0.0002891	1.852	2.891	
CKD5	0.001218	0.0002973	1.218	2.973	
CKD6	0.001549	0.0002984	1.549	2.984	
CKD7	0.002036	0.0002722	2.036	2.722	
CKD11	0.0008235	0.0003027	0.8235	3.027	
CKD13	0.002375	0.0002467	2.375	2.467	
CKD14	0.001973	0.0002776	1.973	2.776	
CKD15	0.002803	0.0002216	2.803	2.216	
CKD18	0.001003	0.0003061	1.003	3.061	
CKD20	0.002391	0.0002473	2.391	2.473	
CKD21	0.002718	0.0002268	2.718	2.268	
CKD23	0.001624	0.0002918	1.624	2.918	
CKD24	0.002935	0.0002161	2.935	2.161	
CKD25	0.001507	0.0003106	1.507	3.106	
CKD26	0.003251	0.0002028	3.251	2.028	
CKD27	0.002414	0.0002449	2.414	2.449	
CKD31	0.002402	0.0002472	2.402	2.472	
CKD32	0.001489	0.0002878	1.489	2.878	
CKD33	0.002371	0.000247	2.371	2.47	
CKD45	0.002874	0.0002196	2.874	2.196	
CKD46	0.001331	0.0002842	1.331	2.842	
CKD49	0.001242	0.0002911	1.242	2.911	
CKD50	0.0007575	0.0003097	0.7575	3.097	
CKD51	0.002103	0.0002671	2.103	2.671	
CKD55	0.0007141	0.0002923	0.7141	2.923	
CKD59	0.0006192	0.0003236	0.6192	3.236	
CKD62	0.002702	0.0002274	2.702	2.274	
N2	0.001258	0.000402	1.258		4.02
N3	0.001075	0.0004371	1.075		4.371
N4	0.001127	0.0004267	1.127		4.267
N6	0.0005899	0.0005485	0.5899		5.485
N7	0.0008665	0.0004971	0.8665		4.971
N8	0.001751	0.0003191	1.751		3.191
N9	0.0009826	0.0004608	0.9826		4.608
N10	0.001394	0.0003733	1.394		3.733
N11	0.001392	0.0003694	1.392		3.694
N13	0.0006849	0.0005246	0.6849		5.246
N14	0.0007527	0.0005385	0.7527		5.385
N15	0.0009714	0.0004661	0.9714		4.661
N16	0.001539	0.0003525	1.539		3.525
N17	0.0008413	0.0005162	0.8413		5.162
N18	0.001776	0.0003181	1.776		3.181
N20	0.001206	0.000404	1.206		4.04
N21	0.0007058	0.0005143	0.7058		5.143
N24	0.001062	0.0004487	1.062		4.487
N25	0.00135	0.0003754	1.35		3.754

N27	0.001305	0.0003975	1.305	3.975
N29	0.0006108	0.0005587	0.6108	5.587
N31	0.000974	0.0004724	0.974	4.724
N32	0.001335	0.0003823	1.335	3.823
N33	0.001729	0.0003198	1.729	3.198
N35	0.0004466	0.0005522	0.4466	5.522
N36	0.0007627	0.000541	0.7627	5.41
N38	0.0005282	0.0005501	0.5282	5.501

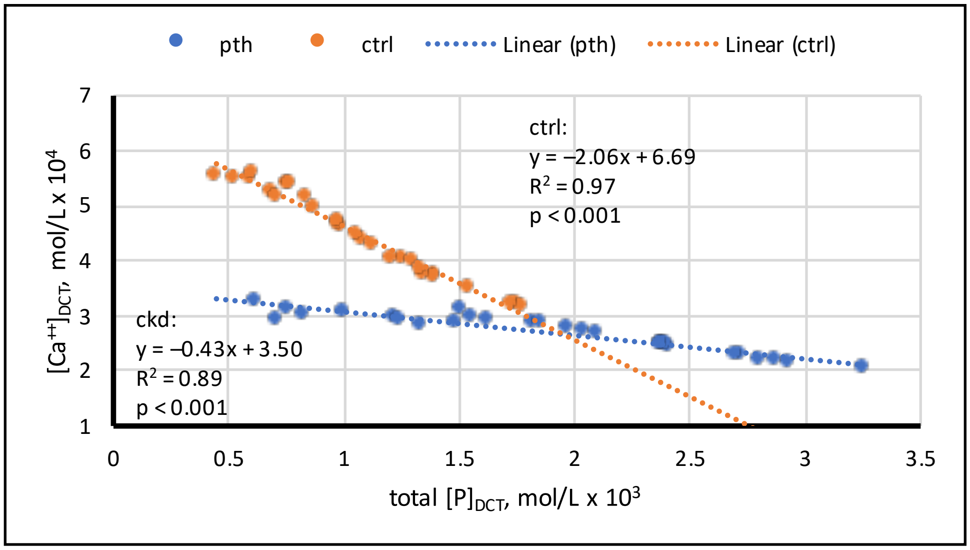

SUMMARY OUTPUT ckd

Regression Statistics	
Multiple R	0.94603641
R Square	0.89498489
Adjusted R Sq	0.89094585
Standard Erro	0.11273045
Observations	28

ANOVA

	df	*SS*	*MS*	*F*	*Significance F*
Regression	1	2.81591612	2.81591612	221.583423	3.0821E-14
Residual	26	0.33041199	0.01270815		
Total	27	3.14632811			

	Coefficients	*Standard Error*	*t Stat*	*P-value*	*Lower 95%*
Intercept	3.50243586	0.05845259	59.9192564	2.1327E-29	3.38228484
X Variable 1	-0.4288915	0.02881236	-14.885678	3.0821E-14	-0.4881162

SUMMARY OUTPUT ctrl

Regression Statistics	
Multiple R	0.98615356
R Square	0.97249883
Adjusted R Sq	0.97139879
Standard Erro	0.13530366

Observations	27

ANOVA

	df	SS	MS	F	Significance F
Regression	1	16.184418	16.184418	884.052396	4.9673E-21
Residual	25	0.457677	0.01830708		
Total	26	16.642095			

	Coefficients	Standard Error	t Stat	P-value	Lower 95%
Intercept	6.68532931	0.07895706	84.6704471	2.8932E-32	6.52271421
X Variable 1	-2.0622783	0.06935987	-29.733019	4.9673E-21	-2.2051276

Upper 95%	*Lower 95.0%*	*Upper 95.0%*
3.62258689	3.38228484	3.62258689
-0.3696669	-0.4881162	-0.3696669

Upper 95%	*Lower 95.0%*	*Upper 95.0%*
6.84794441	6.52271421	6.84794441
-1.919429	-2.2051276	-1.919429

code	Tot(Ca)	CaHPO4	tot Ca x 10^4	ckd	ctrl
CKD2	0.0003778	2.52E-05	3.778	2.52	
CKD4	0.000385	2.56E-05	3.85	2.557	
CKD5	0.0003778	1.74E-05	3.778	1.743	
CKD6	0.000385	2.22E-05	3.85	2.221	
CKD7	0.0003778	2.64E-05	3.778	2.635	
CKD11	0.0003778	1.21E-05	3.778	1.205	
CKD13	0.0003707	2.77E-05	3.707	2.769	
CKD14	0.0003778	2.61E-05	3.778	2.61	
CKD15	0.0003564	2.92E-05	3.564	2.922	
CKD18	0.000385	1.48E-05	3.85	1.481	
CKD20	0.0004064	2.77E-05	4.064	2.766	
CKD21	0.0003778	2.89E-05	3.778	2.889	
CKD23	0.0003778	2.27E-05	3.778	2.273	
CKD24	0.000385	2.96E-05	3.85	2.959	
CKD25	0.0003992	2.24E-05	3.992	2.244	
CKD26	0.0003992	3.06E-05	3.992	3.056	
CKD27	0.000385	2.78E-05	3.85	2.779	
CKD31	0.0004206	2.77E-05	4.206	2.766	
CKD32	0.0003707	2.06E-05	3.707	2.058	
CKD33	0.0003707	2.77E-05	3.707	2.767	
CKD45	0.0003992	2.94E-05	3.992	2.936	
CKD46	0.0003636	1.82E-05	3.636	1.819	
CKD49	0.0003707	1.74E-05	3.707	1.741	
CKD50	0.000385	1.13E-05	3.85	1.133	
CKD51	0.000385	2.66E-05	3.85	2.661	
CKD55	0.0003636	1.01E-05	3.636	1.011	
CKD59	0.0003992	9.70E-06	3.992	0.9696	
CKD62	0.0003707	2.89E-05	3.707	2.885	
N2	0.0006986	2.17E-05	6.986		2.168
N3	0.0006612	2.08E-05	6.612		2.079
N4	0.0006737	2.10E-05	6.737		2.104
N6	0.0006612	1.54E-05	6.612		1.54
N7	0.0006737	1.95E-05	6.737		1.949
N8	0.0006487	2.43E-05	6.487		2.434
N9	0.0006612	2.03E-05	6.612		2.025
N10	0.0006737	2.25E-05	6.737		2.25
N11	0.0006363	2.26E-05	6.363		2.261
N13	0.0006363	1.71E-05	6.363		1.71
N14	0.0006862	1.87E-05	6.862		1.873
N15	0.0006737	2.01E-05	6.737		2.013
N16	0.0006986	2.32E-05	6.986		2.315
N17	0.0007236	1.91E-05	7.236		1.913
N18	0.0006737	2.44E-05	6.737		2.438
N20	0.0006363	2.16E-05	6.363		2.162
N21	0.0006249	1.73E-05	6.249		1.729
N24	0.0007111	2.05E-05	7.111		2.052
N25	0.0006249	2.24E-05	6.249		2.243

N27	0.0007361	2.18E-05	7.361	2.18
N29	0.0006737	1.62E-05	6.737	1.622
N31	0.0007111	2.00E-05	7.111	1.999
N32	0.0006612	2.22E-05	6.612	2.223
N33	0.0006249	2.43E-05	6.249	2.431
N35	0.0006612	1.18E-05	6.612	1.175
N36	0.0007111	1.87E-05	7.111	1.869
N38	0.0006612	1.38E-05	6.612	1.383

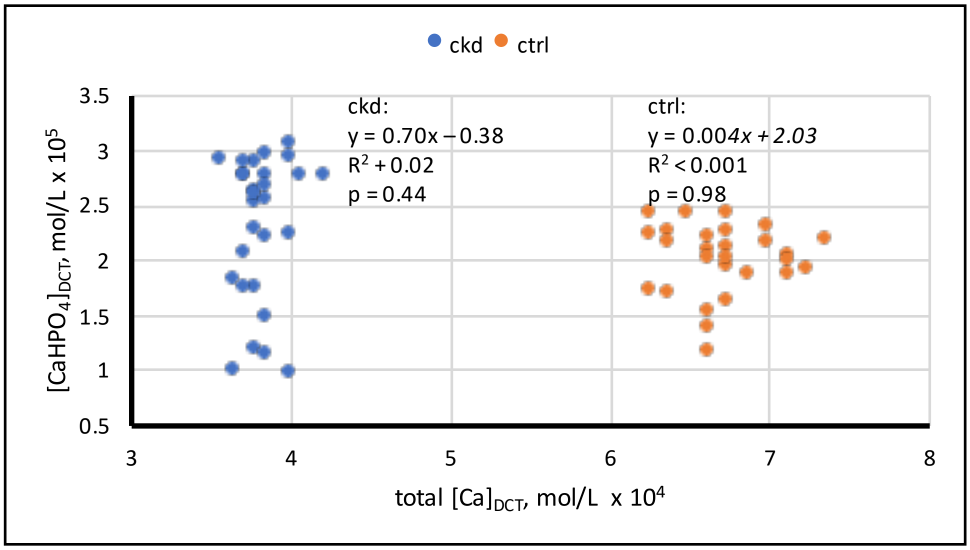

SUMMARY OUTPUT ckd

Regression Statistics	
Multiple R	0.15123823
R Square	0.022873
Adjusted R Sq	-0.0147088
Standard Erro	0.66103629
Observations	28

ANOVA

	df	*SS*	*MS*	*F*	*Significance F*
Regression	1	0.26594762	0.26594762	0.608619	0.44235295
Residual	26	11.3611933	0.43696897		
Total	27	11.6271409			

	Coefficients	*Standard Error*	*t Stat*	*P-value*	*Lower 95%*
Intercept	-0.3765229	3.43197954	-0.1097101	0.91348169	-7.4310579
X Variable 1	0.70013776	0.897451	0.78014037	0.44235295	-1.1445992

SUMMARY OUTPUT ctrl

Regression Statistics	
Multiple R	0.00429057
R Square	1.8409E-05
Adjusted R Sq	-0.0399809
Standard Erro	0.32422422
Observations	27

ANOVA

	df	*SS*	*MS*	*F*	*Significance F*
Regression	1	4.838E-05	4.838E-05	0.00046023	0.98305456
Residual	25	2.62803369	0.10512135		
Total	26	2.62808207			

	Coefficients	*Standard Error*	*t Stat*	*P-value*	*Lower 95%*
Intercept	2.03489065	1.38607786	1.4680926	0.15454931	-0.8197901
X Variable 1	-0.0044268	0.20634704	-0.0214531	0.98305456	-0.4294065

Upper 95%	*Lower 95.0%*	*Upper 95.0%*
6.67801209	-7.4310579	6.67801209
2.54487472	-1.1445992	2.54487472

Upper 95%	*Lower 95.0%*	*Upper 95.0%*
4.88957144	-0.8197901	4.88957144
0.4205529	-0.4294065	0.4205529

code	Tot(P)	CaHPO4	tot P x 10^3	ckd	ctrl
CKD2	0.00182	2.52E-05	1.82	2.52	
CKD4	0.001852	2.56E-05	1.852	2.557	
CKD5	0.001218	1.74E-05	1.218	1.743	
CKD6	0.001549	2.22E-05	1.549	2.221	
CKD7	0.002036	2.64E-05	2.036	2.635	
CKD11	0.0008235	1.21E-05	0.8235	1.205	
CKD13	0.002375	2.77E-05	2.375	2.769	
CKD14	0.001973	2.61E-05	1.973	2.61	
CKD15	0.002803	2.92E-05	2.803	2.922	
CKD18	0.001003	1.48E-05	1.003	1.481	
CKD20	0.002391	2.77E-05	2.391	2.766	
CKD21	0.002718	2.89E-05	2.718	2.889	
CKD23	0.001624	2.27E-05	1.624	2.273	
CKD24	0.002935	2.96E-05	2.935	2.959	
CKD25	0.001507	2.24E-05	1.507	2.244	
CKD26	0.003251	3.06E-05	3.251	3.056	
CKD27	0.002414	2.78E-05	2.414	2.779	
CKD31	0.002402	2.77E-05	2.402	2.766	
CKD32	0.001489	2.06E-05	1.489	2.058	
CKD33	0.002371	2.77E-05	2.371	2.767	
CKD45	0.002874	2.94E-05	2.874	2.936	
CKD46	0.001331	1.82E-05	1.331	1.819	
CKD49	0.001242	1.74E-05	1.242	1.741	
CKD50	0.0007575	1.13E-05	0.7575	1.133	
CKD51	0.002103	2.66E-05	2.103	2.661	
CKD55	0.0007141	1.01E-05	0.7141	1.011	
CKD59	0.0006192	9.70E-06	0.6192	0.9696	
CKD62	0.002702	2.89E-05	2.702	2.885	
N2	0.001258	2.17E-05	1.258		2.168
N3	0.001075	2.08E-05	1.075		2.079
N4	0.001127	2.10E-05	1.127		2.104
N6	0.0005899	1.54E-05	0.5899		1.54
N7	0.0008665	1.95E-05	0.8665		1.949
N8	0.001751	2.43E-05	1.751		2.434
N9	0.0009826	2.03E-05	0.9826		2.025
N10	0.001394	2.25E-05	1.394		2.25
N11	0.001392	2.26E-05	1.392		2.261
N13	0.0006849	1.71E-05	0.6849		1.71
N14	0.0007527	1.87E-05	0.7527		1.873
N15	0.0009714	2.01E-05	0.9714		2.013
N16	0.001539	2.32E-05	1.539		2.315
N17	0.0008413	1.91E-05	0.8413		1.913
N18	0.001776	2.44E-05	1.776		2.438
N20	0.001206	2.16E-05	1.206		2.162
N21	0.0007058	1.73E-05	0.7058		1.729
N24	0.001062	2.05E-05	1.062		2.052
N25	0.00135	2.24E-05	1.35		2.243

N27	0.001305	2.18E-05	1.305	2.18
N29	0.0006108	1.62E-05	0.6108	1.622
N31	0.000974	2.00E-05	0.974	1.999
N32	0.001335	2.22E-05	1.335	2.223
N33	0.001729	2.43E-05	1.729	2.431
N35	0.0004466	1.18E-05	0.4466	1.175
N36	0.0007627	1.87E-05	0.7627	1.869
N38	0.0005282	1.38E-05	0.5282	1.383

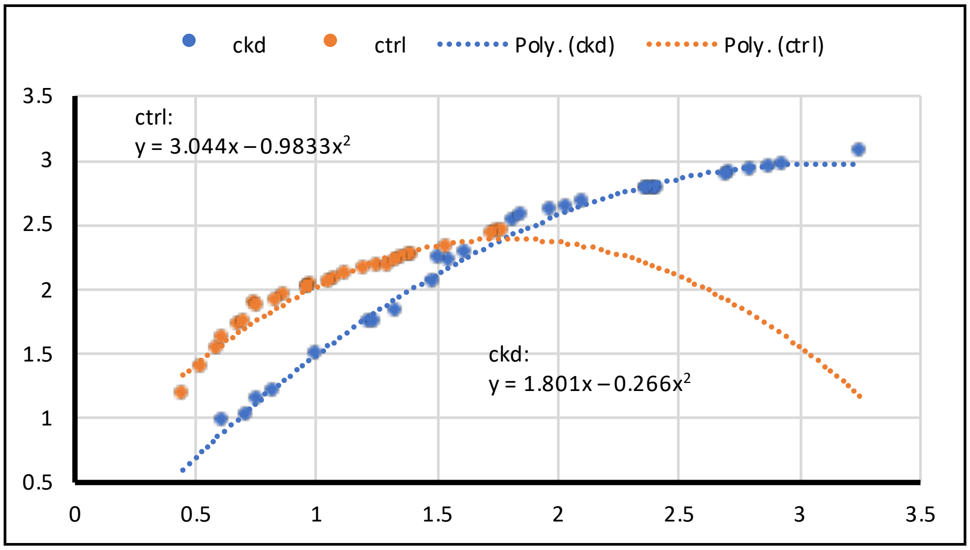

ckd
ctrl
Poly. (ckd)
Poly. (ctrl)
ctrl:
y = 3.044x – 0.9833x2
ckd:
y = 1.801x – 0.266x2
3.5
3
2.5
2
1.5
1
0.5
0
0.5
1
1.5
2
2.5
3
3.5

code	CaHPO4	Ca+2		cahpo4 x 105	ckd	ctrl
CKD2	2.52E-05	0.0002891		2.52	2.891	
CKD4	2.56E-05	0.0002891		2.557	2.891	
CKD5	1.74E-05	0.0002973		1.743	2.973	
CKD6	2.22E-05	0.0002984		2.221	2.984	
CKD7	2.64E-05	0.0002722		2.635	2.722	
CKD11	1.21E-05	0.0003027		1.205	3.027	
CKD13	2.77E-05	0.0002467		2.769	2.467	
CKD14	2.61E-05	0.0002776		2.61	2.776	
CKD15	2.92E-05	0.0002216		2.922	2.216	
CKD18	1.48E-05	0.0003061		1.481	3.061	
CKD20	2.77E-05	0.0002473		2.766	2.473	
CKD21	2.89E-05	0.0002268		2.889	2.268	
CKD23	2.27E-05	0.0002918		2.273	2.918	
CKD24	2.96E-05	0.0002161		2.959	2.161	
CKD25	2.24E-05	0.0003106		2.244	3.106	
CKD26	3.06E-05	0.0002028		3.056	2.028	
CKD27	2.78E-05	0.0002449		2.779	2.449	
CKD31	2.77E-05	0.0002472		2.766	2.472	
CKD32	2.06E-05	0.0002878		2.058	2.878	
CKD33	2.77E-05	0.000247		2.767	2.47	
CKD45	2.94E-05	0.0002196		2.936	2.196	
CKD46	1.82E-05	0.0002842		1.819	2.842	
CKD49	1.74E-05	0.0002911		1.741	2.911	
CKD50	1.13E-05	0.0003097		1.133	3.097	
CKD51	2.66E-05	0.0002671		2.661	2.671	
CKD55	1.01E-05	0.0002923		1.011	2.923	
CKD59	9.70E-06	0.0003236		0.9696	3.236	
CKD62	2.89E-05	0.0002274		2.885	2.274	
N2	2.17E-05	0.000402		2.168		4.02
N3	2.08E-05	0.0004371		2.079		4.371
N4	2.10E-05	0.0004267		2.104		4.267
N6	1.54E-05	0.0005485		1.54		5.485
N7	1.95E-05	0.0004971		1.949		4.971
N8	2.43E-05	0.0003191		2.434		3.191
N9	2.03E-05	0.0004608		2.025		4.608
N10	2.25E-05	0.0003733		2.25		3.733
N11	2.26E-05	0.0003694		2.261		3.694
N13	1.71E-05	0.0005246		1.71		5.246
N14	1.87E-05	0.0005385		1.873		5.385
N15	2.01E-05	0.0004661		2.013		4.661
N16	2.32E-05	0.0003525		2.315		3.525
N17	1.91E-05	0.0005162		1.913		5.162
N18	2.44E-05	0.0003181		2.438		3.181
N20	2.16E-05	0.000404		2.162		4.04
N21	1.73E-05	0.0005143		1.729		5.143
N24	2.05E-05	0.0004487		2.052		4.487
N25	2.24E-05	0.0003754		2.243		3.754

N27	2.18E-05	0.0003975	2.18	3.975
N29	1.62E-05	0.0005587	1.622	5.587
N31	2.00E-05	0.0004724	1.999	4.724
N32	2.22E-05	0.0003823	2.223	3.823
N33	2.43E-05	0.0003198	2.431	3.198
N35	1.18E-05	0.0005522	1.175	5.522
N36	1.87E-05	0.000541	1.869	5.41
N38	1.38E-05	0.0005501	1.383	5.501

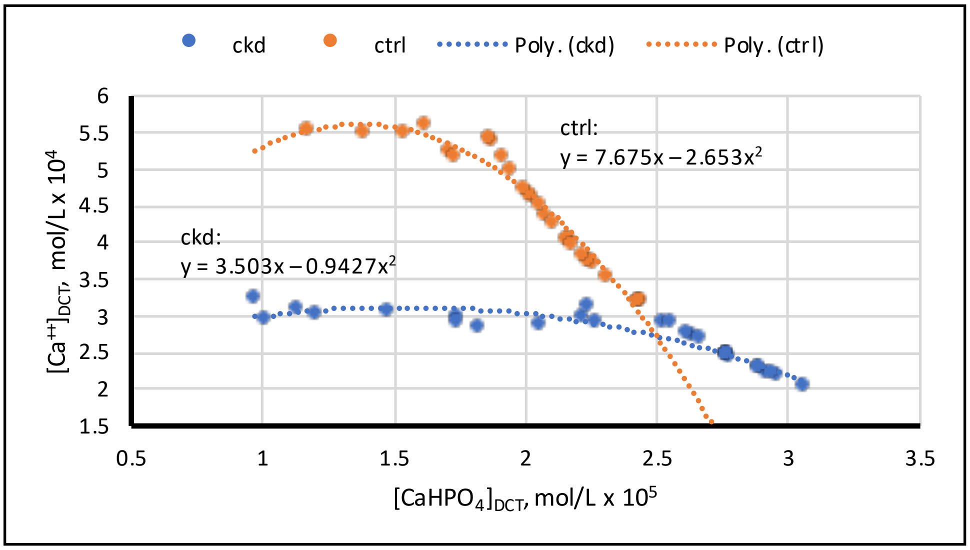

ckd
ctrl
Poly. (ckd)
Poly. (ctrl)
ctrl:
y = 7.675x – 2.653x2
ckd:
y = 3.503x – 0.9427x2
[Ca++]DCT, mol/L x 104
[CaHPO4]DCT, mol/L x 105
6
5.5
5
4.5
4
3.5
3
2.5
2
1.5
0.5
1
1.5
2
2.5
3
3.5

code	CaCitric	Ca+2	Cacit x 10^5	ckd	ctrl
CKD2	3.11E-05	0.0002891	3.113	2.891	
CKD4	3.11E-05	0.0002891	3.113	2.891	
CKD5	3.15E-05	0.0002973	3.154	2.973	
CKD6	3.17E-05	0.0002984	3.166	2.984	
CKD7	3.01E-05	0.0002722	3.012	2.722	
CKD11	3.18E-05	0.0003027	3.18	3.027	
CKD13	2.85E-05	0.0002467	2.849	2.467	
CKD14	3.04E-05	0.0002776	3.044	2.776	
CKD15	2.68E-05	0.0002216	2.675	2.216	
CKD18	3.20E-05	0.0003061	3.202	3.061	
CKD20	2.85E-05	0.0002473	2.853	2.473	
CKD21	2.71E-05	0.0002268	2.712	2.268	
CKD23	3.13E-05	0.0002918	3.125	2.918	
CKD24	2.64E-05	0.0002161	2.635	2.161	
CKD25	3.23E-05	0.0003106	3.231	3.106	
CKD26	2.53E-05	0.0002028	2.534	2.028	
CKD27	2.84E-05	0.0002449	2.836	2.449	
CKD31	2.85E-05	0.0002472	2.852	2.472	
CKD32	3.10E-05	0.0002878	3.101	2.878	
CKD33	2.85E-05	0.000247	2.851	2.47	
CKD45	2.66E-05	0.0002196	2.66	2.196	
CKD46	3.08E-05	0.0002842	3.076	2.842	
CKD49	3.12E-05	0.0002911	3.118	2.911	
CKD50	3.22E-05	0.0003097	3.217	3.097	
CKD51	2.98E-05	0.0002671	2.98	2.671	
CKD55	3.12E-05	0.0002923	3.118	2.923	
CKD59	3.29E-05	0.0003236	3.294	3.236	
CKD62	2.72E-05	0.0002274	2.716	2.274	
N2	3.68E-05	0.000402	3.676		4.02
N3	3.82E-05	0.0004371	3.82		4.371
N4	3.78E-05	0.0004267	3.779		4.267
N6	4.21E-05	0.0005485	4.205		5.485
N7	4.04E-05	0.0004971	4.04		4.971
N8	3.28E-05	0.0003191	3.279		3.191
N9	3.91E-05	0.0004608	3.911		4.608
N10	3.55E-05	0.0003733	3.548		3.733
N11	3.53E-05	0.0003694	3.531		3.694
N13	4.13E-05	0.0005246	4.13		5.246
N14	4.18E-05	0.0005385	4.175		5.385
N15	3.93E-05	0.0004661	3.93		4.661
N16	3.45E-05	0.0003525	3.449		3.525
N17	4.10E-05	0.0005162	4.104		5.162
N18	3.27E-05	0.0003181	3.274		3.181
N20	3.69E-05	0.000404	3.685		4.04
N21	4.10E-05	0.0005143	4.096		5.143
N24	3.87E-05	0.0004487	3.865		4.487
N25	3.56E-05	0.0003754	3.557		3.754

N27	3.66E-05	0.0003975	3.657	3.975
N29	4.24E-05	0.0005587	4.236	5.587
N31	3.95E-05	0.0004724	3.953	4.724
N32	3.59E-05	0.0003823	3.59	3.823
N33	3.28E-05	0.0003198	3.283	3.198
N35	4.22E-05	0.0005522	4.215	5.522
N36	4.18E-05	0.000541	4.183	5.41
N38	4.21E-05	0.0005501	4.209	5.501

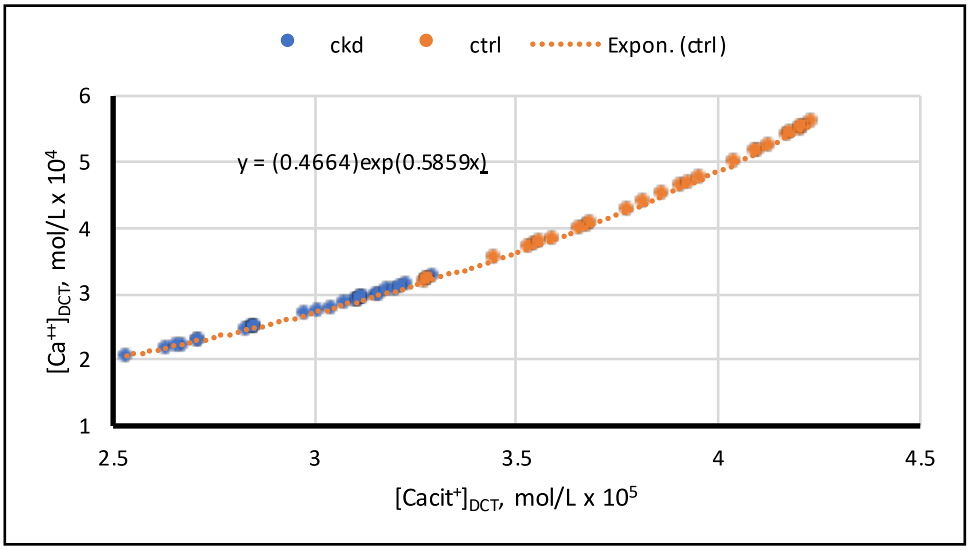

ckd
ctrl
Expon. (ctrl)
y = (0.4664)exp(0.5859x)
[Ca++]DCT, mol/L x 10^4
[Cacit+]DCT, mol/L x 10^5
6
5
4
3
2
1
2.5
3
3.5
4
4.5

code	CaOxalic	Ca+2	Caox x 10^7	CKD	CTRL
CKD2	6.64E-07	0.0002891	6.644	2.891	
CKD4	6.64E-07	0.0002891	6.641	2.891	
CKD5	6.82E-07	0.0002973	6.823	2.973	
CKD6	6.85E-07	0.0002984	6.848	2.984	
CKD7	6.28E-07	0.0002722	6.28	2.722	
CKD11	6.94E-07	0.0003027	6.944	3.027	
CKD13	5.73E-07	0.0002467	5.73	2.467	
CKD14	6.40E-07	0.0002776	6.395	2.776	
CKD15	5.18E-07	0.0002216	5.176	2.216	
CKD18	7.01E-07	0.0003061	7.012	3.061	
CKD20	5.74E-07	0.0002473	5.742	2.473	
CKD21	5.29E-07	0.0002268	5.291	2.268	
CKD23	6.70E-07	0.0002918	6.697	2.918	
CKD24	5.05E-07	0.0002161	5.054	2.161	
CKD25	7.09E-07	0.0003106	7.09	3.106	
CKD26	4.75E-07	0.0002028	4.754	2.028	
CKD27	5.69E-07	0.0002449	5.688	2.449	
CKD31	5.74E-07	0.0002472	5.738	2.472	
CKD32	6.62E-07	0.0002878	6.618	2.878	
CKD33	5.74E-07	0.000247	5.736	2.47	
CKD45	5.13E-07	0.0002196	5.129	2.196	
CKD46	6.54E-07	0.0002842	6.543	2.842	
CKD49	6.69E-07	0.0002911	6.694	2.911	
CKD50	7.09E-07	0.0003097	7.085	3.097	
CKD51	6.17E-07	0.0002671	6.172	2.671	
CKD55	6.73E-07	0.0002923	6.728	2.923	
CKD59	7.38E-07	0.0003236	7.384	3.236	
CKD62	5.30E-07	0.0002274	5.304	2.274	
N2	8.94E-07	0.000402	8.942		4.02
N3	9.63E-07	0.0004371	9.627		4.371
N4	9.43E-07	0.0004267	9.427		4.267
N6	1.17E-06	0.0005485	11.71		5.485
N7	1.08E-06	0.0004971	10.77		4.971
N8	7.27E-07	0.0003191	7.266		3.191
N9	1.01E-06	0.0004608	10.08		4.608
N10	8.37E-07	0.0003733	8.372		3.733
N11	8.30E-07	0.0003694	8.295		3.694
N13	1.13E-06	0.0005246	11.27		5.246
N14	1.15E-06	0.0005385	11.53		5.385
N15	1.02E-06	0.0004661	10.18		4.661
N16	7.95E-07	0.0003525	7.95		3.525
N17	1.11E-06	0.0005162	11.12		5.162
N18	7.25E-07	0.0003181	7.245		3.181
N20	8.98E-07	0.000404	8.983		4.04
N21	1.11E-06	0.0005143	11.08		5.143
N24	9.85E-07	0.0004487	9.851		4.487
N25	8.41E-07	0.0003754	8.411		3.754

N27	8.85E-07	0.0003975	8.853	3.975
N29	1.19E-06	0.0005587	11.89	5.587
N31	1.03E-06	0.0004724	10.3	4.724
N32	8.55E-07	0.0003823	8.553	3.823
N33	7.28E-07	0.0003198	7.281	3.198
N35	1.18E-06	0.0005522	11.78	5.522
N36	1.16E-06	0.000541	11.57	5.41
N38	1.17E-06	0.0005501	11.74	5.501

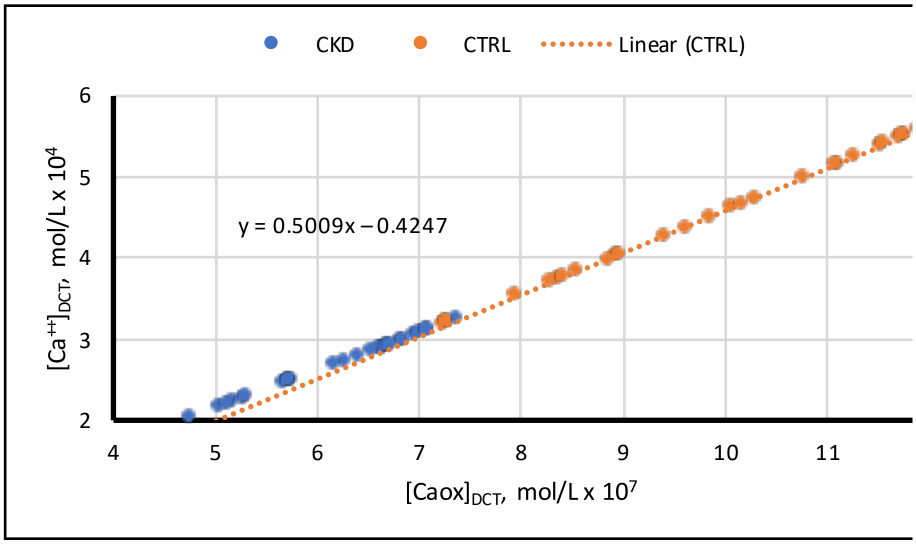
CKD
CTRL
Linear (CTRL)
y = 0.5009x – 0.4247
[Ca++]DCT, mol/L x 10^4
[Caox]DCT, mol/L x 10^7
6
5
4
3
2
4
5
6
7
8
9
10
11

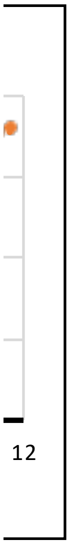
12

code	CaHCO3+	Ca+2		CaHCO3+ x 10^5	CKD	CTRL
CKD2	1.40E-05	0.0002891		1.398	2.891	
CKD4	1.40E-05	0.0002891		1.398	2.891	
CKD5	1.44E-05	0.0002973		1.443	2.973	
CKD6	1.45E-05	0.0002984		1.446	2.984	
CKD7	1.32E-05	0.0002722		1.315	2.722	
CKD11	1.47E-05	0.0003027		1.473	3.027	
CKD13	1.19E-05	0.0002467		1.19	2.467	
CKD14	1.34E-05	0.0002776		1.341	2.776	
CKD15	1.07E-05	0.0002216		1.066	2.216	
CKD18	1.49E-05	0.0003061		1.488	3.061	
CKD20	1.19E-05	0.0002473		1.192	2.473	
CKD21	1.09E-05	0.0002268		1.091	2.268	
CKD23	1.41E-05	0.0002918		1.412	2.918	
CKD24	1.04E-05	0.0002161		1.039	2.161	
CKD25	1.50E-05	0.0003106		1.504	3.106	
CKD26	9.72E-06	0.0002028		0.9724	2.028	
CKD27	1.18E-05	0.0002449		1.18	2.449	
CKD31	1.19E-05	0.0002472		1.192	2.472	
CKD32	1.39E-05	0.0002878		1.394	2.878	
CKD33	1.19E-05	0.000247		1.191	2.47	
CKD45	1.06E-05	0.0002196		1.055	2.196	
CKD46	1.38E-05	0.0002842		1.378	2.842	
CKD49	1.41E-05	0.0002911		1.413	2.911	
CKD50	1.51E-05	0.0003097		1.506	3.097	
CKD51	1.29E-05	0.0002671		1.29	2.671	
CKD55	1.42E-05	0.0002923		1.423	2.923	
CKD59	1.58E-05	0.0003236		1.577	3.236	
CKD62	1.09E-05	0.0002274		1.094	2.274	
N2	1.95E-05	0.000402		1.949		4.02
N3	2.12E-05	0.0004371		2.119		4.371
N4	2.07E-05	0.0004267		2.069		4.267
N6	2.66E-05	0.0005485		2.661		5.485
N7	2.41E-05	0.0004971		2.411		4.971
N8	1.54E-05	0.0003191		1.544		3.191
N9	2.24E-05	0.0004608		2.235		4.608
N10	1.81E-05	0.0003733		1.809		3.733
N11	1.79E-05	0.0003694		1.79		3.694
N13	2.54E-05	0.0005246		2.544		5.246
N14	2.61E-05	0.0005385		2.611		5.385
N15	2.26E-05	0.0004661		2.261		4.661
N16	1.71E-05	0.0003525		1.707		3.525
N17	2.50E-05	0.0005162		2.503		5.162
N18	1.54E-05	0.0003181		1.539		3.181
N20	1.96E-05	0.000404		1.959		4.04
N21	2.50E-05	0.0005143		2.495		5.143
N24	2.18E-05	0.0004487		2.176		4.487
N25	1.82E-05	0.0003754		1.819		3.754

N27	1.93E-05	0.0003975	1.927	3.975
N29	2.71E-05	0.0005587	2.709	5.587
N31	2.29E-05	0.0004724	2.291	4.724
N32	1.85E-05	0.0003823	1.853	3.823
N33	1.55E-05	0.0003198	1.548	3.198
N35	2.68E-05	0.0005522	2.681	5.522
N36	2.62E-05	0.000541	2.623	5.41
N38	2.67E-05	0.0005501	2.67	5.501

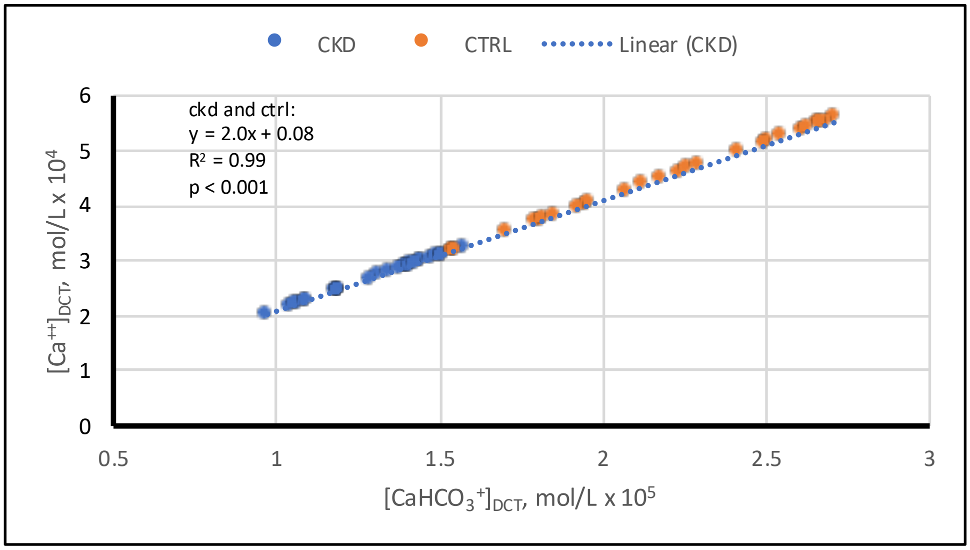

SUMMARY OUTPUT

Regression Statistics	
Multiple R	0.99990318
R Square	0.99980638
Adjusted R Sq	0.99979893
Standard Erro	0.00484054
Observations	28

ANOVA

	df	*SS*	*MS*	*F*	*Significance F*
Regression	1	3.14571891	3.14571891	134255.489	8.3322E-50
Residual	26	0.0006092	2.3431E-05		
Total	27	3.14632811			

	Coefficients	*Standard Error*	*t Stat*	*P-value*	*Lower 95%*
Intercept	0.08094458	0.00718503	11.2657282	1.6763E-11	0.06617554
X Variable 1	2.00525904	0.00547274	366.408909	8.3322E-50	1.99400967

SUMMARY OUTPUT

Regression Statistics	
Multiple R	0.99999741
R Square	0.99999482
Adjusted R Sq	0.99999462
Standard Erro	0.00185607
Observations	27

ANOVA

	df	*SS*	*MS*	*F*	*Significance F*

Regression	1	16.6420088	16.6420088	4830782.67	1.3263E-67
Residual	25	8.6125E-05	3.445E-06		
Total	26	16.642095			

	Coefficients	*Standard Error*	*t Stat*	*P-value*	*Lower 95%*
Intercept	0.01513399	0.00205767	7.35490649	1.0504E-07	0.01089614
X Variable 1	2.05554215	0.00093523	2197.90415	1.3263E-67	2.05361601

Upper 95%	*Lower 95.0%*	*Upper 95.0%*
0.09571361	0.06617554	0.09571361
2.01650841	1.99400967	2.01650841

Upper 95%	*Lower 95.0%*	*Upper 95.0%*
0.01937185	0.01089614	0.01937185
2.05746829	2.05361601	2.05746829

code	CaSO4	Ca+2	CaSO4 x 10^6	CKD	CTRL
CKD2	7.16E-06	0.0002891	7.161	2.891	
CKD4	7.16E-06	0.0002891	7.156	2.891	
CKD5	7.40E-06	0.0002973	7.401	2.973	
CKD6	7.41E-06	0.0002984	7.412	2.984	
CKD7	6.73E-06	0.0002722	6.732	2.722	
CKD11	7.56E-06	0.0003027	7.564	3.027	
CKD13	6.09E-06	0.0002467	6.091	2.467	
CKD14	6.87E-06	0.0002776	6.867	2.776	
CKD15	5.45E-06	0.0002216	5.454	2.216	
CKD18	7.63E-06	0.0003061	7.634	3.061	
CKD20	6.11E-06	0.0002473	6.105	2.473	
CKD21	5.59E-06	0.0002268	5.585	2.268	
CKD23	7.23E-06	0.0002918	7.232	2.918	
CKD24	5.31E-06	0.0002161	5.314	2.161	
CKD25	7.70E-06	0.0003106	7.697	3.106	
CKD26	4.97E-06	0.0002028	4.973	2.028	
CKD27	6.04E-06	0.0002449	6.043	2.449	
CKD31	6.10E-06	0.0002472	6.101	2.472	
CKD32	7.15E-06	0.0002878	7.148	2.878	
CKD33	6.10E-06	0.000247	6.098	2.47	
CKD45	5.40E-06	0.0002196	5.4	2.196	
CKD46	7.07E-06	0.0002842	7.068	2.842	
CKD49	7.25E-06	0.0002911	7.249	2.911	
CKD50	7.73E-06	0.0003097	7.734	3.097	
CKD51	6.61E-06	0.0002671	6.605	2.671	
CKD55	7.32E-06	0.0002923	7.318	2.923	
CKD59	8.10E-06	0.0003236	8.096	3.236	
CKD62	5.60E-06	0.0002274	5.6	2.274	
N2	9.94E-06	0.000402	9.94		4.02
N3	1.08E-05	0.0004371	10.8		4.371
N4	1.05E-05	0.0004267	10.54		4.267
N6	1.35E-05	0.0005485	13.49		5.485
N7	1.23E-05	0.0004971	12.25		4.971
N8	7.90E-06	0.0003191	7.898		3.191
N9	1.14E-05	0.0004608	11.37		4.608
N10	9.24E-06	0.0003733	9.237		3.733
N11	9.14E-06	0.0003694	9.143		3.694
N13	1.29E-05	0.0005246	12.91		5.246
N14	1.32E-05	0.0005385	13.24		5.385
N15	1.15E-05	0.0004661	11.5		4.661
N16	8.72E-06	0.0003525	8.724		3.525
N17	1.27E-05	0.0005162	12.7		5.162
N18	7.87E-06	0.0003181	7.873		3.181
N20	9.99E-06	0.000404	9.991		4.04
N21	1.27E-05	0.0005143	12.66		5.143
N24	1.11E-05	0.0004487	11.08		4.487
N25	9.29E-06	0.0003754	9.286		3.754

N27	9.83E-06	0.0003975	9.83	3.975
N29	1.37E-05	0.0005587	13.72	5.587
N31	1.17E-05	0.0004724	11.65	4.724
N32	9.46E-06	0.0003823	9.46	3.823
N33	7.92E-06	0.0003198	7.916	3.198
N35	1.36E-05	0.0005522	13.6	5.522
N36	1.33E-05	0.000541	13.3	5.41
N38	1.35E-05	0.0005501	13.53	5.501

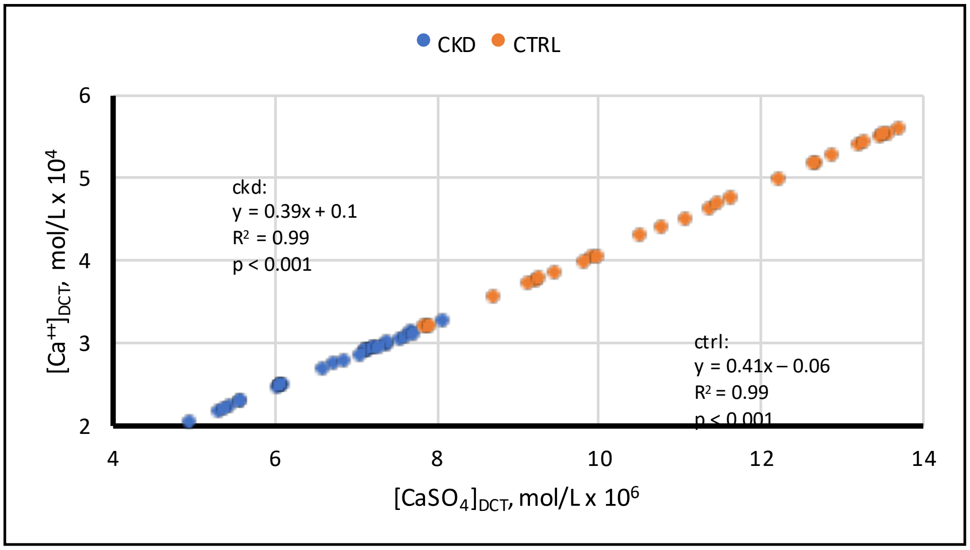

SUMMARY OUTPUT

Regression Statistics	
Multiple R	0.99976533
R Square	0.99953071
Adjusted R Sq	0.99951266
Standard Erro	0.00753591
Observations	28

ANOVA

	df	*SS*	*MS*	*F*	*Significance F*
Regression	1	3.14485157	3.14485157	55376.9213	8.3005E-45
Residual	26	0.00147654	5.679E-05		
Total	27	3.14632811			

	Coefficients	*Standard Error*	*t Stat*	*P-value*	*Lower 95%*
Intercept	0.10043303	0.01110526	9.04373368	1.6466E-09	0.07760584
X Variable 1	0.38840533	0.00165052	235.323015	8.3005E-45	0.38501264

SUMMARY OUTPUT

Regression Statistics	
Multiple R	0.99998437
R Square	0.99996875
Adjusted R Sq	0.9999675

Standard Erro	0.00456101
Observations	27

ANOVA

	df	*SS*	*MS*	*F*	*Significance F*
Regression	1	16.6415749	16.6415749	799966.534	7.6616E-58
Residual	25	0.00052007	2.0803E-05		
Total	26	16.642095			

	Coefficients	*Standard Error*	*t Stat*	*P-value*	*Lower 95%*
Intercept	-0.0633549	0.00514293	-12.318817	4.0859E-12	-0.0739469
X Variable 1	0.41115241	0.00045969	894.408483	7.6616E-58	0.41020566

Upper 95%	*Lower 95.0%*	*Upper 95.0%*
0.12326023	0.07760584	0.12326023
0.39179802	0.38501264	0.39179802

Upper 95%	*Lower 95.0%*	*Upper 95.0%*
-0.0527628	-0.0739469	-0.0527628
0.41209916	0.41020566	0.41209916

code	Tot(Ca)	tot ca x 10^4	ckd	ctrl
CKD2	0.0003778	3.778	158	
CKD4	0.000385	3.85	41	
CKD5	0.0003778	3.778	59	
CKD6	0.000385	3.85	54	
CKD7	0.0003778	3.778	129	
CKD11	0.0003778	3.778	50	
CKD13	0.0003707	3.707	56	
CKD14	0.0003778	3.778	145	
CKD15	0.0003564	3.564	156	
CKD18	0.000385	3.85	67	
CKD20	0.0004064	4.064	182	
CKD21	0.0003778	3.778	126	
CKD23	0.0003778	3.778	63	
CKD24	0.000385	3.85	103	
CKD25	0.0003992	3.992	42	
CKD26	0.0003992	3.992	69	
CKD27	0.000385	3.85	72	
CKD31	0.0004206	4.206	31	
CKD32	0.0003707	3.707	91	
CKD33	0.0003707	3.707	54	
CKD45	0.0003992	3.992	127	
CKD46	0.0003636	3.636	39	
CKD49	0.0003707	3.707	48	
CKD50	0.000385	3.85	48	
CKD51	0.000385	3.85	73	
CKD55	0.0003636	3.636	32	
CKD59	0.0003992	3.992	28	
CKD62	0.0003707	3.707	178	
N2	0.0006986	6.986		21
N3	0.0006612	6.612		44
N4	0.0006737	6.737		45
N6	0.0006612	6.612		31
N7	0.0006737	6.737		18
N8	0.0006487	6.487		24
N9	0.0006612	6.612		36
N10	0.0006737	6.737		22
N11	0.0006363	6.363		60
N13	0.0006363	6.363		28
N14	0.0006862	6.862		20
N15	0.0006737	6.737		34
N16	0.0006986	6.986		17
N17	0.0007236	7.236		29
N18	0.0006737	6.737		25
N20	0.0006363	6.363		19
N21	0.0006249	6.249		26
N24	0.0007111	7.111		21
N25	0.0006249	6.249		41

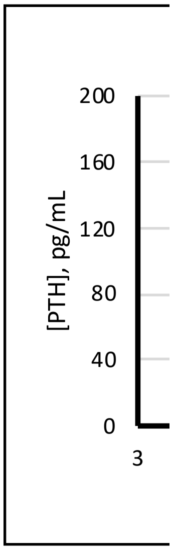

SUMMARY OU

Regression
Multiple R
R Square
Adjusted R Sq
Standard Erro
Observations

ANOVA

Regression
Residual
Total

Intercept
X Variable 1

SUMMARY OU

Regression
Multiple R
R Square
Adjusted R Sq
Standard Erro
Observations

N27	0.0007361	7.361	16	
N29	0.0006737	6.737	23	ANOVA
N31	0.0007111	7.111	19	
N32	0.0006612	6.612	24	Regression
N33	0.0006249	6.249	65	Residual
N35	0.0006612	6.612	24	Total
N36	0.0007111	7.111	25	
N38	0.0006612	6.612	26	
				Intercept
				X Variable 1

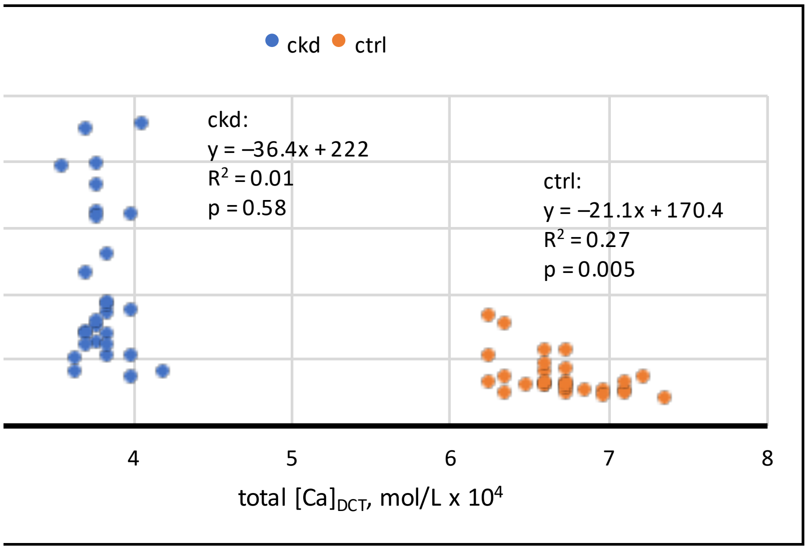

TPUT ckd

Statistics
0.1084377
0.01175874
-0.0262505
48.2692304
28

df	*SS*	*MS*	*F*	*Significance F*
1	720.794931	720.794931	0.30936486	0.5828259
26	60577.8836	2329.9186		
27	61298.6786			

Coefficients	*Standard Error*	*t Stat*	*P-value*	*Lower 95%*	*Upper 95%*	*Lower 95.0%*
222.188442	250.605019	0.88660811	0.38341765	-292.93755	737.314436	-292.93755
-36.449478	65.5323621	-0.5562058	0.5828259	-171.15318	98.2542217	-171.15318

TPUT ctrl

Statistics
0.52245748
0.27296181
0.24388029
10.8043163
27

df	*SS*	*MS*	*F*	*Significance F*
1	1095.66872	1095.66872	9.38608932	0.00517895
25	2918.33128	116.733251		
26	4014			

Coefficients	*Standard Error*	*t Stat*	*P-value*	*Lower 95%*	*Upper 95%*	*Lower 95.0%*
170.364805	46.1890955	3.68842046	0.00109768	75.2365821	265.493028	75.2365821
-21.066501	6.87622484	-3.0636725	0.00517895	-35.228351	-6.9046509	-35.228351

Upper 95.0%
737.314436
98.2542217

Upper 95.0%
265.493028
-6.9046509

code	Ca+2	Ca++ x 10^4	ckd	ctrl
CKD2	0.0002891	2.891	158	
CKD4	0.0002891	2.891	41	
CKD5	0.0002973	2.973	59	
CKD6	0.0002984	2.984	54	
CKD7	0.0002722	2.722	129	
CKD11	0.0003027	3.027	50	
CKD13	0.0002467	2.467	56	
CKD14	0.0002776	2.776	145	
CKD15	0.0002216	2.216	156	
CKD18	0.0003061	3.061	67	
CKD20	0.0002473	2.473	182	
CKD21	0.0002268	2.268	126	
CKD23	0.0002918	2.918	63	
CKD24	0.0002161	2.161	103	
CKD25	0.0003106	3.106	42	
CKD26	0.0002028	2.028	69	
CKD27	0.0002449	2.449	72	
CKD31	0.0002472	2.472	31	
CKD32	0.0002878	2.878	91	
CKD33	0.000247	2.47	54	
CKD45	0.0002196	2.196	127	
CKD46	0.0002842	2.842	39	
CKD49	0.0002911	2.911	48	
CKD50	0.0003097	3.097	48	
CKD51	0.0002671	2.671	73	
CKD55	0.0002923	2.923	32	
CKD59	0.0003236	3.236	28	
CKD62	0.0002274	2.274	178	
N2	0.000402	4.02		21
N3	0.0004371	4.371		44
N4	0.0004267	4.267		45
N6	0.0005485	5.485		31
N7	0.0004971	4.971		18
N8	0.0003191	3.191		24
N9	0.0004608	4.608		36
N10	0.0003733	3.733		22
N11	0.0003694	3.694		60
N13	0.0005246	5.246		28
N14	0.0005385	5.385		20
N15	0.0004661	4.661		34
N16	0.0003525	3.525		17
N17	0.0005162	5.162		29
N18	0.0003181	3.181		25
N20	0.000404	4.04		19
N21	0.0005143	5.143		26
N24	0.0004487	4.487		21
N25	0.0003754	3.754		41

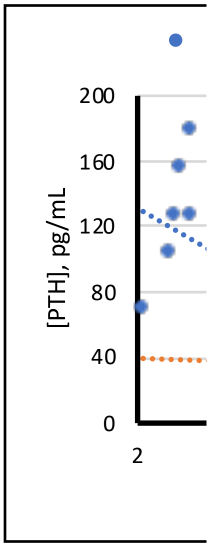

SUMMARY OU

Regression
Multiple R
R Square
Adjusted R Sq
Standard Erro
Observations

ANOVA

Regression
Residual
Total

Intercept
X Variable 1

SUMMARY OU

Regression
Multiple R
R Square
Adjusted R Sq
Standard Erro
Observations

N27	0.0003975	3.975	16	
N29	0.0005587	5.587	23	ANOVA
N31	0.0004724	4.724	19	
N32	0.0003823	3.823	24	Regression
N33	0.0003198	3.198	65	Residual
N35	0.0005522	5.522	24	Total
N36	0.000541	5.41	25	
N38	0.0005501	5.501	26	
				Intercept
				X Variable 1

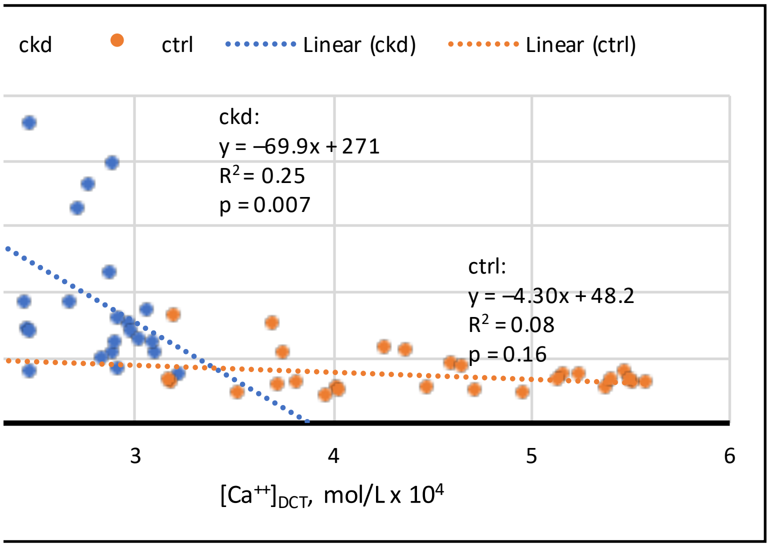

TPUT ckd

Statistics
0.50059897
0.25059933
0.22177622
42.0335356
28

df	*SS*	*MS*	*F*	*Significance F*
1	15361.4075	15361.4075	8.69439097	0.00666481
26	45937.2711	1766.81812		
27	61298.6786			

Coefficients	*Standard Error*	*t Stat*	*P-value*	*Lower 95%*	*Upper 95%*	*Lower 95.0%*
271.005231	64.2892793	4.21540315	0.00026607	138.856724	403.153737	138.856724
-69.873661	23.6970299	-2.9486253	0.00666481	-118.5836	-21.163719	-118.5836

TPUT ctrl

Statistics
0.27715743
0.07681624
0.03988889
12.1748259
27

df	SS	MS	F	Significance F
1	308.340384	308.340384	2.08019905	0.1616327
25	3705.65962	148.226385		
26	4014			

Coefficients	Standard Error	t Stat	P-value	Lower 95%	Upper 95%	Lower 95.0%
48.2364603	13.5416899	3.56207097	0.00151052	20.3468279	76.1260927	20.3468279
-4.304386	2.98441188	-1.4422895	0.1616327	-10.450897	1.84212536	-10.450897

Upper 95.0%
403.153737
-21.163719

Upper 95.0%
76.1260927
1.84212536

code	Ca+2	Ca++ x 10^4	ckd	ctrl	logCa++ x 104
CKD2	0.0002891	2.891	158		0.46104809
CKD4	0.0002891	2.891	41		0.46104809
CKD5	0.0002973	2.973	59		0.47319491
CKD6	0.0002984	2.984	54		0.47479882
CKD7	0.0002722	2.722	129		0.43488812
CKD11	0.0003027	3.027	50		0.48101242
CKD13	0.0002467	2.467	56		0.39216915
CKD14	0.0002776	2.776	145		0.44341946
CKD15	0.0002216	2.216	156		0.34556976
CKD18	0.0003061	3.061	67		0.48586333
CKD20	0.0002473	2.473	182		0.39322412
CKD21	0.0002268	2.268	126		0.35564305
CKD23	0.0002918	2.918	63		0.46508529
CKD24	0.0002161	2.161	103		0.33465477
CKD25	0.0003106	3.106	42		0.49220145
CKD26	0.0002028	2.028	69		0.30706795
CKD27	0.0002449	2.449	72		0.38898879
CKD31	0.0002472	2.472	31		0.39304847
CKD32	0.0002878	2.878	91		0.45909079
CKD33	0.000247	2.47	54		0.39269695
CKD45	0.0002196	2.196	127		0.34163234
CKD46	0.0002842	2.842	39		0.45362407
CKD49	0.0002911	2.911	48		0.46404221
CKD50	0.0003097	3.097	48		0.49094121
CKD51	0.0002671	2.671	73		0.42667389
CKD55	0.0002923	2.923	32		0.46582882
CKD59	0.0003236	3.236	28		0.51000851
CKD62	0.0002274	2.274	178		0.35679046
N2	0.000402	4.02		21	0.60422605
N3	0.0004371	4.371		44	0.64058081
N4	0.0004267	4.267		45	0.63012264
N6	0.0005485	5.485		31	0.73917663
N7	0.0004971	4.971		18	0.69644376
N8	0.0003191	3.191		24	0.5039268
N9	0.0004608	4.608		36	0.66351247
N10	0.0003733	3.733		22	0.57205799
N11	0.0003694	3.694		60	0.56749689
N13	0.0005246	5.246		28	0.71982829
N14	0.0005385	5.385		20	0.73118571
N15	0.0004661	4.661		34	0.6684791
N16	0.0003525	3.525		17	0.54715912
N17	0.0005162	5.162		29	0.712818
N18	0.0003181	3.181		25	0.50256367
N20	0.000404	4.04		19	0.60638137
N21	0.0005143	5.143		26	0.71121652
N24	0.0004487	4.487		21	0.65195607
N25	0.0003754	3.754		41	0.57449427

N27	0.0003975	3.975	16	0.59933713
N29	0.0005587	5.587	23	0.74717867
N31	0.0004724	4.724	19	0.67430989
N32	0.0003823	3.823	24	0.5824043
N33	0.0003198	3.198	65	0.50487846
N35	0.0005522	5.522	24	0.7420964
N36	0.000541	5.41	25	0.73319727
N38	0.0005501	5.501	26	0.74044164

logPTH
2.19865709
1.61278386
1.77085201
1.73239376
2.11058971
1.69897
1.74818803
2.161368
2.1931246
1.8260748
2.26007139
2.10037055
1.79934055
2.01283722
1.62324929
1.83884909
1.8573325
1.49136169
1.95904139
1.73239376
2.10380372
1.59106461
1.68124124
1.68124124
1.86332286
1.50514998
1.44715803
2.25042
1.32221929
1.64345268
1.65321251
1.49136169
1.25527251
1.38021124
1.5563025
1.34242268
1.77815125
1.44715803
1.30103
1.53147892
1.23044892
1.462398
1.39794001
1.2787536
1.41497335
1.32221929
1.61278386

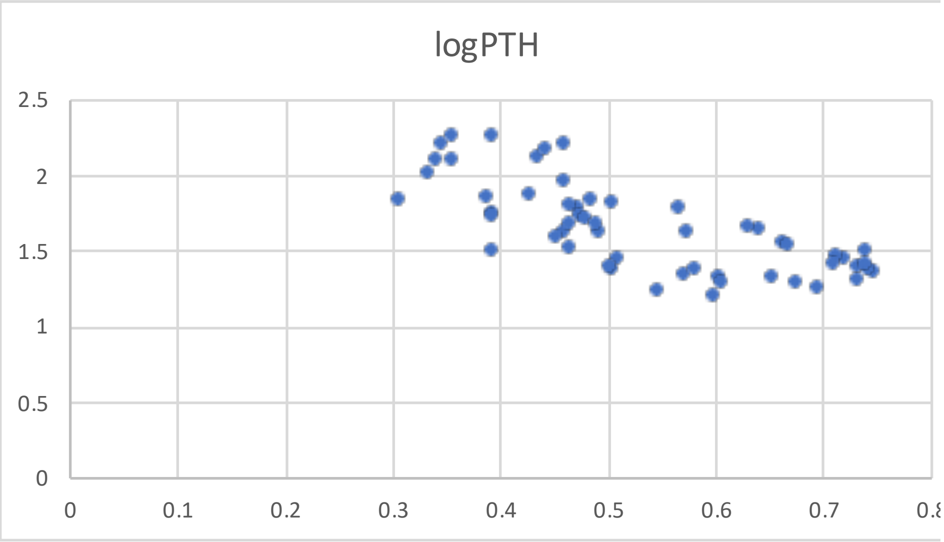

Column1	
Mean	0.53294046
Standard Erro	0.01740821
Median	0.50256367
Mode	0.46104809
Standard Devi	0.12910276
Sample Variar	0.01666752
Kurtosis	-1.1235188
Skewness	0.20873048
Range	0.44011072
Minimum	0.30706795
Maximum	0.74717867
Sum	29.3117252
Count	55

log Ca++ x 104

Column1	
Mean	1.64552531
Standard Erro	0.03975921
Median	1.61278386
Mode	1.38021124
Standard Devi	0.29486219
Sample Variar	0.08694371
Kurtosis	-0.6740973
Skewness	0.54246903
Range	1.05595141
Minimum	1.20411998
Maximum	2.26007139
Sum	90.5038919
Count	55

SUMMARY OUTPUT

Regression Statistics	
Multiple R	0.73551584
R Square	0.54098355
Adjusted R Sq	0.53232287
Standard Erro	0.20164718
Observations	55

ANOVA

	df	*SS*	*MS*	*F*
Regression	1	2.539896341	2.53989634	62.4642727
Residual	53	2.15506401	0.04066159	
Total	54	4.694960351		

1.20411998
1.36172784
1.2787536
1.38021124
1.81291336
1.38021124
1.39794001
1.41497335

	Coefficients	*Standard Error*	*t Stat*	*P-value*
Intercept	2.54079585	0.116493717	21.8105827	3.873E-28
X Variable 1	-1.6798697	0.212549341	-7.9034342	1.5964E-10

8

pth

Significance F
1.5964E-10

logCa++ x 104	mean
0.461048092	0.533
0.461048092	0.533
0.473194909	0.533
0.474798819	0.533
0.434888121	0.533
0.481012421	0.533
0.392169149	0.533
0.443419462	0.533
0.345569756	0.533
0.48586333	0.533
0.393224116	0.533
0.35564305	0.533
0.465085288	0.533
0.334654767	0.533
0.492201451	0.533
0.307067951	0.533
0.388988785	0.533
0.393048466	0.533
0.45909079	0.533
0.392696953	0.533
0.341632336	0.533
0.453624074	0.533
0.464042205	0.533
0.490941205	0.533
0.426673888	0.533
0.465828815	0.533
0.510008513	0.533
0.35679046	0.533
0.604226053	0.533
0.640580806	0.533
0.630122643	0.533
0.739176632	0.533
0.696443763	0.533
0.503926804	0.533
0.66351247	0.533
0.57205799	0.533
0.567496891	0.533
0.719828286	0.533
0.731185708	0.533
0.668479103	0.533
0.547159121	0.533
0.712818	0.533
0.502563669	0.533
0.606381365	0.533
0.711216524	0.533
0.65195607	0.533
0.574494268	0.533

Lower 95%	*Upper 95%*	*Lower 95.0%*	*Upper 95.0%*
2.30713905	2.77445266	2.30713905	2.77445266
-2.1061897	-1.2535497	-2.1061897	-1.2535497

0.599337133	0.533
0.747178671	0.533
0.674309889	0.533
0.582404298	0.533
0.504878459	0.533
0.742096402	0.533
0.733197265	0.533
0.740441645	0.533

SD	standardized log([Ca++} x 104)	logPTH	mean	SD
0.129	-0.557766731	2.19865709	1.646	0.295
0.129	-0.557766731	1.61278386	1.646	0.295
0.129	-0.463605355	1.77085201	1.646	0.295
0.129	-0.451171947	1.73239376	1.646	0.295
0.129	-0.760557203	2.11058971	1.646	0.295
0.129	-0.403004489	1.69897	1.646	0.295
0.129	-1.091712019	1.74818803	1.646	0.295
0.129	-0.694422777	2.161368	1.646	0.295
0.129	-1.452947627	2.1931246	1.646	0.295
0.129	-0.365400546	1.8260748	1.646	0.295
0.129	-1.083533982	2.26007139	1.646	0.295
0.129	-1.374860076	2.10037055	1.646	0.295
0.129	-0.526470639	1.79934055	1.646	0.295
0.129	-1.537559947	2.01283722	1.646	0.295
0.129	-0.316267819	1.62324929	1.646	0.295
0.129	-1.751411235	1.83884909	1.646	0.295
0.129	-1.116366007	1.8573325	1.646	0.295
0.129	-1.084895609	1.49136169	1.646	0.295
0.129	-0.572939615	1.95904139	1.646	0.295
0.129	-1.087620517	1.73239376	1.646	0.295
0.129	-1.483470265	2.10380372	1.646	0.295
0.129	-0.615317259	1.59106461	1.646	0.295
0.129	-0.534556547	1.68124124	1.646	0.295
0.129	-0.326037168	1.68124124	1.646	0.295
0.129	-0.824233426	1.86332286	1.646	0.295
0.129	-0.520706858	1.50514998	1.646	0.295
0.129	-0.178228582	1.44715803	1.646	0.295
0.129	-1.365965424	2.25042	1.646	0.295
0.129	0.552139946	1.32221929	1.646	0.295
0.129	0.83395974	1.64345268	1.646	0.295
0.129	0.752888704	1.65321251	1.646	0.295
0.129	1.598268464	1.49136169	1.646	0.295
0.129	1.267005916	1.25527251	1.646	0.295
0.129	-0.225373611	1.38021124	1.646	0.295
0.129	1.011724577	1.5563025	1.646	0.295
0.129	0.302775116	1.34242268	1.646	0.295
0.129	0.26741776	1.77815125	1.646	0.295
0.129	1.448281289	1.44715803	1.646	0.295
0.129	1.536323315	1.30103	1.646	0.295
0.129	1.050225604	1.53147892	1.646	0.295
0.129	0.10976063	1.23044892	1.646	0.295
0.129	1.393937986	1.462398	1.646	0.295
0.129	-0.23594055	1.39794001	1.646	0.295
0.129	0.568847792	1.2787536	1.646	0.295
0.129	1.381523444	1.41497335	1.646	0.295
0.129	0.922140074	1.32221929	1.646	0.295
0.129	0.321660994	1.61278386	1.646	0.295

0.129	0.514241341	1.20411998	1.646	0.295
0.129	1.660299778	1.36172784	1.646	0.295
0.129	1.095425496	1.2787536	1.646	0.295
0.129	0.382979054	1.38021124	1.646	0.295
0.129	-0.217996439	1.81291336	1.646	0.295
0.129	1.620902343	1.38021124	1.646	0.295
0.129	1.551916784	1.39794001	1.646	0.295
0.129	1.608074767	1.41497335	1.646	0.295

standardized logPTH	standardized log([Ca++} x 104)	standardized logPTH
1.873413854	-0.557766731	1.873413854
-0.112597096	-0.557766731	-0.112597096
0.423227158	-0.463605355	0.423227158
0.292860203	-0.451171947	0.292860203
1.574880374	-0.760557203	1.574880374
0.179559337	-0.403004489	0.179559337
0.346400092	-1.091712019	0.346400092
1.747010177	-0.694422777	1.747010177
1.854659655	-1.452947627	1.854659655
0.61042306	-0.365400546	0.61042306
2.081597925	-1.083533982	2.081597925
1.540239136	-1.374860076	1.540239136
0.519798473	-0.526470639	0.519798473
1.243516016	-1.537559947	1.243516016
-0.077121049	-0.316267819	-0.077121049
0.653725731	-1.751411235	0.653725731
0.716381344	-1.116366007	0.716381344
-0.524197648	-1.084895609	-0.524197648
1.061157262	-0.572939615	1.061157262
0.292860203	-1.087620517	0.292860203
1.55187702	-1.483470265	1.55187702
-0.186221671	-0.615317259	-0.186221671
0.119461822	-0.534556547	0.119461822
0.119461822	-0.326037168	0.119461822
0.736687661	-0.824233426	0.736687661
-0.477457701	-0.520706858	-0.477457701
-0.674040572	-0.178228582	-0.674040572
2.048881364	-1.365965424	2.048881364
-1.097561713	0.552139946	-1.097561713
-0.008634995	0.83395974	-0.008634995
0.024449199	0.752888704	0.024449199
-0.524197648	1.598268464	-0.524197648
-1.324499983	1.267005916	-1.324499983
-0.900978842	-0.225373611	-0.900978842
-0.304059319	1.011724577	-0.304059319
-1.029075658	0.302775116	-1.029075658
0.44797034	0.26741776	0.44797034
-0.674040572	1.448281289	-0.674040572
-1.169389845	1.536323315	-1.169389845
-0.388207061	1.050225604	-0.388207061
-1.408647724	0.10976063	-1.408647724
-0.622379668	1.393937986	-0.622379668
-0.840881327	-0.23594055	-0.840881327
-1.244903048	0.568847792	-1.244903048
-0.783141193	1.381523444	-0.783141193
-1.097561713	0.922140074	-1.097561713
-0.112597096	0.321660994	-0.112597096

-1.497898364	0.514241341	-1.497898364
-0.963634454	1.660299778	-0.963634454
-1.244903048	1.095425496	-1.244903048
-0.900978842	0.382979054	-0.900978842
0.565807989	-0.217996439	0.565807989
-0.900978842	1.620902343	-0.900978842
-0.840881327	1.551916784	-0.840881327
-0.783141193	1.608074767	-0.783141193

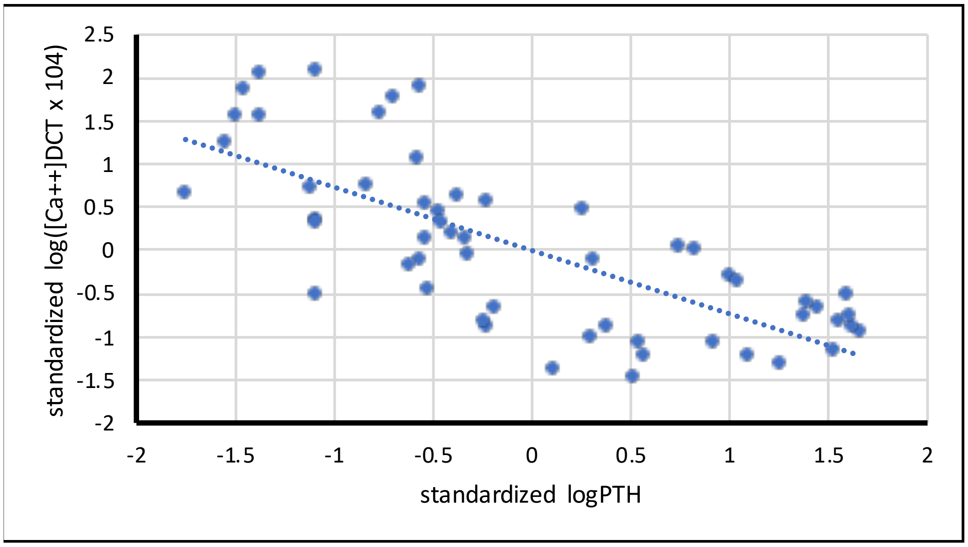

SUMMARY OUTPUT

Regression Statistics	
Multiple R	0.73551584
R Square	0.54098355
Adjusted R Sq	0.53232287
Standard Erro	0.68354976
Observations	55

ANOVA

	df	*SS*	*MS*	*F*	*Significance F*
Regression	1	29.1858241	29.1858241	62.4642727	1.5964E-10
Residual	53	24.7637347	0.46724028		
Total	54	53.9495588			

	Coefficients	*Standard Error*	*t Stat*	*P-value*	*Lower 95%*	*Upper 95%*
Intercept	-0.0019482	0.09216984	-0.0211369	0.98321581	-0.1868175	0.1829211
X Variable 1	-0.7345871	0.0929453	-7.9034342	1.5964E-10	-0.9210118	-0.5481624

Lower 95.0%	*Upper 95.0%*
-0.1868175	0.1829211
-0.9210118	-0.5481624

code	Ca+2	Ca++ x 10^4	10/(Ca++ x 10^4	ckd and ctrl
CKD2	0.0002891	2.891	3.459010723	158
CKD4	0.0002891	2.891	3.459010723	41
CKD5	0.0002973	2.973	3.363605785	59
CKD6	0.0002984	2.984	3.351206434	54
CKD7	0.0002722	2.722	3.673769287	129
CKD11	0.0003027	3.027	3.303600925	50
CKD13	0.0002467	2.467	4.053506283	56
CKD14	0.0002776	2.776	3.602305476	145
CKD15	0.0002216	2.216	4.512635379	156
CKD18	0.0003061	3.061	3.26690624	67
CKD20	0.0002473	2.473	4.043671654	182
CKD21	0.0002268	2.268	4.409171076	126
CKD23	0.0002918	2.918	3.427004798	63
CKD24	0.0002161	2.161	4.627487274	103
CKD25	0.0003106	3.106	3.219575016	42
CKD26	0.0002028	2.028	4.930966469	69
CKD27	0.0002449	2.449	4.083299306	72
CKD31	0.0002472	2.472	4.045307443	31
CKD32	0.0002878	2.878	3.474635163	91
CKD33	0.000247	2.47	4.048582996	54
CKD45	0.0002196	2.196	4.553734062	127
CKD46	0.0002842	2.842	3.518648839	39
CKD49	0.0002911	2.911	3.43524562	48
CKD50	0.0003097	3.097	3.228931224	48
CKD51	0.0002671	2.671	3.743916136	73
CKD55	0.0002923	2.923	3.421142662	32
CKD59	0.0003236	3.236	3.090234858	28
CKD62	0.0002274	2.274	4.397537379	178
N2	0.000402	4.02	2.487562189	21
N3	0.0004371	4.371	2.287805994	44
N4	0.0004267	4.267	2.343566909	45
N6	0.0005485	5.485	1.823154057	31
N7	0.0004971	4.971	2.011667673	18
N8	0.0003191	3.191	3.133813851	24
N9	0.0004608	4.608	2.170138889	36
N10	0.0003733	3.733	2.678810608	22
N11	0.0003694	3.694	2.707092583	60
N13	0.0005246	5.246	1.906214258	28
N14	0.0005385	5.385	1.857010214	20
N15	0.0004661	4.661	2.145462347	34
N16	0.0003525	3.525	2.836879433	17
N17	0.0005162	5.162	1.93723363	29
N18	0.0003181	3.181	3.143665514	25
N20	0.000404	4.04	2.475247525	19
N21	0.0005143	5.143	1.944390434	26
N24	0.0004487	4.487	2.228660575	21
N25	0.0003754	3.754	2.663825253	41

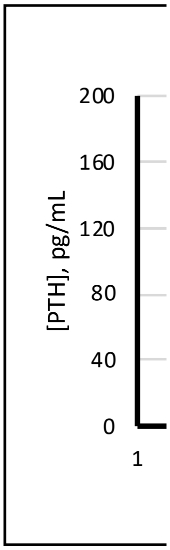

SUMMARY OU

Regression
Multiple R
R Square
Adjusted R Sq
Standard Erro
Observations

ANOVA

Regression
Residual
Total

Intercept
X Variable 1

N27	0.0003975	3.975	2.51572327	16
N29	0.0005587	5.587	1.78986934	23
N31	0.0004724	4.724	2.116850127	19
N32	0.0003823	3.823	2.615746796	24
N33	0.0003198	3.198	3.126954346	65
N35	0.0005522	5.522	1.810938066	24
N36	0.000541	5.41	1.848428835	25
N38	0.0005501	5.501	1.8178513	26

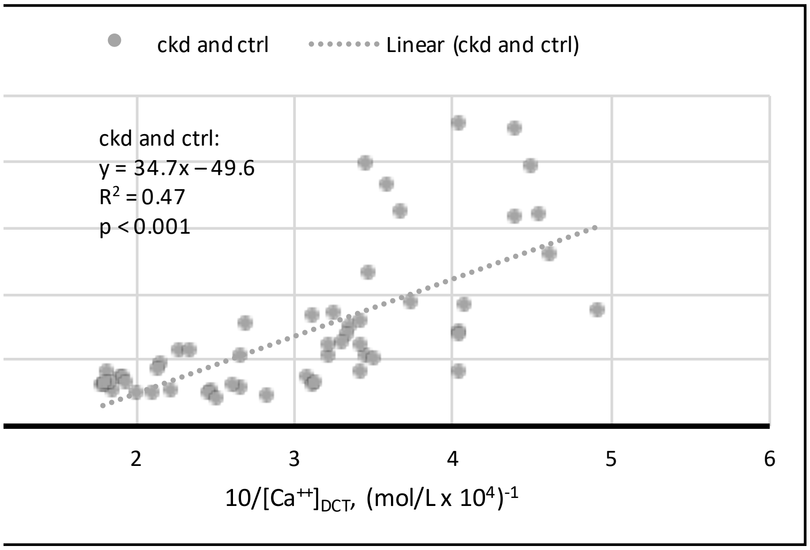

TPUT

Statistics
0.688916
0.47460525
0.46469214
32.2987801
55

df	*SS*	*MS*	*F*	*Significance F*
1	49945.3337	49945.3337	47.8765314	6.0828E-09
53	55290.1935	1043.2112		
54	105235.527			

Coefficients	*Standard Error*	*t Stat*	*P-value*	*Lower 95%*	*Upper 95%*	*Lower 95.0%*
-49.625932	15.9351943	-3.1142345	0.00297305	-81.587884	-17.66398	-81.587884
34.6878371	5.01320982	6.91928691	6.0828E-09	24.6326116	44.7430626	24.6326116

Upper 95.0%
-17.66398
44.7430626

code	Ca+2	Ca++ x 10^4	ckd	ctrl	ckd and ctrl
CKD2	0.0002891	2.891	158		70.4276721
CKD4	0.0002891	2.891	41		70.4276721
CKD5	0.0002973	2.973	59		67.1171208
CKD6	0.0002984	2.984	54		66.6868633
CKD7	0.0002722	2.722	129		77.8797943
CKD11	0.0003027	3.027	50		65.0349521
CKD13	0.0002467	2.467	56		91.056668
CKD14	0.0002776	2.776	145		75.4
CKD15	0.0002216	2.216	156		106.988448
CKD18	0.0003061	3.061	67		63.7616465
CKD20	0.0002473	2.473	182		90.7154064
CKD21	0.0002268	2.268	126		103.398236
CKD23	0.0002918	2.918	63		69.3170665
CKD24	0.0002161	2.161	103		110.973808
CKD25	0.0003106	3.106	42		62.1192531
CKD26	0.0002028	2.028	69		121.504536
CKD27	0.0002449	2.449	72		92.0904859
CKD31	0.0002472	2.472	31		90.7721683
CKD32	0.0002878	2.878	91		70.9698402
CKD33	0.000247	2.47	54		90.88583
CKD45	0.0002196	2.196	127		108.414572
CKD46	0.0002842	2.842	39		72.4971147
CKD49	0.0002911	2.911	48		69.603023
CKD50	0.0003097	3.097	48		62.4439135
CKD51	0.0002671	2.671	73		80.3138899
CKD55	0.0002923	2.923	32		69.1136504
CKD59	0.0003236	3.236	28		57.6311496
CKD62	0.0002274	2.274	178		102.994547
N2	0.000402	4.02		21	36.718408
N3	0.0004371	4.371		44	29.786868
N4	0.0004267	4.267		45	31.7217717
N6	0.0005485	5.485		31	13.6634458
N7	0.0004971	4.971		18	20.2048682
N8	0.0003191	3.191		24	59.1433406
N9	0.0004608	4.608		36	25.7038194
N10	0.0003733	3.733		22	43.3547281
N11	0.0003694	3.694		60	44.3361126
N13	0.0005246	5.246		28	16.5456348
N14	0.0005385	5.385		20	14.8382544
N15	0.0004661	4.661		34	24.8475434
N16	0.0003525	3.525		17	48.8397163
N17	0.0005162	5.162		29	17.622007
N18	0.0003181	3.181		25	59.4851933
N20	0.000404	4.04		19	36.2910891
N21	0.0005143	5.143		26	17.870348
N24	0.0004487	4.487		21	27.734522
N25	0.0003754	3.754		41	42.8347363

N27	0.0003975	3.975	16	37.6955975
N29	0.0005587	5.587	23	12.5084661
N31	0.0004724	4.724	19	23.8546994
N32	0.0003823	3.823	24	41.1664138
N33	0.0003198	3.198	65	58.9053158
N35	0.0005522	5.522	24	13.2395509
N36	0.000541	5.41	25	14.5404806
N38	0.0005501	5.501	26	13.4794401

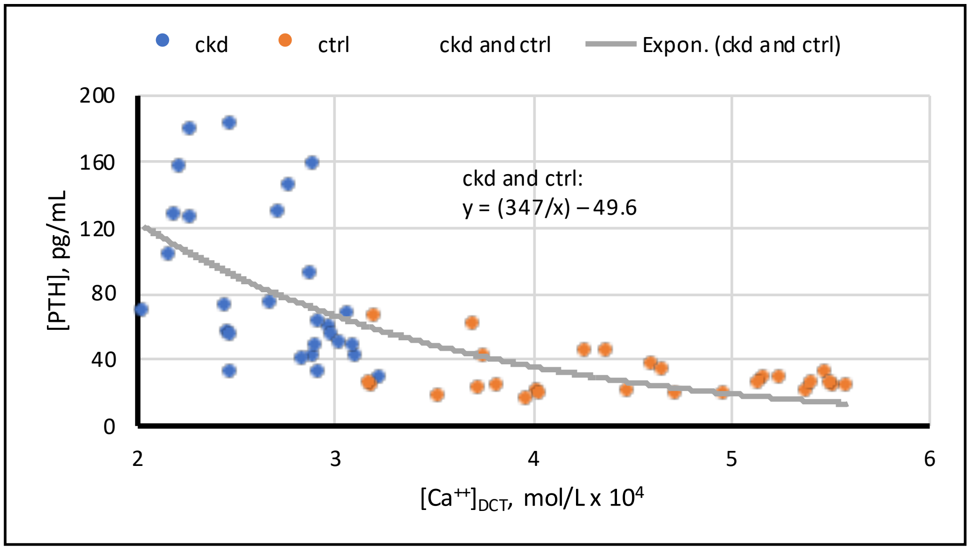

ckd
ctrl
ckd and ctrl
Expon. (ckd and ctrl)
ckd and ctrl:
y = (347/x) – 49.6
[PTH], pg/mL
200
160
120
80
40
0
2
3
4
5
6
[Ca++]DCT, mol/L x 10^4

code	Ca+2	Ca++ x 10^4	ckd	ctrl	hyperbola ckd & ctrl
CKD2	0.0002891	2.891	158		
CKD4	0.0002891	2.891	41		
CKD5	0.0002973	2.973	59		
CKD6	0.0002984	2.984	54		
CKD7	0.0002722	2.722	129		
CKD11	0.0003027	3.027	50		
CKD13	0.0002467	2.467	56		
CKD14	0.0002776	2.776	145		
CKD15	0.0002216	2.216	156		
CKD18	0.0003061	3.061	67		
CKD20	0.0002473	2.473	182		
CKD21	0.0002268	2.268	126		
CKD23	0.0002918	2.918	63		
CKD24	0.0002161	2.161	103		
CKD25	0.0003106	3.106	42		
CKD26	0.0002028	2.028	69		
CKD27	0.0002449	2.449	72		
CKD31	0.0002472	2.472	31		
CKD32	0.0002878	2.878	91		
CKD33	0.000247	2.47	54		
CKD45	0.0002196	2.196	127		
CKD46	0.0002842	2.842	39		
CKD49	0.0002911	2.911	48		
CKD50	0.0003097	3.097	48		
CKD51	0.0002671	2.671	73		
CKD55	0.0002923	2.923	32		
CKD59	0.0003236	3.236	28		
CKD62	0.0002274	2.274	178		
N2	0.000402	4.02		21	
N3	0.0004371	4.371		44	
N4	0.0004267	4.267		45	
N6	0.0005485	5.485		31	
N7	0.0004971	4.971		18	
N8	0.0003191	3.191		24	
N9	0.0004608	4.608		36	
N10	0.0003733	3.733		22	
N11	0.0003694	3.694		60	
N13	0.0005246	5.246		28	
N14	0.0005385	5.385		20	
N15	0.0004661	4.661		34	
N16	0.0003525	3.525		17	
N17	0.0005162	5.162		29	
N18	0.0003181	3.181		25	
N20	0.000404	4.04		19	
N21	0.0005143	5.143		26	
N24	0.0004487	4.487		21	
N25	0.0003754	3.754		41	

N27	0.0003975	3.975	16	
N29	0.0005587	5.587	23	
N31	0.0004724	4.724	19	
N32	0.0003823	3.823	24	
N33	0.0003198	3.198	65	
N35	0.0005522	5.522	24	
N36	0.000541	5.41	25	
N38	0.0005501	5.501	26	
		2		123.9
		2.1		115.6380952
		2.2		108.1272727
		2.3		101.2695652
		2.4		94.98333333
		2.5		89.2
		2.6		83.86153846
		2.7		78.91851852
		2.8		74.32857143
		2.9		70.05517241
		3		66.06666667
		3.1		62.33548387
		3.2		58.8375
		3.3		55.55151515
		3.4		52.45882353
		3.5		49.54285714
		3.6		46.78888889
		3.7		44.18378378
		3.8		41.71578947
		3.9		39.37435897
		4		37.15
		4.1		35.03414634
		4.2		33.01904762
		4.3		31.09767442
		4.4		29.26363636
		4.5		27.51111111
		4.6		25.83478261
		4.7		24.22978723
		4.8		22.69166667
		4.9		21.21632653
		5		19.8
		5.1		18.43921569
		5.2		17.13076923
		5.3		15.87169811
		5.4		14.65925926
		5.5		13.49090909
		5.6		12.36428571
		5.7		11.27719298
		5.8		10.22758621

5.9	9.213559322
6	8.233333333

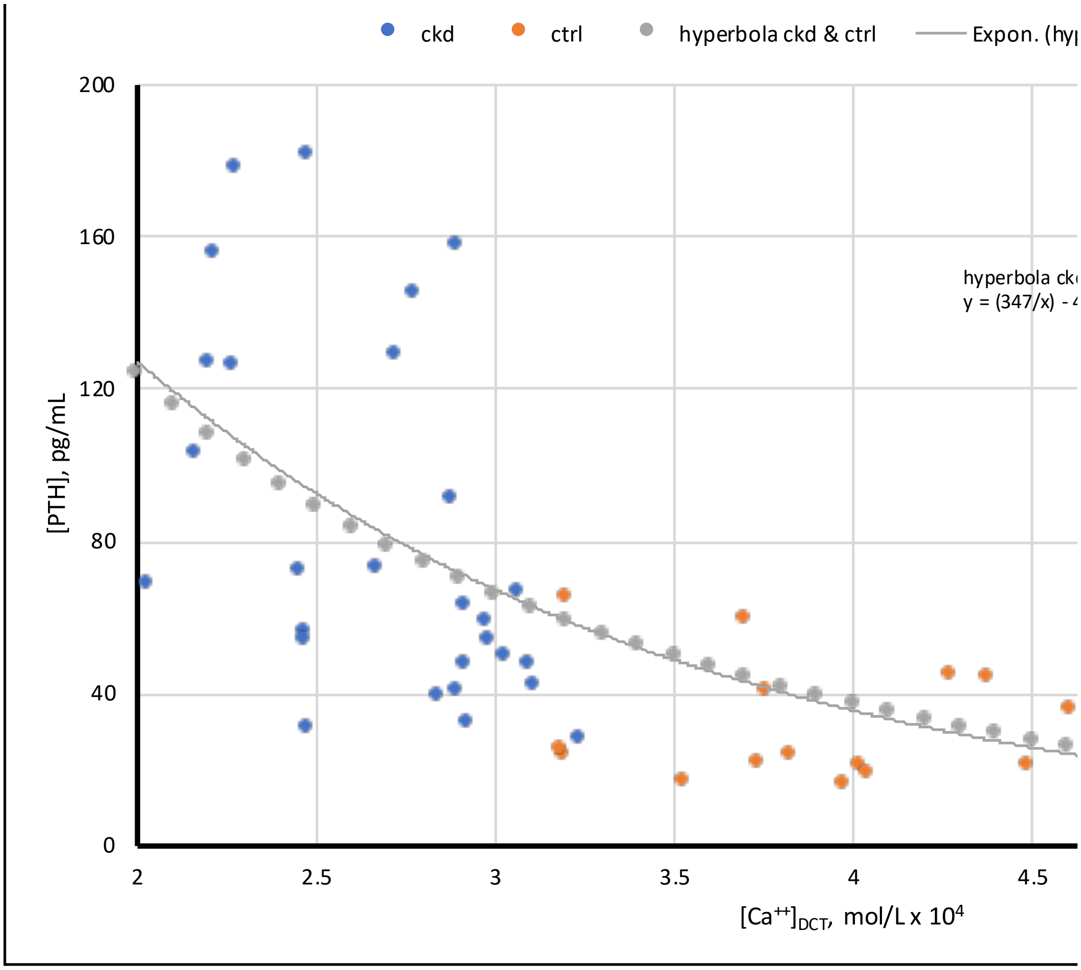

ckd
ctrl
hyperbola ckd & ctrl
Expon. (hy
200
160
120
80
40
0
[PTH], pg/mL
hyperbola ck
y = (347/x) -
2
2.5
3
3.5
4
4.5
$[Ca^{++}]_{DCT}$, mol/L x 10^4

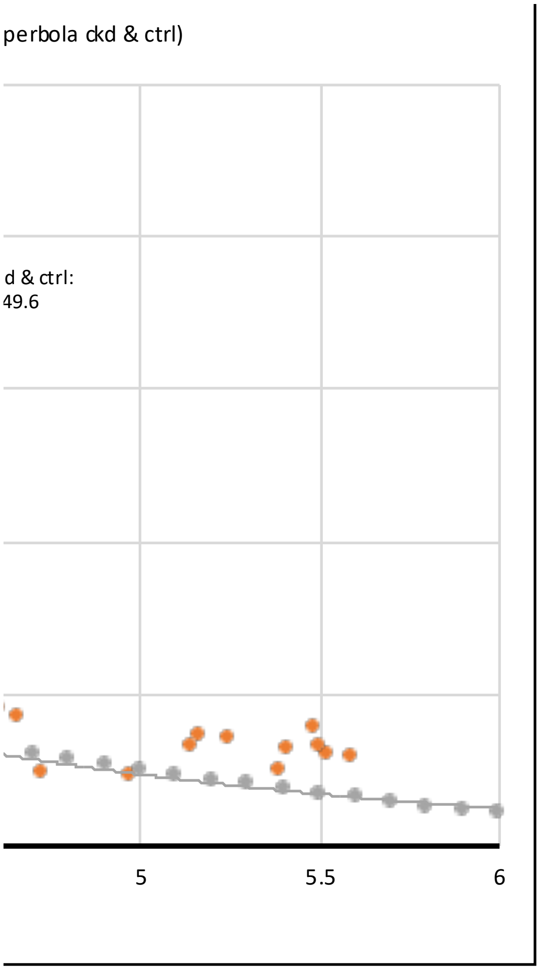
perbola ckd & ctrl)
d & ctrl:
49.6
5
5.5
6

code	Tot(P)	tot P x 10^3	ckd	ctrl
CKD2	0.00182	1.82	158	
CKD4	0.001852	1.852	41	
CKD5	0.001218	1.218	59	
CKD6	0.001549	1.549	54	
CKD7	0.002036	2.036	129	
CKD11	0.0008235	0.8235	50	
CKD13	0.002375	2.375	56	
CKD14	0.001973	1.973	145	
CKD15	0.002803	2.803	156	
CKD18	0.001003	1.003	67	
CKD20	0.002391	2.391	182	
CKD21	0.002718	2.718	126	
CKD23	0.001624	1.624	63	
CKD24	0.002935	2.935	103	
CKD25	0.001507	1.507	42	
CKD26	0.003251	3.251	69	
CKD27	0.002414	2.414	72	
CKD31	0.002402	2.402	31	
CKD32	0.001489	1.489	91	
CKD33	0.002371	2.371	54	
CKD45	0.002874	2.874	127	
CKD46	0.001331	1.331	39	
CKD49	0.001242	1.242	48	
CKD50	0.0007575	0.7575	48	
CKD51	0.002103	2.103	73	
CKD55	0.0007141	0.7141	32	
CKD59	0.0006192	0.6192	28	
CKD62	0.002702	2.702	178	
N2	0.001258	1.258		21
N3	0.001075	1.075		44
N4	0.001127	1.127		45
N6	0.0005899	0.5899		31
N7	0.0008665	0.8665		18
N8	0.001751	1.751		24
N9	0.0009826	0.9826		36
N10	0.001394	1.394		22
N11	0.001392	1.392		60
N13	0.0006849	0.6849		28
N14	0.0007527	0.7527		20
N15	0.0009714	0.9714		34
N16	0.001539	1.539		17
N17	0.0008413	0.8413		29
N18	0.001776	1.776		25
N20	0.001206	1.206		19
N21	0.0007058	0.7058		26
N24	0.001062	1.062		21
N25	0.00135	1.35		41

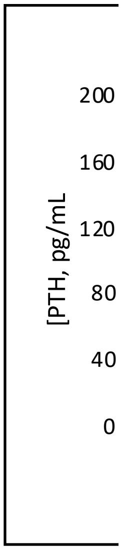

SUMMARY OU

Regression
Multiple R
R Square
Adjusted R Sq
Standard Erro
Observations

ANOVA

Regression
Residual
Total

Intercept
X Variable 1

SUMMARY OU

Regression
Multiple R
R Square
Adjusted R Sq
Standard Erro
Observations

N27	0.001305	1.305	16	
N29	0.0006108	0.6108	23	ANOVA
N31	0.000974	0.974	19	
N32	0.001335	1.335	24	Regression
N33	0.001729	1.729	65	Residual
N35	0.0004466	0.4466	24	Total
N36	0.0007627	0.7627	25	
N38	0.0005282	0.5282	26	
				Intercept
				X Variable 1

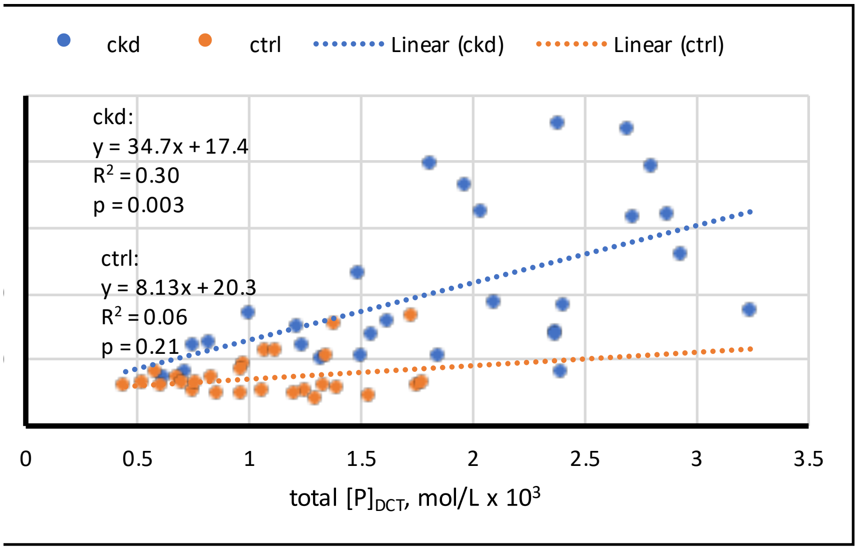

TPUT ckd

Statistics
0.54792622
0.30022314
0.27330865
40.6180126
28

df	SS	MS	F	Significance F
1	18403.282	18403.282	11.1547012	0.00254162
26	42895.3966	1649.82295		
27	61298.6786			

Coefficients	Standard Error	t Stat	P-value	Lower 95%	Upper 95%	Lower 95.0%
17.3899266	21.0611084	0.82568905	0.41649288	-25.901802	60.6816549	-25.901802
34.6725079	10.3814086	3.33986544	0.00254162	13.3332169	56.011799	13.3332169

TPUT

Statistics
0.25035381
0.06267703
0.02518411
12.2677046
27

df	*SS*	*MS*	*F*	*Significance F*
1	251.585607	251.585607	1.6717032	0.20785198
25	3762.41439	150.496576		
26	4014			

Coefficients	*Standard Error*	*t Stat*	*P-value*	*Lower 95%*	*Upper 95%*	*Lower 95.0%*
20.261812	7.15887404	2.83030709	0.00904204	5.51783489	35.0057891	5.51783489
8.13095617	6.28871675	1.29294362	0.20785198	-4.8208984	21.0828108	-4.8208984

Upper 95.0%
60.6816549
56.011799

Upper 95.0%
35.0057891
21.0828108

code	CaHPO4	cahpo4 x 10^5	ckd	ctrl
CKD2	2.52E-05	2.52	158	
CKD4	2.56E-05	2.557	41	
CKD5	1.74E-05	1.743	59	
CKD6	2.22E-05	2.221	54	
CKD7	2.64E-05	2.635	129	
CKD11	1.21E-05	1.205	50	
CKD13	2.77E-05	2.769	56	
CKD14	2.61E-05	2.61	145	
CKD15	2.92E-05	2.922	156	
CKD18	1.48E-05	1.481	67	
CKD20	2.77E-05	2.766	182	
CKD21	2.89E-05	2.889	126	
CKD23	2.27E-05	2.273	63	
CKD24	2.96E-05	2.959	103	
CKD25	2.24E-05	2.244	42	
CKD26	3.06E-05	3.056	69	
CKD27	2.78E-05	2.779	72	
CKD31	2.77E-05	2.766	31	
CKD32	2.06E-05	2.058	91	
CKD33	2.77E-05	2.767	54	
CKD45	2.94E-05	2.936	127	
CKD46	1.82E-05	1.819	39	
CKD49	1.74E-05	1.741	48	
CKD50	1.13E-05	1.133	48	
CKD51	2.66E-05	2.661	73	
CKD55	1.01E-05	1.011	32	
CKD59	9.70E-06	0.9696	28	
CKD62	2.89E-05	2.885	178	
N2	2.17E-05	2.168		21
N3	2.08E-05	2.079		44
N4	2.10E-05	2.104		45
N6	1.54E-05	1.54		31
N7	1.95E-05	1.949		18
N8	2.43E-05	2.434		24
N9	2.03E-05	2.025		36
N10	2.25E-05	2.25		22
N11	2.26E-05	2.261		60
N13	1.71E-05	1.71		28
N14	1.87E-05	1.873		20
N15	2.01E-05	2.013		34
N16	2.32E-05	2.315		17
N17	1.91E-05	1.913		29
N18	2.44E-05	2.438		25
N20	2.16E-05	2.162		19
N21	1.73E-05	1.729		26
N24	2.05E-05	2.052		21
N25	2.24E-05	2.243		41

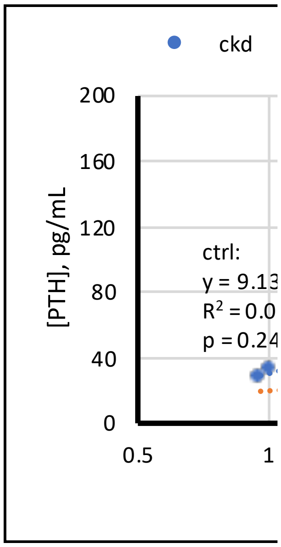

SUMMARY OU

Regression
Multiple R
R Square
Adjusted R Sq
Standard Erro
Observations

ANOVA

Regression
Residual
Total

Intercept
X Variable 1

SUMMARY OU

Regression
Multiple R
R Square
Adjusted R Sq
Standard Erro
Observations

N27	2.18E-05	2.18	16	
N29	1.62E-05	1.622	23	ANOVA
N31	2.00E-05	1.999	19	
N32	2.22E-05	2.223	24	Regression
N33	2.43E-05	2.431	65	Residual
N35	1.18E-05	1.175	24	Total
N36	1.87E-05	1.869	25	
N38	1.38E-05	1.383	26	
				Intercept
				X Variable 1

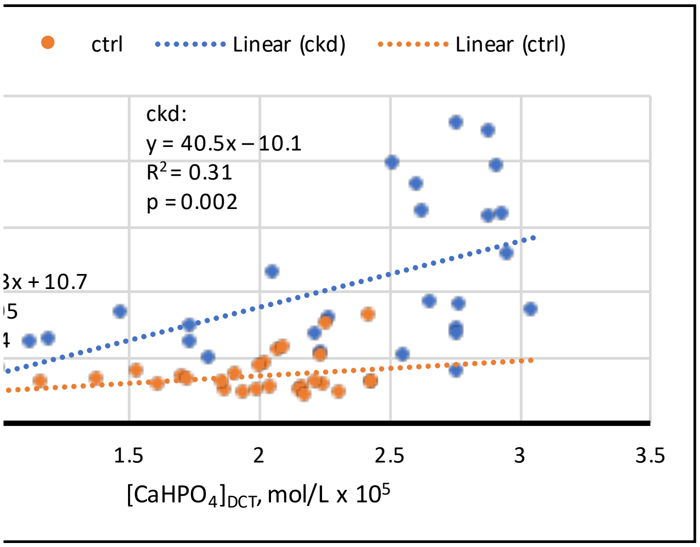

TPUT ckd

Statistics
0.55709942
0.31035976
0.28383513
40.3227534
28

df	*SS*	*MS*	*F*	*Significance F*
1	19024.6431	19024.6431	11.7008162	0.00207387
26	42274.0355	1625.92444		
27	61298.6786			

Coefficients	*Standard Error*	*t Stat*	*P-value*	*Lower 95%*	*Upper 95%*	*Lower 95.0%*
-10.107593	28.2357058	-0.357972	0.72325416	-68.146917	47.9317317	-68.146917
40.4503042	11.8253421	3.42064559	0.00207387	16.1429654	64.7576429	16.1429654

TPUT ctrl

Statistics
0.23357271
0.05455621
0.01673846
12.3207327
27

df	*SS*	*MS*	*F*	*Significance F*
1	218.988633	218.988633	1.44260854	0.24097389
25	3795.01137	151.800455		
26	4014			

Coefficients	*Standard Error*	*t Stat*	*P-value*	*Lower 95%*	*Upper 95%*	*Lower 95.0%*
10.6960083	15.4228889	0.69351523	0.49437994	-21.068026	42.4600425	-21.068026
9.12832983	7.60006093	1.2010864	0.24097389	-6.5242887	24.7809483	-6.5242887

Upper 95.0%
47.9317317
64.7576429

Upper 95.0%
42.4600425
24.7809483
