# Supplementary material for: Chemical evidence for the tradeoff-in-the-nephron hypothesis to explain secondary hyperparathyroidism
Source: PLoS One. 2022 Aug 1;17(8):e0272380. doi: 10.1371/journal.pone.0272380 (PMC9342777; doi:10.1371/journal.pone.0272380)

Figure 4. Regressions assuming pH 7.0 and precipitation of amorphous Ca3(PO4)2

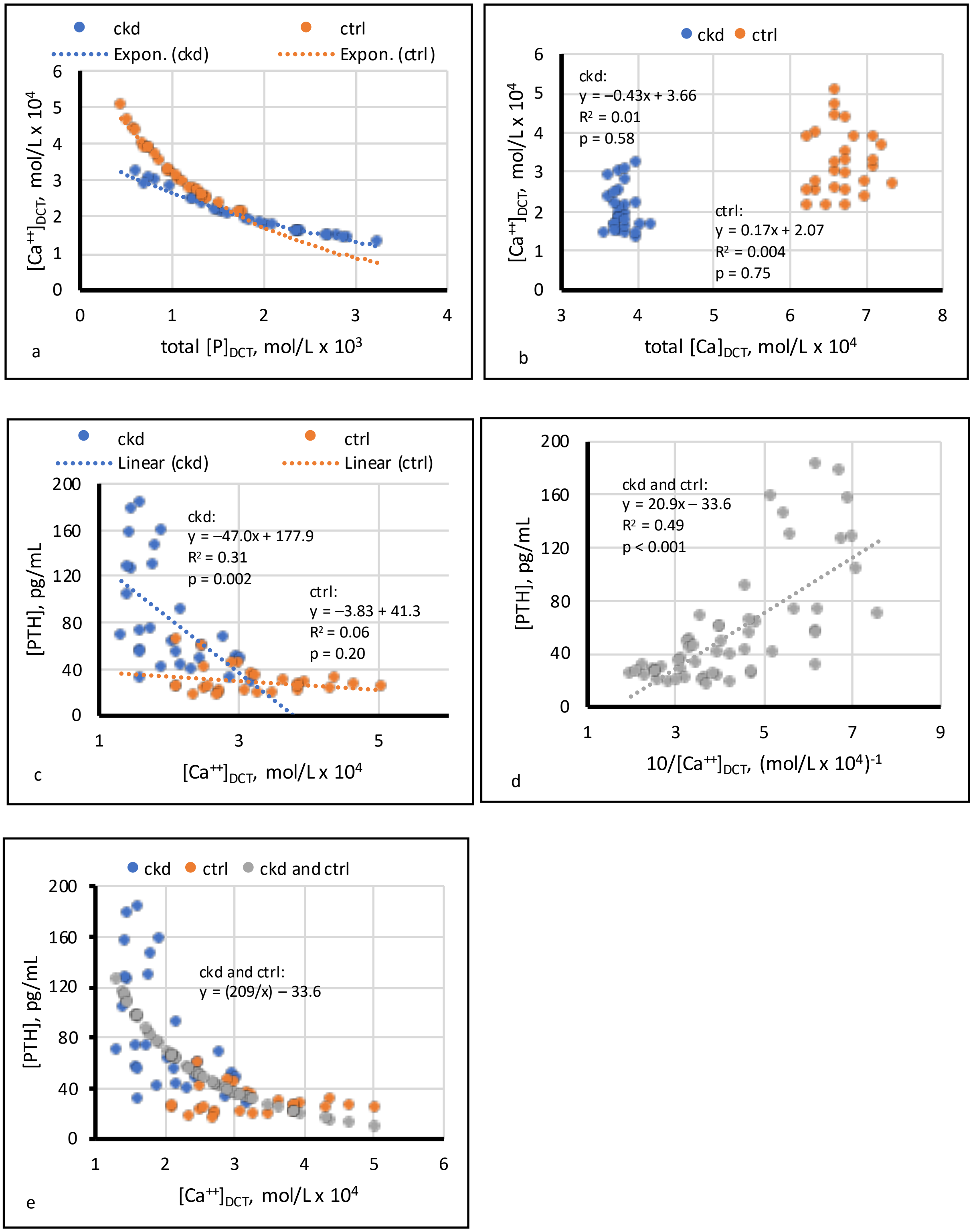

Figure 4. Regressions assuming pH 7.0 and precipitation of amorphous $Ca_3(PO_4)_2$

code	IStr	Tot(P)	Tot(Ca)	Ca+2	CaCitric	CaOxalic	CaHPO4	CaHCO3+
CKD2	0.03661	0.00182	0.000378	0.000192	2.47E-05	4.54E-07	1.98E-05	9.96E-06
CKD4	0.03663	0.001852	0.000385	0.00019	2.46E-05	4.50E-07	1.99E-05	9.87E-06
CKD5	0.03602	0.001218	0.000378	0.000249	2.89E-05	5.78E-07	1.74E-05	1.29E-05
CKD6	0.03631	0.001549	0.000385	0.000214	2.64E-05	5.02E-07	1.88E-05	1.11E-05
CKD7	0.03683	0.002036	0.000378	0.000179	2.36E-05	4.23E-07	2.05E-05	9.24E-06
CKD11	0.03576	0.000824	0.000378	0.000299	3.20E-05	6.84E-07	1.46E-05	1.56E-05
CKD13	0.03718	0.002375	0.000371	0.000161	2.21E-05	3.84E-07	2.16E-05	8.32E-06
CKD14	0.03679	0.001973	0.000378	0.000182	2.39E-05	4.32E-07	2.03E-05	9.44E-06
CKD15	0.03767	0.002803	0.000356	0.000144	2.05E-05	3.44E-07	2.29E-05	7.42E-06
CKD18	0.03584	0.001003	0.000385	0.00028	3.09E-05	6.46E-07	1.64E-05	1.46E-05
CKD20	0.03719	0.002391	0.000406	0.000162	2.21E-05	3.84E-07	2.16E-05	8.34E-06
CKD21	0.03758	0.002718	0.000378	0.000148	2.08E-05	3.52E-07	2.26E-05	7.61E-06
CKD23	0.03638	0.001624	0.000378	0.000207	2.59E-05	4.87E-07	1.91E-05	1.07E-05
CKD24	0.0378	0.002935	0.000385	0.000141	2.01E-05	3.36E-07	2.32E-05	7.23E-06
CKD25	0.03626	0.001507	0.000399	0.000219	2.68E-05	5.13E-07	1.86E-05	1.14E-05
CKD26	0.03818	0.003251	0.000399	0.000132	1.92E-05	3.15E-07	2.39E-05	6.76E-06
CKD27	0.03726	0.002414	0.000385	0.00016	2.20E-05	3.81E-07	2.17E-05	8.25E-06
CKD31	0.03721	0.002402	0.000421	0.000162	2.21E-05	3.84E-07	2.16E-05	8.34E-06
CKD32	0.03626	0.001489	0.000371	0.000218	2.68E-05	5.13E-07	1.86E-05	1.13E-05
CKD33	0.03719	0.002371	0.000371	0.000161	2.21E-05	3.84E-07	2.16E-05	8.33E-06
CKD45	0.03774	0.002874	0.000399	0.000143	2.04E-05	3.41E-07	2.30E-05	7.35E-06
CKD46	0.03612	0.001331	0.000364	0.000234	2.79E-05	5.47E-07	1.79E-05	1.22E-05
CKD49	0.03605	0.001242	0.000371	0.000245	2.86E-05	5.70E-07	1.75E-05	1.27E-05
CKD50	0.03573	0.000758	0.000385	0.000306	3.24E-05	6.99E-07	1.37E-05	1.59E-05
CKD51	0.03689	0.002103	0.000385	0.000175	2.33E-05	4.16E-07	2.07E-05	9.06E-06
CKD55	0.03563	0.000714	0.000364	0.000289	3.14E-05	6.64E-07	1.22E-05	1.51E-05
CKD59	0.03553	0.000619	0.000399	0.00032	3.31E-05	7.29E-07	1.18E-05	1.67E-05
CKD62	0.03756	0.002702	0.000371	0.000148	2.09E-05	3.53E-07	2.26E-05	7.63E-06
N2	0.03593	0.001258	0.000699	0.000273	3.05E-05	6.30E-07	1.66E-05	1.42E-05
N3	0.0358	0.001075	0.000661	0.000301	3.21E-05	6.88E-07	1.58E-05	1.57E-05
N4	0.03583	0.001127	0.000674	0.000292	3.16E-05	6.71E-07	1.60E-05	1.52E-05
N6	0.03561	0.00059	0.000661	0.000439	3.86E-05	9.66E-07	1.31E-05	2.29E-05
N7	0.03568	0.000867	0.000674	0.00035	3.47E-05	7.90E-07	1.47E-05	1.82E-05
N8	0.03636	0.001751	0.000649	0.000212	2.63E-05	4.97E-07	1.89E-05	1.10E-05
N9	0.03575	0.000983	0.000661	0.00032	3.31E-05	7.28E-07	1.53E-05	1.67E-05
N10	0.03603	0.001394	0.000674	0.000251	2.91E-05	5.84E-07	1.73E-05	1.31E-05
N11	0.03605	0.001392	0.000636	0.000248	2.89E-05	5.78E-07	1.74E-05	1.29E-05
N13	0.03563	0.000685	0.000636	0.000397	3.69E-05	8.84E-07	1.38E-05	2.07E-05
N14	0.03563	0.000753	0.000686	0.000386	3.64E-05	8.62E-07	1.40E-05	2.01E-05
N15	0.03574	0.000971	0.000674	0.000324	3.34E-05	7.37E-07	1.52E-05	1.69E-05
N16	0.03614	0.001539	0.000699	0.000236	2.80E-05	5.50E-07	1.79E-05	1.23E-05
N17	0.03567	0.000841	0.000724	0.000366	3.55E-05	8.23E-07	1.43E-05	1.91E-05
N18	0.03637	0.001776	0.000674	0.000211	2.62E-05	4.96E-07	1.89E-05	1.09E-05
N20	0.0359	0.001206	0.000636	0.000275	3.05E-05	6.34E-07	1.66E-05	1.43E-05
N21	0.03563	0.000706	0.000625	0.000388	3.65E-05	8.66E-07	1.39E-05	2.02E-05
N24	0.03578	0.001062	0.000711	0.00031	3.26E-05	7.08E-07	1.56E-05	1.61E-05
N25	0.03602	0.00135	0.000625	0.000253	2.92E-05	5.87E-07	1.73E-05	1.31E-05

N27	0.03594	0.001305	0.000736	0.00027	3.02E-05	6.23E-07	1.67E-05	1.40E-05
N29	0.03561	0.000611	0.000674	0.000434	3.84E-05	9.56E-07	1.32E-05	2.26E-05
N31	0.03573	0.000974	0.000711	0.000329	3.37E-05	7.48E-07	1.51E-05	1.72E-05
N32	0.03599	0.001335	0.000661	0.000258	2.95E-05	5.98E-07	1.71E-05	1.34E-05
N33	0.03635	0.001729	0.000625	0.000212	2.63E-05	4.98E-07	1.88E-05	1.10E-05
N35	0.03563	0.000447	0.000661	0.000505	4.10E-05	1.09E-06	1.22E-05	2.63E-05
N36	0.03563	0.000763	0.000711	0.000388	3.65E-05	8.66E-07	1.39E-05	2.02E-05
N38	0.0356	0.000528	0.000661	0.000465	3.96E-05	1.02E-06	1.27E-05	2.42E-05
CKD16	0.03652	0.001797	0.000428	0.000196	2.51E-05	4.63E-07	1.96E-05	1.02E-05
CKD41	0.03698	0.002144	0.000307	0.00017	2.29E-05	4.04E-07	2.10E-05	8.80E-06

CaSO4	lgSI(Ca3PO4	lgSI(Brushite)
4.77E-06	-6.21E-07	-0.7214
4.72E-06	4.14E-07	-0.7193
6.18E-06	-1.04E-06	-0.7773
5.31E-06	-1.24E-06	-0.7445
4.42E-06	-2.07E-07	-0.7053
7.43E-06	-0.07418	-0.8544
3.98E-06	1.24E-06	-0.6829
4.52E-06	4.14E-07	-0.7099
3.55E-06	-1.04E-06	-0.6586
6.98E-06	-8.28E-07	-0.8037
3.99E-06	-2.07E-07	-0.6834
3.63E-06	-6.21E-07	-0.6638
5.14E-06	4.14E-07	-0.7374
3.45E-06	0	-0.6529
5.43E-06	4.14E-07	-0.7493
3.22E-06	2.07E-07	-0.6386
3.95E-06	-8.28E-07	-0.6812
3.99E-06	-2.07E-07	-0.6833
5.43E-06	-4.14E-07	-0.7492
3.98E-06	-1.24E-06	-0.6831
3.51E-06	8.28E-07	-0.6565
5.82E-06	-1.24E-06	-0.7643
6.09E-06	4.14E-07	-0.7742
7.60E-06	-0.1165	-0.8807
4.33E-06	4.14E-07	-0.701
7.19E-06	-0.2401	-0.9301
7.97E-06	-0.2308	-0.9478
3.64E-06	8.28E-07	-0.6644
6.79E-06	0	-0.7978
7.47E-06	-1.04E-06	-0.8187
7.27E-06	-1.04E-06	-0.8127
1.09E-05	4.14E-07	-0.901
8.69E-06	-4.14E-07	-0.8518
5.25E-06	2.07E-07	-0.7422
7.95E-06	6.21E-07	-0.8322
6.25E-06	8.28E-07	-0.7797
6.18E-06	0	-0.7772
9.84E-06	-4.14E-07	-0.8793
9.56E-06	8.28E-07	-0.873
8.05E-06	0	-0.8351
5.86E-06	2.07E-07	-0.7658
9.09E-06	4.14E-07	-0.8617
5.23E-06	-2.07E-07	-0.7415
6.83E-06	0	-0.7991
9.61E-06	-6.21E-07	-0.8741
7.70E-06	1.24E-06	-0.8254
6.29E-06	-2.07E-07	-0.781

6.70E-06	6.21E-07	-0.7951
1.07E-05	-1.66E-06	-0.8985
8.18E-06	-2.07E-07	-0.8386
6.42E-06	1.24E-06	-0.7855
5.27E-06	-6.21E-07	-0.7427
1.24E-05	8.28E-07	-0.9314
9.61E-06	-2.07E-07	-0.8742
1.15E-05	0	-0.9137
4.87E-06	0	-0.7258
4.21E-06	-6.21E-07	-0.6947

code	[P]s	EP	Ecr	EP/Ecr	[P]u	[cr]s	[cr]u	EP/Ccr	TRP/Ccr	FEP	FTRP	pth 1-84
CKD2	2.7	597	1024.3	0.582837	27.1	2.9	53.4	1.471723	1.228277	0.545083	0.454917	158
CKD4	2.5	665.4	1458	0.456379	47.7	2	144.5	0.660208	1.839792	0.264083	0.735917	41
CKD5	2.5	646.8	1468	0.440599	45.3	2.1	156.2	0.609027	1.890973	0.243611	0.756389	59
CKD6	4.2	992.4	1157.2	0.857587	93.2	1.7	91.2	1.737281	2.462719	0.413638	0.586362	54
CKD7	3.8	604.3	1228.9	0.491741	55.3	3.4	127.9	1.470055	2.329945	0.386857	0.613143	129
CKD11	3.2	424.6	731	0.580848	42.2	2	99.5	0.848241	2.351759	0.265075	0.734925	50
CKD13	3.7	816.4	1224.6	0.666667	29.5	2.8	49.2	1.678862	2.02114	0.453746	0.546254	56
CKD14	5.3	431.5	1148.5	0.375707	43.3	4.8	101.7	2.043658	3.25634	0.385596	0.614404	145
CKD15	4.4	963.6	1195.3	0.806157	29.5	2.9	40	2.13875	2.26125	0.48608	0.51392	156
CKD18	3.3	564	746	0.756032	40.5	1.8	61.5	1.185366	2.114634	0.359202	0.640798	67
CKD20	3.7	747	1164	0.641753	34.6	3.1	63.2	1.697152	2.002848	0.45869	0.54131	182
CKD21	4.4	1189	1372.6	0.866239	79	2.3	108.9	1.668503	2.731497	0.379205	0.620795	126
CKD23	3.4	888.3	1076	0.825558	31	1.9	39.4	1.494924	1.905076	0.439683	0.560317	63
CKD24	4.8	917	1311	0.699466	44.1	3	53.8	2.459108	2.340892	0.512314	0.487686	103
CKD25	3.9	1036	1922.2	0.538966	22.6	1.6	34.6	1.045087	2.854913	0.267971	0.732029	42
CKD26	4.5	914.3	1293.6	0.706787	47.6	3.5	64.7	2.574961	1.925039	0.572214	0.427786	69
CKD27	2.7	1056	1339	0.788648	100.1	2.3	100.1	2.3	1.610889	0.851852	0.148148	72
CKD31	4	863.2	1169.6	0.73803	65.2	2.7	81.9	2.149451	1.85055	0.537363	0.462637	31
CKD32	3.9	674.5	853.6	0.790183	41	2.2	46.6	1.935622	1.964378	0.496313	0.503687	91
CKD33	4.1	1037.3	1605.9	0.645931	52	2.3	97.8	1.222904	2.877096	0.298269	0.701731	54
CKD45	2.7	1302.1	1710.4	0.761284	58.3	2.2	75.2	1.705585	0.994415	0.631698	0.368302	127
CKD46	3.2	873.1	1674.2	0.521503	43.6	1.6	178.7	0.390375	2.80963	0.121992	0.878008	39
CKD49	2.9	814.8	1146.5	0.710685	41	1.6	68	0.964706	1.935294	0.332657	0.667343	48
CKD50	3.1	579.9	1055	0.549668	19.7	1.4	46.3	0.59568	2.50432	0.192155	0.807845	48
CKD51	2.8	1117	1866	0.598607	43.4	2	126.8	0.684543	2.11546	0.244479	0.755521	73
CKD55	2.6	312.4	1167.3	0.267626	10.6	2.8	70.3	0.422191	2.17781	0.162381	0.837619	32
CKD59	3.5	454.7	1080.7	0.420746	38.5	1.7	104.2	0.628119	2.871881	0.179463	0.820537	28
CKD62	3.6	1139.8	1536.3	0.741912	19.3	2.8	26.1	2.070498	1.529502	0.575138	0.424862	178
CKD16	3.8	617.6	865.6	0.713494	69.5	2.7	99	1.895455	1.904545	0.498804	0.501196	169
CKD41	2.7	904.5	1679.1	0.538681	14.5	2.4	53.2	0.654135	2.04586	0.242272	0.757728	79

FGF23	1,25	eGFR	100/eGFR	25D	Cai	ECa/Ccr	[Ca]uf
35.403	25.1	21	4.761905	37.4	4.61	0.168	5.3
13.383	38	23	4.347826	42.2	5.09	0.039	5.4
13.543	55.9	34	2.941176	47.4	4.81	0.027	5.3
26.494	74.7	41	2.439024	44.3	5.09	0.097	5.4
30.706	39.6	19	5.263158	41.7	4.93	0.053	5.3
17.384	89.6	33	3.030303	55.8	4.93	0.046	5.3
23.805	20.4	22	4.545455	21	5.05	0.114	5.2
68.316	64.2	14	7.142857	34.2	4.53	0.094	5.3
26	27.6	22	4.545455	18.9	4.73	0.145	5
17.415	57.2	36	2.777778	42.7	4.85	0.059	5.4
43.713	33.7	20	5	49.1	5.05	0.123	5.7
26.399	19.3	28	3.571429	20.5	4.77	0.034	5.3
35.711	49.6	35	2.857143	27.7	5.13	0.019	5.3
48.446	25.1	20	5	31	4.73	0.028	5.4
12.507	52.6	44	2.272727	lost	5.25	0.069	5.6
35.564	21.4	18	5.555556	25.6	5.45	0.119	5.6
42.96	44.9	28	3.571429	26.4	5.13	0.018	5.4
24.519		23	4.347826	27.5	5.13	0.105	5.9
89.109	21.7	29	3.448276	32.2	4.81	0.038	5.2
26.285	27.3	28	3.571429	49.5	5.01	0.019	5.2
25.303	47.8	29	3.448276	30.1	4.81	0.009	5.6
17.44	31.8	42	2.380952	24	4.97	0.037	5.1
48.504	25.5	42	2.380952	24.6	4.97	0.08	5.2
14.623	75.1	49	2.040816	36.9	4.89	0.07	5.4
19.062	26.5	34	2.941176	17.8	5.09	0.002	5.4
20.402	43.4	28	3.571429	35.9	5.01	0.092	5.1
16.126	74.9	47	2.12766	51.9	5.01	0.011	5.6
20.506	53.1	27	3.703704	21.5	5.17	0.21	5.2
36.399	58.6	22	4.545455	71.2	5.41	0.055	6
38.187	30	27	3.703704	17.4	4.89	0.014	4.3

CODE	[cr]s	eGFR	[P]s	[Ca]i	[Ca]uf	[PTH]1-84	1-84 & 7-84	[PTH]7-84
N2	0.9	89	3.1	5.00875	5.6	21	24	3
N3	0.8	101	3.5	4.88854	5.3	44	77	33
N4	0.7	93	3.5	5.12896	5.4	45	72	27
N6	0.8	103	3	4.96868	5.3	31	52	21
N7	0.7	94	3.5	5.04882	5.4	18	28	10
N8	1	79	2.8	4.92861	5.2	24	29	5
N9	0.8	77	3.2	5.08889	5.3	36	57	21
N10	0.8	73	3.4	5.12896	5.4	22	31	9
N11	0.8	108	2.9	4.96868	5.1	60	120	60
N13	0.7	87	4.9	4.8084	5.1	28	50	22
N14	0.9	93	2.1	5.04882	5.5	20	30	10
N15	0.7	96	3.1	5.08889	5.4	34	59	25
N16	0.9	96	3.4	5.16903	5.6	17	22	5
N17	1.1	73	3.4	4.84847	5.8	29	49	20
N18	0.7	90	4	5.08889	5.2	25	45	20
N20	0.8	75	4.1	5.12896	5.1	19	32	13
N21	0.8	75	2.9	5.00875	5.00875	26	51	25
N24	0.8	75	4	5.08889	5.7	21	36	15
N25	1.1	89	3.2	5.00875	5.00875	41	82	41
N27	0.8	74	3.1	5.24917	5.9	16	27	11
N29	0.7	85	4.2	5.00875	5.4	23	46	23
N31	0.9	72	3.4	5.04882	5.7	19	30	11
N32	1.3	89	3.6	5.08889	5.3	24	36	12
N33	0.9	93	4.9	5.00875	5.00875	65	93	28
N35	0.9	78	4	4.96868	5.3	24	51	27
N36	1	84	2.7	4.96868	5.7	25	47	22
N38	0.7	87	3.2	5.2091	5.3	26	49	23
N19	1	78	3.2	5.24917	5.4	23	38	13

25D	1,25D	FGF23	24h EP	24h Ecr	24h EP/Ecr	24h EP/Ccr	spot EP/Ccr
40.5	66.7	11.018	999.6	1493.8	0.66916589	0.602249297	0.44787
27.7	38.7	4.5325	969.6	1717.8	0.56444289	0.451554314	0.41669
35.3	71.8	23.379	936	1173	0.79795396	0.558567775	0.470909
55.8	66.1	117.63	542.5	1354.8	0.40042811	0.320342486	0.186121
33.5	60	9.3336	727.2	937.3	0.77584551	0.54309186	0.438913
30.8	35	14.798	1235.2	2084.7	0.59250732	0.592507315	0.278234
46.2	55.3	17.591	675.5	1196.4	0.5646105	0.451688399	0.3855
47.8	47	12.978	908.7	1238.4	0.73376938	0.587015504	0.380812
20	90.9	15.294	1342	1105.4	1.21404017	0.971232133	0.45463
25.4	23.4	12.749	532	910	0.58461538	0.409230769	0.583639
24.9	25.8	34.843	625	1416.1	0.44135301	0.397217711	0.21772
22.1	83.3	16.026	832.6	1279.5	0.65072294	0.455506057	0.445848
30.7	68.5	32.967	1319.3	1731.6	0.76189651	0.685706861	0.589039
21.2	53.9	8.3066	548.3	2387.5	0.22965445	0.252619895	0.287347
27.4	28.1	16.158	1427.2	1272.9	1.12121926	0.784853484	0.43
32.5	35.1	13.3182	807.8	1426.5	0.56628111	0.453024886	0.343947
n/a	36.4	12.5182	472.6	867.8	0.54459553	0.435676423	0.292998
19.8	52.8	12.1842	711	1138.5	0.62450593	0.499604743	0.38037
17.5	31.7	7.932	1072.7	2022.9	0.53027831	0.583306145	0.668646
98.1	136.3	10.6568	862.4	1252.8	0.68837803	0.550702427	0.366213
31.1	91	11.7211	463.5	1039.5	0.44588745	0.312121212	0.225379
22.9	94.2	12.6715	626.1	1058.3	0.59160918	0.532448266	0.38008
40.4	77.1	10.5577	882	2416	0.36506623	0.474586093	0.31234
15.2	25	19.7492	1436	2001.7	0.71739022	0.645651196	0.46075
29.6	36.7	15.9907	311	861.7	0.36091447	0.324823024	0.58508
21.9	41.6	5.8652	572	805.2	0.71038251	0.710382514	0.5
30.3	64.6	23.7653	410.3	801.5	0.51191516	0.358340611	0.110526
17	38.5	14.5962	n/a	n/a	n/a	n/a	0.8197941

spot TRP/Ccr	24h ECa	24h ECa/Ecr	24h ECa/Ccr	spot ECa/Ccr	spot TRCa/Ccr	100/eGFR
2.65213	127.5	0.085352792	0.076817512	0.08872	5.51128	1.123596
3.08331	139.2	0.081033881	0.064827104	0.02369	5.27631	0.990099
3.029091	71.2	0.060699062	0.042489344	0.082197	5.317803	1.075269
2.813879	49	0.0361677	0.02893416	0.008181	5.281819	0.970874
3.061087	96.3	0.102741918	0.071919343	0.091483	5.308517	1.06383
2.521766	110.9	0.053197103	0.053197103	0.067762	5.132238	1.265823
2.8145	104.5	0.087345369	0.069876296	0.04264	5.25736	1.298701
3.019188	138.5	0.111837855	0.089470284	0.066421	5.333579	1.369863
2.44537	263.4	0.238284784	0.190627827	0.11654	4.98	0.925926
4.316361	210	0.230769231	0.161538462	0.116361	4.983639	1.149425
1.88228	103.7	0.073229292	0.065906363	0.044376	5.455624	1.075269
2.654152	75.9	0.059320047	0.041524033	0.022022	5.377978	1.041667
2.810961	158.2	0.091360591	0.082224532	0.084353	5.515647	1.041667
3.112653	122.4	0.051267016	0.056393717	0.044898	5.755102	1.369863
3.57	236.8	0.186031896	0.130222327	0.05125	5.14875	1.111111
3.756053	100.9	0.070732562	0.05658605	0.014674	5.085326	1.333333
2.607002	60.9	0.07017746	0.056141968	0.010054	4.998696	1.333333
3.61963	344.4	0.302503294	0.242002635	0.07507	5.62493	1.333333
2.531354	169.6	0.083840032	0.092224035	0.042271	4.966479	1.123596
2.733787	227.9	0.181912516	0.145530013	0.13279	5.76703	1.351351
3.974621	37	0.035594036	0.024915825	0.031818	5.368182	1.176471
3.01992	54.1	0.05111972	0.046007748	0.024502	5.675498	1.388889
3.28766	97	0.040149007	0.052193709	0.0726	5.2274	1.123596
4.43925	113.1	0.056501973	0.050851776	0.01593	4.99282	1.075269
3.41492	116.6	0.135313914	0.121782523	0.01924	5.28076	1.282051
2.2	53.9	0.066939891	0.066939891	n/a	5.7	1.190476
3.089474	200	0.249532127	0.174672489	0.090526	5.209474	1.149425
3.0102059	n/a	n/a	n/a	0.0734109	5.3265891	1.282051

code	Tot(Ca)	Ca+2		tot Ca x 10^4	ckd	ctrl
CKD2	0.0003778	0.0001922		3.778	1.922	
CKD4	0.000385	0.0001904		3.85	1.904	
CKD5	0.0003778	0.0002485		3.778	2.485	
CKD6	0.000385	0.0002137		3.85	2.137	
CKD7	0.0003778	0.0001786		3.778	1.786	
CKD11	0.0003778	0.0002985		3.778	2.985	
CKD13	0.0003707	0.0001612		3.707	1.612	
CKD14	0.0003778	0.0001824		3.778	1.824	
CKD15	0.0003564	0.0001442		3.564	1.442	
CKD18	0.000385	0.0002803		3.85	2.803	
CKD20	0.0004064	0.0001616		4.064	1.616	
CKD21	0.0003778	0.0001477		3.778	1.477	
CKD23	0.0003778	0.0002069		3.778	2.069	
CKD24	0.000385	0.0001406		3.85	1.406	
CKD25	0.0003992	0.0002185		3.992	2.185	
CKD26	0.0003992	0.0001317		3.992	1.317	
CKD27	0.000385	0.0001599		3.85	1.599	
CKD31	0.0004206	0.0001615		4.206	1.615	
CKD32	0.0003707	0.0002184		3.707	2.184	
CKD33	0.0003707	0.0001614		3.707	1.614	
CKD45	0.0003992	0.0001429		3.992	1.429	
CKD46	0.0003636	0.000234		3.636	2.34	
CKD49	0.0003707	0.0002449		3.707	2.449	
CKD50	0.000385	0.0003055		3.85	3.055	
CKD51	0.000385	0.0001751		3.85	1.751	
CKD55	0.0003636	0.0002886		3.636	2.886	
CKD59	0.0003992	0.0003198		3.992	3.198	
CKD62	0.0003707	0.0001481		3.707	1.481	
N2	0.0006986	0.000273		6.986		2.73
N3	0.0006612	0.0003005		6.612		3.005
N4	0.0006737	0.0002923		6.737		2.923
N6	0.0006612	0.0004387		6.612		4.387
N7	0.0006737	0.0003498		6.737		3.498
N8	0.0006487	0.0002115		6.487		2.115
N9	0.0006612	0.0003196		6.612		3.196
N10	0.0006737	0.0002511		6.737		2.511
N11	0.0006363	0.0002483		6.363		2.483
N13	0.0006363	0.0003971		6.363		3.971
N14	0.0006862	0.0003856		6.862		3.856
N15	0.0006737	0.000324		6.737		3.24
N16	0.0006986	0.0002357		6.986		2.357
N17	0.0007236	0.0003661		7.236		3.661
N18	0.0006737	0.0002108		6.737		2.108
N20	0.0006363	0.0002745		6.363		2.745
N21	0.0006249	0.0003876		6.249		3.876
N24	0.0007111	0.0003098		7.111		3.098
N25	0.0006249	0.0002527		6.249		2.527

N27	0.0007361	0.0002695	7.361		2.695
N29	0.0006737	0.0004337	6.737		4.337
N31	0.0007111	0.0003292	7.111		3.292
N32	0.0006612	0.000258	6.612		2.58
N33	0.0006249	0.000212	6.249		2.12
N35	0.0006612	0.0005046	6.612		5.046
N36	0.0007111	0.0003878	7.111		3.878
N38	0.0006612	0.0004652	6.612		4.652

CKD16	0.0004277	0.0001961	4.277	1.961	
CKD41	0.0003065	0.0001701	3.065	1.701	

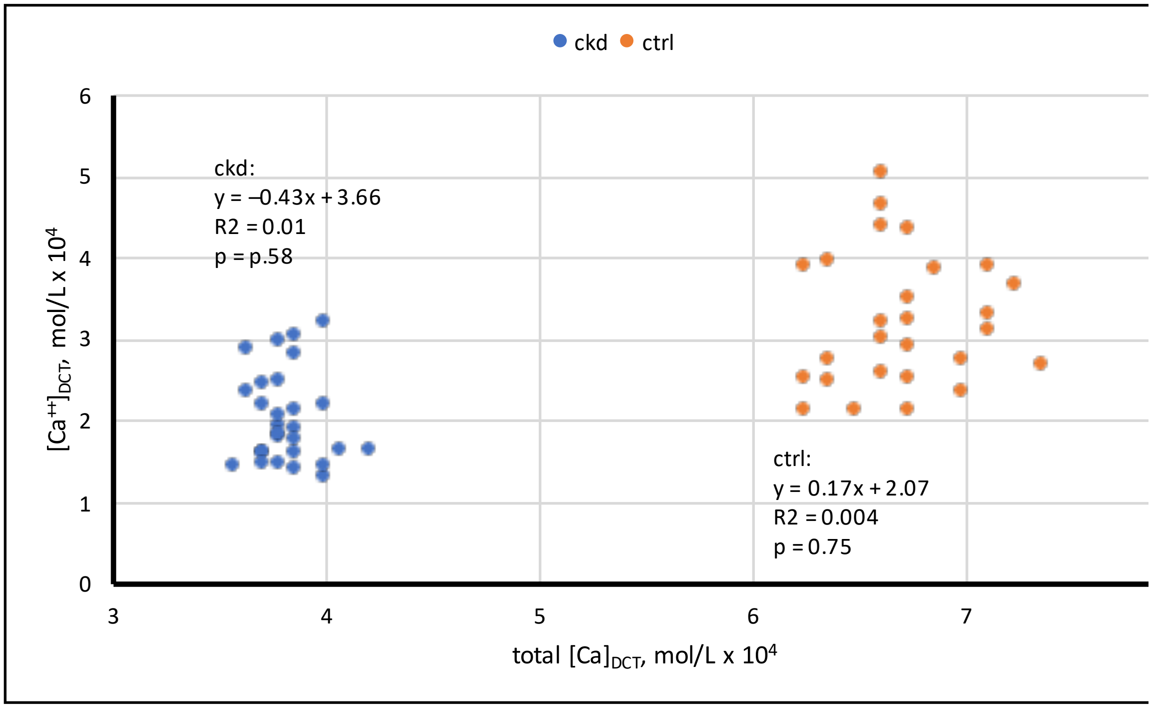

SUMMARY OUTPUT

Regression Statistics	
Multiple R	0.10835599
R Square	0.01174102
Adjusted R Sq	-0.0262689
Standard Erro	0.56808318
Observations	28

ANOVA

	df	*SS*	*MS*	*F*	*Significance F*
Regression	1	0.09968556	0.09968556	0.30889323	0.58311163
Residual	26	8.39068112	0.3227185		
Total	27	8.49036668			

	Coefficients	*Standard Error*	*t Stat*	*P-value*	*Lower 95%*
Intercept	3.65852005	2.94938403	1.24043529	0.22589207	-2.4040257
X Variable 1	-0.4286488	0.77125392	-0.5557816	0.58311163	-2.0139839

Regression Statistics		ctrl
Multiple R	0.06438722	
R Square	0.00414571	
Adjusted R Sq	-0.0356885	
Standard Erro	0.83116551	
Observations	27	

ANOVA

	df	*SS*	*MS*	*F*	*Significance F*
Regression	1	0.07189829	0.07189829	0.10407431	0.74967659
Residual	25	17.2709027	0.69083611		
Total	26	17.342801			

	Coefficients	*Standard Error*	*t Stat*	*P-value*	*Lower 95%*
Intercept	2.07289081	3.55328204	0.58337356	0.56486814	-5.2452305
X Variable 1	0.17065227	0.52898127	0.3226055	0.74967659	-0.918805

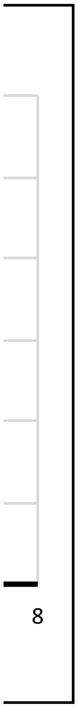

Upper 95%	Lower 95.0%	Upper 95.0%
9.72106575	-2.4040257	9.72106575
1.15668636	-2.0139839	1.15668636

Upper 95%	*Lower 95.0%*	*Upper 95.0%*
9.39101217	-5.2452305	9.39101217
1.26010958	-0.918805	1.26010958

code	Tot(P)	Ca+2	tot P x 10^3	ckd	ctrl
CKD2	0.00182	0.0001922	1.82	1.922	
CKD4	0.001852	0.0001904	1.852	1.904	
CKD5	0.001218	0.0002485	1.218	2.485	
CKD6	0.001549	0.0002137	1.549	2.137	
CKD7	0.002036	0.0001786	2.036	1.786	
CKD11	0.0008235	0.0002985	0.8235	2.985	
CKD13	0.002375	0.0001612	2.375	1.612	
CKD14	0.001973	0.0001824	1.973	1.824	
CKD15	0.002803	0.0001442	2.803	1.442	
CKD18	0.001003	0.0002803	1.003	2.803	
CKD20	0.002391	0.0001616	2.391	1.616	
CKD21	0.002718	0.0001477	2.718	1.477	
CKD23	0.001624	0.0002069	1.624	2.069	
CKD24	0.002935	0.0001406	2.935	1.406	
CKD25	0.001507	0.0002185	1.507	2.185	
CKD26	0.003251	0.0001317	3.251	1.317	
CKD27	0.002414	0.0001599	2.414	1.599	
CKD31	0.002402	0.0001615	2.402	1.615	
CKD32	0.001489	0.0002184	1.489	2.184	
CKD33	0.002371	0.0001614	2.371	1.614	
CKD45	0.002874	0.0001429	2.874	1.429	
CKD46	0.001331	0.000234	1.331	2.34	
CKD49	0.001242	0.0002449	1.242	2.449	
CKD50	0.0007575	0.0003055	0.7575	3.055	
CKD51	0.002103	0.0001751	2.103	1.751	
CKD55	0.0007141	0.0002886	0.7141	2.886	
CKD59	0.0006192	0.0003198	0.6192	3.198	
CKD62	0.002702	0.0001481	2.702	1.481	
N2	0.001258	0.000273	1.258		2.73
N3	0.001075	0.0003005	1.075		3.005
N4	0.001127	0.0002923	1.127		2.923
N6	0.0005899	0.0004387	0.5899		4.387
N7	0.0008665	0.0003498	0.8665		3.498
N8	0.001751	0.0002115	1.751		2.115
N9	0.0009826	0.0003196	0.9826		3.196
N10	0.001394	0.0002511	1.394		2.511
N11	0.001392	0.0002483	1.392		2.483
N13	0.0006849	0.0003971	0.6849		3.971
N14	0.0007527	0.0003856	0.7527		3.856
N15	0.0009714	0.000324	0.9714		3.24
N16	0.001539	0.0002357	1.539		2.357
N17	0.0008413	0.0003661	0.8413		3.661
N18	0.001776	0.0002108	1.776		2.108
N20	0.001206	0.0002745	1.206		2.745
N21	0.0007058	0.0003876	0.7058		3.876
N24	0.001062	0.0003098	1.062		3.098
N25	0.00135	0.0002527	1.35		2.527

N27	0.001305	0.0002695	1.305		2.695
N29	0.0006108	0.0004337	0.6108		4.337
N31	0.000974	0.0003292	0.974		3.292
N32	0.001335	0.000258	1.335		2.58
N33	0.001729	0.000212	1.729		2.12
N35	0.0004466	0.0005046	0.4466		5.046
N36	0.0007627	0.0003878	0.7627		3.878
N38	0.0005282	0.0004652	0.5282		4.652
CKD16	0.001797	0.0001961	1.797	1.961	
CKD41	0.002144	0.0001701	2.144	1.701	

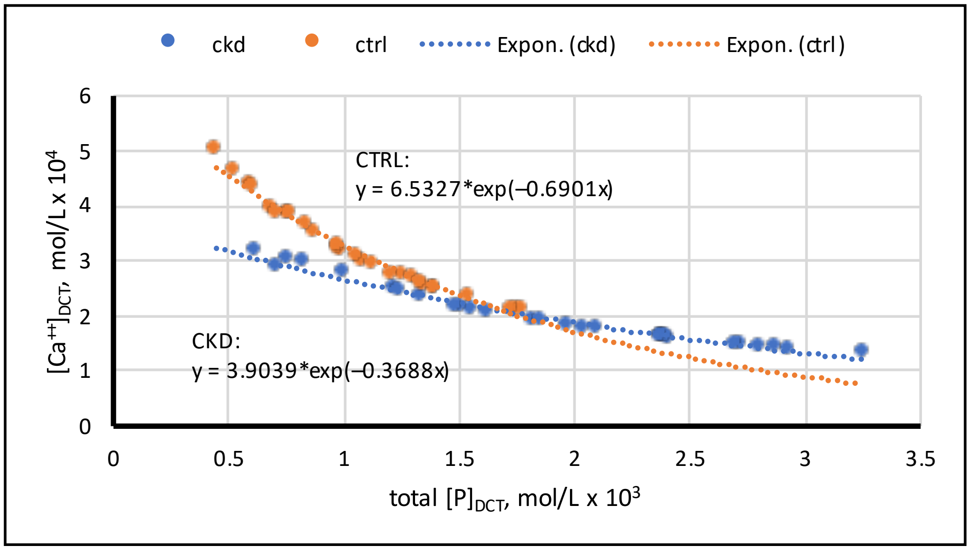

ckd
ctrl
Expon. (ckd)
Expon. (ctrl)
CTRL:
y = 6.5327*exp(–0.6901x)
CKD:
y = 3.9039*exp(–0.3688x)
[Ca++]DCT, mol/L x 10^4
total [P]DCT, mol/L x 10^3
0
1
2
3
4
5
6
0
0.5
1
1.5
2
2.5
3
3.5

code	Tot(Ca)	CaHPO4		tot Ca x 10^4	ckd	ctrl
CKD2	0.0003778	1.98E-05		3.778	1.979	
CKD4	0.000385	1.99E-05		3.85	1.989	
CKD5	0.0003778	1.74E-05		3.778	1.74	
CKD6	0.000385	1.88E-05		3.85	1.876	
CKD7	0.0003778	2.05E-05		3.778	2.053	
CKD11	0.0003778	1.46E-05		3.778	1.457	
CKD13	0.0003707	2.16E-05		3.707	2.162	
CKD14	0.0003778	2.03E-05		3.778	2.032	
CKD15	0.0003564	2.29E-05		3.564	2.286	
CKD18	0.000385	1.64E-05		3.85	1.638	
CKD20	0.0004064	2.16E-05		4.064	2.16	
CKD21	0.0003778	2.26E-05		3.778	2.259	
CKD23	0.0003778	1.91E-05		3.778	1.907	
CKD24	0.000385	2.32E-05		3.85	2.316	
CKD25	0.0003992	1.86E-05		3.992	1.856	
CKD26	0.0003992	2.39E-05		3.992	2.393	
CKD27	0.000385	2.17E-05		3.85	2.171	
CKD31	0.0004206	2.16E-05		4.206	2.16	
CKD32	0.0003707	1.86E-05		3.707	1.856	
CKD33	0.0003707	2.16E-05		3.707	2.161	
CKD45	0.0003992	2.30E-05		3.992	2.297	
CKD46	0.0003636	1.79E-05		3.636	1.793	
CKD49	0.0003707	1.75E-05		3.707	1.753	
CKD50	0.000385	1.37E-05		3.85	1.372	
CKD51	0.000385	2.07E-05		3.85	2.074	
CKD55	0.0003636	1.22E-05		3.636	1.224	
CKD59	0.0003992	1.18E-05		3.992	1.175	
CKD62	0.0003707	2.26E-05		3.707	2.256	
N2	0.0006986	1.66E-05		6.986		1.66
N3	0.0006612	1.58E-05		6.612		1.582
N4	0.0006737	1.60E-05		6.737		1.604
N6	0.0006612	1.31E-05		6.612		1.309
N7	0.0006737	1.47E-05		6.737		1.466
N8	0.0006487	1.89E-05		6.487		1.887
N9	0.0006612	1.53E-05		6.612		1.534
N10	0.0006737	1.73E-05		6.737		1.731
N11	0.0006363	1.74E-05		6.363		1.741
N13	0.0006363	1.38E-05		6.363		1.376
N14	0.0006862	1.40E-05		6.862		1.396
N15	0.0006737	1.52E-05		6.737		1.523
N16	0.0006986	1.79E-05		6.986		1.787
N17	0.0007236	1.43E-05		7.236		1.433
N18	0.0006737	1.89E-05		6.737		1.89
N20	0.0006363	1.66E-05		6.363		1.655
N21	0.0006249	1.39E-05		6.249		1.393
N24	0.0007111	1.56E-05		7.111		1.558
N25	0.0006249	1.73E-05		6.249		1.726

N27	0.0007361	1.67E-05	7.361		1.671
N29	0.0006737	1.32E-05	6.737		1.317
N31	0.0007111	1.51E-05	7.111		1.511
N32	0.0006612	1.71E-05	6.612		1.708
N33	0.0006249	1.88E-05	6.249		1.884
N35	0.0006612	1.22E-05	6.612		1.221
N36	0.0007111	1.39E-05	7.111		1.392
N38	0.0006612	1.27E-05	6.612		1.271
CKD16	0.0004277	1.96E-05	4.277	1.959	
CKD41	0.0003065	2.10E-05	3.065	2.104	

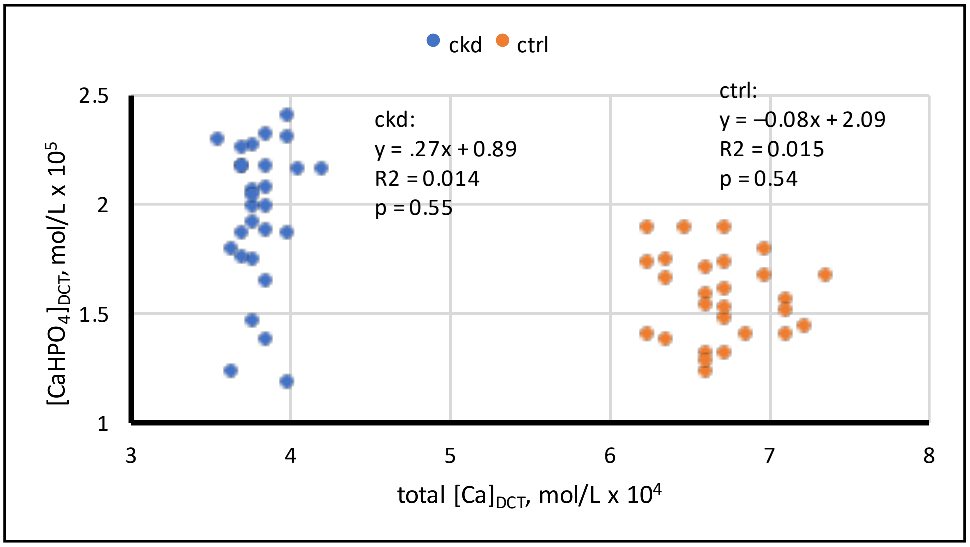

SUMMARY OUTPUT ckd

Regression Statistics	
Multiple R	0.11778831
R Square	0.01387409
Adjusted R Sq	-0.0240538
Standard Erro	0.3343503
Observations	28

ANOVA

	df	*SS*	*MS*	*F*	*Significance F*
Regression	1	0.04089298	0.04089298	0.3658014	0.55054432
Residual	26	2.90654312	0.11179012		
Total	27	2.94743611			

	Coefficients	*Standard Error*	*t Stat*	*P-value*	*Lower 95%*
Intercept	0.89348437	1.73588561	0.51471385	0.61110113	-2.6746796
X Variable 1	0.27454266	0.4539282	0.60481518	0.55054432	-0.6585201

SUMMARY OUTPUT ctrl

Regression Statistics	
Multiple R	0.12458859
R Square	0.01552232
Adjusted R Sq	-0.0238568
Standard Erro	0.19683955
Observations	27

ANOVA

	df	*SS*	*MS*	*F*	*Significance F*

Regression	1	0.01527268	0.01527268	0.39417645	0.53580562
Residual	25	0.96864517	0.03874581		
Total	26	0.98391785			

	Coefficients	*Standard Error*	*t Stat*	*P-value*	*Lower 95%*
Intercept	2.09171374	0.84150078	2.48569436	0.01997371	0.35861045
X Variable 1	-0.0786521	0.12527521	-0.6278347	0.53580562	-0.3366612

Upper 95%	*Lower 95.0%*	*Upper 95.0%*
4.46164833	-2.6746796	4.46164833
1.20760543	-0.6585201	1.20760543

Upper 95%	*Lower 95.0%*	*Upper 95.0%*
3.82481703	0.35861045	3.82481703
0.17935699	-0.3366612	0.17935699

code	Tot(P)	CaHPO4	tot P x 10^3	ckd	ctrl
CKD2	0.00182	1.98E-05	1.82	1.979	
CKD4	0.001852	1.99E-05	1.852	1.989	
CKD5	0.001218	1.74E-05	1.218	1.74	
CKD6	0.001549	1.88E-05	1.549	1.876	
CKD7	0.002036	2.05E-05	2.036	2.053	
CKD11	0.0008235	1.46E-05	0.8235	1.457	
CKD13	0.002375	2.16E-05	2.375	2.162	
CKD14	0.001973	2.03E-05	1.973	2.032	
CKD15	0.002803	2.29E-05	2.803	2.286	
CKD18	0.001003	1.64E-05	1.003	1.638	
CKD20	0.002391	2.16E-05	2.391	2.16	
CKD21	0.002718	2.26E-05	2.718	2.259	
CKD23	0.001624	1.91E-05	1.624	1.907	
CKD24	0.002935	2.32E-05	2.935	2.316	
CKD25	0.001507	1.86E-05	1.507	1.856	
CKD26	0.003251	2.39E-05	3.251	2.393	
CKD27	0.002414	2.17E-05	2.414	2.171	
CKD31	0.002402	2.16E-05	2.402	2.16	
CKD32	0.001489	1.86E-05	1.489	1.856	
CKD33	0.002371	2.16E-05	2.371	2.161	
CKD45	0.002874	2.30E-05	2.874	2.297	
CKD46	0.001331	1.79E-05	1.331	1.793	
CKD49	0.001242	1.75E-05	1.242	1.753	
CKD50	0.0007575	1.37E-05	0.7575	1.372	
CKD51	0.002103	2.07E-05	2.103	2.074	
CKD55	0.0007141	1.22E-05	0.7141	1.224	
CKD59	0.0006192	1.18E-05	0.6192	1.175	
CKD62	0.002702	2.26E-05	2.702	2.256	
N2	0.001258	1.66E-05	1.258		1.66
N3	0.001075	1.58E-05	1.075		1.582
N4	0.001127	1.60E-05	1.127		1.604
N6	0.0005899	1.31E-05	0.5899		1.309
N7	0.0008665	1.47E-05	0.8665		1.466
N8	0.001751	1.89E-05	1.751		1.887
N9	0.0009826	1.53E-05	0.9826		1.534
N10	0.001394	1.73E-05	1.394		1.731
N11	0.001392	1.74E-05	1.392		1.741
N13	0.0006849	1.38E-05	0.6849		1.376
N14	0.0007527	1.40E-05	0.7527		1.396
N15	0.0009714	1.52E-05	0.9714		1.523
N16	0.001539	1.79E-05	1.539		1.787
N17	0.0008413	1.43E-05	0.8413		1.433
N18	0.001776	1.89E-05	1.776		1.89
N20	0.001206	1.66E-05	1.206		1.655
N21	0.0007058	1.39E-05	0.7058		1.393
N24	0.001062	1.56E-05	1.062		1.558
N25	0.00135	1.73E-05	1.35		1.726

N27	0.001305	1.67E-05	1.305		1.671
N29	0.0006108	1.32E-05	0.6108		1.317
N31	0.000974	1.51E-05	0.974		1.511
N32	0.001335	1.71E-05	1.335		1.708
N33	0.001729	1.88E-05	1.729		1.884
N35	0.0004466	1.22E-05	0.4466		1.221
N36	0.0007627	1.39E-05	0.7627		1.392
N38	0.0005282	1.27E-05	0.5282		1.271
CKD16	0.001797	1.96E-05	1.797	1.959	
CKD41	0.002144	2.10E-05	2.144	2.104	

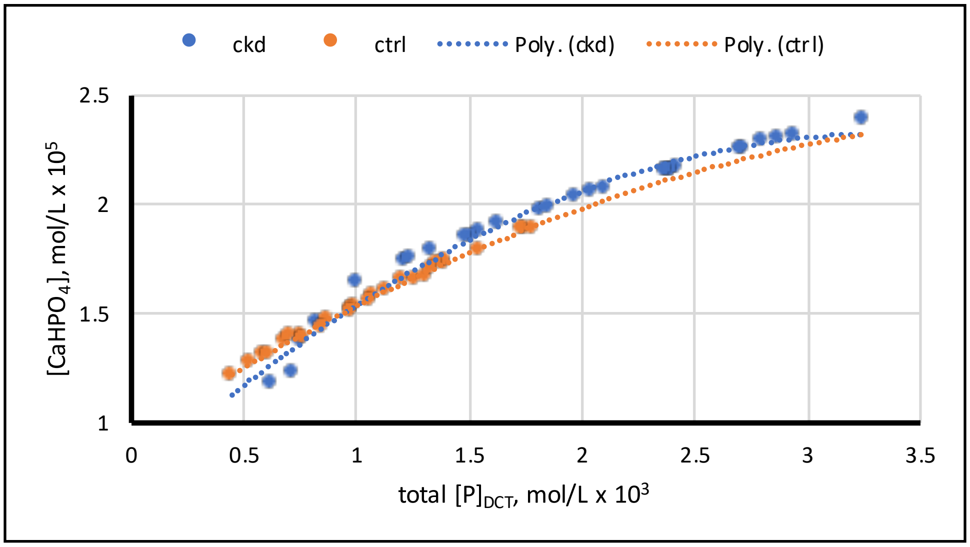

ckd
ctrl
Poly. (ckd)
Poly. (ctrl)
[CaHPO4], mol/L x 10^5
total [P]DCT, mol/L x 10^3
2.5
2
1.5
1
0
0.5
1
1.5
2
2.5
3
3.5

code	CaHPO4	Ca+2		CaHPO4 x 10^5	ckd	ctrl
CKD2	1.98E-05	0.0001922		1.979	1.922	
CKD4	1.99E-05	0.0001904		1.989	1.904	
CKD5	1.74E-05	0.0002485		1.74	2.485	
CKD6	1.88E-05	0.0002137		1.876	2.137	
CKD7	2.05E-05	0.0001786		2.053	1.786	
CKD11	1.46E-05	0.0002985		1.457	2.985	
CKD13	2.16E-05	0.0001612		2.162	1.612	
CKD14	2.03E-05	0.0001824		2.032	1.824	
CKD15	2.29E-05	0.0001442		2.286	1.442	
CKD18	1.64E-05	0.0002803		1.638	2.803	
CKD20	2.16E-05	0.0001616		2.16	1.616	
CKD21	2.26E-05	0.0001477		2.259	1.477	
CKD23	1.91E-05	0.0002069		1.907	2.069	
CKD24	2.32E-05	0.0001406		2.316	1.406	
CKD25	1.86E-05	0.0002185		1.856	2.185	
CKD26	2.39E-05	0.0001317		2.393	1.317	
CKD27	2.17E-05	0.0001599		2.171	1.599	
CKD31	2.16E-05	0.0001615		2.16	1.615	
CKD32	1.86E-05	0.0002184		1.856	2.184	
CKD33	2.16E-05	0.0001614		2.161	1.614	
CKD45	2.30E-05	0.0001429		2.297	1.429	
CKD46	1.79E-05	0.000234		1.793	2.34	
CKD49	1.75E-05	0.0002449		1.753	2.449	
CKD50	1.37E-05	0.0003055		1.372	3.055	
CKD51	2.07E-05	0.0001751		2.074	1.751	
CKD55	1.22E-05	0.0002886		1.224	2.886	
CKD59	1.18E-05	0.0003198		1.175	3.198	
CKD62	2.26E-05	0.0001481		2.256	1.481	
N2	1.66E-05	0.000273		1.66		2.73
N3	1.58E-05	0.0003005		1.582		3.005
N4	1.60E-05	0.0002923		1.604		2.923
N6	1.31E-05	0.0004387		1.309		4.387
N7	1.47E-05	0.0003498		1.466		3.498
N8	1.89E-05	0.0002115		1.887		2.115
N9	1.53E-05	0.0003196		1.534		3.196
N10	1.73E-05	0.0002511		1.731		2.511
N11	1.74E-05	0.0002483		1.741		2.483
N13	1.38E-05	0.0003971		1.376		3.971
N14	1.40E-05	0.0003856		1.396		3.856
N15	1.52E-05	0.000324		1.523		3.24
N16	1.79E-05	0.0002357		1.787		2.357
N17	1.43E-05	0.0003661		1.433		3.661
N18	1.89E-05	0.0002108		1.89		2.108
N20	1.66E-05	0.0002745		1.655		2.745
N21	1.39E-05	0.0003876		1.393		3.876
N24	1.56E-05	0.0003098		1.558		3.098
N25	1.73E-05	0.0002527		1.726		2.527

N27	1.67E-05	0.0002695	1.671		2.695
N29	1.32E-05	0.0004337	1.317		4.337
N31	1.51E-05	0.0003292	1.511		3.292
N32	1.71E-05	0.000258	1.708		2.58
N33	1.88E-05	0.000212	1.884		2.12
N35	1.22E-05	0.0005046	1.221		5.046
N36	1.39E-05	0.0003878	1.392		3.878
N38	1.27E-05	0.0004652	1.271		4.652
CKD16	1.96E-05	0.0001961	1.959	1.961	
CKD41	2.10E-05	0.0001701	2.104	1.701	

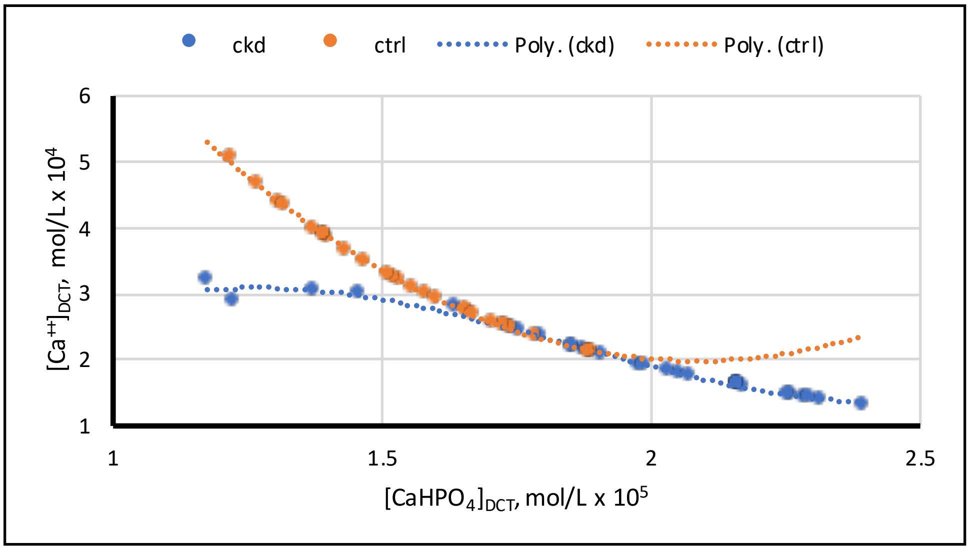

ckd
ctrl
Poly. (ckd)
Poly. (ctrl)
6
5
4
3
2
1
1
1.5
2
2.5
[Ca++]DCT, mol/L x 10^4
[CaHPO4]DCT, mol/L x 10^5

code	CaCitric	Ca+2	Cacit x 10^5	ckd	ctrl
CKD2	2.47E-05	0.0001922	2.474	1.922	
CKD4	2.46E-05	0.0001904	2.459	1.904	
CKD5	2.89E-05	0.0002485	2.888	2.485	
CKD6	2.64E-05	0.0002137	2.642	2.137	
CKD7	2.36E-05	0.0001786	2.362	1.786	
CKD11	3.20E-05	0.0002985	3.196	2.985	
CKD13	2.21E-05	0.0001612	2.209	1.612	
CKD14	2.39E-05	0.0001824	2.393	1.824	
CKD15	2.05E-05	0.0001442	2.05	1.442	
CKD18	3.09E-05	0.0002803	3.09	2.803	
CKD20	2.21E-05	0.0001616	2.212	1.616	
CKD21	2.08E-05	0.0001477	2.083	1.477	
CKD23	2.59E-05	0.0002069	2.59	2.069	
CKD24	2.01E-05	0.0001406	2.014	1.406	
CKD25	2.68E-05	0.0002185	2.677	2.185	
CKD26	1.92E-05	0.0001317	1.924	1.317	
CKD27	2.20E-05	0.0001599	2.197	1.599	
CKD31	2.21E-05	0.0001615	2.211	1.615	
CKD32	2.68E-05	0.0002184	2.677	2.184	
CKD33	2.21E-05	0.0001614	2.21	1.614	
CKD45	2.04E-05	0.0001429	2.036	1.429	
CKD46	2.79E-05	0.000234	2.789	2.34	
CKD49	2.86E-05	0.0002449	2.863	2.449	
CKD50	3.24E-05	0.0003055	3.235	3.055	
CKD51	2.33E-05	0.0001751	2.332	1.751	
CKD55	3.14E-05	0.0002886	3.136	2.886	
CKD59	3.31E-05	0.0003198	3.314	3.198	
CKD62	2.09E-05	0.0001481	2.087	1.481	
N2	3.05E-05	0.000273	3.045		2.73
N3	3.21E-05	0.0003005	3.208		3.005
N4	3.16E-05	0.0002923	3.16		2.923
N6	3.86E-05	0.0004387	3.862		4.387
N7	3.47E-05	0.0003498	3.47		3.498
N8	2.63E-05	0.0002115	2.625		2.115
N9	3.31E-05	0.0003196	3.314		3.196
N10	2.91E-05	0.0002511	2.905		2.511
N11	2.89E-05	0.0002483	2.886		2.483
N13	3.69E-05	0.0003971	3.69		3.971
N14	3.64E-05	0.0003856	3.639		3.856
N15	3.34E-05	0.000324	3.337		3.24
N16	2.80E-05	0.0002357	2.8		2.357
N17	3.55E-05	0.0003661	3.549		3.661
N18	2.62E-05	0.0002108	2.619		2.108
N20	3.05E-05	0.0002745	3.054		2.745
N21	3.65E-05	0.0003876	3.648		3.876
N24	3.26E-05	0.0003098	3.26		3.098
N25	2.92E-05	0.0002527	2.915		2.527

N27	3.02E-05	0.0002695	3.023	2.695
N29	3.84E-05	0.0004337	3.842	4.337
N31	3.37E-05	0.0003292	3.365	3.292
N32	2.95E-05	0.000258	2.95	2.58
N33	2.63E-05	0.000212	2.628	2.12
N35	4.10E-05	0.0005046	4.102	5.046
N36	3.65E-05	0.0003878	3.649	3.878
N38	3.96E-05	0.0004652	3.963	4.652

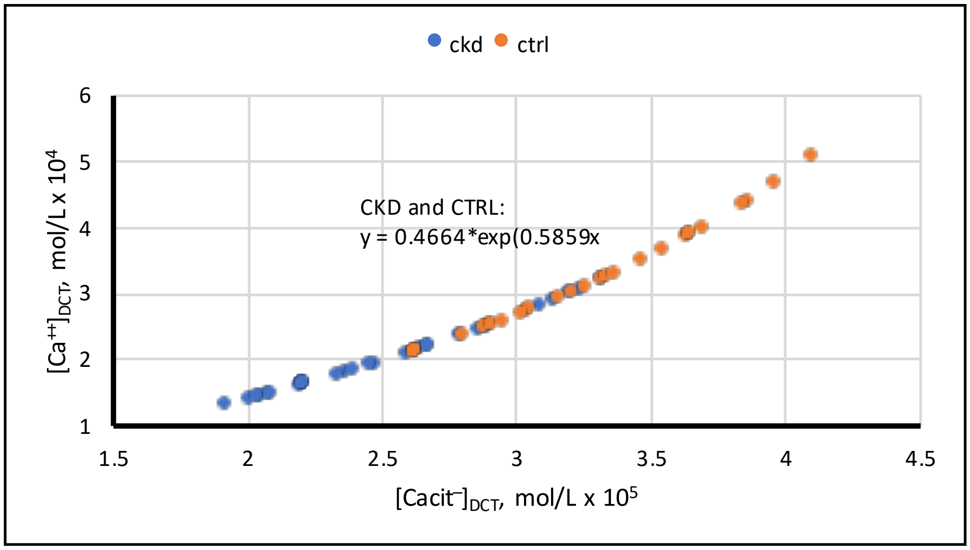

ckd
ctrl
CKD and CTRL:
y = 0.4664*exp(0.5859x
[Ca++]DCT, mol/L x 10^4
[Cacit−]DCT, mol/L x 10^5
1
2
3
4
5
6
1.5
2
2.5
3
3.5
4
4.5

code	Tot(Ca)	pth 1-84	tot Ca x 10^4	ckd	ctrl
CKD2	0.0003778	158	3.778	158	
CKD4	0.000385	41	3.85	41	
CKD5	0.0003778	59	3.778	59	
CKD6	0.000385	54	3.85	54	
CKD7	0.0003778	129	3.778	129	
CKD11	0.0003778	50	3.778	50	
CKD13	0.0003707	56	3.707	56	
CKD14	0.0003778	145	3.778	145	
CKD15	0.0003564	156	3.564	156	
CKD18	0.000385	67	3.85	67	
CKD20	0.0004064	182	4.064	182	
CKD21	0.0003778	126	3.778	126	
CKD23	0.0003778	63	3.778	63	
CKD24	0.000385	103	3.85	103	
CKD25	0.0003992	42	3.992	42	
CKD26	0.0003992	69	3.992	69	
CKD27	0.000385	72	3.85	72	
CKD31	0.0004206	31	4.206	31	
CKD32	0.0003707	91	3.707	91	
CKD33	0.0003707	54	3.707	54	
CKD45	0.0003992	127	3.992	127	
CKD46	0.0003636	39	3.636	39	
CKD49	0.0003707	48	3.707	48	
CKD50	0.000385	48	3.85	48	
CKD51	0.000385	73	3.85	73	
CKD55	0.0003636	32	3.636	32	
CKD59	0.0003992	28	3.992	28	
CKD62	0.0003707	178	3.707	178	
N2	0.0006986	21	6.986		21
N3	0.0006612	44	6.612		44
N4	0.0006737	45	6.737		45
N6	0.0006612	31	6.612		31
N7	0.0006737	18	6.737		18
N8	0.0006487	24	6.487		24
N9	0.0006612	36	6.612		36
N10	0.0006737	22	6.737		22
N11	0.0006363	60	6.363		60
N13	0.0006363	28	6.363		28
N14	0.0006862	20	6.862		20
N15	0.0006737	34	6.737		34
N16	0.0006986	17	6.986		17
N17	0.0007236	29	7.236		29
N18	0.0006737	25	6.737		25
N20	0.0006363	19	6.363		19
N21	0.0006249	26	6.249		26
N24	0.0007111	21	7.111		21
N25	0.0006249	41	6.249		41

N27	0.0007361	16	7.361	16
N29	0.0006737	23	6.737	23
N31	0.0007111	19	7.111	19
N32	0.0006612	24	6.612	24
N33	0.0006249	65	6.249	65
N35	0.0006612	24	6.612	24
N36	0.0007111	25	7.111	25
N38	0.0006612	26	6.612	26

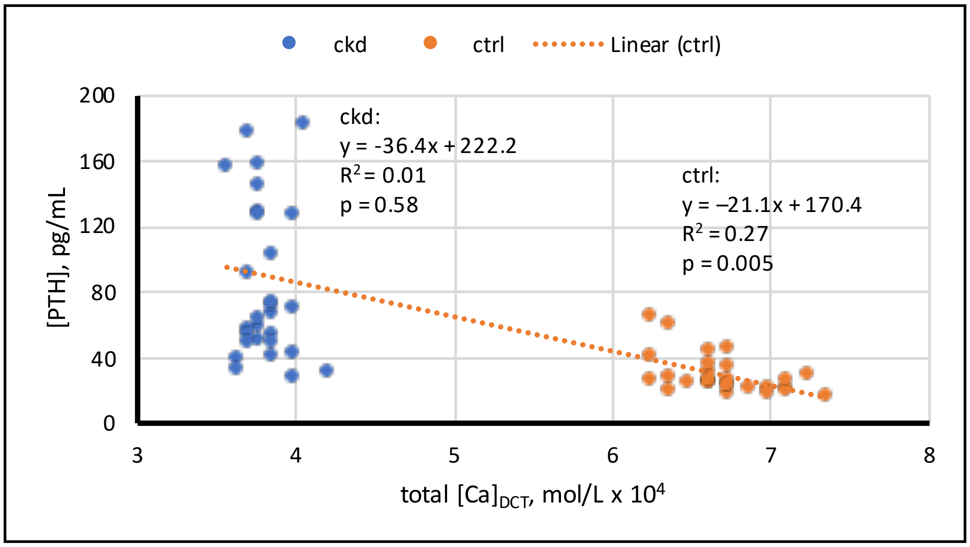

SUMMARY OUTPUT ckd

Regression Statistics	
Multiple R	0.1084377
R Square	0.01175874
Adjusted R Sq	-0.0262505
Standard Erro	48.2692304
Observations	28

ANOVA

	df	*SS*	*MS*	*F*	*Significance F*
Regression	1	720.794931	720.794931	0.30936486	0.5828259
Residual	26	60577.8836	2329.9186		
Total	27	61298.6786			

	Coefficients	*Standard Error*	*t Stat*	*P-value*	*Lower 95%*
Intercept	222.188442	250.605019	0.88660811	0.38341765	-292.93755
X Variable 1	-36.449478	65.5323621	-0.5562058	0.5828259	-171.15318

SUMMARY OUTPUT ctrl

Regression Statistics	
Multiple R	0.52245748
R Square	0.27296181
Adjusted R Sq	0.24388029
Standard Erro	10.8043163
Observations	27

ANOVA

	df	*SS*	*MS*	*F*	*Significance F*
Regression	1	1095.66872	1095.66872	9.38608932	0.00517895

Residual	25	2918.33128	116.733251
Total	26	4014	

	Coefficients	*Standard Error*	*t Stat*	*P-value*	*Lower 95%*
Intercept	170.364805	46.1890955	3.68842046	0.00109768	75.2365821
X Variable 1	-21.066501	6.87622484	-3.0636725	0.00517895	-35.228351

Upper 95%	*Lower 95.0%*	*Upper 95.0%*
737.314436	-292.93755	737.314436
98.2542217	-171.15318	98.2542217

Upper 95%	*Lower 95.0%*	*Upper 95.0%*
265.493028	75.2365821	265.493028
-6.9046509	-35.228351	-6.9046509

code	Ca+2	pth 1-84		Ca++ x 10^4	ckd	ctrl
CKD2	0.0001922	158		1.922	158	
CKD4	0.0001904	41		1.904	41	
CKD5	0.0002485	59		2.485	59	
CKD6	0.0002137	54		2.137	54	
CKD7	0.0001786	129		1.786	129	
CKD11	0.0002985	50		2.985	50	
CKD13	0.0001612	56		1.612	56	
CKD14	0.0001824	145		1.824	145	
CKD15	0.0001442	156		1.442	156	
CKD18	0.0002803	67		2.803	67	
CKD20	0.0001616	182		1.616	182	
CKD21	0.0001477	126		1.477	126	
CKD23	0.0002069	63		2.069	63	
CKD24	0.0001406	103		1.406	103	
CKD25	0.0002185	42		2.185	42	
CKD26	0.0001317	69		1.317	69	
CKD27	0.0001599	72		1.599	72	
CKD31	0.0001615	31		1.615	31	
CKD32	0.0002184	91		2.184	91	
CKD33	0.0001614	54		1.614	54	
CKD45	0.0001429	127		1.429	127	
CKD46	0.000234	39		2.34	39	
CKD49	0.0002449	48		2.449	48	
CKD50	0.0003055	48		3.055	48	
CKD51	0.0001751	73		1.751	73	
CKD55	0.0002886	32		2.886	32	
CKD59	0.0003198	28		3.198	28	
CKD62	0.0001481	178		1.481	178	
N2	0.000273	21		2.73		21
N3	0.0003005	44		3.005		44
N4	0.0002923	45		2.923		45
N6	0.0004387	31		4.387		31
N7	0.0003498	18		3.498		18
N8	0.0002115	24		2.115		24
N9	0.0003196	36		3.196		36
N10	0.0002511	22		2.511		22
N11	0.0002483	60		2.483		60
N13	0.0003971	28		3.971		28
N14	0.0003856	20		3.856		20
N15	0.000324	34		3.24		34
N16	0.0002357	17		2.357		17
N17	0.0003661	29		3.661		29
N18	0.0002108	25		2.108		25
N20	0.0002745	19		2.745		19
N21	0.0003876	26		3.876		26
N24	0.0003098	21		3.098		21
N25	0.0002527	41		2.527		41

N27	0.0002695	16	2.695	16
N29	0.0004337	23	4.337	23
N31	0.0003292	19	3.292	19
N32	0.000258	24	2.58	24
N33	0.000212	65	2.12	65
N35	0.0005046	24	5.046	24
N36	0.0003878	25	3.878	25
N38	0.0004652	26	4.652	26

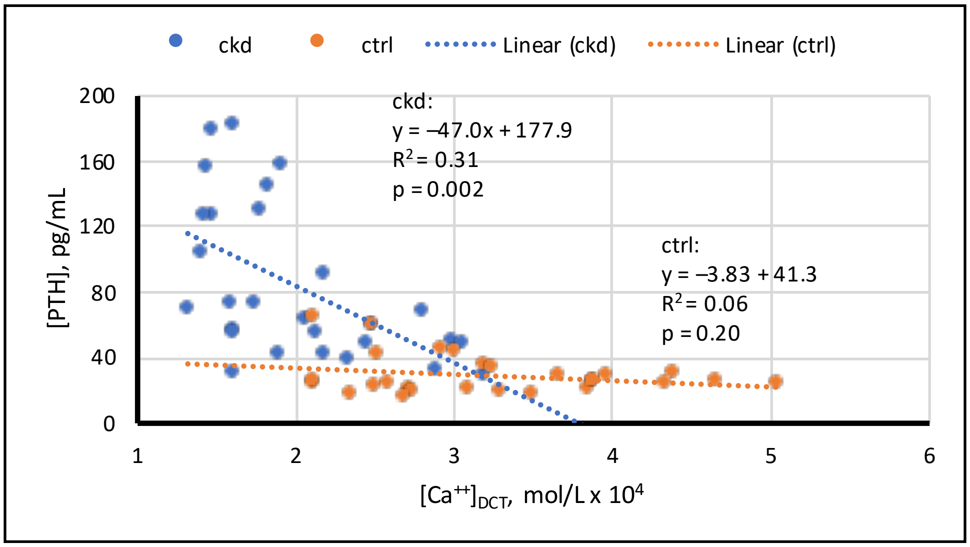

SUMMARY OUTPUT ckd

Regression Statistics	
Multiple R	0.55313447
R Square	0.30595774
Adjusted R Sq	0.27926381
Standard Erro	40.45124
Observations	28

ANOVA

	df	*SS*	*MS*	*F*	*Significance F*
Regression	1	18754.8052	18754.8052	11.4616958	0.00226604
Residual	26	42543.8733	1636.30282		
Total	27	61298.6786			

	Coefficients	*Standard Error*	*t Stat*	*P-value*	*Lower 95%*
Intercept	177.850271	29.0712728	6.11773254	1.8186E-06	118.093414
X Variable 1	-46.99948	13.8825299	-3.3855126	0.00226604	-75.535429

Regression Statistics	ctrl
Multiple R	0.25201193
R Square	0.06351001
Adjusted R Sq	0.02605041
Standard Erro	12.2622524
Observations	27

ANOVA

	df	*SS*	*MS*	*F*	*Significance F*
Regression	1	254.929182	254.929182	1.69542684	0.20475675

Residual	25	3759.07082	150.362833
Total	26	4014	

	Coefficients	*Standard Error*	*t Stat*	*P-value*	*Lower 95%*
Intercept	41.3378985	9.76493218	4.23330114	0.00027161	21.2266443
X Variable 1	-3.8339828	2.9444937	-1.3020856	0.20475675	-9.8982811

Upper 95%	*Lower 95.0%*	*Upper 95.0%*
237.607128	118.093414	237.607128
-18.463531	-75.535429	-18.463531

Upper 95%	*Lower 95.0%*	*Upper 95.0%*
61.4491528	21.2266443	61.4491528
2.23031553	-9.8982811	2.23031553

code	Ca++ x 10^4	pth		Ca++ x 10^4	log Ca++ x 104	mean
CKD2	1.922	158		1.922	0.283753383	0.39
CKD4	1.904	41		1.904	0.279666944	0.39
CKD5	2.485	59		2.485	0.395326393	0.39
CKD6	2.137	54		2.137	0.329804522	0.39
CKD7	1.786	129		1.786	0.251881455	0.39
CKD11	2.985	50		2.985	0.474944335	0.39
CKD13	1.612	56		1.612	0.207365037	0.39
CKD14	1.824	145		1.824	0.261024834	0.39
CKD15	1.442	156		1.442	0.15896526	0.39
CKD18	2.803	67		2.803	0.447623098	0.39
CKD20	1.616	182		1.616	0.208441356	0.39
CKD21	1.477	126		1.477	0.169380495	0.39
CKD23	2.069	63		2.069	0.315760491	0.39
CKD24	1.406	103		1.406	0.147985321	0.39
CKD25	2.185	42		2.185	0.339451441	0.39
CKD26	1.317	69		1.317	0.119585775	0.39
CKD27	1.599	72		1.599	0.203848464	0.39
CKD31	1.615	31		1.615	0.208172527	0.39
CKD32	2.184	91		2.184	0.339252634	0.39
CKD33	1.614	54		1.614	0.20790353	0.39
CKD45	1.429	127		1.429	0.155032229	0.39
CKD46	2.34	39		2.34	0.369215857	0.39
CKD49	2.449	48		2.449	0.388988785	0.39
CKD50	3.055	48		3.055	0.485011215	0.39
CKD51	1.751	73		1.751	0.243286146	0.39
CKD55	2.886	32		2.886	0.460296327	0.39
CKD59	3.198	28		3.198	0.504878459	0.39
CKD62	1.481	178		1.481	0.170555059	0.39
N2	2.73	21		2.73	0.436162647	0.39
N3	3.005	44		3.005	0.477844476	0.39
N4	2.923	45		2.923	0.465828815	0.39
N6	4.387	31		4.387	0.642167634	0.39
N7	3.498	18		3.498	0.543819805	0.39
N8	2.115	24		2.115	0.325310372	0.39
N9	3.196	36		3.196	0.504606771	0.39
N10	2.511	22		2.511	0.399846713	0.39
N11	2.483	60		2.483	0.39497672	0.39
N13	3.971	28		3.971	0.598899887	0.39
N14	3.856	20		3.856	0.586137025	0.39
N15	3.24	34		3.24	0.51054501	0.39
N16	2.357	17		2.357	0.372359583	0.39
N17	3.661	29		3.661	0.563599729	0.39
N18	2.108	25		2.108	0.323870607	0.39
N20	2.745	19		2.745	0.438542349	0.39
N21	3.876	26		3.876	0.588383768	0.39
N24	3.098	21		3.098	0.491081413	0.39
N25	2.527	41		2.527	0.402605242	0.39

N27	2.695	16	2.695	0.43055877	0.39
N29	4.337	23	4.337	0.637189422	0.39
N31	3.292	19	3.292	0.517459827	0.39
N32	2.58	24	2.58	0.411619706	0.39
N33	2.12	65	2.12	0.326335861	0.39
N35	5.046	24	5.046	0.702947246	0.39
N36	3.878	25	3.878	0.588607805	0.39
N38	4.652	26	4.652	0.667639706	0.39

Column1	
Mean	0.39047906
Standard Erro	0.020426707
Median	0.395326393
Mode	#N/A
Standard Devi	0.151488511
Sample Variar	0.022948769
Kurtosis	-0.8599047
Skewness	0.058257104
Range	0.583361471
Minimum	0.119585775
Maximum	0.702947246
Sum	21.47634828
Count	55

SD	standardized Ca++ x 104	pth	logpth	mean	SD
0.15	-0.708310778	158	2.19865709	1.65	0.29
0.15	-0.735553706	41	1.61278386	1.65	0.29
0.15	0.035509287	59	1.77085201	1.65	0.29
0.15	-0.401303186	54	1.73239376	1.65	0.29
0.15	-0.920790303	129	2.11058971	1.65	0.29
0.15	0.56629557	50	1.69897	1.65	0.29
0.15	-1.217566417	56	1.74818803	1.65	0.29
0.15	-0.85983444	145	2.161368	1.65	0.29
0.15	-1.540231597	156	2.1931246	1.65	0.29
0.15	0.384153985	67	1.8260748	1.65	0.29
0.15	-1.210390957	182	2.26007139	1.65	0.29
0.15	-1.470796698	126	2.10037055	1.65	0.29
0.15	-0.494930062	63	1.79934055	1.65	0.29
0.15	-1.613431195	103	2.01283722	1.65	0.29
0.15	-0.336990391	42	1.62324929	1.65	0.29
0.15	-1.8027615	69	1.83884909	1.65	0.29
0.15	-1.241010242	72	1.8573325	1.65	0.29
0.15	-1.212183156	31	1.49136169	1.65	0.29
0.15	-0.338315773	91	1.95904139	1.65	0.29
0.15	-1.213976464	54	1.73239376	1.65	0.29
0.15	-1.566451808	127	2.10380372	1.65	0.29
0.15	-0.138560951	39	1.59106461	1.65	0.29
0.15	-0.006741433	48	1.68124124	1.65	0.29
0.15	0.633408097	48	1.68124124	1.65	0.29
0.15	-0.978092359	73	1.86332286	1.65	0.29
0.15	0.468642178	32	1.50514998	1.65	0.29
0.15	0.765856396	28	1.44715803	1.65	0.29
0.15	-1.462966277	178	2.25042	1.65	0.29
0.15	0.30775098	21	1.32221929	1.65	0.29
0.15	0.585629842	44	1.64345268	1.65	0.29
0.15	0.505525436	45	1.65321251	1.65	0.29
0.15	1.681117563	31	1.49136169	1.65	0.29
0.15	1.025465368	18	1.25527251	1.65	0.29
0.15	-0.431264189	24	1.38021124	1.65	0.29
0.15	0.764045138	36	1.5563025	1.65	0.29
0.15	0.065644751	22	1.34242268	1.65	0.29
0.15	0.03317813	60	1.77815125	1.65	0.29
0.15	1.392665914	28	1.44715803	1.65	0.29
0.15	1.307580168	20	1.30103	1.65	0.29
0.15	0.803633401	34	1.53147892	1.65	0.29
0.15	-0.117602783	17	1.23044892	1.65	0.29
0.15	1.157331526	29	1.462398	1.65	0.29
0.15	-0.440862623	25	1.39794001	1.65	0.29
0.15	0.323615659	19	1.2787536	1.65	0.29
0.15	1.322558456	26	1.41497335	1.65	0.29
0.15	0.673876089	21	1.32221929	1.65	0.29
0.15	0.084034946	41	1.61278386	1.65	0.29

0.15	0.270391797	16	1.20411998	1.65	0.29
0.15	1.647929481	23	1.36172784	1.65	0.29
0.15	0.849732177	19	1.2787536	1.65	0.29
0.15	0.144131373	24	1.38021124	1.65	0.29
0.15	-0.424427594	65	1.81291336	1.65	0.29
0.15	2.086314975	24	1.38021124	1.65	0.29
0.15	1.324052032	25	1.39794001	1.65	0.29
0.15	1.850931374	26	1.41497335	1.65	0.29

Column1	
Mean	1.64552531
Standard Erro	0.03975921
Median	1.61278386
Mode	1.38021124
Standard Devi	0.29486219
Sample Varian	0.08694371
Kurtosis	-0.6740973
Skewness	0.54246903
Range	1.05595141
Minimum	1.20411998
Maximum	2.26007139
Sum	90.5038919
Count	55

standardized logPTH	standardized Ca++ x 104	standardized logPTH
1.891920989	-0.708310778	1.891920989
-0.128331529	-0.735553706	-0.128331529
0.416731075	0.035509287	0.416731075
0.284116413	-0.401303186	0.284116413
1.58824038	-0.920790303	1.58824038
0.168862084	0.56629557	0.168862084
0.338579403	-1.217566417	0.338579403
1.763337939	-0.85983444	1.763337939
1.872843443	-1.540231597	1.872843443
0.607154492	0.384153985	0.607154492
2.103694441	-1.210390957	2.103694441
1.55300188	-1.470796698	1.55300188
0.514967412	-0.494930062	0.514967412
1.251162844	-1.613431195	1.251162844
-0.092243826	-0.336990391	-0.092243826
0.651203761	-1.8027615	0.651203761
0.714939643	-1.241010242	0.714939643
-0.547028642	-1.212183156	-0.547028642
1.065659974	-0.338315773	1.065659974
0.284116413	-1.213976464	0.284116413
1.564840417	-1.566451808	1.564840417
-0.203225493	-0.138560951	-0.203225493
0.107728405	-0.006741433	0.107728405
0.107728405	0.633408097	0.107728405
0.735596069	-0.978092359	0.735596069
-0.499482833	0.468642178	-0.499482833
-0.699455064	0.765856396	-0.699455064
2.070413801	-1.462966277	2.070413801
-1.130278294	0.30775098	-1.130278294
-0.022576978	0.585629842	-0.022576978
0.011077634	0.505525436	0.011077634
-0.547028642	1.681117563	-0.547028642
-1.361129293	1.025465368	-1.361129293
-0.930306063	-0.431264189	-0.930306063
-0.323094825	0.764045138	-0.323094825
-1.060611445	0.065644751	-1.060611445
0.441900863	0.03317813	0.441900863
-0.699455064	1.392665914	-0.699455064
-1.203344843	1.307580168	-1.203344843
-0.40869339	0.803633401	-0.40869339
-1.446727857	-0.117602783	-1.446727857
-0.646903456	1.157331526	-0.646903456
-0.869172384	-0.440862623	-0.869172384
-1.280159997	0.323615659	-1.280159997
-0.810436731	1.322558456	-0.810436731
-1.130278294	0.673876089	-1.130278294
-0.128331529	0.084034946	-0.128331529

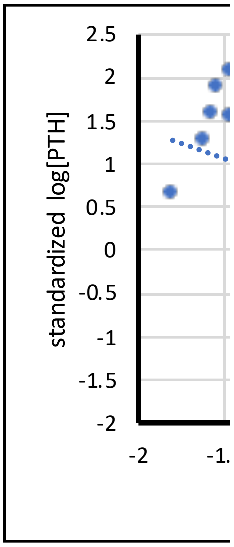

SUMMARY OU

Regression

Multiple R

R Square

Adjusted R Sq

Standard Erro

Observations

ANOVA

Regression

Residual

Total

Intercept

X Variable 1

-1.537517301	0.270391797	-1.537517301
-0.994041945	1.647929481	-0.994041945
-1.280159997	0.849732177	-1.280159997
-0.930306063	0.144131373	-0.930306063
0.561770195	-0.424427594	0.561770195
-0.930306063	2.086314975	-0.930306063
-0.869172384	1.324052032	-0.869172384
-0.810436731	1.850931374	-0.810436731

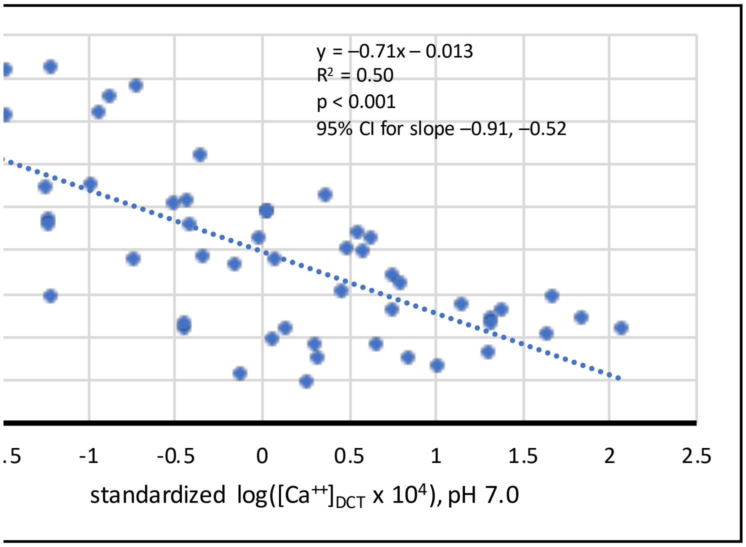

TPUT

Statistics
0.70958062
0.50350466
0.49413683
0.72316537
55

df	*SS*	*MS*	*F*	*Significance F*
1	28.1086139	28.1086139	53.7482331	1.3214E-09
53	27.7173118	0.52296815		
54	55.8259257			

Coefficients	*Standard Error*	*t Stat*	*P-value*	*Lower 95%*	*Upper 95%*	*Lower 95.0%*
-0.0131484	0.09751209	-0.1348388	0.89325014	-0.2087329	0.18243608	-0.2087329
-0.7143884	0.09744337	-7.3313186	1.3214E-09	-0.9098351	-0.5189418	-0.9098351

Upper 95.0%
0.18243608
-0.5189418

code	Ca+2	pth 1-84	Ca++ x 10^4	10/[Ca++] x 10^4	pth 1-84
CKD2	0.0001922	158	1.922	5.202913632	158
CKD4	0.0001904	41	1.904	5.25210084	41
CKD5	0.0002485	59	2.485	4.024144869	59
CKD6	0.0002137	54	2.137	4.679457183	54
CKD7	0.0001786	129	1.786	5.599104143	129
CKD11	0.0002985	50	2.985	3.350083752	50
CKD13	0.0001612	56	1.612	6.203473945	56
CKD14	0.0001824	145	1.824	5.48245614	145
CKD15	0.0001442	156	1.442	6.93481276	156
CKD18	0.0002803	67	2.803	3.567606136	67
CKD20	0.0001616	182	1.616	6.188118812	182
CKD21	0.0001477	126	1.477	6.770480704	126
CKD23	0.0002069	63	2.069	4.833252779	63
CKD24	0.0001406	103	1.406	7.112375533	103
CKD25	0.0002185	42	2.185	4.576659039	42
CKD26	0.0001317	69	1.317	7.593014427	69
CKD27	0.0001599	72	1.599	6.253908693	72
CKD31	0.0001615	31	1.615	6.191950464	31
CKD32	0.0002184	91	2.184	4.578754579	91
CKD33	0.0001614	54	1.614	6.195786865	54
CKD45	0.0001429	127	1.429	6.99790063	127
CKD46	0.000234	39	2.34	4.273504274	39
CKD49	0.0002449	48	2.449	4.083299306	48
CKD50	0.0003055	48	3.055	3.273322422	48
CKD51	0.0001751	73	1.751	5.711022273	73
CKD55	0.0002886	32	2.886	3.465003465	32
CKD59	0.0003198	28	3.198	3.126954346	28
CKD62	0.0001481	178	1.481	6.752194463	178
N2	0.000273	21	2.73	3.663003663	21
N3	0.0003005	44	3.005	3.327787022	44
N4	0.0002923	45	2.923	3.421142662	45
N6	0.0004387	31	4.387	2.279462047	31
N7	0.0003498	18	3.498	2.858776444	18
N8	0.0002115	24	2.115	4.728132388	24
N9	0.0003196	36	3.196	3.128911139	36
N10	0.0002511	22	2.511	3.982477101	22
N11	0.0002483	60	2.483	4.027386226	60
N13	0.0003971	28	3.971	2.518257366	28
N14	0.0003856	20	3.856	2.593360996	20
N15	0.000324	34	3.24	3.086419753	34
N16	0.0002357	17	2.357	4.242681375	17
N17	0.0003661	29	3.661	2.731494127	29
N18	0.0002108	25	2.108	4.743833017	25
N20	0.0002745	19	2.745	3.64298725	19
N21	0.0003876	26	3.876	2.57997936	26
N24	0.0003098	21	3.098	3.227888961	21
N25	0.0002527	41	2.527	3.957261575	41

N27	0.0002695	16	2.695	3.710575139	16
N29	0.0004337	23	4.337	2.305741296	23
N31	0.0003292	19	3.292	3.037667072	19
N32	0.000258	24	2.58	3.875968992	24
N33	0.000212	65	2.12	4.716981132	65
N35	0.0005046	24	5.046	1.981767737	24
N36	0.0003878	25	3.878	2.578648788	25
N38	0.0004652	26	4.652	2.14961307	26

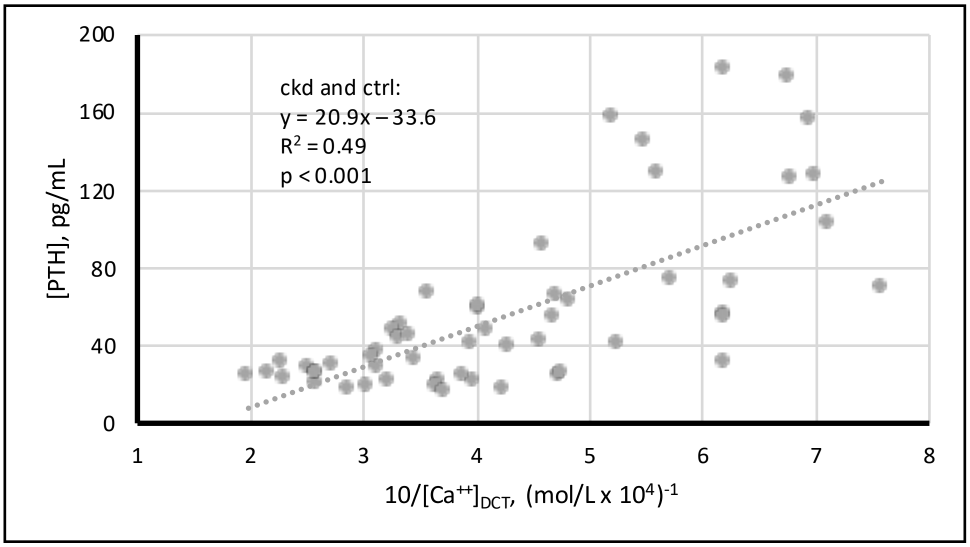

SUMMARY OUTPUT

Regression Statistics	
Multiple R	0.70283314
R Square	0.49397442
Adjusted R Sq	0.48442677
Standard Erro	31.6978269
Observations	55

ANOVA

	df	*SS*	*MS*	*F*	*Significance F*
Regression	1	51983.6589	51983.6589	51.7377889	2.2066E-09
Residual	53	53251.8684	1004.75223		
Total	54	105235.527			

	Coefficients	*Standard Error*	*t Stat*	*P-value*	*Lower 95%*
Intercept	-33.597331	13.2266456	-2.5401249	0.01404808	-60.126623
X Variable 1	20.8611635	2.90024439	7.1928985	2.2066E-09	15.0440099

Upper 95%	*Lower 95.0%*	*Upper 95.0%*
-7.06804	-60.126623	-7.06804
26.6783171	15.0440099	26.6783171

code	Ca+2	pth 1-84	Ca++ x 10^4	10/[Ca++] x 10^4	ckd
CKD2	0.0001922	158	1.922	5.202913632	158
CKD4	0.0001904	41	1.904	5.25210084	41
CKD5	0.0002485	59	2.485	4.024144869	59
CKD6	0.0002137	54	2.137	4.679457183	54
CKD7	0.0001786	129	1.786	5.599104143	129
CKD11	0.0002985	50	2.985	3.350083752	50
CKD13	0.0001612	56	1.612	6.203473945	56
CKD14	0.0001824	145	1.824	5.48245614	145
CKD15	0.0001442	156	1.442	6.93481276	156
CKD18	0.0002803	67	2.803	3.567606136	67
CKD20	0.0001616	182	1.616	6.188118812	182
CKD21	0.0001477	126	1.477	6.770480704	126
CKD23	0.0002069	63	2.069	4.833252779	63
CKD24	0.0001406	103	1.406	7.112375533	103
CKD25	0.0002185	42	2.185	4.576659039	42
CKD26	0.0001317	69	1.317	7.593014427	69
CKD27	0.0001599	72	1.599	6.253908693	72
CKD31	0.0001615	31	1.615	6.191950464	31
CKD32	0.0002184	91	2.184	4.578754579	91
CKD33	0.0001614	54	1.614	6.195786865	54
CKD45	0.0001429	127	1.429	6.99790063	127
CKD46	0.000234	39	2.34	4.273504274	39
CKD49	0.0002449	48	2.449	4.083299306	48
CKD50	0.0003055	48	3.055	3.273322422	48
CKD51	0.0001751	73	1.751	5.711022273	73
CKD55	0.0002886	32	2.886	3.465003465	32
CKD59	0.0003198	28	3.198	3.126954346	28
CKD62	0.0001481	178	1.481	6.752194463	178
N2	0.000273	21	2.73	3.663003663	
N3	0.0003005	44	3.005	3.327787022	
N4	0.0002923	45	2.923	3.421142662	
N6	0.0004387	31	4.387	2.279462047	
N7	0.0003498	18	3.498	2.858776444	
N8	0.0002115	24	2.115	4.728132388	
N9	0.0003196	36	3.196	3.128911139	
N10	0.0002511	22	2.511	3.982477101	
N11	0.0002483	60	2.483	4.027386226	
N13	0.0003971	28	3.971	2.518257366	
N14	0.0003856	20	3.856	2.593360996	
N15	0.000324	34	3.24	3.086419753	
N16	0.0002357	17	2.357	4.242681375	
N17	0.0003661	29	3.661	2.731494127	
N18	0.0002108	25	2.108	4.743833017	
N20	0.0002745	19	2.745	3.64298725	
N21	0.0003876	26	3.876	2.57997936	
N24	0.0003098	21	3.098	3.227888961	
N25	0.0002527	41	2.527	3.957261575	

N27	0.0002695	16	2.695	3.710575139
N29	0.0004337	23	4.337	2.305741296
N31	0.0003292	19	3.292	3.037667072
N32	0.000258	24	2.58	3.875968992
N33	0.000212	65	2.12	4.716981132
N35	0.0005046	24	5.046	1.981767737
N36	0.0003878	25	3.878	2.578648788
N38	0.0004652	26	4.652	2.14961307

ctrl	ckd and ctrl
	75.1408949
	76.1689076
	50.5046278
	64.2006551
	83.4212766
	36.4167504
	96.0526055
	80.9833333
	111.337587
	40.9629682
	95.7316832
	107.903047
	67.4149831
	115.048649
	62.0521739
	125.094002
	97.1066917
	95.8117647
	62.0959707
	95.8919455
	112.656123
	55.7162393
	51.7409555
	34.8124386
	85.7603655
	38.8185724
	31.7533458
	107.520864
21	42.9567766
44	35.9507488
45	37.9018816
31	14.0407568
18	26.1484277
24	65.2179669
36	31.7942428
22	49.6337714
60	50.5723721
28	19.0315789
20	20.6012448
34	30.9061728
17	55.0720407
29	23.4882273
25	65.5461101
19	42.5384335
26	20.3215686
21	33.8628793
41	49.1067669

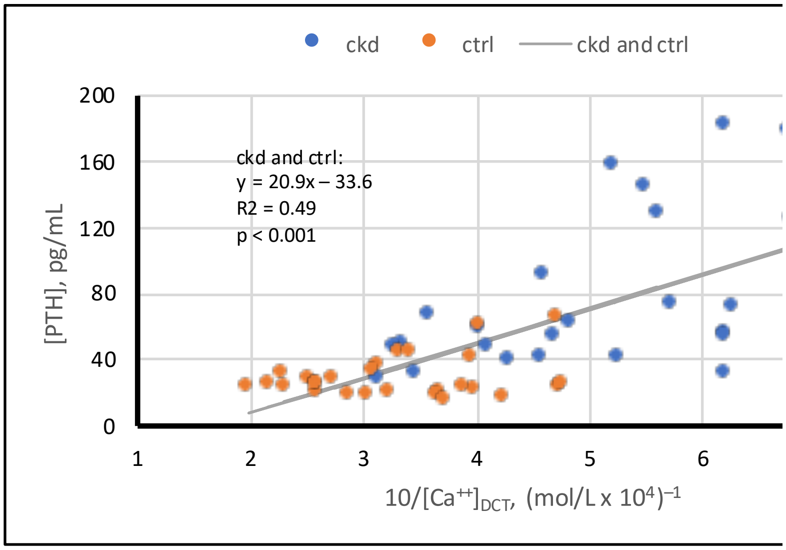

16	43.9510204
23	14.5899931
19	29.8872418
24	47.4077519
65	64.9849057
24	7.8189457
25	20.2937597
26	11.3269132

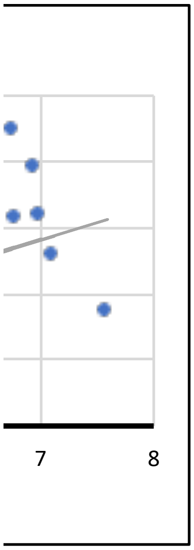
7
8

code	Ca++ x 10^4	ckd	ctrl	ckd and ctrl
CKD2	1.922	158		75.0408949
CKD4	1.904	41		76.0689076
CKD5	2.485	59		50.4046278
CKD6	2.137	54		64.1006551
CKD7	1.786	129		83.3212766
CKD11	2.985	50		36.3167504
CKD13	1.612	56		95.9526055
CKD14	1.824	145		80.8833333
CKD15	1.442	156		111.237587
CKD18	2.803	67		40.8629682
CKD20	1.616	182		95.6316832
CKD21	1.477	126		107.803047
CKD23	2.069	63		67.3149831
CKD24	1.406	103		114.948649
CKD25	2.185	42		61.9521739
CKD26	1.317	69		124.994002
CKD27	1.599	72		97.0066917
CKD31	1.615	31		95.7117647
CKD32	2.184	91		61.9959707
CKD33	1.614	54		95.7919455
CKD45	1.429	127		112.556123
CKD46	2.34	39		55.6162393
CKD49	2.449	48		51.6409555
CKD50	3.055	48		34.7124386
CKD51	1.751	73		85.6603655
CKD55	2.886	32		38.7185724
CKD59	3.198	28		31.6533458
CKD62	1.481	178		107.420864
N2	2.73		21	42.8567766
N3	3.005		44	35.8507488
N4	2.923		45	37.8018816
N6	4.387		31	13.9407568
N7	3.498		18	26.0484277
N8	2.115		24	65.1179669
N9	3.196		36	31.6942428
N10	2.511		22	49.5337714
N11	2.483		60	50.4723721
N13	3.971		28	18.9315789
N14	3.856		20	20.5012448
N15	3.24		34	30.8061728
N16	2.357		17	54.9720407
N17	3.661		29	23.3882273
N18	2.108		25	65.4461101
N20	2.745		19	42.4384335
N21	3.876		26	20.2215686
N24	3.098		21	33.7628793
N25	2.527		41	49.0067669

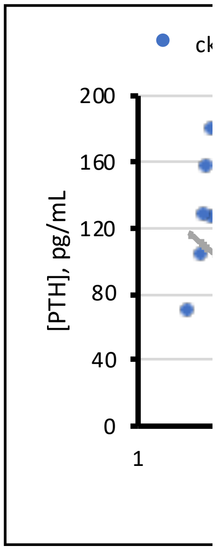

N27	2.695	16	43.8510204
N29	4.337	23	14.4899931
N31	3.292	19	29.7872418
N32	2.58	24	47.3077519
N33	2.12	65	64.8849057
N35	5.046	24	7.7189457
N36	3.878	25	20.1937597
N38	4.652	26	11.2269132

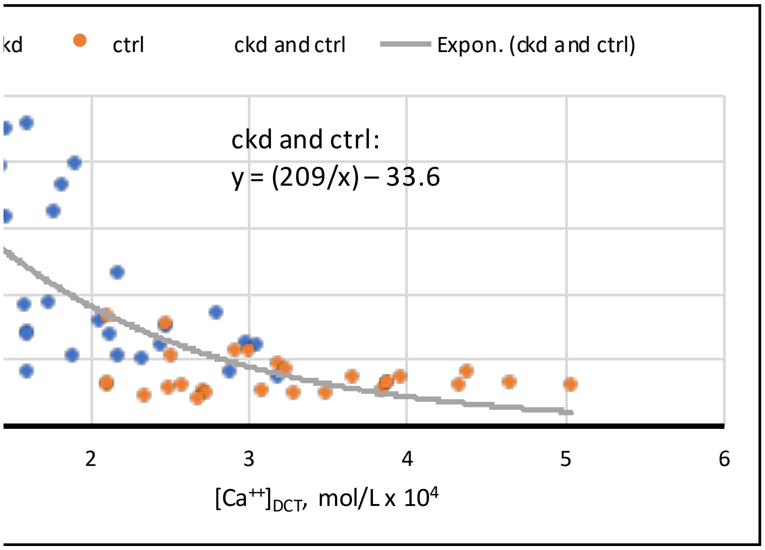

kd
ctrl
ckd and ctrl
Expon. (ckd and ctrl)
ckd and ctrl:
y = (209/x) – 33.6
2
3
4
5
6
[Ca++]DCT, mol/L x 10^4

code	Tot(P)	tot P x 10^3	ckd	ctrl
CKD2	0.00182	1.82	158	
CKD4	0.001852	1.852	41	
CKD5	0.001218	1.218	59	
CKD6	0.001549	1.549	54	
CKD7	0.002036	2.036	129	
CKD11	0.0008235	0.8235	50	
CKD13	0.002375	2.375	56	
CKD14	0.001973	1.973	145	
CKD15	0.002803	2.803	156	
CKD18	0.001003	1.003	67	
CKD20	0.002391	2.391	182	
CKD21	0.002718	2.718	126	
CKD23	0.001624	1.624	63	
CKD24	0.002935	2.935	103	
CKD25	0.001507	1.507	42	
CKD26	0.003251	3.251	69	
CKD27	0.002414	2.414	72	
CKD31	0.002402	2.402	31	
CKD32	0.001489	1.489	91	
CKD33	0.002371	2.371	54	
CKD45	0.002874	2.874	127	
CKD46	0.001331	1.331	39	
CKD49	0.001242	1.242	48	
CKD50	0.0007575	0.7575	48	
CKD51	0.002103	2.103	73	
CKD55	0.0007141	0.7141	32	
CKD59	0.0006192	0.6192	28	
CKD62	0.002702	2.702	178	
N2	0.001258	1.258		21
N3	0.001075	1.075		44
N4	0.001127	1.127		45
N6	0.0005899	0.5899		31
N7	0.0008665	0.8665		18
N8	0.001751	1.751		24
N9	0.0009826	0.9826		36
N10	0.001394	1.394		22
N11	0.001392	1.392		60
N13	0.0006849	0.6849		28
N14	0.0007527	0.7527		20
N15	0.0009714	0.9714		34
N16	0.001539	1.539		17
N17	0.0008413	0.8413		29
N18	0.001776	1.776		25
N20	0.001206	1.206		19
N21	0.0007058	0.7058		26
N24	0.001062	1.062		21
N25	0.00135	1.35		41

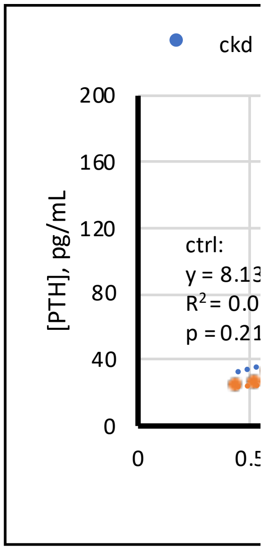

SUMMARY OU

Regression
Multiple R
R Square
Adjusted R Sq
Standard Erro
Observations

ANOVA

Regression
Residual
Total

Intercept
X Variable 1

SUMMARY OU

Regression
Multiple R
R Square
Adjusted R Sq
Standard Erro
Observations

ANOVA

Regression

N27	0.001305	1.305	16	Residual
N29	0.0006108	0.6108	23	Total
N31	0.000974	0.974	19	
N32	0.001335	1.335	24	
N33	0.001729	1.729	65	Intercept
N35	0.0004466	0.4466	24	X Variable 1
N36	0.0007627	0.7627	25	
N38	0.0005282	0.5282	26	

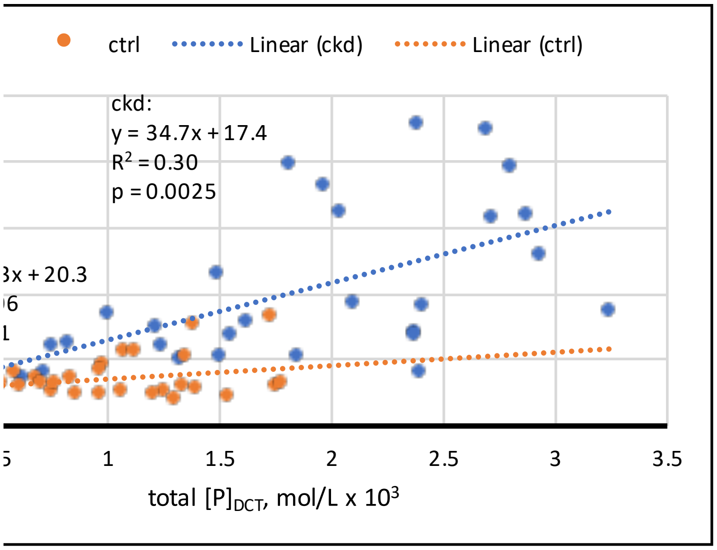

TPUT ckd

Statistics
0.54792622
0.30022314
0.27330865
40.6180126
28

df	SS	MS	F	Significance F
1	18403.282	18403.282	11.1547012	0.00254162
26	42895.3966	1649.82295		
27	61298.6786			

Coefficients	Standard Error	t Stat	P-value	Lower 95%	Upper 95%	Lower 95.0%
17.3899266	21.0611084	0.82568905	0.41649288	-25.901802	60.6816549	-25.901802
34.6725079	10.3814086	3.33986544	0.00254162	13.3332169	56.011799	13.3332169

TPUT ctrl

Statistics
0.25035381
0.06267703
0.02518411
12.2677046
27

df	SS	MS	F	Significance F
1	251.585607	251.585607	1.6717032	0.20785198

25	3762.41439	150.496576
26	4014	

Coefficients	*Standard Error*	*t Stat*	*P-value*	*Lower 95%*	*Upper 95%*	*Lower 95.0%*
20.261812	7.15887404	2.83030709	0.00904204	5.51783489	35.0057891	5.51783489
8.13095617	6.28871675	1.29294362	0.20785198	-4.8208984	21.0828108	-4.8208984

Upper 95.0%
60.6816549
56.011799

Upper 95.0%
35.0057891
21.0828108

code	CaHPO4	CaHPO4 x 10^5	ckd	ctrl
CKD2	1.98E-05	1.979	158	
CKD4	1.99E-05	1.989	41	
CKD5	1.74E-05	1.74	59	
CKD6	1.88E-05	1.876	54	
CKD7	2.05E-05	2.053	129	
CKD11	1.46E-05	1.457	50	
CKD13	2.16E-05	2.162	56	
CKD14	2.03E-05	2.032	145	
CKD15	2.29E-05	2.286	156	
CKD18	1.64E-05	1.638	67	
CKD20	2.16E-05	2.16	182	
CKD21	2.26E-05	2.259	126	
CKD23	1.91E-05	1.907	63	
CKD24	2.32E-05	2.316	103	
CKD25	1.86E-05	1.856	42	
CKD26	2.39E-05	2.393	69	
CKD27	2.17E-05	2.171	72	
CKD31	2.16E-05	2.16	31	
CKD32	1.86E-05	1.856	91	
CKD33	2.16E-05	2.161	54	
CKD45	2.30E-05	2.297	127	
CKD46	1.79E-05	1.793	39	
CKD49	1.75E-05	1.753	48	
CKD50	1.37E-05	1.372	48	
CKD51	2.07E-05	2.074	73	
CKD55	1.22E-05	1.224	32	
CKD59	1.18E-05	1.175	28	
CKD62	2.26E-05	2.256	178	
N2	1.66E-05	1.66		21
N3	1.58E-05	1.582		44
N4	1.60E-05	1.604		45
N6	1.31E-05	1.309		31
N7	1.47E-05	1.466		18
N8	1.89E-05	1.887		24
N9	1.53E-05	1.534		36
N10	1.73E-05	1.731		22
N11	1.74E-05	1.741		60
N13	1.38E-05	1.376		28
N14	1.40E-05	1.396		20
N15	1.52E-05	1.523		34
N16	1.79E-05	1.787		17
N17	1.43E-05	1.433		29
N18	1.89E-05	1.89		25
N20	1.66E-05	1.655		19
N21	1.39E-05	1.393		26
N24	1.56E-05	1.558		21
N25	1.73E-05	1.726		41

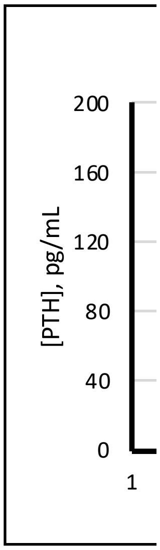

SUMMARY OU

Regression
Multiple R
R Square
Adjusted R Sq
Standard Erro
Observations

ANOVA

Regression
Residual
Total

Intercept
X Variable 1

SUMMARY OU

Regression
Multiple R
R Square
Adjusted R Sq
Standard Erro

N27	1.67E-05	1.671	16	Observations
N29	1.32E-05	1.317	23	
N31	1.51E-05	1.511	19	ANOVA
N32	1.71E-05	1.708	24	
N33	1.88E-05	1.884	65	Regression
N35	1.22E-05	1.221	24	Residual
N36	1.39E-05	1.392	25	Total
N38	1.27E-05	1.271	26	
				Intercept
				X Variable 1

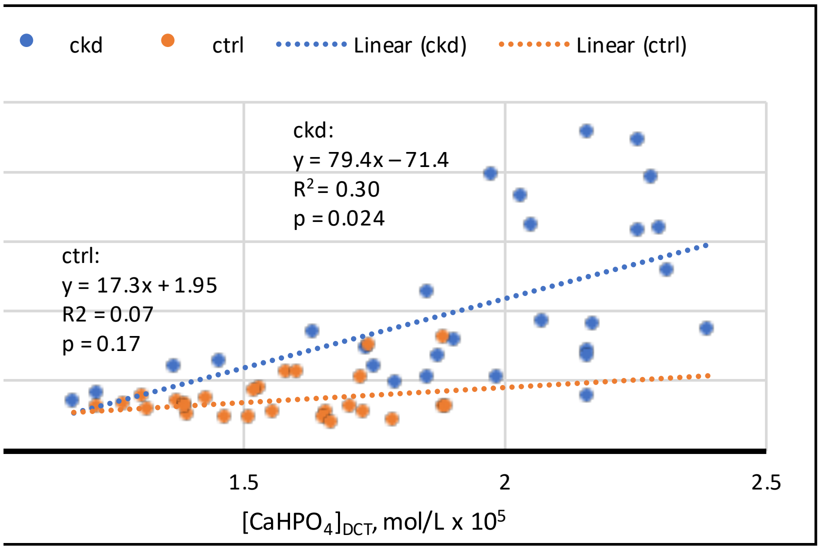

TPUT ckd

Statistics
0.55064785
0.30321305
0.27641355
40.5311463
28

df	*SS*	*MS*	*F*	*Significance F*
1	18586.5593	18586.5593	11.3141317	0.00239422
26	42712.1193	1642.77382		
27	61298.6786			

Coefficients	*Standard Error*	*t Stat*	*P-value*	*Lower 95%*	*Upper 95%*	*Lower 95.0%*
-71.375996	46.4987678	-1.5350083	0.13686231	-166.95558	24.2035902	-166.95558
79.4103849	23.6084072	3.36364857	0.00239422	30.882609	127.938161	30.882609

TPUT ctrl

Statistics
0.27076303
0.07331262
0.03624513
12.1979066

27

df	*SS*	*MS*	*F*	*Significance F*
1	294.27686	294.27686	1.97781427	0.17192987
25	3719.72314	148.788926		
26	4014			

Coefficients	*Standard Error*	*t Stat*	*P-value*	*Lower 95%*	*Upper 95%*	*Lower 95.0%*
1.95326688	19.3746344	0.10081568	0.92050096	-37.94954	41.8560734	-37.94954
17.2941267	12.29719	1.40634785	0.17192987	-8.0324102	42.6206636	-8.0324102

Upper 95.0%
24.2035902
127.938161

Upper 95.0%
41.8560734
42.6206636

code	CaHPO4	CaHPO4 x 10^5	pth
CKD2	1.98E-05	1.979	158
CKD4	1.99E-05	1.989	41
CKD5	1.74E-05	1.74	59
CKD6	1.88E-05	1.876	54
CKD7	2.05E-05	2.053	129
CKD11	1.46E-05	1.457	50
CKD13	2.16E-05	2.162	56
CKD14	2.03E-05	2.032	145
CKD15	2.29E-05	2.286	156
CKD18	1.64E-05	1.638	67
CKD20	2.16E-05	2.16	182
CKD21	2.26E-05	2.259	126
CKD23	1.91E-05	1.907	63
CKD24	2.32E-05	2.316	103
CKD25	1.86E-05	1.856	42
CKD26	2.39E-05	2.393	69
CKD27	2.17E-05	2.171	72
CKD31	2.16E-05	2.16	31
CKD32	1.86E-05	1.856	91
CKD33	2.16E-05	2.161	54
CKD45	2.30E-05	2.297	127
CKD46	1.79E-05	1.793	39
CKD49	1.75E-05	1.753	48
CKD50	1.37E-05	1.372	48
CKD51	2.07E-05	2.074	73
CKD55	1.22E-05	1.224	32
CKD59	1.18E-05	1.175	28
CKD62	2.26E-05	2.256	178
N2	1.66E-05	1.66	21
N3	1.58E-05	1.582	44
N4	1.60E-05	1.604	45
N6	1.31E-05	1.309	31
N7	1.47E-05	1.466	18
N8	1.89E-05	1.887	24
N9	1.53E-05	1.534	36
N10	1.73E-05	1.731	22
N11	1.74E-05	1.741	60
N13	1.38E-05	1.376	28
N14	1.40E-05	1.396	20
N15	1.52E-05	1.523	34
N16	1.79E-05	1.787	17
N17	1.43E-05	1.433	29
N18	1.89E-05	1.89	25
N20	1.66E-05	1.655	19
N21	1.39E-05	1.393	26
N24	1.56E-05	1.558	21
N25	1.73E-05	1.726	41

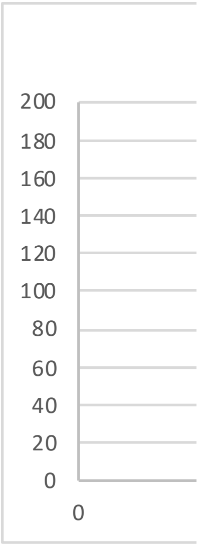

N27	1.67E-05	1.671	16
N29	1.32E-05	1.317	23
N31	1.51E-05	1.511	19
N32	1.71E-05	1.708	24
N33	1.88E-05	1.884	65
N35	1.22E-05	1.221	24
N36	1.39E-05	1.392	25
N38	1.27E-05	1.271	26

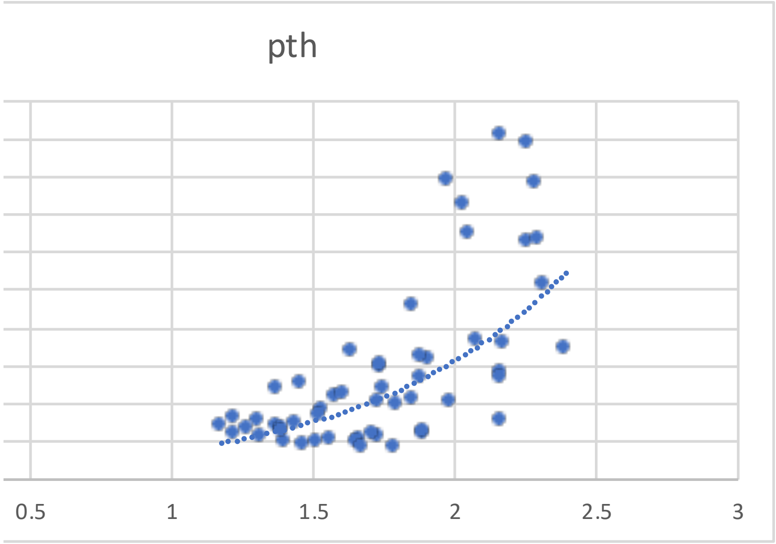
pth
0.5
1
1.5
2
2.5
3

pth	CaHPO4 x 10^5
158	1.979
41	1.989
59	1.74
54	1.876
129	2.053
50	1.457
56	2.162
145	2.032
156	2.286
67	1.638
182	2.16
126	2.259
63	1.907
103	2.316
42	1.856
69	2.393
72	2.171
31	2.16
91	1.856
54	2.161
127	2.297
39	1.793
48	1.753
48	1.372
73	2.074
32	1.224
28	1.175
178	2.256
21	1.66
44	1.582
45	1.604
31	1.309
18	1.466
24	1.887
36	1.534
22	1.731
60	1.741
28	1.376
20	1.396
34	1.523
17	1.787
29	1.433
25	1.89
19	1.655
26	1.393
21	1.558
41	1.726

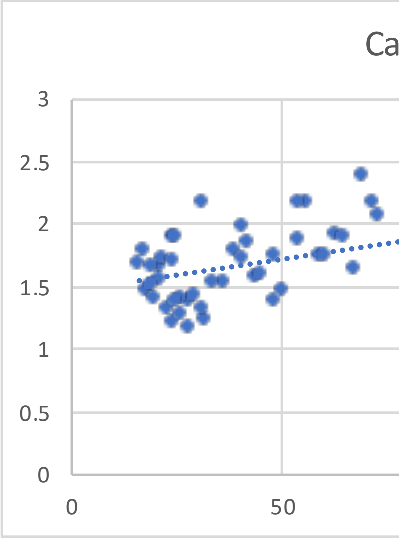

16	1.671
23	1.317
19	1.511
24	1.708
65	1.884
24	1.221
25	1.392
26	1.271

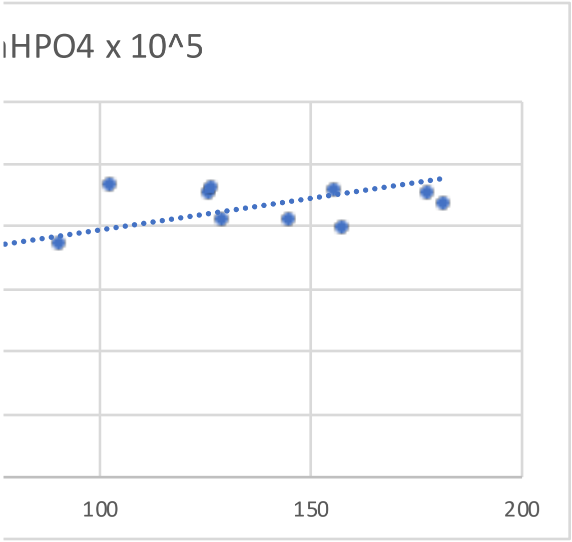

HPO4 x 10^5
100
150
200

code	CaHPO4	CaHPO4 x 10^5	pth		CaHPO4 x 10^5	10/cahpo4
CKD2	0.00001979	1.979	158		1.979	5.0530571
CKD4	0.00001989	1.989	41		1.989	5.02765209
CKD5	0.0000174	1.74	59		1.74	5.74712644
CKD6	0.00001876	1.876	54		1.876	5.33049041
CKD7	0.00002053	2.053	129		2.053	4.8709206
CKD11	0.00001457	1.457	50		1.457	6.86341798
CKD13	0.00002162	2.162	56		2.162	4.6253469
CKD14	0.00002032	2.032	145		2.032	4.92125984
CKD15	0.00002286	2.286	156		2.286	4.37445319
CKD18	0.00001638	1.638	67		1.638	6.10500611
CKD20	0.0000216	2.16	182		2.16	4.62962963
CKD21	0.00002259	2.259	126		2.259	4.42673749
CKD23	0.00001907	1.907	63		1.907	5.24383849
CKD24	0.00002316	2.316	103		2.316	4.31778929
CKD25	0.00001856	1.856	42		1.856	5.38793103
CKD26	0.00002393	2.393	69		2.393	4.17885499
CKD27	0.00002171	2.171	72		2.171	4.60617227
CKD31	0.0000216	2.16	31		2.16	4.62962963
CKD32	0.00001856	1.856	91		1.856	5.38793103
CKD33	0.00002161	2.161	54		2.161	4.62748727
CKD45	0.00002297	2.297	127		2.297	4.35350457
CKD46	0.00001793	1.793	39		1.793	5.57724484
CKD49	0.00001753	1.753	48		1.753	5.70450656
CKD50	0.00001372	1.372	48		1.372	7.28862974
CKD51	0.00002074	2.074	73		2.074	4.82160077
CKD55	0.00001224	1.224	32		1.224	8.16993464
CKD59	0.00001175	1.175	28		1.175	8.5106383
CKD62	0.00002256	2.256	178		2.256	4.43262411
N2	0.0000166	1.66	21		1.66	6.02409639
N3	0.00001582	1.582	44		1.582	6.32111252
N4	0.00001604	1.604	45		1.604	6.23441397
N6	0.00001309	1.309	31		1.309	7.6394194
N7	0.00001466	1.466	18		1.466	6.8212824
N8	0.00001887	1.887	24		1.887	5.29941706
N9	0.00001534	1.534	36		1.534	6.51890482
N10	0.00001731	1.731	22		1.731	5.77700751
N11	0.00001741	1.741	60		1.741	5.74382539
N13	0.00001376	1.376	28		1.376	7.26744186
N14	0.00001396	1.396	20		1.396	7.16332378
N15	0.00001523	1.523	34		1.523	6.56598818
N16	0.00001787	1.787	17		1.787	5.5959709
N17	0.00001433	1.433	29		1.433	6.97836706
N18	0.0000189	1.89	25		1.89	5.29100529
N20	0.00001655	1.655	19		1.655	6.04229607
N21	0.00001393	1.393	26		1.393	7.1787509
N24	0.00001558	1.558	21		1.558	6.41848524
N25	0.00001726	1.726	41		1.726	5.79374276

N27	0.00001671	1.671	16		1.671	5.98444045
N29	0.00001317	1.317	23		1.317	7.59301443
N31	0.00001511	1.511	19		1.511	6.61813369
N32	0.00001708	1.708	24		1.708	5.85480094
N33	0.00001884	1.884	65		1.884	5.30785563
N35	0.00001221	1.221	24		1.221	8.19000819
N36	0.00001392	1.392	25		1.392	7.18390805
N38	0.00001271	1.271	26		1.271	7.86782061

pth
158
41
59
54
129
50
56
145
156
67
182
126
63
103
42
69
72
31
91
54
127
39
48
48
73
32
28
178
21
44
45
31
18
24
36
22
60
28

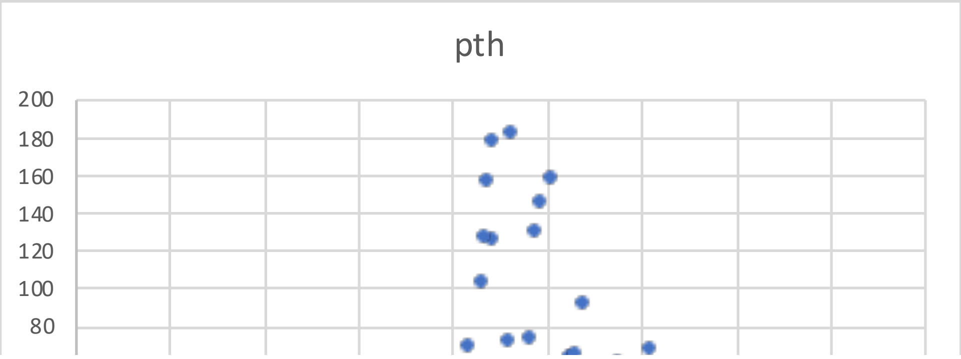

Supplement: S5 File — (PDF) [file pone.0272380.s014.pdf]
