# Supplementary material for: Chemical evidence for the tradeoff-in-the-nephron hypothesis to explain secondary hyperparathyroidism
Source: PLoS One. 2022 Aug 1;17(8):e0272380. doi: 10.1371/journal.pone.0272380 (PMC9342777; doi:10.1371/journal.pone.0272380)
Supplement: S6 File — (PDF) [file pone.0272380.s015.pdf]

code	eGFR	PTH		logeGFR	logPTH
CKD2	21	158		1.32221929	2.19865709
CKD4	23	41		1.36172784	1.61278386
CKD5	34	59		1.53147892	1.77085201
CKD6	41	54		1.61278386	1.73239376
CKD7	19	129		1.2787536	2.11058971
CKD11	33	50		1.51851394	1.69897
CKD13	22	56		1.34242268	1.74818803
CKD14	14	145		1.14612804	2.161368
CKD15	22	156		1.34242268	2.1931246
CKD18	36	67		1.5563025	1.8260748
CKD20	20	182		1.30103	2.26007139
CKD21	28	126		1.44715803	2.10037055
CKD23	35	63		1.54406804	1.79934055
CKD24	20	103		1.30103	2.01283722
CKD25	44	42		1.64345268	1.62324929
CKD26	18	69		1.25527251	1.83884909
CKD27	28	72		1.44715803	1.8573325
CKD31	23	31		1.36172784	1.49136169
CKD32	29	91		1.462398	1.95904139
CKD33	28	54		1.44715803	1.73239376
CKD45	29	127		1.462398	2.10380372
CKD46	42	39		1.62324929	1.59106461
CKD49	42	48		1.62324929	1.68124124
CKD50	49	48		1.69019608	1.68124124
CKD51	34	73		1.53147892	1.86332286
CKD55	28	32		1.44715803	1.50514998
CKD59	47	28		1.67209786	1.44715803
CKD62	27	178		1.43136376	2.25042
N2	89	21		1.94939001	1.32221929
N3	101	44		2.00432137	1.64345268
N4	93	45		1.96848295	1.65321251
N6	103	31		2.01283722	1.49136169
N7	94	18		1.97312785	1.25527251
N8	79	24		1.89762709	1.38021124
N9	77	36		1.88649073	1.5563025
N10	73	22		1.86332286	1.34242268
N11	108	60		2.03342376	1.77815125
N13	87	28		1.93951925	1.44715803
N14	93	20		1.96848295	1.30103
N15	96	34		1.98227123	1.53147892
N16	96	17		1.98227123	1.23044892
N17	73	29		1.86332286	1.462398
N18	90	25		1.95424251	1.39794001
N20	75	19		1.87506126	1.2787536
N21	75	26		1.87506126	1.41497335
N24	75	21		1.87506126	1.32221929
N25	89	41		1.94939001	1.61278386

N27	74	16	1.86923172	1.20411998
N29	85	23	1.92941893	1.36172784
N31	72	19	1.8573325	1.2787536
N32	89	24	1.94939001	1.38021124
N33	93	65	1.96848295	1.81291336
N35	78	24	1.8920946	1.38021124
N36	84	25	1.92427929	1.39794001
N38	87	26	1.93951925	1.41497335

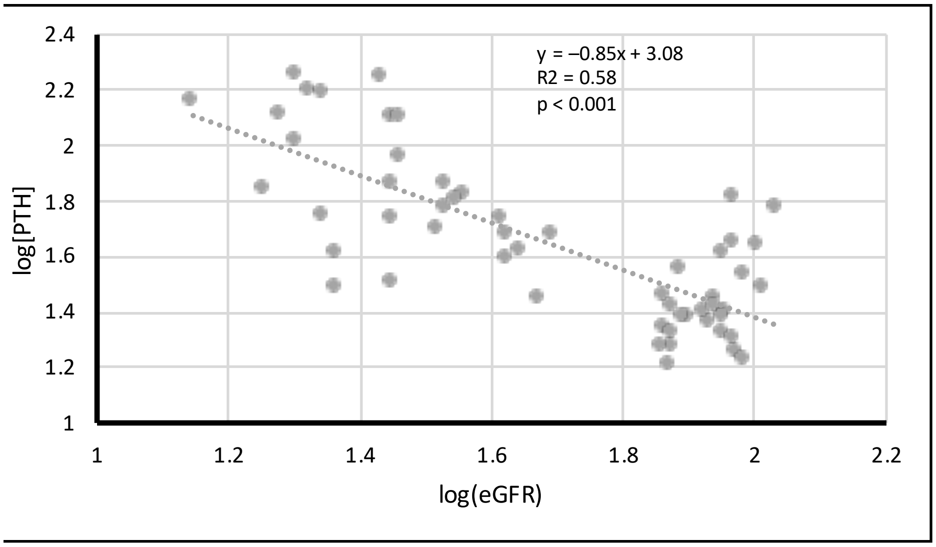

Column1		log eGFR
Mean	1.68887008	
Standard Erro	0.03553327	
Median	1.69019608	
Mode	1.44715803	
Standard Devi	0.2635218	
Sample Variar	0.06944374	
Kurtosis	-1.4064901	
Skewness	-0.319388	
Range	0.88729572	
Minimum	1.14612804	
Maximum	2.03342376	
Sum	92.8878546	
Count	55	

Column1		logPTH
Mean	1.64552531	
Standard Erro	0.03975921	
Median	1.61278386	
Mode	1.38021124	
Standard Devi	0.29486219	
Sample Variar	0.08694371	
Kurtosis	-0.6740973	
Skewness	0.54246903	
Range	1.05595141	
Minimum	1.20411998	
Maximum	2.26007139	
Sum	90.5038919	
Count	55	

SUMMARY OUTPUT

Regression Statistics	
Multiple R	0.76168949
R Square	0.58017088
Adjusted R Sq	0.57224958
Standard Erro	0.19284763
Observations	55

ANOVA

	df	*SS*	*MS*	*F*	*Significance F*
Regression	1	2.72387928	2.72387928	73.2418388	1.4516E-11
Residual	53	1.97108107	0.03719021		
Total	54	4.69496035			

	Coefficients	*Standard Error*	*t Stat*	*P-value*	*Lower 95%*	*Upper 95%*
Intercept	3.08490953	0.17018714	18.126572	2.2412E-24	2.74355735	3.42626171
X Variable 1	-0.8522765	0.09958659	-8.5581446	1.4516E-11	-1.0520219	-0.6525311

logeGFR	mean	SD	standardized logeGFR
1.32221929	1.689	0.264	-1.3893209
1.36172784	1.689	0.264	-1.2396673
1.53147892	1.689	0.264	-0.5966708
1.61278386	1.689	0.264	-0.2886975
1.2787536	1.689	0.264	-1.5539636
1.51851394	1.689	0.264	-0.6457805
1.34242268	1.689	0.264	-1.3127929
1.14612804	1.689	0.264	-2.0563332
1.34242268	1.689	0.264	-1.3127929
1.5563025	1.689	0.264	-0.502642
1.30103	1.689	0.264	-1.4695833
1.44715803	1.689	0.264	-0.9160681
1.54406804	1.689	0.264	-0.5489847
1.30103	1.689	0.264	-1.4695833
1.64345268	1.689	0.264	-0.1725277
1.25527251	1.689	0.264	-1.6429072
1.44715803	1.689	0.264	-0.9160681
1.36172784	1.689	0.264	-1.2396673
1.462398	1.689	0.264	-0.8583409
1.44715803	1.689	0.264	-0.9160681
1.462398	1.689	0.264	-0.8583409
1.62324929	1.689	0.264	-0.2490557
1.62324929	1.689	0.264	-0.2490557
1.69019608	1.689	0.264	0.00453061
1.53147892	1.689	0.264	-0.5966708
1.44715803	1.689	0.264	-0.9160681
1.67209786	1.689	0.264	-0.0640233
1.43136376	1.689	0.264	-0.9758948
1.94939001	1.689	0.264	0.98632578
2.00432137	1.689	0.264	1.19439914
1.96848295	1.689	0.264	1.05864753
2.01283722	1.689	0.264	1.22665615
1.97312785	1.689	0.264	1.07624187
1.89762709	1.689	0.264	0.79025413
1.88649073	1.689	0.264	0.74807093
1.86332286	1.689	0.264	0.66031386
2.03342376	1.689	0.264	1.30463544
1.93951925	1.689	0.264	0.94893656
1.96848295	1.689	0.264	1.05864753
1.98227123	1.689	0.264	1.11087588
1.98227123	1.689	0.264	1.11087588
1.86332286	1.689	0.264	0.66031386
1.95424251	1.689	0.264	1.00470648
1.87506126	1.689	0.264	0.70477751
1.87506126	1.689	0.264	0.70477751
1.87506126	1.689	0.264	0.70477751
1.94939001	1.689	0.264	0.98632578

Lower 95.0%	*Upper 95.0%*	1.86923172	1.689	0.264	0.68269591
2.74355735	3.42626171	1.92941893	1.689	0.264	0.91067775
-1.0520219	-0.6525311	1.8573325	1.689	0.264	0.63762309
		1.94939001	1.689	0.264	0.98632578
		1.96848295	1.689	0.264	1.05864753
		1.8920946	1.689	0.264	0.76929774
		1.92427929	1.689	0.264	0.89120942
		1.93951925	1.689	0.264	0.94893656

logPTH	mean	SD	standardized logPTH
2.19865709	1.646	0.295	1.873413854
1.61278386	1.646	0.295	-0.112597096
1.77085201	1.646	0.295	0.423227158
1.73239376	1.646	0.295	0.292860203
2.11058971	1.646	0.295	1.574880374
1.69897	1.646	0.295	0.179559337
1.74818803	1.646	0.295	0.346400092
2.161368	1.646	0.295	1.747010177
2.1931246	1.646	0.295	1.854659655
1.8260748	1.646	0.295	0.61042306
2.26007139	1.646	0.295	2.081597925
2.10037055	1.646	0.295	1.540239136
1.79934055	1.646	0.295	0.519798473
2.01283722	1.646	0.295	1.243516016
1.62324929	1.646	0.295	-0.077121049
1.83884909	1.646	0.295	0.653725731
1.8573325	1.646	0.295	0.716381344
1.49136169	1.646	0.295	-0.524197648
1.95904139	1.646	0.295	1.061157262
1.73239376	1.646	0.295	0.292860203
2.10380372	1.646	0.295	1.55187702
1.59106461	1.646	0.295	-0.186221671
1.68124124	1.646	0.295	0.119461822
1.68124124	1.646	0.295	0.119461822
1.86332286	1.646	0.295	0.736687661
1.50514998	1.646	0.295	-0.477457701
1.44715803	1.646	0.295	-0.674040572
2.25042	1.646	0.295	2.048881364
1.32221929	1.646	0.295	-1.097561713
1.64345268	1.646	0.295	-0.008634995
1.65321251	1.646	0.295	0.024449199
1.49136169	1.646	0.295	-0.524197648
1.25527251	1.646	0.295	-1.324499983
1.38021124	1.646	0.295	-0.900978842
1.5563025	1.646	0.295	-0.304059319
1.34242268	1.646	0.295	-1.029075658
1.77815125	1.646	0.295	0.44797034
1.44715803	1.646	0.295	-0.674040572
1.30103	1.646	0.295	-1.169389845
1.53147892	1.646	0.295	-0.388207061
1.23044892	1.646	0.295	-1.408647724
1.462398	1.646	0.295	-0.622379668
1.39794001	1.646	0.295	-0.840881327
1.2787536	1.646	0.295	-1.244903048
1.41497335	1.646	0.295	-0.783141193
1.32221929	1.646	0.295	-1.097561713
1.61278386	1.646	0.295	-0.112597096

1.20411998	1.646	0.295	-1.497898364
1.36172784	1.646	0.295	-0.963634454
1.2787536	1.646	0.295	-1.244903048
1.38021124	1.646	0.295	-0.900978842
1.81291336	1.646	0.295	0.565807989
1.38021124	1.646	0.295	-0.900978842
1.39794001	1.646	0.295	-0.840881327
1.41497335	1.646	0.295	-0.783141193

standardized logeGFR	standardized logPTH
-1.389320853	1.873413854
-1.239667288	-0.112597096
-0.596670769	0.423227158
-0.288697512	0.292860203
-1.553963633	1.574880374
-0.645780531	0.179559337
-1.312792876	0.346400092
-2.056333198	1.747010177
-1.312792876	1.854659655
-0.502642043	0.61042306
-1.46958335	2.081597925
-0.916068063	1.540239136
-0.54898468	0.519798473
-1.46958335	1.243516016
-0.172527741	-0.077121049
-1.642907178	0.653725731
-0.916068063	0.716381344
-1.239667288	-0.524197648
-0.858340917	1.061157262
-0.916068063	0.292860203
-0.858340917	1.55187702
-0.249055718	-0.186221671
-0.249055718	0.119461822
0.004530606	0.119461822
-0.596670769	0.736687661
-0.916068063	-0.477457701
-0.064023265	-0.674040572
-0.975894833	2.048881364
0.986325783	-1.097561713
1.194399143	-0.008634995
1.058647532	0.024449199
1.226656154	-0.524197648
1.07624187	-1.324499983
0.790254134	-0.900978842
0.748070929	-0.304059319
0.660313864	-1.029075658
1.304635437	0.44797034
0.948936563	-0.674040572
1.058647532	-1.169389845
1.110875883	-0.388207061
1.110875883	-1.408647724
0.660313864	-0.622379668
1.004706475	-0.840881327
0.704777513	-1.244903048
0.704777513	-0.783141193
0.704777513	-1.097561713
0.986325783	-0.112597096

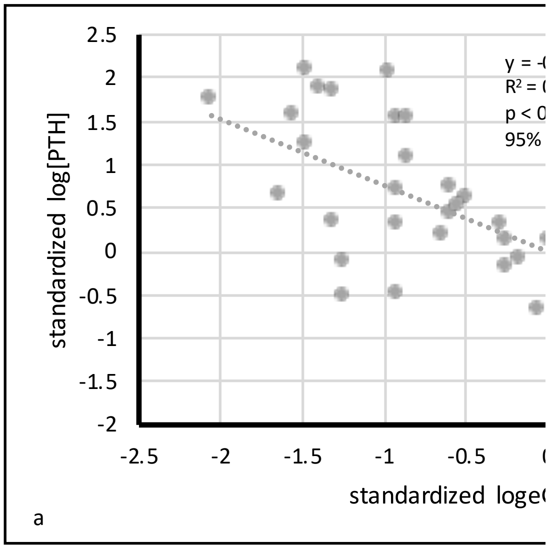

SUMMARY OUTPUT

Regression Statistics	
Multiple R	0.76168949
R Square	0.58017088
Adjusted R Sq	0.57224958
Standard Erro	0.65372078
Observations	55

ANOVA

	df	*SS*
Regression	1	31.299963
Residual	53	22.6495957
Total	54	53.9495588

	Coefficients	*Standard Error*
Intercept	-0.0019845	0.0881477
X Variable 1	-0.7627152	0.08912156

0.682695908	-1.497898364
0.910677749	-0.963634454
0.637623093	-1.244903048
0.986325783	-0.900978842
1.058647532	0.565807989
0.769297737	-0.900978842
0.891209417	-0.840881327
0.948936563	-0.783141193

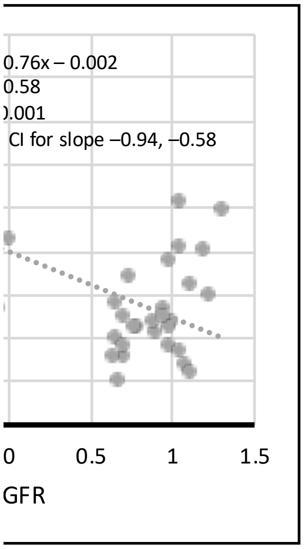

MS	F	Significance F
31.299963	73.2418388	1.4516E-11
0.42735086		

t Stat	P-value	Lower 95%	Upper 95%	Lower 95.0%	Upper 95.0%
-0.0225129	0.98212334	-0.1787864	0.17481744	-0.1787864	0.17481744
-8.5581446	1.4516E-11	-0.9414704	-0.58396	-0.9414704	-0.58396

code	eGFR	PTH	logeGFR	ckd	ctrl
CKD2	21	158	1.32221929	2.19865709	
CKD4	23	41	1.36172784	1.61278386	
CKD5	34	59	1.53147892	1.77085201	
CKD6	41	54	1.61278386	1.73239376	
CKD7	19	129	1.2787536	2.11058971	
CKD11	33	50	1.51851394	1.69897	
CKD13	22	56	1.34242268	1.74818803	
CKD14	14	145	1.14612804	2.161368	
CKD15	22	156	1.34242268	2.1931246	
CKD18	36	67	1.5563025	1.8260748	
CKD20	20	182	1.30103	2.26007139	
CKD21	28	126	1.44715803	2.10037055	
CKD23	35	63	1.54406804	1.79934055	
CKD24	20	103	1.30103	2.01283722	
CKD25	44	42	1.64345268	1.62324929	
CKD26	18	69	1.25527251	1.83884909	
CKD27	28	72	1.44715803	1.8573325	
CKD31	23	31	1.36172784	1.49136169	
CKD32	29	91	1.462398	1.95904139	
CKD33	28	54	1.44715803	1.73239376	
CKD45	29	127	1.462398	2.10380372	
CKD46	42	39	1.62324929	1.59106461	
CKD49	42	48	1.62324929	1.68124124	
CKD50	49	48	1.69019608	1.68124124	
CKD51	34	73	1.53147892	1.86332286	
CKD55	28	32	1.44715803	1.50514998	
CKD59	47	28	1.67209786	1.44715803	
CKD62	27	178	1.43136376	2.25042	
N2	89	21	1.94939001		1.32221929
N3	101	44	2.00432137		1.64345268
N4	93	45	1.96848295		1.65321251
N6	103	31	2.01283722		1.49136169
N7	94	18	1.97312785		1.25527251
N8	79	24	1.89762709		1.38021124
N9	77	36	1.88649073		1.5563025
N10	73	22	1.86332286		1.34242268
N11	108	60	2.03342376		1.77815125
N13	87	28	1.93951925		1.44715803
N14	93	20	1.96848295		1.30103
N15	96	34	1.98227123		1.53147892
N16	96	17	1.98227123		1.23044892
N17	73	29	1.86332286		1.462398
N18	90	25	1.95424251		1.39794001
N20	75	19	1.87506126		1.2787536
N21	75	26	1.87506126		1.41497335
N24	75	21	1.87506126		1.32221929
N25	89	41	1.94939001		1.61278386

N27	74	16	1.86923172	1.20411998
N29	85	23	1.92941893	1.36172784
N31	72	19	1.8573325	1.2787536
N32	89	24	1.94939001	1.38021124
N33	93	65	1.96848295	1.81291336
N35	78	24	1.8920946	1.38021124
N36	84	25	1.92427929	1.39794001
N38	87	26	1.93951925	1.41497335

ckd and ctrl

1.9561136
1.92253134
1.77824292
1.70913372
1.99305944
1.78926315
1.93894072
2.10579117
1.93894072
1.75714287
1.9741245
1.84991567
1.76754216
1.9741245
1.68306522
2.01301837
1.84991567
1.92253134
1.8369617
1.84991567
1.8369617
1.7002381
1.7002381
1.64333333
1.77824292
1.84991567
1.65871682
1.8633408
1.42301849
1.37632683
1.40678949
1.36908836
1.40284132
1.46701697
1.47648288
1.49617557
1.35158981
1.43140864
1.40678949
1.39506945
1.39506945
1.49617557
1.41889387
1.48619793
1.48619793
1.48619793
1.42301849

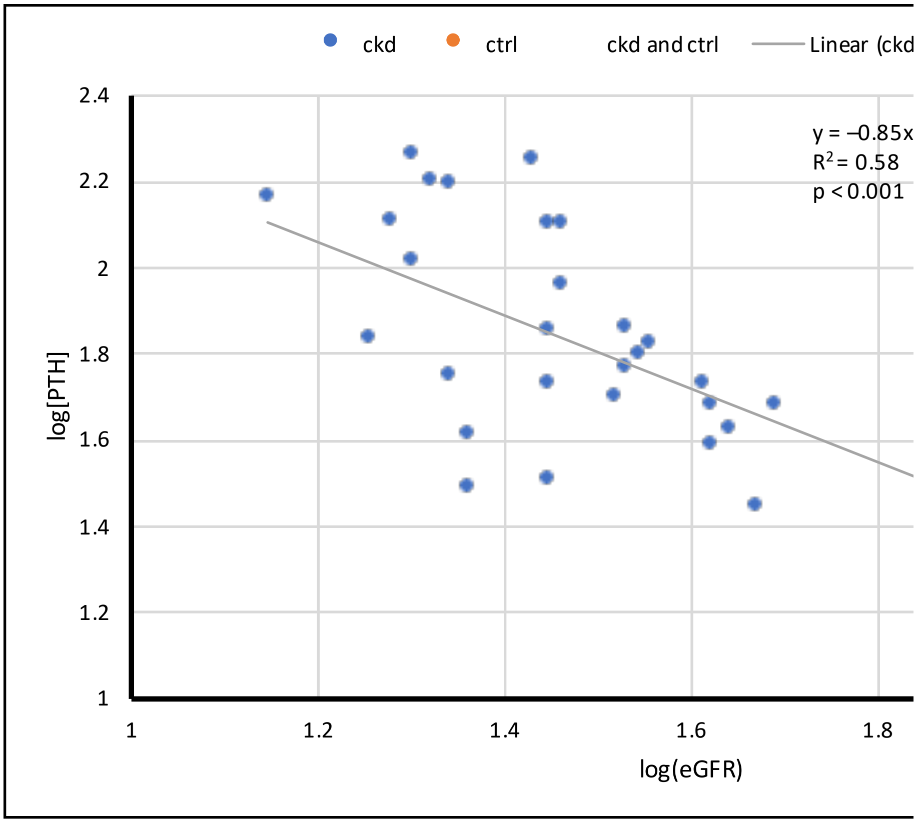

1.49115304
1.43999391
1.50126738
1.42301849
1.40678949
1.47171959
1.44436261
1.43140864

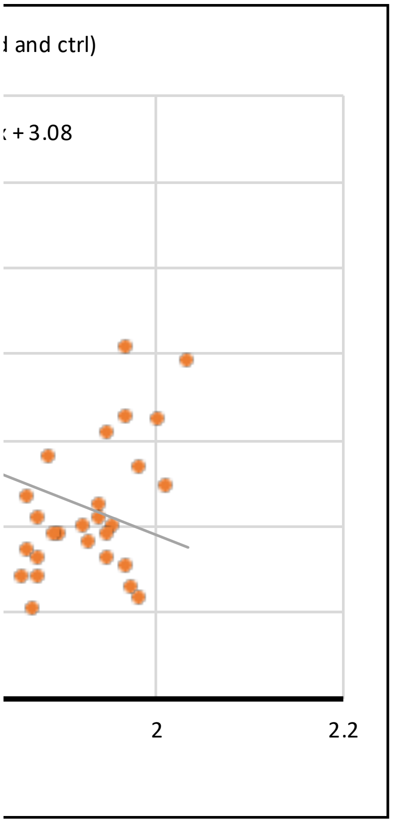

standardized logeGFR	ckd	ctrl
-1.389320853	1.873413854	
-1.239667288	-0.112597096	
-0.596670769	0.423227158	
-0.288697512	0.292860203	
-1.553963633	1.574880374	
-0.645780531	0.179559337	
-1.312792876	0.346400092	
-2.056333198	1.747010177	
-1.312792876	1.854659655	
-0.502642043	0.61042306	
-1.46958335	2.081597925	
-0.916068063	1.540239136	
-0.54898468	0.519798473	
-1.46958335	1.243516016	
-0.172527741	-0.077121049	
-1.642907178	0.653725731	
-0.916068063	0.716381344	
-1.239667288	-0.524197648	
-0.858340917	1.061157262	
-0.916068063	0.292860203	
-0.858340917	1.55187702	
-0.249055718	-0.186221671	
-0.249055718	0.119461822	
0.004530606	0.119461822	
-0.596670769	0.736687661	
-0.916068063	-0.477457701	
-0.064023265	-0.674040572	
-0.975894833	2.048881364	
0.986325783		-1.0975617
1.194399143		-0.008635
1.058647532		0.0244492
1.226656154		-0.5241976
1.07624187		-1.3245
0.790254134		-0.9009788
0.748070929		-0.3040593
0.660313864		-1.0290757
1.304635437		0.44797034
0.948936563		-0.6740406
1.058647532		-1.1693898
1.110875883		-0.3882071
1.110875883		-1.4086477
0.660313864		-0.6223797
1.004706475		-0.8408813
0.704777513		-1.244903
0.704777513		-0.7831412
0.704777513		-1.0975617
0.986325783		-0.1125971

0.682695908	-1.4978984
0.910677749	-0.9636345
0.637623093	-1.244903
0.986325783	-0.9009788
1.058647532	0.56580799
0.769297737	-0.9009788
0.891209417	-0.8408813
0.948936563	-0.7831412

ckd and ctrl
1.05388385
0.94014714
0.45146978
0.21741011
1.17901236
0.4887932
0.99572259
1.56081323
0.99572259
0.38000795
1.11488335
0.69421173
0.41522836
1.11488335
0.12912108
1.24660946
0.69421173
0.94014714
0.6503391
0.69421173
0.6503391
0.18728235
0.18728235
-0.0054433
0.45146978
0.69421173
0.04665768
0.73968007
-0.7516076
-0.9097433
-0.8065721
-0.9342587
-0.8199438
-0.6025931
-0.5705339
-0.5038385
-0.9935229
-0.7231918
-0.8065721
-0.8462657
-0.8462657
-0.5038385
-0.7655769
-0.5376309
-0.5376309
-0.5376309
-0.7516076

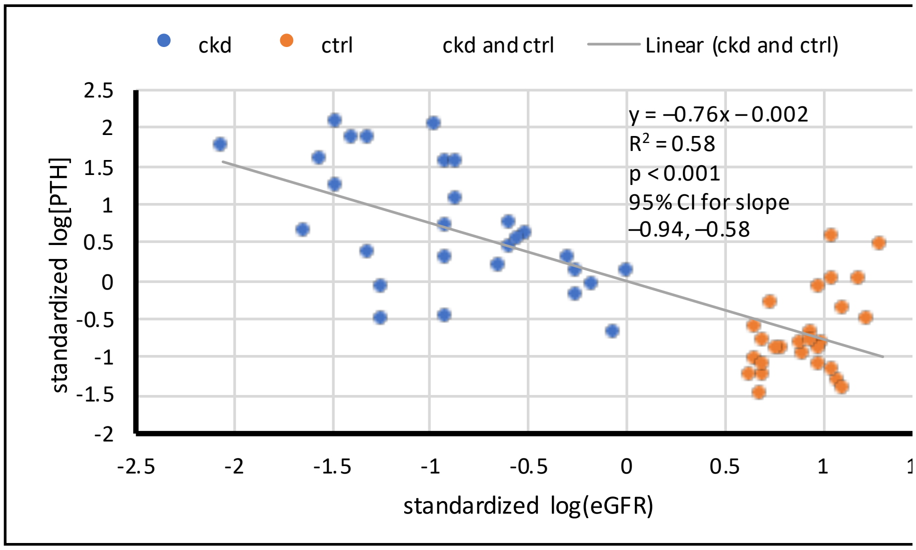

-0.5208489
-0.6941151
-0.4865936
-0.7516076
-0.8065721
-0.5866663
-0.6793192
-0.7231918
