# Supplementary material for: Chemical evidence for the tradeoff-in-the-nephron hypothesis to explain secondary hyperparathyroidism
Source: PLoS One. 2022 Aug 1;17(8):e0272380. doi: 10.1371/journal.pone.0272380 (PMC9342777; doi:10.1371/journal.pone.0272380)
Supplement: S7 File — (PDF) [file pone.0272380.s016.pdf]

code	Ca+2	Ca++ x 10^4	ckd	ctrl	logCa++ x 104
CKD2	0.0002891	2.891	158		0.46104809
CKD4	0.0002891	2.891	41		0.46104809
CKD5	0.0002973	2.973	59		0.47319491
CKD6	0.0002984	2.984	54		0.47479882
CKD7	0.0002722	2.722	129		0.43488812
CKD11	0.0003027	3.027	50		0.48101242
CKD13	0.0002467	2.467	56		0.39216915
CKD14	0.0002776	2.776	145		0.44341946
CKD15	0.0002216	2.216	156		0.34556976
CKD18	0.0003061	3.061	67		0.48586333
CKD20	0.0002473	2.473	182		0.39322412
CKD21	0.0002268	2.268	126		0.35564305
CKD23	0.0002918	2.918	63		0.46508529
CKD24	0.0002161	2.161	103		0.33465477
CKD25	0.0003106	3.106	42		0.49220145
CKD26	0.0002028	2.028	69		0.30706795
CKD27	0.0002449	2.449	72		0.38898879
CKD31	0.0002472	2.472	31		0.39304847
CKD32	0.0002878	2.878	91		0.45909079
CKD33	0.000247	2.47	54		0.39269695
CKD45	0.0002196	2.196	127		0.34163234
CKD46	0.0002842	2.842	39		0.45362407
CKD49	0.0002911	2.911	48		0.46404221
CKD50	0.0003097	3.097	48		0.49094121
CKD51	0.0002671	2.671	73		0.42667389
CKD55	0.0002923	2.923	32		0.46582882
CKD59	0.0003236	3.236	28		0.51000851
CKD62	0.0002274	2.274	178		0.35679046
N2	0.000402	4.02		21	0.60422605
N3	0.0004371	4.371		44	0.64058081
N4	0.0004267	4.267		45	0.63012264
N6	0.0005485	5.485		31	0.73917663
N7	0.0004971	4.971		18	0.69644376
N8	0.0003191	3.191		24	0.5039268
N9	0.0004608	4.608		36	0.66351247
N10	0.0003733	3.733		22	0.57205799
N11	0.0003694	3.694		60	0.56749689
N13	0.0005246	5.246		28	0.71982829
N14	0.0005385	5.385		20	0.73118571
N15	0.0004661	4.661		34	0.6684791
N16	0.0003525	3.525		17	0.54715912
N17	0.0005162	5.162		29	0.712818
N18	0.0003181	3.181		25	0.50256367
N20	0.000404	4.04		19	0.60638137
N21	0.0005143	5.143		26	0.71121652
N24	0.0004487	4.487		21	0.65195607
N25	0.0003754	3.754		41	0.57449427

N27	0.0003975	3.975	16	0.59933713
N29	0.0005587	5.587	23	0.74717867
N31	0.0004724	4.724	19	0.67430989
N32	0.0003823	3.823	24	0.5824043
N33	0.0003198	3.198	65	0.50487846
N35	0.0005522	5.522	24	0.7420964
N36	0.000541	5.41	25	0.73319727
N38	0.0005501	5.501	26	0.74044164

logPTH
2.19865709
1.61278386
1.77085201
1.73239376
2.11058971
1.69897
1.74818803
2.161368
2.1931246
1.8260748
2.26007139
2.10037055
1.79934055
2.01283722
1.62324929
1.83884909
1.8573325
1.49136169
1.95904139
1.73239376
2.10380372
1.59106461
1.68124124
1.68124124
1.86332286
1.50514998
1.44715803
2.25042
1.32221929
1.64345268
1.65321251
1.49136169
1.25527251
1.38021124
1.5563025
1.34242268
1.77815125
1.44715803
1.30103
1.53147892
1.23044892
1.462398
1.39794001
1.2787536
1.41497335
1.32221929
1.61278386

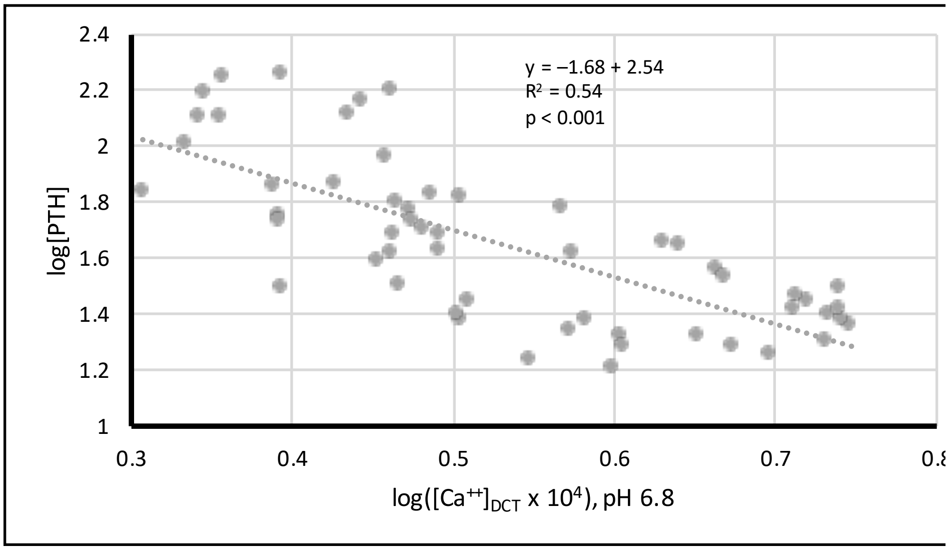

Column1		log Ca++ x 104	Column1	
Mean	0.53294046		Mean	1.64552531
Standard Erro	0.01740821		Standard Erro	0.03975921
Median	0.50256367		Median	1.61278386
Mode	0.46104809		Mode	1.38021124
Standard Devi	0.12910276		Standard Devi	0.29486219
Sample Variar	0.01666752		Sample Variar	0.08694371
Kurtosis	-1.1235188		Kurtosis	-0.6740973
Skewness	0.20873048		Skewness	0.54246903
Range	0.44011072		Range	1.05595141
Minimum	0.30706795		Minimum	1.20411998
Maximum	0.74717867		Maximum	2.26007139
Sum	29.3117252		Sum	90.5038919
Count	55		Count	55

SUMMARY OUTPUT

Regression Statistics	
Multiple R	0.73551584
R Square	0.54098355
Adjusted R Sq	0.53232287
Standard Erro	0.20164718
Observations	55

ANOVA

	df	*SS*	*MS*	*F*
Regression	1	2.539896341	2.53989634	62.4642727
Residual	53	2.15506401	0.04066159	
Total	54	4.694960351		

1.20411998
1.36172784
1.2787536
1.38021124
1.81291336
1.38021124
1.39794001
1.41497335

	Coefficients	*Standard Error*	*t Stat*	*P-value*
Intercept	2.54079585	0.116493717	21.8105827	3.873E-28
X Variable 1	-1.6798697	0.212549341	-7.9034342	1.5964E-10

8

pth

Significance F	
1.5964E-10	

logCa++ x 104	mean
0.461048092	0.533
0.461048092	0.533
0.473194909	0.533
0.474798819	0.533
0.434888121	0.533
0.481012421	0.533
0.392169149	0.533
0.443419462	0.533
0.345569756	0.533
0.48586333	0.533
0.393224116	0.533
0.35564305	0.533
0.465085288	0.533
0.334654767	0.533
0.492201451	0.533
0.307067951	0.533
0.388988785	0.533
0.393048466	0.533
0.45909079	0.533
0.392696953	0.533
0.341632336	0.533
0.453624074	0.533
0.464042205	0.533
0.490941205	0.533
0.426673888	0.533
0.465828815	0.533
0.510008513	0.533
0.35679046	0.533
0.604226053	0.533
0.640580806	0.533
0.630122643	0.533
0.739176632	0.533
0.696443763	0.533
0.503926804	0.533
0.66351247	0.533
0.57205799	0.533
0.567496891	0.533
0.719828286	0.533
0.731185708	0.533
0.668479103	0.533
0.547159121	0.533
0.712818	0.533
0.502563669	0.533
0.606381365	0.533
0.711216524	0.533
0.65195607	0.533
0.574494268	0.533

Lower 95%	*Upper 95%*	*Lower 95.0%*	*Upper 95.0%*
2.30713905	2.77445266	2.30713905	2.77445266
-2.1061897	-1.2535497	-2.1061897	-1.2535497

0.599337133	0.533
0.747178671	0.533
0.674309889	0.533
0.582404298	0.533
0.504878459	0.533
0.742096402	0.533
0.733197265	0.533
0.740441645	0.533

SD	standardized log([Ca++} x 104)	logPTH	mean	SD
0.129	-0.557766731	2.19865709	1.646	0.295
0.129	-0.557766731	1.61278386	1.646	0.295
0.129	-0.463605355	1.77085201	1.646	0.295
0.129	-0.451171947	1.73239376	1.646	0.295
0.129	-0.760557203	2.11058971	1.646	0.295
0.129	-0.403004489	1.69897	1.646	0.295
0.129	-1.091712019	1.74818803	1.646	0.295
0.129	-0.694422777	2.161368	1.646	0.295
0.129	-1.452947627	2.1931246	1.646	0.295
0.129	-0.365400546	1.8260748	1.646	0.295
0.129	-1.083533982	2.26007139	1.646	0.295
0.129	-1.374860076	2.10037055	1.646	0.295
0.129	-0.526470639	1.79934055	1.646	0.295
0.129	-1.537559947	2.01283722	1.646	0.295
0.129	-0.316267819	1.62324929	1.646	0.295
0.129	-1.751411235	1.83884909	1.646	0.295
0.129	-1.116366007	1.8573325	1.646	0.295
0.129	-1.084895609	1.49136169	1.646	0.295
0.129	-0.572939615	1.95904139	1.646	0.295
0.129	-1.087620517	1.73239376	1.646	0.295
0.129	-1.483470265	2.10380372	1.646	0.295
0.129	-0.615317259	1.59106461	1.646	0.295
0.129	-0.534556547	1.68124124	1.646	0.295
0.129	-0.326037168	1.68124124	1.646	0.295
0.129	-0.824233426	1.86332286	1.646	0.295
0.129	-0.520706858	1.50514998	1.646	0.295
0.129	-0.178228582	1.44715803	1.646	0.295
0.129	-1.365965424	2.25042	1.646	0.295
0.129	0.552139946	1.32221929	1.646	0.295
0.129	0.83395974	1.64345268	1.646	0.295
0.129	0.752888704	1.65321251	1.646	0.295
0.129	1.598268464	1.49136169	1.646	0.295
0.129	1.267005916	1.25527251	1.646	0.295
0.129	-0.225373611	1.38021124	1.646	0.295
0.129	1.011724577	1.5563025	1.646	0.295
0.129	0.302775116	1.34242268	1.646	0.295
0.129	0.26741776	1.77815125	1.646	0.295
0.129	1.448281289	1.44715803	1.646	0.295
0.129	1.536323315	1.30103	1.646	0.295
0.129	1.050225604	1.53147892	1.646	0.295
0.129	0.10976063	1.23044892	1.646	0.295
0.129	1.393937986	1.462398	1.646	0.295
0.129	-0.23594055	1.39794001	1.646	0.295
0.129	0.568847792	1.2787536	1.646	0.295
0.129	1.381523444	1.41497335	1.646	0.295
0.129	0.922140074	1.32221929	1.646	0.295
0.129	0.321660994	1.61278386	1.646	0.295

0.129	0.514241341	1.20411998	1.646	0.295
0.129	1.660299778	1.36172784	1.646	0.295
0.129	1.095425496	1.2787536	1.646	0.295
0.129	0.382979054	1.38021124	1.646	0.295
0.129	-0.217996439	1.81291336	1.646	0.295
0.129	1.620902343	1.38021124	1.646	0.295
0.129	1.551916784	1.39794001	1.646	0.295
0.129	1.608074767	1.41497335	1.646	0.295

standardized logPTH	standardized log([Ca++} x 104)	standardized logPTH
1.873413854	-0.557766731	1.873413854
-0.112597096	-0.557766731	-0.112597096
0.423227158	-0.463605355	0.423227158
0.292860203	-0.451171947	0.292860203
1.574880374	-0.760557203	1.574880374
0.179559337	-0.403004489	0.179559337
0.346400092	-1.091712019	0.346400092
1.747010177	-0.694422777	1.747010177
1.854659655	-1.452947627	1.854659655
0.61042306	-0.365400546	0.61042306
2.081597925	-1.083533982	2.081597925
1.540239136	-1.374860076	1.540239136
0.519798473	-0.526470639	0.519798473
1.243516016	-1.537559947	1.243516016
-0.077121049	-0.316267819	-0.077121049
0.653725731	-1.751411235	0.653725731
0.716381344	-1.116366007	0.716381344
-0.524197648	-1.084895609	-0.524197648
1.061157262	-0.572939615	1.061157262
0.292860203	-1.087620517	0.292860203
1.55187702	-1.483470265	1.55187702
-0.186221671	-0.615317259	-0.186221671
0.119461822	-0.534556547	0.119461822
0.119461822	-0.326037168	0.119461822
0.736687661	-0.824233426	0.736687661
-0.477457701	-0.520706858	-0.477457701
-0.674040572	-0.178228582	-0.674040572
2.048881364	-1.365965424	2.048881364
-1.097561713	0.552139946	-1.097561713
-0.008634995	0.83395974	-0.008634995
0.024449199	0.752888704	0.024449199
-0.524197648	1.598268464	-0.524197648
-1.324499983	1.267005916	-1.324499983
-0.900978842	-0.225373611	-0.900978842
-0.304059319	1.011724577	-0.304059319
-1.029075658	0.302775116	-1.029075658
0.44797034	0.26741776	0.44797034
-0.674040572	1.448281289	-0.674040572
-1.169389845	1.536323315	-1.169389845
-0.388207061	1.050225604	-0.388207061
-1.408647724	0.10976063	-1.408647724
-0.622379668	1.393937986	-0.622379668
-0.840881327	-0.23594055	-0.840881327
-1.244903048	0.568847792	-1.244903048
-0.783141193	1.381523444	-0.783141193
-1.097561713	0.922140074	-1.097561713
-0.112597096	0.321660994	-0.112597096

-1.497898364	0.514241341	-1.497898364
-0.963634454	1.660299778	-0.963634454
-1.244903048	1.095425496	-1.244903048
-0.900978842	0.382979054	-0.900978842
0.565807989	-0.217996439	0.565807989
-0.900978842	1.620902343	-0.900978842
-0.840881327	1.551916784	-0.840881327
-0.783141193	1.608074767	-0.783141193

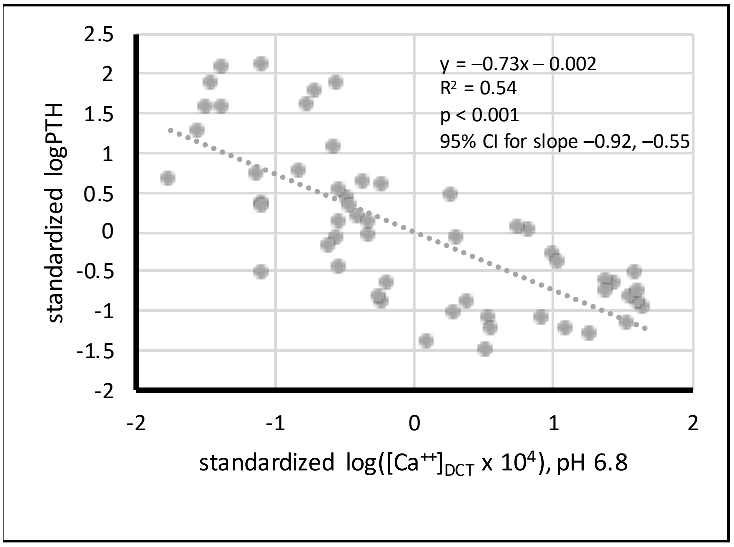

SUMMARY OUTPUT

Regression Statistics	
Multiple R	0.73551584
R Square	0.54098355
Adjusted R Sq	0.53232287
Standard Erro	0.68354976
Observations	55

ANOVA

	df	*SS*	*MS*	*F*	*Significance F*
Regression	1	29.1858241	29.1858241	62.4642727	1.5964E-10
Residual	53	24.7637347	0.46724028		
Total	54	53.9495588			

	Coefficients	*Standard Error*	*t Stat*	*P-value*	*Lower 95%*	*Upper 95%*
Intercept	-0.0019482	0.09216984	-0.0211369	0.98321581	-0.1868175	0.1829211
X Variable 1	-0.7345871	0.0929453	-7.9034342	1.5964E-10	-0.9210118	-0.5481624

Lower 95.0%	*Upper 95.0%*
-0.1868175	0.1829211
-0.9210118	-0.5481624

code	logCa++ x 104	ckd	ctrl	ckd and ctrl
CKD2	0.461048092	2.198657087		1.76543921
CKD4	0.461048092	1.612783857		1.76543921
CKD5	0.473194909	1.770852012		1.74503255
CKD6	0.474798819	1.73239376		1.74233798
CKD7	0.434888121	2.11058971		1.80938796
CKD11	0.481012421	1.698970004		1.73189913
CKD13	0.392169149	1.748188027		1.88115583
CKD14	0.443419462	2.161368002		1.7950553
CKD15	0.345569756	2.193124598		1.95944281
CKD18	0.48586333	1.826074803		1.72374961
CKD20	0.393224116	2.260071388		1.87938348
CKD21	0.35564305	2.100370545		1.94251968
CKD23	0.465085288	1.799340549		1.75865672
CKD24	0.334654767	2.012837225		1.97777999
CKD25	0.492201451	1.62324929		1.71310156
CKD26	0.307067951	1.838849091		2.02412584
CKD27	0.388988785	1.857332496		1.88649884
CKD31	0.393048466	1.491361694		1.87967858
CKD32	0.45909079	1.959041392		1.76872747
CKD33	0.392696953	1.73239376		1.88026912
CKD45	0.341632336	2.103803721		1.96605768
CKD46	0.453624074	1.591064607		1.77791156
CKD49	0.464042205	1.681241237		1.76040909
CKD50	0.490941205	1.681241237		1.71521878
CKD51	0.426673888	1.86332286		1.82318787
CKD55	0.465828815	1.505149978		1.75740759
CKD59	0.510008513	1.447158031		1.6831857
CKD62	0.35679046	2.250420002		1.94059203
N2	0.604226053		1.32221929	1.52490023
N3	0.640580806		1.64345268	1.46382425
N4	0.630122643		1.65321251	1.48139396
N6	0.739176632		1.49136169	1.29818326
N7	0.696443763		1.25527251	1.36997448
N8	0.503926804		1.38021124	1.69340297
N9	0.66351247		1.5563025	1.42529905
N10	0.57205799		1.34242268	1.57894258
N11	0.567496891		1.77815125	1.58660522
N13	0.719828286		1.44715803	1.33068848
N14	0.731185708		1.30103	1.31160801
N15	0.668479103		1.53147892	1.41695511
N16	0.547159121		1.23044892	1.62077268
N17	0.712818		1.462398	1.34246576
N18	0.502563669		1.39794001	1.69569304
N20	0.606381365		1.2787536	1.52127931
N21	0.711216524		1.41497335	1.34515624
N24	0.65195607		1.32221929	1.4447138
N25	0.574494268		1.61278386	1.57484963

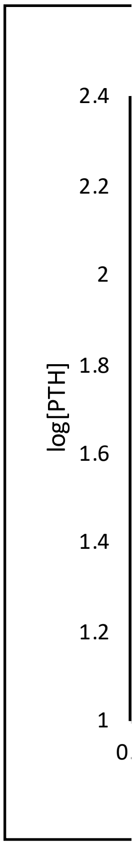

N27	0.599337133	1.20411998	1.53311362
N29	0.747178671	1.36172784	1.28473983
N31	0.674309889	1.2787536	1.40715939
N32	0.582404298	1.38021124	1.56156078
N33	0.504878459	1.81291336	1.69180419
N35	0.742096402	1.38021124	1.29327804
N36	0.733197265	1.39794001	1.30822859
N38	0.740441645	1.41497335	1.29605804

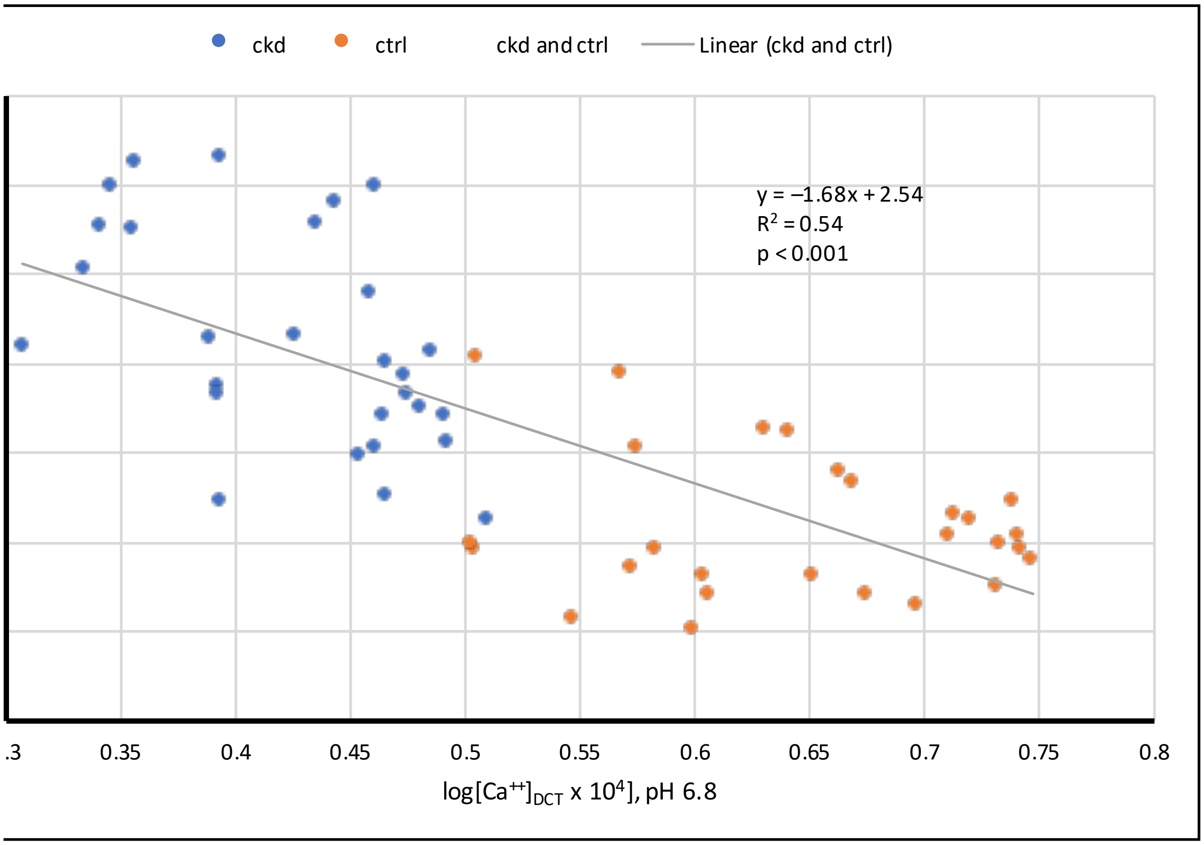
ckd
ctrl
ckd and ctrl
Linear (ckd and ctrl)
y = –1.68x + 2.54
R^2 = 0.54
p < 0.001
.3
0.35
0.4
0.45
0.5
0.55
0.6
0.65
0.7
0.75
0.8
log[Ca^{++}]$_{DCT}$ x 10^4], pH 6.8

standardized log([Ca++} x 104)	ckd	ctrl	ckd and ctrl
-0.557766731	1.873413854		0.40516971
-0.557766731	-0.112597096		0.40516971
-0.463605355	0.423227158		0.33643191
-0.451171947	0.292860203		0.32735552
-0.760557203	1.574880374		0.55320676
-0.403004489	0.179559337		0.29219328
-1.091712019	0.346400092		0.79494977
-0.694422777	1.747010177		0.50492863
-1.452947627	1.854659655		1.05865177
-0.365400546	0.61042306		0.2647424
-1.083533982	2.081597925		0.78897981
-1.374860076	1.540239136		1.00164786
-0.526470639	0.519798473		0.38232357
-1.537559947	1.243516016		1.12041876
-0.316267819	-0.077121049		0.22887551
-1.751411235	0.653725731		1.2765302
-1.116366007	0.716381344		0.81294718
-1.084895609	-0.524197648		0.78997379
-0.572939615	1.061157262		0.41624592
-1.087620517	0.292860203		0.79196298
-1.483470265	1.55187702		1.08093329
-0.615317259	-0.186221671		0.4471816
-0.534556547	0.119461822		0.38822628
-0.326037168	0.119461822		0.23600713
-0.824233426	0.736687661		0.5996904
-0.520706858	-0.477457701		0.37811601
-0.178228582	-0.674040572		0.12810686
-1.365965424	2.048881364		0.99515476
0.552139946		-1.0975617	-0.4050622
0.83395974		-0.008635	-0.6107906
0.752888704		0.0244492	-0.5516088
1.598268464		-0.5241976	-1.168736
1.267005916		-1.3245	-0.9269143
-0.225373611		-0.9009788	0.16252274
1.011724577		-0.3040593	-0.7405589
0.302775116		-1.0290757	-0.2230258
0.26741776		0.44797034	-0.197215
1.448281289		-0.6740406	-1.0592453
1.536323315		-1.1693898	-1.123516
1.050225604		-0.3882071	-0.7686647
0.10976063		-1.4086477	-0.0821253
1.393937986		-0.6223797	-1.0195747
-0.23594055		-0.8408813	0.1702366
0.568847792		-1.244903	-0.4172589
1.381523444		-0.7831412	-1.0105121
0.922140074		-1.0975617	-0.6751623
0.321660994		-0.1125971	-0.2368125

standardized log[PTH]

0.514241341		-1.4978984	-0.3773962
1.660299778		-0.9636345	-1.2140188
1.095425496		-1.244903	-0.8016606
0.382979054		-0.9009788	-0.2815747
-0.217996439		0.56580799	0.1571374
1.620902343		-0.9009788	-1.1852587
1.551916784		-0.8408813	-1.1348993
1.608074767		-0.7831412	-1.1758946
