# Supplementary material for: Chemical evidence for the tradeoff-in-the-nephron hypothesis to explain secondary hyperparathyroidism
Source: PLoS One. 2022 Aug 1;17(8):e0272380. doi: 10.1371/journal.pone.0272380 (PMC9342777; doi:10.1371/journal.pone.0272380)
Supplement: S8 File — (PDF) [file pone.0272380.s017.pdf]

code	Ca++ x 10^4	pth	logCax10^4	logPTh
CKD2	1.922	158	0.28375338	2.19865709
CKD4	1.904	41	0.27966694	1.61278386
CKD5	2.485	59	0.39532639	1.77085201
CKD6	2.137	54	0.32980452	1.73239376
CKD7	1.786	129	0.25188145	2.11058971
CKD11	2.985	50	0.47494434	1.69897
CKD13	1.612	56	0.20736504	1.74818803
CKD14	1.824	145	0.26102483	2.161368
CKD15	1.442	156	0.15896526	2.1931246
CKD18	2.803	67	0.4476231	1.8260748
CKD20	1.616	182	0.20844136	2.26007139
CKD21	1.477	126	0.1693805	2.10037055
CKD23	2.069	63	0.31576049	1.79934055
CKD24	1.406	103	0.14798532	2.01283722
CKD25	2.185	42	0.33945144	1.62324929
CKD26	1.317	69	0.11958577	1.83884909
CKD27	1.599	72	0.20384846	1.8573325
CKD31	1.615	31	0.20817253	1.49136169
CKD32	2.184	91	0.33925263	1.95904139
CKD33	1.614	54	0.20790353	1.73239376
CKD45	1.429	127	0.15503223	2.10380372
CKD46	2.34	39	0.36921586	1.59106461
CKD49	2.449	48	0.38898879	1.68124124
CKD50	3.055	48	0.48501121	1.68124124
CKD51	1.751	73	0.24328615	1.86332286
CKD55	2.886	32	0.46029633	1.50514998
CKD59	3.198	28	0.50487846	1.44715803
CKD62	1.481	178	0.17055506	2.25042
N2	2.73	21	0.43616265	1.32221929
N3	3.005	44	0.47784448	1.64345268
N4	2.923	45	0.46582882	1.65321251
N6	4.387	31	0.64216763	1.49136169
N7	3.498	18	0.54381981	1.25527251
N8	2.115	24	0.32531037	1.38021124
N9	3.196	36	0.50460677	1.5563025
N10	2.511	22	0.39984671	1.34242268
N11	2.483	60	0.39497672	1.77815125
N13	3.971	28	0.59889989	1.44715803
N14	3.856	20	0.58613703	1.30103
N15	3.24	34	0.51054501	1.53147892
N16	2.357	17	0.37235958	1.23044892
N17	3.661	29	0.56359973	1.462398
N18	2.108	25	0.32387061	1.39794001
N20	2.745	19	0.43854235	1.2787536
N21	3.876	26	0.58838377	1.41497335
N24	3.098	21	0.49108141	1.32221929
N25	2.527	41	0.40260524	1.61278386

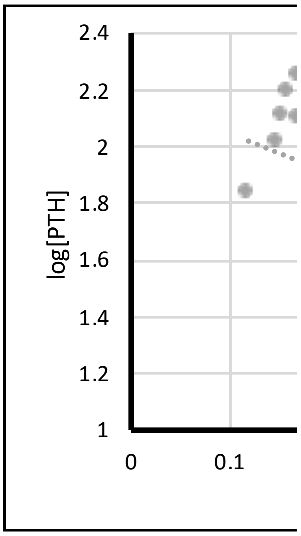

SUMMARY OU

Regression

Multiple R
R Square
Adjusted R Sq
Standard Erro
Observations

ANOVA

Regression
Residual
Total

Intercept
X Variable 1

N27	2.695	16	0.43055877	1.20411998
N29	4.337	23	0.63718942	1.36172784
N31	3.292	19	0.51745983	1.2787536
N32	2.58	24	0.41161971	1.38021124
N33	2.12	65	0.32633586	1.81291336
N35	5.046	24	0.70294725	1.38021124
N36	3.878	25	0.5886078	1.39794001
N38	4.652	26	0.66763971	1.41497335

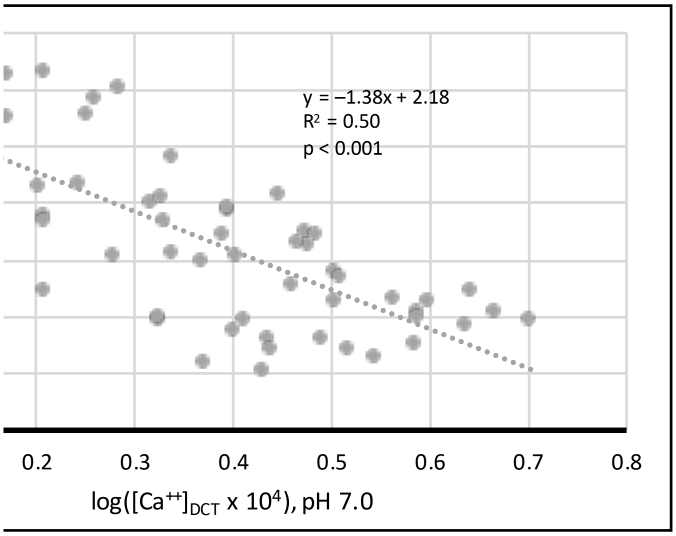

TPUT

Statistics
0.70958062
0.50350466
0.49413683
0.20971796
55

df	*SS*	*MS*	*F*	*Significance F*
1	2.363934429	2.36393443	53.7482331	1.3214E-09
53	2.331025922	0.04398162		
54	4.694960351			

Coefficients	*Standard Error*	*t Stat*	*P-value*	*Lower 95%*	*Upper 95%*	*Lower 95.0%*
2.18483582	0.078810627	27.7226042	3.2159E-33	2.02676172	2.34290992	2.02676172
-1.3811509	0.188390517	-7.3313186	1.3214E-09	-1.7590144	-1.0032874	-1.7590144

Ca++ x 10^4	log Ca++ x 10^4	mean log Ca++	SD	standardized ([illegible]
1.922	0.283753383	0.39	0.15	-0.7083108
1.904	0.279666944	0.39	0.15	-0.7355537
2.485	0.395326393	0.39	0.15	0.03550929
2.137	0.329804522	0.39	0.15	-0.4013032
1.786	0.251881455	0.39	0.15	-0.9207903
2.985	0.474944335	0.39	0.15	0.56629557
1.612	0.207365037	0.39	0.15	-1.2175664
1.824	0.261024834	0.39	0.15	-0.8598344
1.442	0.15896526	0.39	0.15	-1.5402316
2.803	0.447623098	0.39	0.15	0.38415399
1.616	0.208441356	0.39	0.15	-1.210391
1.477	0.169380495	0.39	0.15	-1.4707967
2.069	0.315760491	0.39	0.15	-0.4949301
1.406	0.147985321	0.39	0.15	-1.6134312
2.185	0.339451441	0.39	0.15	-0.3369904
1.317	0.119585775	0.39	0.15	-1.8027615
1.599	0.203848464	0.39	0.15	-1.2410102
1.615	0.208172527	0.39	0.15	-1.2121832
2.184	0.339252634	0.39	0.15	-0.3383158
1.614	0.20790353	0.39	0.15	-1.2139765
1.429	0.155032229	0.39	0.15	-1.5664518
2.34	0.369215857	0.39	0.15	-0.138561
2.449	0.388988785	0.39	0.15	-0.0067414
3.055	0.485011215	0.39	0.15	0.6334081
1.751	0.243286146	0.39	0.15	-0.9780924
2.886	0.460296327	0.39	0.15	0.46864218
3.198	0.504878459	0.39	0.15	0.7658564
1.481	0.170555059	0.39	0.15	-1.4629663
2.73	0.436162647	0.39	0.15	0.30775098
3.005	0.477844476	0.39	0.15	0.58562984
2.923	0.465828815	0.39	0.15	0.50552544
4.387	0.642167634	0.39	0.15	1.68111756
3.498	0.543819805	0.39	0.15	1.02546537
2.115	0.325310372	0.39	0.15	-0.4312642
3.196	0.504606771	0.39	0.15	0.76404514
2.511	0.399846713	0.39	0.15	0.06564475
2.483	0.39497672	0.39	0.15	0.03317813
3.971	0.598899887	0.39	0.15	1.39266591
3.856	0.586137025	0.39	0.15	1.30758017
3.24	0.51054501	0.39	0.15	0.8036334
2.357	0.372359583	0.39	0.15	-0.1176028
3.661	0.563599729	0.39	0.15	1.15733153
2.108	0.323870607	0.39	0.15	-0.4408626
2.745	0.438542349	0.39	0.15	0.32361566
3.876	0.588383768	0.39	0.15	1.32255846
3.098	0.491081413	0.39	0.15	0.67387609
2.527	0.402605242	0.39	0.15	0.08403495

Upper 95.0%
2.342909917
-1.003287388

2.695	0.43055877	0.39	0.15	0.2703918
4.337	0.637189422	0.39	0.15	1.64792948
3.292	0.517459827	0.39	0.15	0.84973218
2.58	0.411619706	0.39	0.15	0.14413137
2.12	0.326335861	0.39	0.15	-0.4244276
5.046	0.702947246	0.39	0.15	2.08631497
3.878	0.588607805	0.39	0.15	1.32405203
4.652	0.667639706	0.39	0.15	1.85093137

Column1	
Mean	0.39047906
Standard Erro	0.020426707
Median	0.395326393
Mode	#N/A
Standard Devi	0.151488511
Sample Variar	0.022948769
Kurtosis	-0.859904702
Skewness	0.058257104
Range	0.583361471
Minimum	0.119585775
Maximum	0.702947246
Sum	21.47634828
Count	55

Ca++ x 104	pth	logpth	mean	SD	standardized logPTH
	158	2.19865709	1.65	0.29	1.891920989
	41	1.61278386	1.65	0.29	-0.128331529
	59	1.77085201	1.65	0.29	0.416731075
	54	1.73239376	1.65	0.29	0.284116413
	129	2.11058971	1.65	0.29	1.58824038
	50	1.69897	1.65	0.29	0.168862084
	56	1.74818803	1.65	0.29	0.338579403
	145	2.161368	1.65	0.29	1.763337939
	156	2.1931246	1.65	0.29	1.872843443
	67	1.8260748	1.65	0.29	0.607154492
	182	2.26007139	1.65	0.29	2.103694441
	126	2.10037055	1.65	0.29	1.55300188
	63	1.79934055	1.65	0.29	0.514967412
	103	2.01283722	1.65	0.29	1.251162844
	42	1.62324929	1.65	0.29	-0.092243826
	69	1.83884909	1.65	0.29	0.651203761
	72	1.8573325	1.65	0.29	0.714939643
	31	1.49136169	1.65	0.29	-0.547028642
	91	1.95904139	1.65	0.29	1.065659974
	54	1.73239376	1.65	0.29	0.284116413
	127	2.10380372	1.65	0.29	1.564840417
	39	1.59106461	1.65	0.29	-0.203225493
	48	1.68124124	1.65	0.29	0.107728405
	48	1.68124124	1.65	0.29	0.107728405
	73	1.86332286	1.65	0.29	0.735596069
	32	1.50514998	1.65	0.29	-0.499482833
	28	1.44715803	1.65	0.29	-0.699455064
	178	2.25042	1.65	0.29	2.070413801
	21	1.32221929	1.65	0.29	-1.130278294
	44	1.64345268	1.65	0.29	-0.022576978
	45	1.65321251	1.65	0.29	0.011077634
	31	1.49136169	1.65	0.29	-0.547028642
	18	1.25527251	1.65	0.29	-1.361129293
	24	1.38021124	1.65	0.29	-0.930306063
	36	1.5563025	1.65	0.29	-0.323094825
	22	1.34242268	1.65	0.29	-1.060611445
	60	1.77815125	1.65	0.29	0.441900863
	28	1.44715803	1.65	0.29	-0.699455064
	20	1.30103	1.65	0.29	-1.203344843
	34	1.53147892	1.65	0.29	-0.40869339
	17	1.23044892	1.65	0.29	-1.446727857
	29	1.462398	1.65	0.29	-0.646903456
	25	1.39794001	1.65	0.29	-0.869172384
	19	1.2787536	1.65	0.29	-1.280159997
	26	1.41497335	1.65	0.29	-0.810436731
	21	1.32221929	1.65	0.29	-1.130278294
	41	1.61278386	1.65	0.29	-0.128331529

16	1.20411998	1.65	0.29	-1.537517301
23	1.36172784	1.65	0.29	-0.994041945
19	1.2787536	1.65	0.29	-1.280159997
24	1.38021124	1.65	0.29	-0.930306063
65	1.81291336	1.65	0.29	0.561770195
24	1.38021124	1.65	0.29	-0.930306063
25	1.39794001	1.65	0.29	-0.869172384
26	1.41497335	1.65	0.29	-0.810436731

Column1	
Mean	1.64552531
Standard Erro	0.03975921
Median	1.61278386
Mode	1.38021124
Standard Devi	0.29486219
Sample Variar	0.08694371
Kurtosis	-0.6740973
Skewness	0.54246903
Range	1.05595141
Minimum	1.20411998
Maximum	2.26007139
Sum	90.5038919
Count	55

standardized Ca++ x 104	standardized logPTH
-0.708310778	1.891920989
-0.735553706	-0.128331529
0.035509287	0.416731075
-0.401303186	0.284116413
-0.920790303	1.58824038
0.56629557	0.168862084
-1.217566417	0.338579403
-0.85983444	1.763337939
-1.540231597	1.872843443
0.384153985	0.607154492
-1.210390957	2.103694441
-1.470796698	1.55300188
-0.494930062	0.514967412
-1.613431195	1.251162844
-0.336990391	-0.092243826
-1.8027615	0.651203761
-1.241010242	0.714939643
-1.212183156	-0.547028642
-0.338315773	1.065659974
-1.213976464	0.284116413
-1.566451808	1.564840417
-0.138560951	-0.203225493
-0.006741433	0.107728405
0.633408097	0.107728405
-0.978092359	0.735596069
0.468642178	-0.499482833
0.765856396	-0.699455064
-1.462966277	2.070413801
0.30775098	-1.130278294
0.585629842	-0.022576978
0.505525436	0.011077634
1.681117563	-0.547028642
1.025465368	-1.361129293
-0.431264189	-0.930306063
0.764045138	-0.323094825
0.065644751	-1.060611445
0.03317813	0.441900863
1.392665914	-0.699455064
1.307580168	-1.203344843
0.803633401	-0.40869339
-0.117602783	-1.446727857
1.157331526	-0.646903456
-0.440862623	-0.869172384
0.323615659	-1.280159997
1.322558456	-0.810436731
0.673876089	-1.130278294
0.084034946	-0.128331529

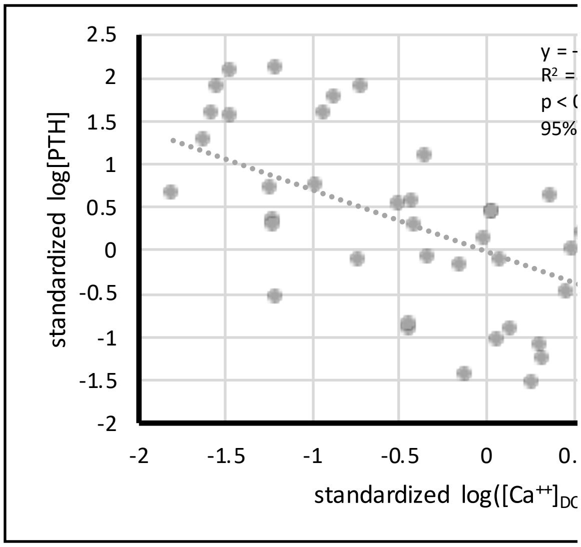

SUMMARY OUTPUT

Regression Statistics	
Multiple R	0.70958062
R Square	0.50350466
Adjusted R Sq	0.49413683
Standard Erro	0.72316537
Observations	55

ANOVA

	df	*SS*
Regression	1	28.1086139
Residual	53	27.7173118
Total	54	55.8259257

	Coefficients	*Standard Error*
Intercept	-0.0131484	0.09751209
X Variable 1	-0.7143884	0.09744337

0.270391797	-1.537517301
1.647929481	-0.994041945
0.849732177	-1.280159997
0.144131373	-0.930306063
-0.424427594	0.561770195
2.086314975	-0.930306063
1.324052032	-0.869172384
1.850931374	-0.810436731

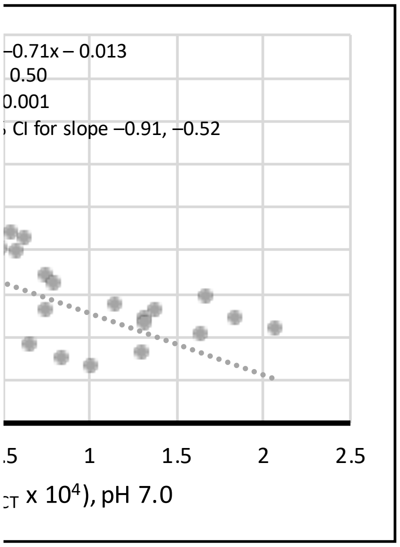

MS	F	Significance F
28.1086139	53.7482331	1.3214E-09
0.52296815		

t Stat	P-value	Lower 95%	Upper 95.0%
-0.1348388	0.89325014	-0.2087329	0.18243608
-7.3313186	1.3214E-09	-0.9098351	-0.5189418

code	logCax10^4	ckd	ctrl	ckd and ctrl
CKD2	0.28375338	2.19865709		1.78842033
CKD4	0.27966694	1.61278386		1.79405962
CKD5	0.39532639	1.77085201		1.63444958
CKD6	0.32980452	1.73239376		1.72486976
CKD7	0.25188145	2.11058971		1.83240359
CKD11	0.47494434	1.69897		1.52457682
CKD13	0.20736504	1.74818803		1.89383625
CKD14	0.26102483	2.161368		1.81978573
CKD15	0.15896526	2.1931246		1.96062794
CKD18	0.4476231	1.8260748		1.56228013
CKD20	0.20844136	2.26007139		1.89235093
CKD21	0.1693805	2.10037055		1.94625492
CKD23	0.31576049	1.79934055		1.74425052
CKD24	0.14798532	2.01283722		1.97578026
CKD25	0.33945144	1.62324929		1.71155701
CKD26	0.11958577	1.83884909		2.01497163
CKD27	0.20384846	1.8573325		1.89868912
CKD31	0.20817253	1.49136169		1.89272191
CKD32	0.33925263	1.95904139		1.71183137
CKD33	0.20790353	1.73239376		1.89309313
CKD45	0.15503223	2.10380372		1.96605552
CKD46	0.36921586	1.59106461		1.67048212
CKD49	0.38898879	1.68124124		1.64319548
CKD50	0.48501121	1.68124124		1.51068452
CKD51	0.24328615	1.86332286		1.84426512
CKD55	0.46029633	1.50514998		1.54479107
CKD59	0.50487846	1.44715803		1.48326773
CKD62	0.17055506	2.25042		1.94463402
N2	0.43616265		1.32221929	1.57809555
N3	0.47784448		1.64345268	1.52057462
N4	0.46582882		1.65321251	1.53715623
N6	0.64216763		1.49136169	1.29380866
N7	0.54381981		1.25527251	1.42952867
N8	0.32531037		1.38021124	1.73107169
N9	0.50460677		1.5563025	1.48364266
N10	0.39984671		1.34242268	1.62821154
N11	0.39497672		1.77815125	1.63493213
N13	0.59889989		1.44715803	1.35351816
N14	0.58613703		1.30103	1.37113091
N15	0.51054501		1.53147892	1.47544789
N16	0.37235958		1.23044892	1.66614378
N17	0.56359973		1.462398	1.40223237
N18	0.32387061		1.39794001	1.73305856
N20	0.43854235		1.2787536	1.57481156
N21	0.58838377		1.41497335	1.3680304
N24	0.49108141		1.32221929	1.50230765
N25	0.40260524		1.61278386	1.62440477

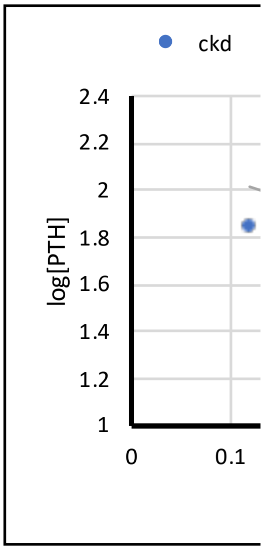

N27	0.43055877	1.20411998	1.5858289
N29	0.63718942	1.36172784	1.3006786
N31	0.51745983	1.2787536	1.46590544
N32	0.41161971	1.38021124	1.61196481
N33	0.32633586	1.81291336	1.72965651
N35	0.70294725	1.38021124	1.2099328
N36	0.5886078	1.39794001	1.36772123
N38	0.66763971	1.41497335	1.25865721

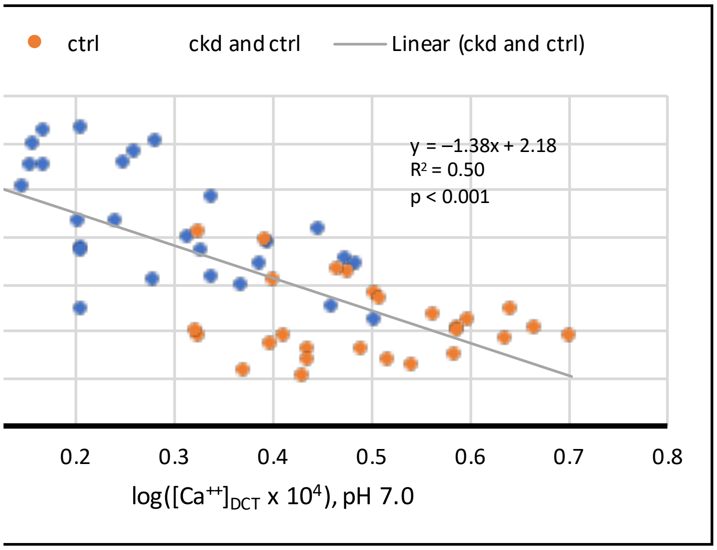

standardized Ca++ x 104
-0.708310778
-0.735553706
0.035509287
-0.401303186
-0.920790303
0.56629557
-1.217566417
-0.85983444
-1.540231597
0.384153985
-1.210390957
-1.470796698
-0.494930062
-1.613431195
-0.336990391
-1.8027615
-1.241010242
-1.212183156
-0.338315773
-1.213976464
-1.566451808
-0.138560951
-0.006741433
0.633408097
-0.978092359
0.468642178
0.765856396
-1.462966277
0.30775098
0.585629842
0.505525436
1.681117563
1.025465368
-0.431264189
0.764045138
0.065644751
0.03317813
1.392665914
1.307580168
0.803633401
-0.117602783
1.157331526
-0.440862623
0.323615659
1.322558456
0.673876089
0.084034946

0.270391797
1.647929481
0.849732177
0.144131373
-0.424427594
2.086314975
1.324052032
1.850931374

ckd	ctrl	ckd and ctrl
1.891920989		0.48990065
-0.128331529		0.50924313
0.416731075		-0.0382116
0.284116413		0.27192526
1.58824038		0.64076112
0.168862084		-0.4150699
0.338579403		0.85147216
1.763337939		0.59748245
1.872843443		1.08056443
0.607154492		-0.2857493
2.103694441		0.84637758
1.55300188		1.03126566
0.514967412		0.33840034
1.251162844		1.13253615
-0.092243826		0.22626318
0.651203761		1.26696067
0.714939643		0.86811727
-0.547028642		0.84765004
1.065659974		0.2272042
0.284116413		0.84892329
1.564840417		1.09918078
-0.203225493		0.08537827
0.107728405		-0.0082136
0.107728405		-0.4627197
0.735596069		0.68144558
-0.499482833		-0.3457359
-0.699455064		-0.556758
2.070413801		1.02570606
	-1.1302783	-0.2315032
	-0.022577	-0.4287972
	0.01107763	-0.3719231
	-0.5470286	-1.2065935
	-1.3611293	-0.7410804
	-0.9303061	0.29319757
	-0.3230948	-0.555472
	-1.0606114	-0.0596078
	0.44190086	-0.0365565
	-0.6994551	-1.0017928
	-1.2033448	-0.9413819
	-0.4086934	-0.5835797
	-1.4467279	0.07049798
	-0.6469035	-0.8347054
	-0.8691724	0.30001246
	-1.28016	-0.2427671
	-0.8104367	-0.9520165
	-1.1302783	-0.491452
	-0.1283315	-0.0726648

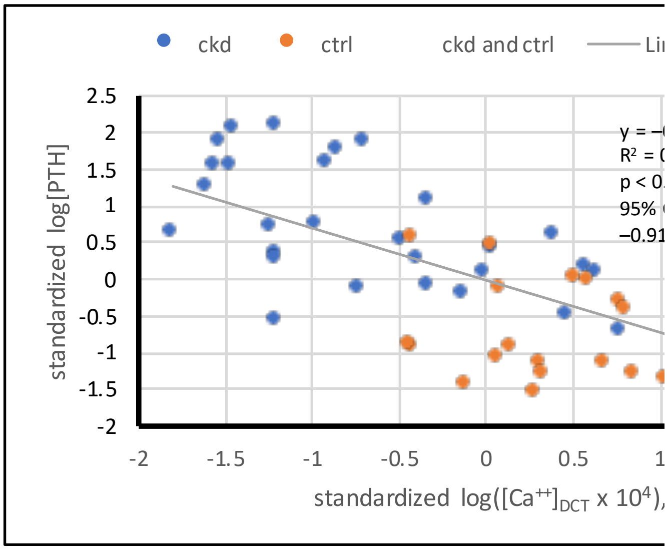

-1.5375173	-0.2049782
-0.9940419	-1.1830299
-1.28016	-0.6163098
-0.9303061	-0.1153333
0.5617702	0.28834359
-0.9303061	-1.4942836
-0.8691724	-0.9530769
-0.8104367	-1.3271613
