# Supplementary material for: Chemical evidence for the tradeoff-in-the-nephron hypothesis to explain secondary hyperparathyroidism
Source: PLoS One. 2022 Aug 1;17(8):e0272380. doi: 10.1371/journal.pone.0272380 (PMC9342777; doi:10.1371/journal.pone.0272380)
Supplement: S9 File — (PDF) [file pone.0272380.s018.pdf]

	Tot(P)	Ca+2	tot P x 1000	Ca++ x 10^4
CKD2	0.001821	0.0002013	1.821	2.013
CKD4	0.001254	0.0002615	1.254	2.615
CKD5	0.001219	0.0002655	1.219	2.655
CKD6	0.001551	0.0002258	1.551	2.258
CKD7	0.002037	0.0001861	2.037	1.861
CKD11	0.0008242	0.0003423	0.8242	3.423
CKD13	0.002377	0.0001668	2.377	1.668
CKD14	0.001974	0.0001902	1.974	1.902
CKD15	0.002806	0.0001483	2.806	1.483
CKD18	0.001004	0.0003036	1.004	3.036
CKD20	0.002393	0.0001678	2.393	1.678
CKD21	0.00272	0.0001523	2.72	1.523
CKD23	0.001626	0.0002178	1.626	2.178
CKD24	0.002937	0.0001447	2.937	1.447
CKD25	0.001508	0.0002317	1.508	2.317
CKD26	0.003254	0.0001353	3.254	1.353
CKD27	0.002416	0.0001657	2.416	1.657
CKD31	0.002404	0.000168	2.404	1.68
CKD32	0.00149	0.0002307	1.49	2.307
CKD33	0.002373	0.000167	2.373	1.67
CKD45	0.002876	0.0001473	2.876	1.473
CKD46	0.001332	0.0002482	1.332	2.482
CKD49	0.001243	0.000261	1.243	2.61
CKD50	0.0007581	0.000362	0.7581	3.62
CKD51	0.002105	0.0001824	2.105	1.824
CKD55	0.0007147	0.0003684	0.7147	3.684
CKD59	0.0006197	0.0004112	0.6197	4.112
CKD62	0.002704	0.0001526	2.704	1.526

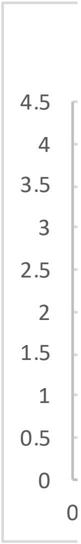

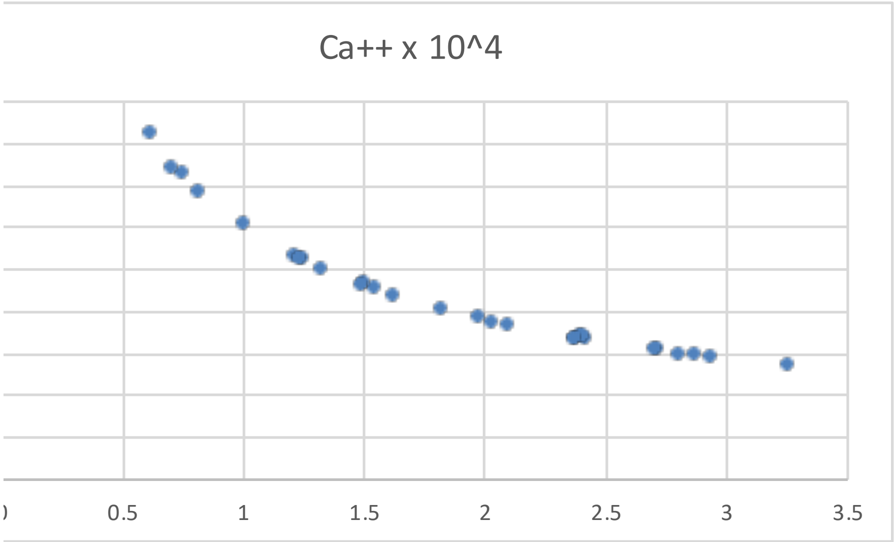

tot P x 1000
1.821
1.254
1.219
1.551
2.037
0.8242
2.377
1.974
2.806
1.004
2.393
2.72
1.626
2.937
1.508
3.254
2.416
2.404
1.49
2.373
2.876
1.332
1.243
0.7581
2.105
0.7147
0.6197
2.704

Ca++ x 10^4	log tot P x 1000	log Ca++
2.013	0.260309946	0.30384377
2.615	0.098297536	0.41747169
2.655	0.086003706	0.42406453
2.258	0.190611798	0.35372394
1.861	0.308991029	0.26974637
3.423	-0.08396739	0.5344069
1.668	0.376029182	0.22219605
1.902	0.295347148	0.27921051
1.483	0.448087667	0.17114115
3.036	0.001733713	0.48230177
1.678	0.378942699	0.22479196
1.523	0.434568904	0.1826999
2.178	0.211120541	0.33805788
1.447	0.467903947	0.16046853
2.317	0.178401342	0.36492603
1.353	0.512417549	0.1312978
1.657	0.38309693	0.21932251
1.68	0.380934463	0.22530928
2.307	0.173186268	0.36304759
1.67	0.375297738	0.22271647
1.473	0.458788882	0.16820275
2.482	0.124504225	0.39480178
2.61	0.094471129	0.41664051
3.62	-0.120273503	0.55870857
1.824	0.3232521	0.26102483
3.684	-0.145876218	0.56631962
4.112	-0.207818504	0.61405311
1.526	0.432006687	0.18355453

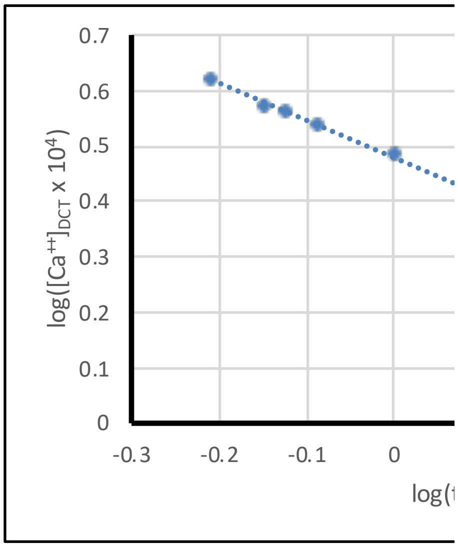

SUMMARY OUTPUT

Regression Statistics	
Multiple R	0.9996531
R Square	0.99930632
Adjusted R Sq	0.99927964
Standard Erro	0.00370884
Observations	28

ANOVA

	df
Regression	1
Residual	26
Total	27

	Coefficients
Intercept	0.47846561
X Variable 1	-0.6747572

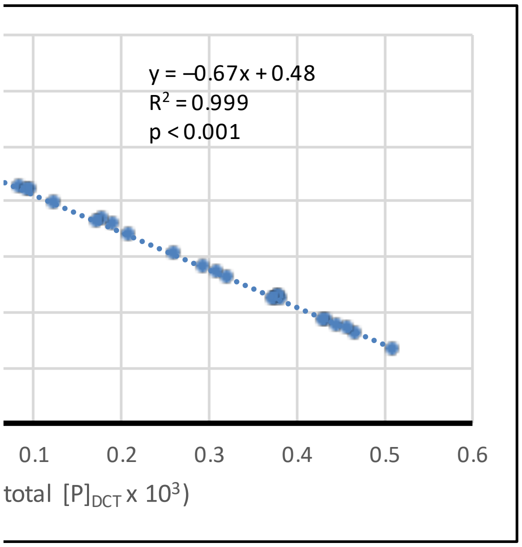

SS	MS	F	Significance F
0.5152144	0.5152144	37455.1642	1.3351E-42
0.00035764	1.3755E-05		
0.51557204			

Standard Error	t Stat	P-value	Lower 95%	Upper 95%	Lower 95.0%	Upper 95.0%
0.0010647	449.39039	4.1306E-52	0.47627709	0.48065413	0.47627709	0.48065413
0.00348652	-193.53337	1.3351E-42	-0.6819238	-0.6675906	-0.6819238	-0.6675906
