# Supplementary material for: Chemical evidence for the tradeoff-in-the-nephron hypothesis to explain secondary hyperparathyroidism
Source: PLoS One. 2022 Aug 1;17(8):e0272380. doi: 10.1371/journal.pone.0272380 (PMC9342777; doi:10.1371/journal.pone.0272380)
Supplement: S10 File — (PDF) [file pone.0272380.s019.pdf]

	Ca+2	Ca++ x 10^4	pth 1-84
CKD2	0.0002116	2.116	158
CKD4	0.0002814	2.814	41
CKD5	0.0002857	2.857	59
CKD6	0.0002398	2.398	54
CKD7	0.0001946	1.946	129
CKD11	0.0003777	3.777	50
CKD13	0.0001731	1.731	56
CKD14	0.0001992	1.992	145
CKD15	0.0001527	1.527	156
CKD18	0.0003315	3.315	67
CKD20	0.0001749	1.749	182
CKD21	0.0001574	1.574	126
CKD23	0.0002304	2.304	63
CKD24	0.0001492	1.492	103
CKD25	0.0002473	2.473	42
CKD26	0.0001392	1.392	69
CKD27	0.0001721	1.721	72
CKD31	0.0001753	1.753	31
CKD32	0.0002448	2.448	91
CKD33	0.0001733	1.733	54
CKD45	0.0001522	1.522	127
CKD46	0.0002647	2.647	39
CKD49	0.00028	2.8	48
CKD50	0.0004026	4.026	48
CKD51	0.0001906	1.906	73
CKD55	0.0004079	4.079	32
CKD59	0.0004652	4.652	28
CKD62	0.0001576	1.576	178

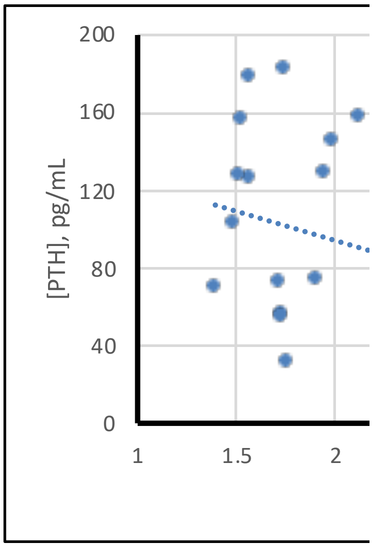

SUMMARY OUTPUT

Regression Statistics	
Multiple R	0.56560149
R Square	0.31990504
Adjusted R Sq	0.29374755
Standard Erro	40.0427282
Observations	28

ANOVA

	df
Regression	1
Residual	26
Total	27

	Coefficients
Intercept	154.448333
X Variable 1	-30.210394

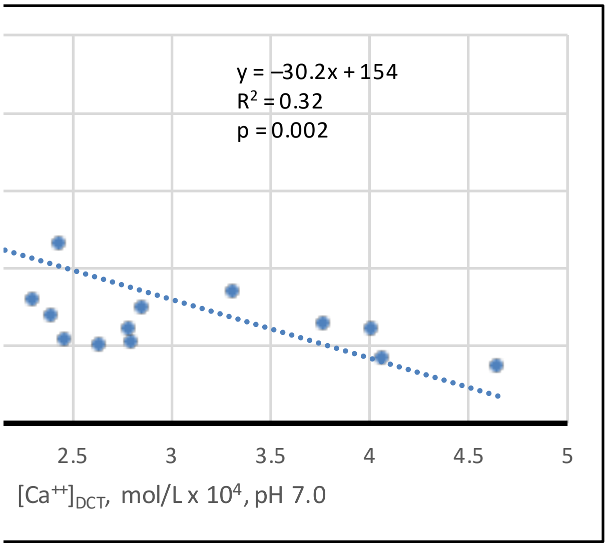

SS	MS	F	Significance F
19609.7565	19609.7565	12.2299556	0.0017086
41688.9221	1603.42008		
61298.6786			

Standard Error	t Stat	P-value	Lower 95%	Upper 95%	Lower 95.0%	Upper 95.0%
21.815692	7.07968982	1.6155E-07	109.605535	199.29113	109.605535	199.29113
8.63861153	-3.4971353	0.0017086	-47.967314	-12.453473	-47.967314	-12.453473
